# Supplementary material for: Insights into substitution strategy towards thermodynamic and property regulation of chemically recyclable polymers
Source: Nat Commun. 2023 Jun 2;14:3198. doi: 10.1038/s41467-023-38916-5 (PMC10238376; doi:10.1038/s41467-023-38916-5)
Supplement: Supplementary file 1 — Supplementary Information [file 41467_2023_38916_MOESM1_ESM.pdf]

Supplementary Information for

Insights into Substitution Strategy Towards Thermodynamic and Property Regulation of Chemically Recyclable Polymers

Yi-Min Tu, Fu-Long Gong, Yan-Chen Wu, Zhongzheng Cai,* and Jian-Bo Zhu*

National Engineering Laboratory of Eco-Friendly Polymeric Materials (Sichuan), College of Chemistry, Sichuan University, 29 Wangjiang Road, Chengdu, 610064, P. R. China

Table of Contents

General Preparations 1
General catalyst synthesis procedure 1
General monomer synthesis procedure 2
NMR spectra of produced monomers 8
General polymerization procedures 35
Polymerization of monomers 35
Monomer conversion monitoring in polymerization process 37
NMR Spectra of Produced Polymers 38
MALDI-TOF Spectra of Polymer 70
Thermal Properties of Polymers 71
Differential Scanning Calorimetry 87
SEC Traces of Polymers 103
Thermodynamic Study 114
Chemical Recycling to Monomer (CRM) 133
General procedure for the CRM of polymers in dilute solutions 133
General procedure for the CRM of polymers under bulk thermal. 150
Mechanical Property 153
Summary of Mechanical Properties 153
Tensile testing of elastomers 161
Supplementary References 164

General Preparations

General synthetic procedure of catalysts

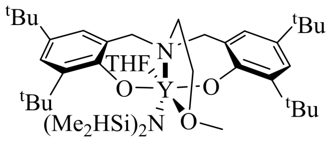

Y-1

The catalyst **Y-1** was prepared according to the reported procedure, and its spectral data is in agreement with the literature.[1] ^{1}H NMR (400 MHz, Benzene-*d*^{6}) δ 7.60 (d, *J* = 2.6 Hz, 2H), 7.09 (d, *J* = 2.5 Hz, 2H), 5.20–5.10 (m, 2H), 3.83 (s, 6H), 3.01 (d, *J* = 12.6 Hz, 2H), 2.85 (s, 3H), 2.70 (t, *J* = 5.4 Hz, 2H), 2.29 (t, *J* = 5.5 Hz, 2H), 1.79 (s, 18H), 1.46 (s, 18H), 1.17 (s, 4H), 0.50 (d, *J* = 3.0 Hz, 12H).

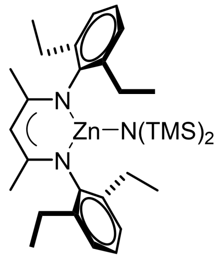

Zn-1

The catalyst **Zn-1** was prepared according to the reported procedure, and its spectral data is in agreement with the literature.[2] ^{1}H NMR (400 MHz, Benzene-*d*^{6}) δ 7.20 (t, *J* = 1.3 Hz, 3H), 7.16 (s, 3H), 4.93 (s, 1H), 2.80 (dq, *J* = 15.3, 7.6 Hz, 4H), 2.65 (dq, *J* = 15.2, 7.5 Hz, 4H), 1.63 (s, 6H), 1.26 (t, *J* = 7.5 Hz, 12H), 0.02 (s, 18H).

General monomer synthetic procedure

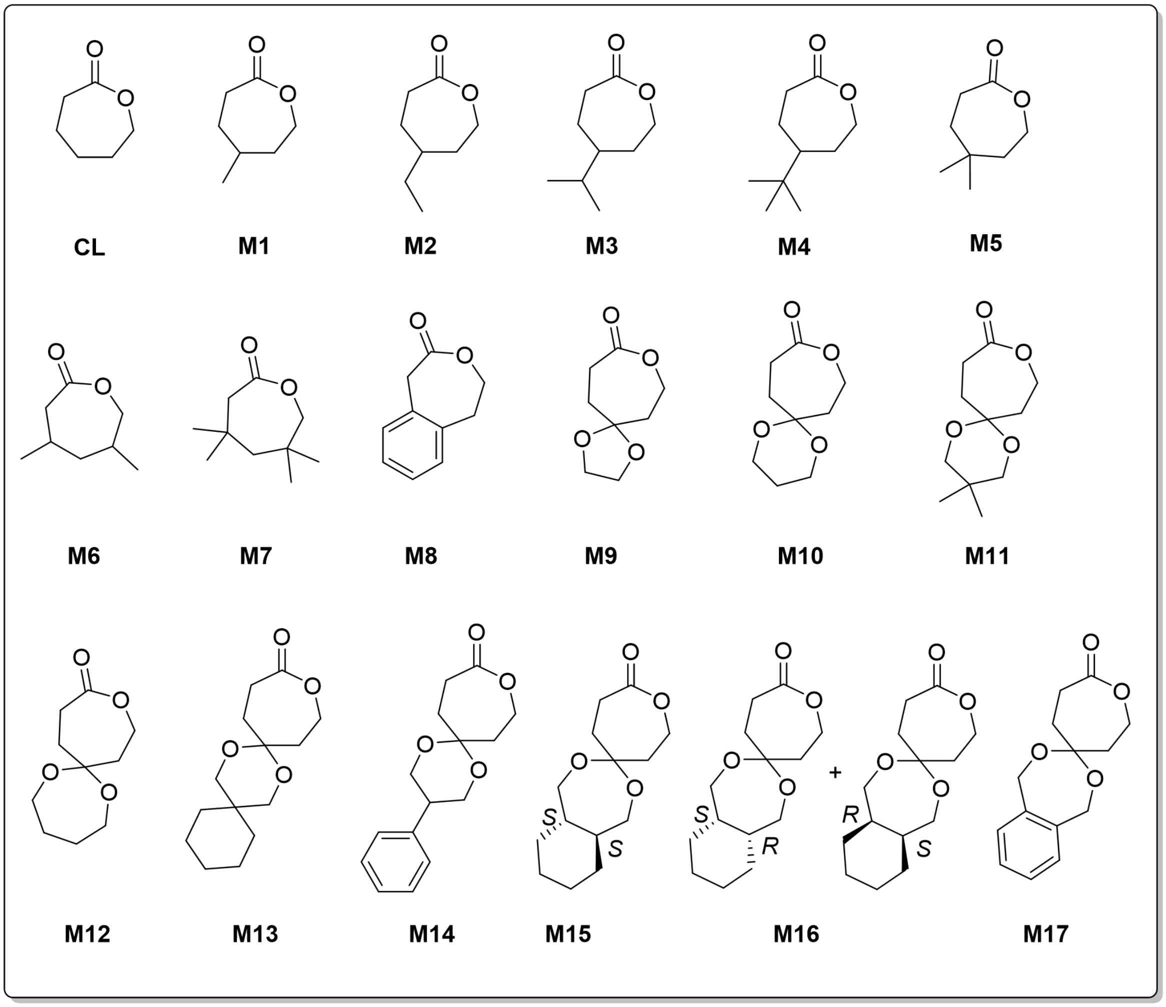

General synthetic procedure for **M1−M7**

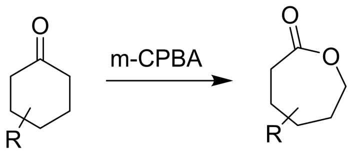

Monomer	Yield/%
**M1**	96
**M2**	71
**M3**	69
**M4**	88
**M5**	78
**M6**	80
**M7**	72

Step: Literature procedures[3-5] were modified for the preparation of **M1**–**M7**. Each substituted cyclohexanone (145 mmol, 1 eq.) was dissolved in dichloromethane (DCM) (100 mL) in a 250 mL round bottom flask (RBF) and was allowed to stir for 10 min in ice bath. Meta-chloroperoxybenzoic acid (m-CPBA, 37 g, 218 mmol, 1.5 eq., 85% purity) was added to the above solution over 30 min. A white precipitate was observed approximately 20 min later after the complete addition of m-CPBA. The reaction was allowed to stir at room temperature overnight. A filtrate was collected by the removal of solid impurities. 200 mL H_2O and sodium sulfite (1.7 eq) were added to the stirring filtrate over 30 min, followed by sodium bicarbonate (20.6 g). The mixture was allowed to stir for another 3 h and then extracted with DCM (3 × 100 mL). The collected organic layers were combined and dried over sodium sulfate, then concentrated by rotary evaporation to yield the crude product. The product was obtained by further purification of the crude product by silica gel column chromatography (ethyl acetate/petroleum ether = 1/5).

Synthetic procedure for the **Diols**

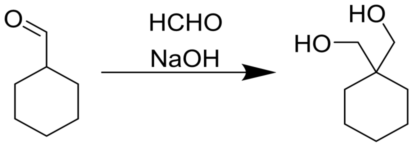

Step: Literature procedures[6] were modified for the preparation of CHD. 39.4 g (0.35 mol) of cyclohexane carboxaldehyde (CHCA) and 96.1 g (1.18 mol) of 37 *wt.*% aqueous formaldehyde solution were charged in a 300 mL three-necked flask and heated to 40 °C. Under stirring, 85.4 g (0.53 mol) of a 25 *wt.*% sodium hydroxide aqueous solution was added over 3.5 h using an additional funnel. After the complete addition, stirring was further continued at 60 °C for 2 h. After completion of the reaction, the reaction mixture was cooled to 15 °C. The resulting solid was filtered to remove the aqueous layer. To 69.6 g of the obtained wet crystals, 139.2 g of water was added and the mixture was stirred and rinsed at 60 °C for 30 min. After washing, the solid was filtered to obtain 68.2 g of wet crystals. To this wet crystal, 90.7 g of acetonitrile was added, heated to 60 °C to dissolve, and then cooled to 5 °C to precipitate a solid for crystallization purification. 37.1 g of purified 1,1-cyclohexanedimethanol (CHD) was obtained (72% yield) by drying the wet crystals.

The 1,1-cyclohexanedimethanol (CHD) was prepared according to the reported procedure, its spectral data is in agreement with the literature.[6] ^{1}H NMR (400 MHz, $CDCl_3$) δ 3.60 (s, 4H), 2.80 (s, 2H), 1.44–1.32 (m, 10H).

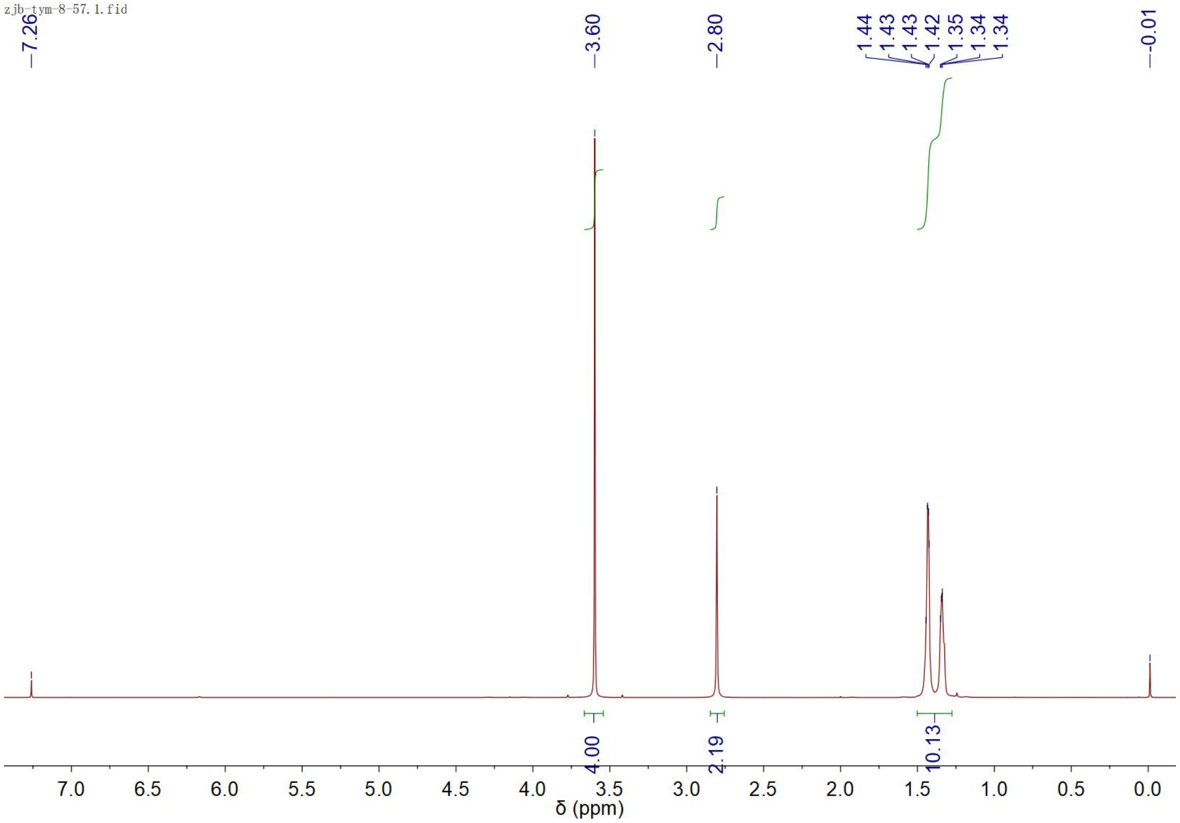

Supplementary Figure 1 ^{1}H NMR ($CDCl_3$, 25 °C) spectrum of 1,1-cyclohexanedimethanol.

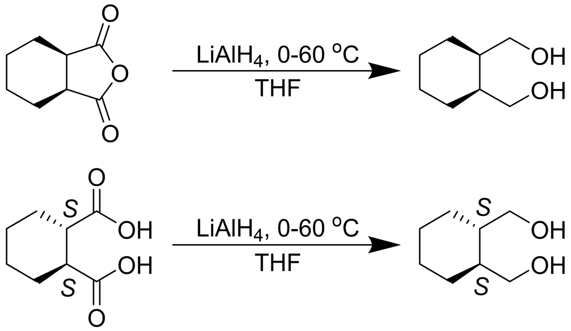

(1*R*,2*S*)-cyclohexane-1,2-diyl-dimethanol and (1*S*,2*S*)-cyclohexane-1,2-diyl-dimethanol were prepared according to the reported literature.[7,8]

(1*R*,2*S*)-cyclohexane-1,2-diyl-dimethanol: ^{1}H-NMR (400 MHz, $CDCl_3$) δ 1.38~1.56 (m, 8H), 1.94 (s, 2H), 3.35 (s, 2H), 3.55~3.60 (m, 2H), 3.73~3.79 (m, 2H). The analytical data is consistent with the reported literature.[8]

(1*S*,2*S*)-cyclohexane-1,2-diyl-dimethanol:^{1}H NMR (400 MHz, $CDCl_3$) δ 0.96~1.16 (m, 2H), 1.19~1.43 (m, 4H), 1.59~1.75 (m, 4H), 3.47~3.61 (m, 4H), 4.19 (s, 1H). The analytical data is consistent with the reported literature.[7]

Synthetic procedure for **M8**

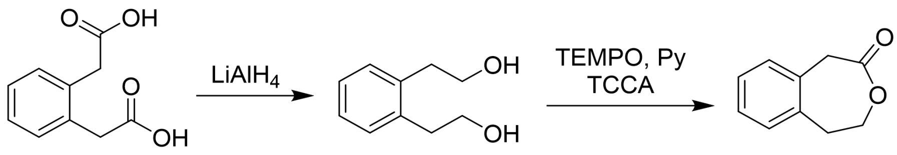

Step One: Literature procedures[9] were modified for the preparation of diethanol. To a solution of 2,2'-(1,2-phenylene) diacetic acid (15.0 g, 77.2 mmol) in THF precooled to 0 °C, was added $LiAlH_4$ powder (11.7 g, 308.8 mmol) slowly. After addition was completed, the mixture was stirred at room temperature overnight. The reaction mixture was then quenched with water (11.7 mL) and 15% NaOH aqueous solution (11.7 mL) at 0 °C. After that, the reaction mixture was treated with THF (100 mL) and anhydrous Na_2SO_4 and filtrated to collect the reaction solution, which was then concentrated to give the crude compound 2,2'-(1,2-phenylene) diethanol and then directly used to the next step without purification. Note: extended wash with THF (2 × 100 mL) could improve the yield.

Step Two: To a mixture of the diol (9.6 g, 58 mmol) and 2,2,6,6-Tetramethyl-1-piperidinyloxy (0.9 g, 5.8 mmol), pyridine (9.2 g, 116 mmol) in CH_3CN (100 mL) was added dropwise with a solution of trichloroisocyanuric acid (TCCA) in CH_3CN (50 mL) over a period of 4 h, and the solution was further stirred for 1 h at room temperature. Saturated $NaHCO_3$ solution (150 mL) was then added to the solution, and ethyl acetate (100 mL) was used to extraction for 3 times. The organic layer was then dried over anhydrous sodium sulfate and concentrated by rotary evaporation to give the crude product. **M8** was collected (7.7 g, 62% total yield for two steps) by further purification via silica gel column chromatography (dichloromethane /ethyl acetate/petroleum ether = 5/1/10).

General synthetic procedure for **M9–M17**

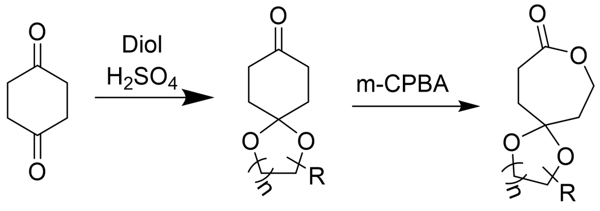

Monomer	Total Yield/%
**M9**	96 (for step two)
**M10**	42
**M11**	40
**M12**	40
**M13**	83
**M14**	58
**M15**	67
**M16**	73
**M17**	55

Step One: 1,4 cyclohexane dione (10–20 g, 90–178 mmol, 1–1.5 eq) and diol (90–178 mmol, 1 equiv.) were dissolved in chloroform (200 ml). Then concentrated sulfuric acid (1.5–3.0 g) was added to the reaction solution at an ambient temperature and stirred for 10 h. The resulting reaction mixture was washed with saturated aqueous sodium bicarbonate solution (100 ml) and the organic layer was separated from the resulting biphasic mixture. The aqueous layer was extracted with DCM (3 × 100 mL). The organic layer was then dried over sodium sulfate and concentrated by rotary evaporation to give the crude product. The product was obtained by petroleum ether (PE) wash (3 × 200 ml) and then directly used to the next step without purification.

Step Two: Each substituted cyclohexanone (145 mmol, 1 equiv.) was dissolved in dichloromethane (DCM) (100 mL) in a 250 mL round bottom flask (RBF) and was allowed to stir for 10 min in ice bath. Meta-chloroperoxybenzoic acid (m-CPBA, 1.5 equiv., 85% purity) was added to the above solution over 30 min. A white precipitate was observed approximately 20 min later after the complete addition of m-CPBA. The reaction was allowed to stir at room temperature overnight. A filtrate was collected by the removal of solid impurities. 200 mL H_2O and sodium sulfite (1.7 equiv.) were added to the stirring filtrate over 30 min, followed by sodium bicarbonate (20.6 g). The mixture was allowed to stir for another 3 h and then extracted with DCM (3 × 100 mL). The collected organic layers were combined and dried over sodium sulfate, then concentrated by rotary evaporation to yield the crude product. The product was obtained by further purification of the crude product by silica gel column chromatography (ethyl acetate/petroleum ether = 1/5).

<u>NMR spectra of produced monomers</u>

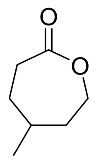

Characterizations of **M1**

Monomer Yield: 16 g; 96%; bp. 105 °C/1 torr; ^{1}H NMR (400 MHz, $CDCl_3$) δ 4.26–4.11 (m, 2H), 2.65–2.54 (m, 2H), 1.93–1.69 (m, 3H), 1.51–1.41 (m, 1H), 1.35–1.25 (m,1H), 0.96 (d, *J* = 6.6 Hz, 3H). Analytical data is consistent with the reported literature.[3]

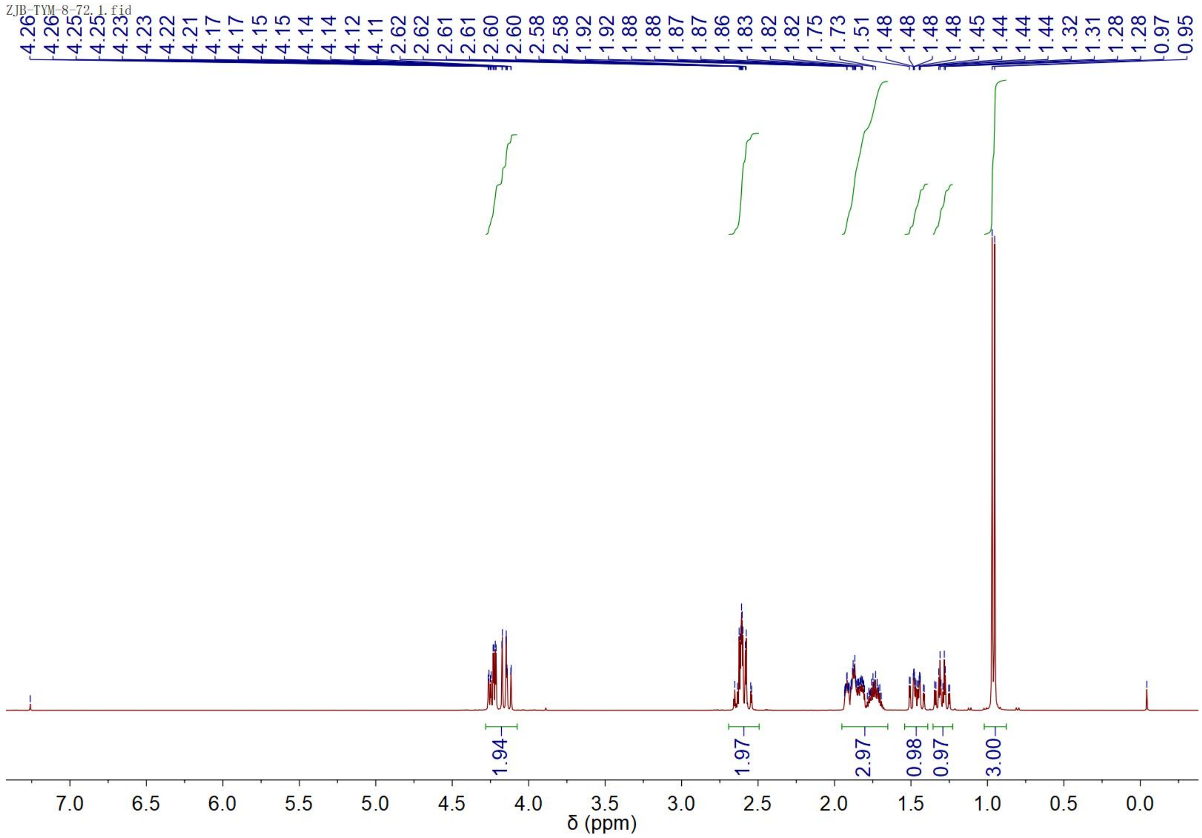

Supplementary Figure 2 ^{1}H NMR ($CDCl_3$, 25 °C) spectrum of **M1**.

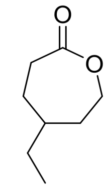

Characterizations of **M2**

Monomer Yield: 14.6 g; 71%; bp. 115 °C/1 torr; ^{1}H NMR (400 MHz, $CDCl_3$) δ 4.31–4.26 (m, 1H), 4.19–4.13 (m, 1H), 2.70–2.55 (m, 2H), 2.00–1.89 (m, 2H), 1.53–1.41 (m, 2H), 1.35–1.25 (m, 3H), 0.92-0.88 (t, J = 7.4 Hz, 3H). Analytical data is consistent with the reported literature.[3]

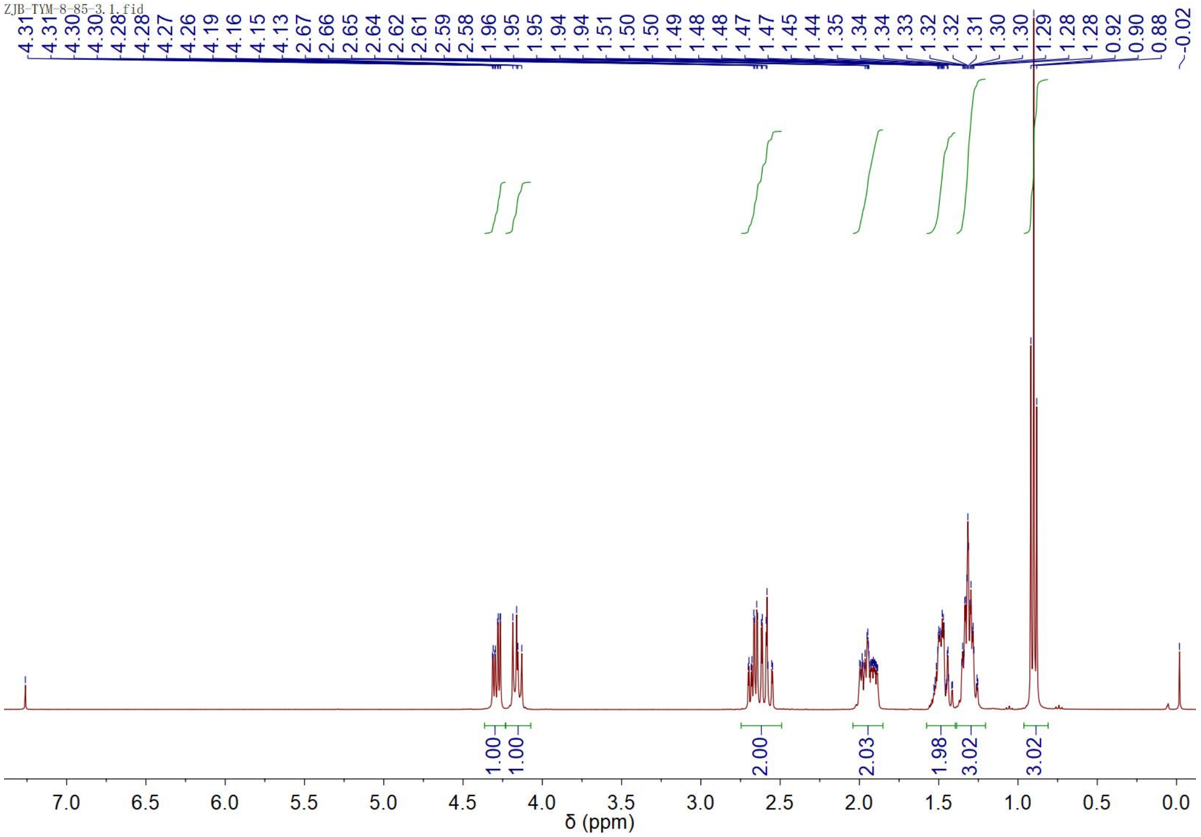

Supplementary Figure 3 ^{1}H NMR ($CDCl_3$, 25 °C) spectrum of **M2**.

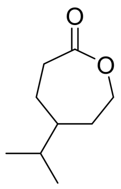

Characterizations of **M3**

Monomer Yield: 15.6 g; 69%; bp. 120 °C/1 torr; ^{1}H NMR (400 MHz, $CDCl_3$) δ 4.34–4.29 (m, 1H), 4.18–4.12 (m, 1H), 2.72–2.53 (m, 2H), 1.93–1.83 (m, 2H), 1.62–1.37 (m, 4H), 0.88 (d, *J* = 6.3 Hz, 6H). Analytical data is consistent with the reported literature.[3]

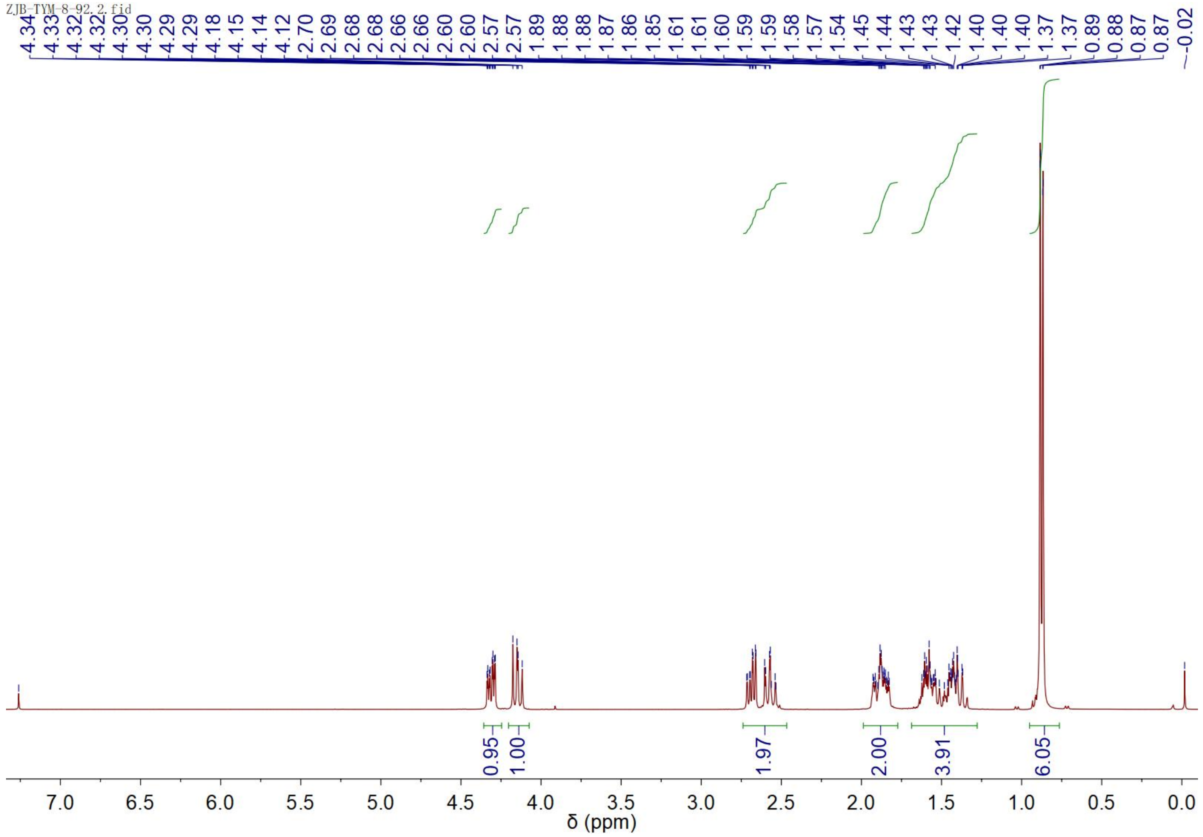

Supplementary Figure 4 ^{1}H NMR ($CDCl_3$, 25 °C) spectrum of **M3**.

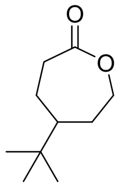

Characterizations of **M4**

Monomer Yield: 22 g; 88%; ^{1}H NMR (400 MHz, $CDCl_3$) δ 4.36–4.31 (m, 1H), 4.17–4.11 (m, 1H), 2.73–2.68 (m, 1H), 2.59–2.56 (m, 1H), 2.08–2.02 (m, 2H), 1.60–1.53 (m, 1H), 1.37–1.30 (m, 2H), 0.89 (s, 9H). Analytical data is consistent with the reported literature.[3]

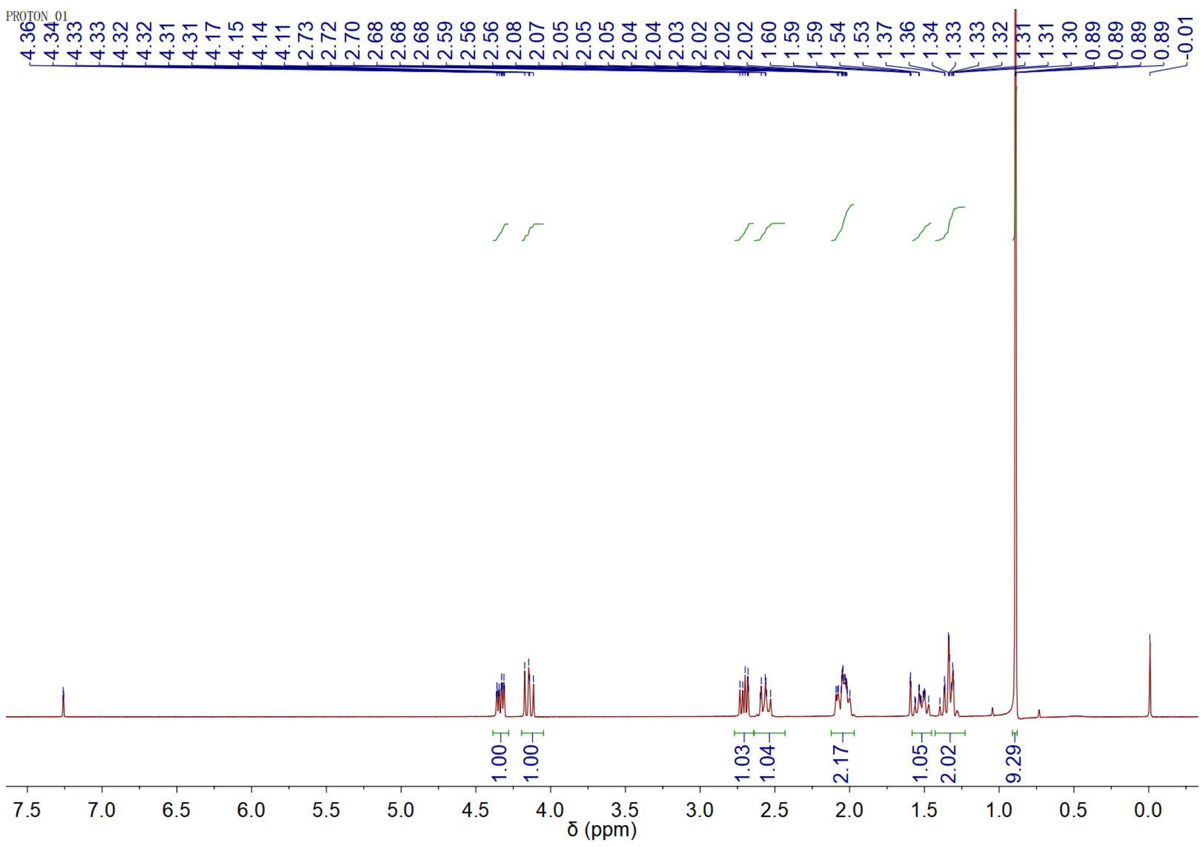

Supplementary Figure 5 ^{1}H NMR ($CDCl_3$, 25 °C) spectrum of **M4**.

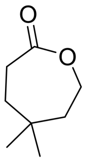

Characterizations of **M5**

Monomer Yield: 16g; 78%; ^{1}H NMR (400 MHz, $CDCl_3$) δ 4.21–4.19 (m, 2H), 2.62–2.59 (m, 2H), 1.66–1.64 (m, 2H), 1.58–1.55 (m, 2H), 1.02 (s, 6H). Analytical data is consistent with the reported literature.[4]

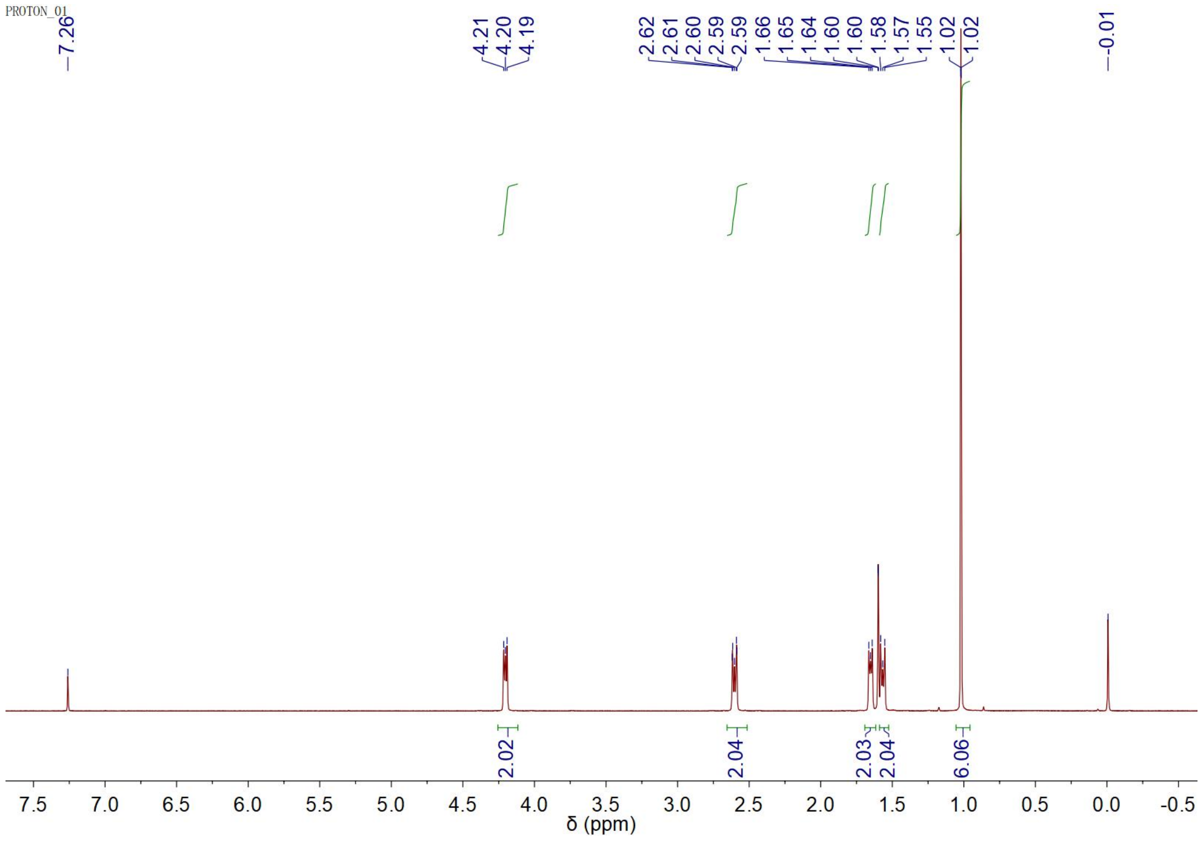

Supplementary Figure 6 ^{1}H NMR ($CDCl_3$, 25 °C) spectrum of **M5**.

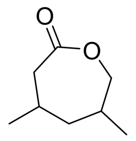

Characterizations of **M6**

Monomer Yield: 16 g; 80% (mixture of diastereomers); bp. 95 °C/0.6 torr; ^{1}H NMR (400 MHz, $CDCl_3$) δ 4.16–3.92 (m, 2H), 2.68–2.48 (m, 2H), 2.16–1.87 (m, 3H), 1.05–0.91 (m, 7H). Analytical data is consistent with the reported literature.[5]

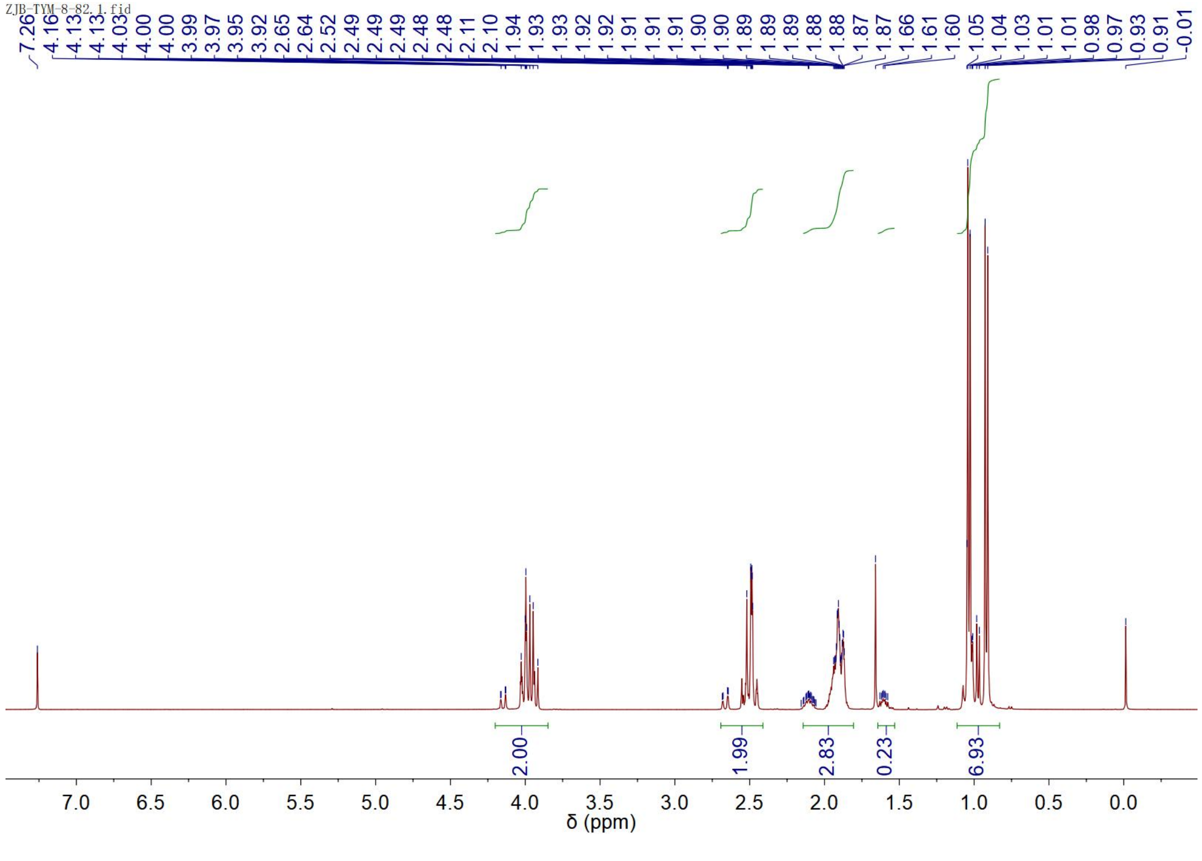

Supplementary Figure 7 ^{1}H NMR ($CDCl_3$, 25 °C) spectrum of **M6**.

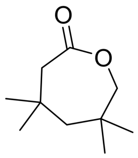

Characterizations of **M7**

Monomer Yield: 17.8 g; 72%; ^{1}H NMR (400 MHz, $CDCl_3$) δ 3.93 (s, 2H), 2.56 (s, 2H), 1.47 (s, 2H), 1.08 (s, 6H), 1.02 (s, 6H). ^{13}C NMR (100 MHz, $CDCl_3$) δ 174.0, 76.5, 55.5, 46.0, 35.0, 32.2, 30.8, 27.1. ESI-MS: calculated for $C_{10}H_{19}O_2$ $[M + H]^+$ 171.1380, found 171.1381.

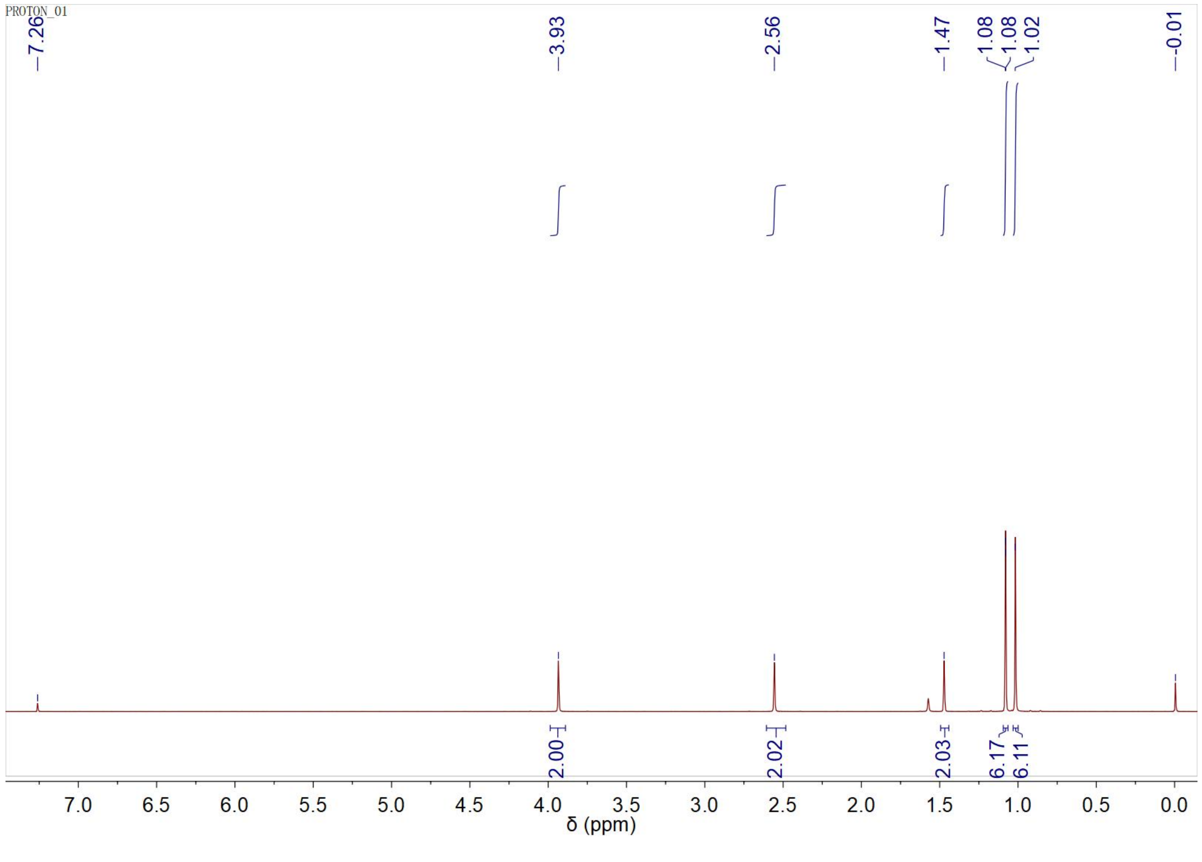

Supplementary Figure 8 ^{1}H NMR ($CDCl_3$, 25 °C) spectrum of **M7**.

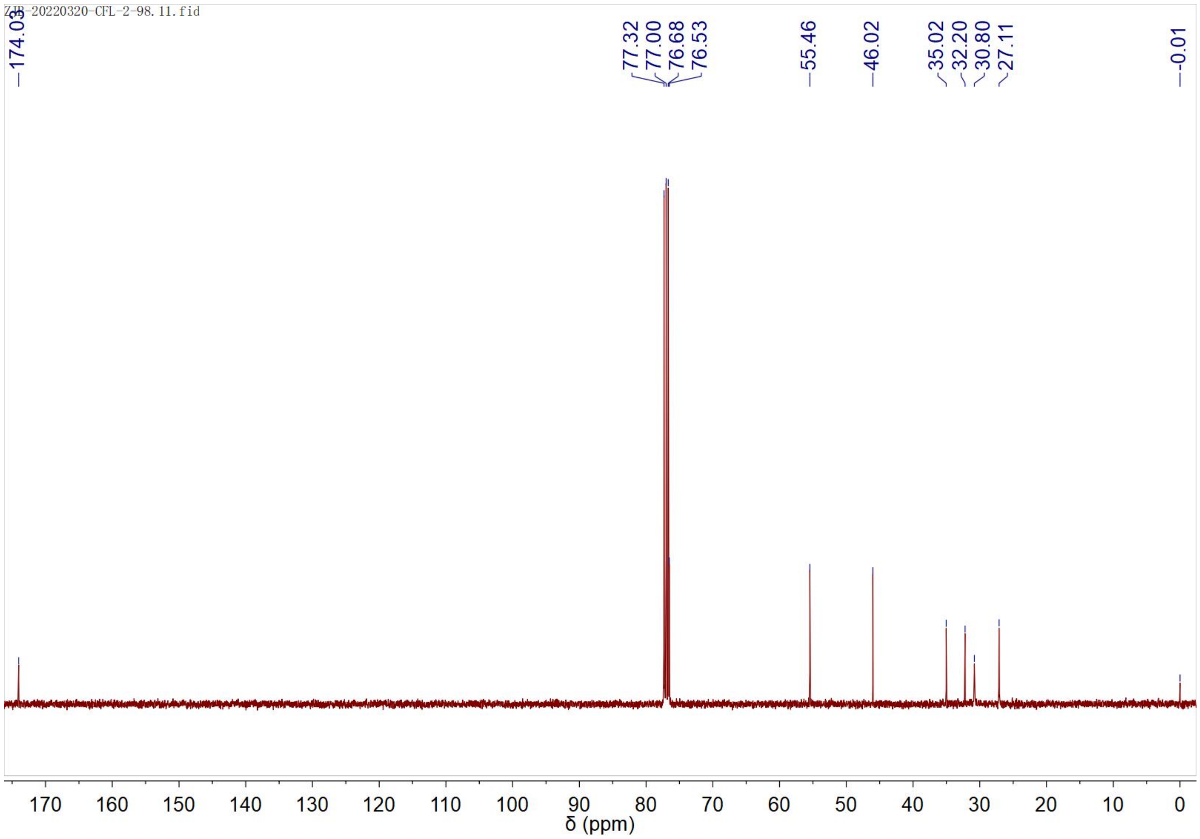

Supplementary Figure 9 ^{13}C NMR ($CDCl_3$, 25 °C) spectrum of **M7**.

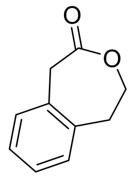

Characterizations of **M8**

Monomer Yield: 7.7g; 82%; ^{1}H NMR (400 MHz, $CDCl_3$) δ 7.23–7.10 (m, 4H), 4.63 (t, J = 5.6 Hz, 2H), 4.06 (s, 2H), 3.32 (t, J = 5.7 Hz, 2H). ^{13}C NMR (100 MHz, $CDCl_3$) δ 172.3, 134.8, 130.9, 130.4, 128.6, 127.7, 127.0, 65.7, 40.4, 33.9. Analytical data is consistent with the reported literature.[10]

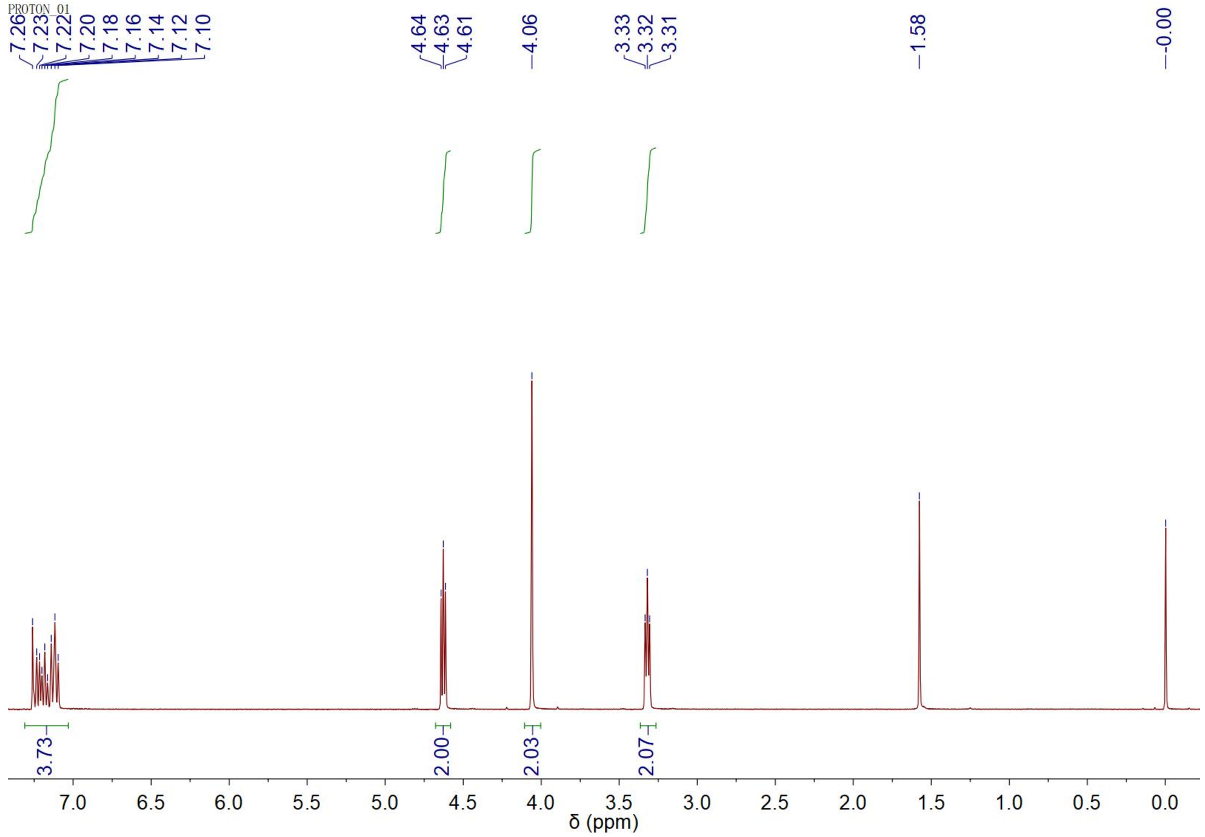

Supplementary Figure 10 ^{1}H NMR ($CDCl_3$, 25 °C) spectrum of **M8**.

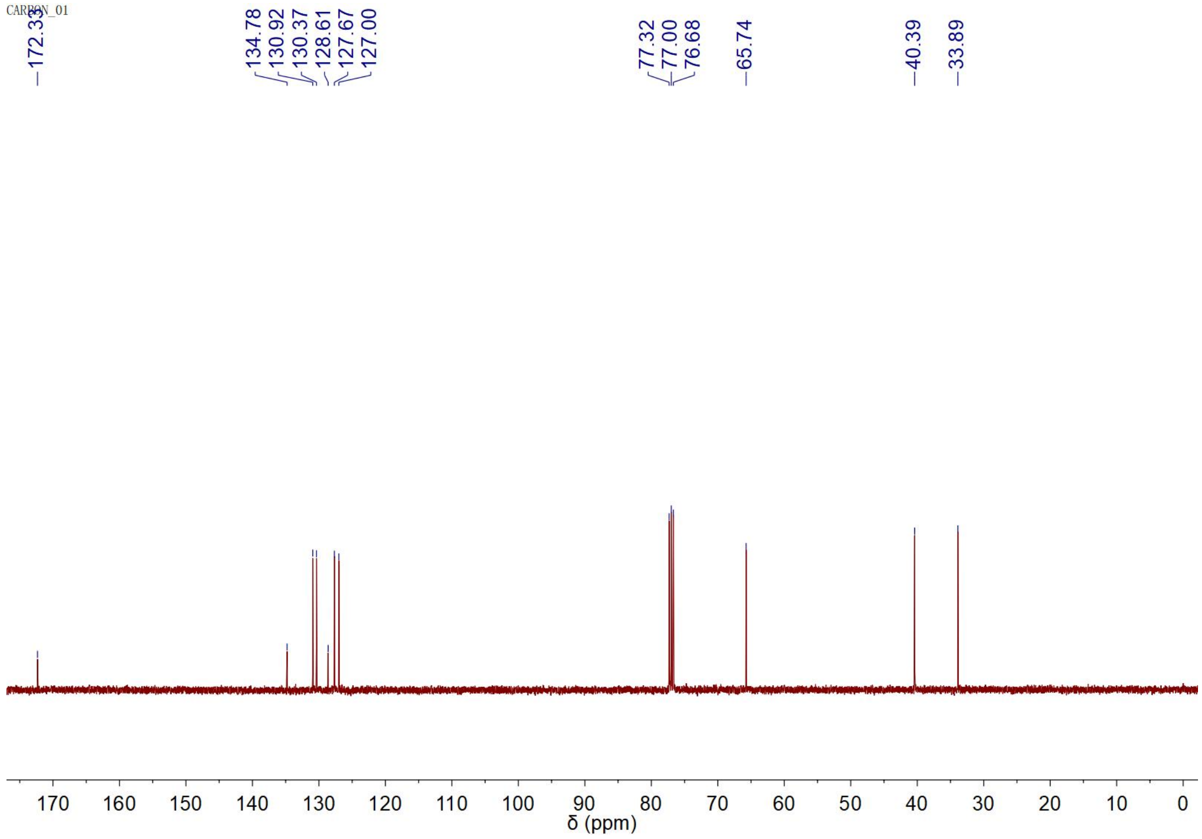

Supplementary Figure 11 ^{13}C NMR ($CDCl_3$, 25 °C) spectrum of **M8**.

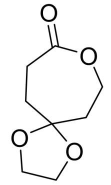

Characterizations of **M9**

Monomer Yield: 24 g; 96%; ^{1}H NMR (400 MHz, $CDCl_3$) δ 4.29–4.27 (m, 2H), 3.98 (s, 4H), 2.71–2.68 (m, 2H), 2.01–1.99 (m, 2H), 1.91–1.88 (m, 2H). Analytical data is consistent with the reported literature.[11]

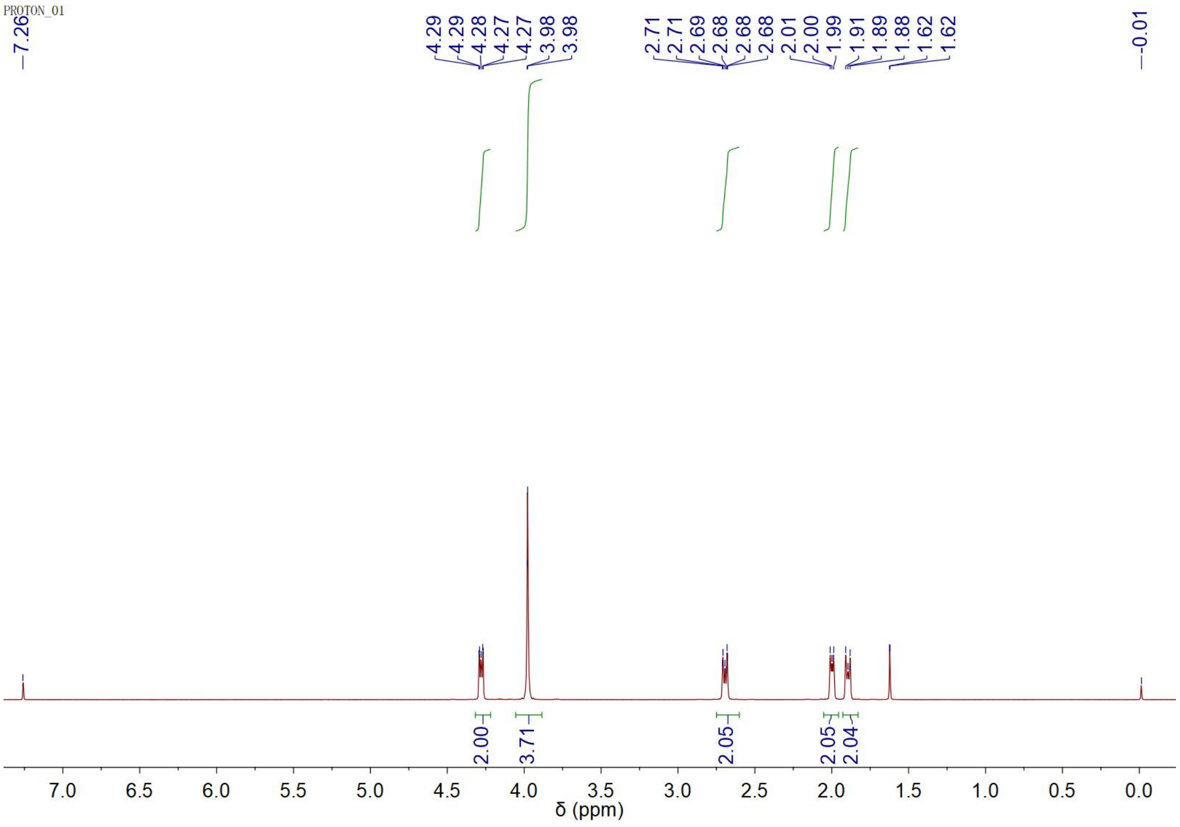

Supplementary Figure 12 ^{1}H NMR ($CDCl_3$, 25 °C) spectrum of **M9**.

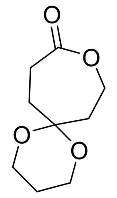

Characterizations of **M10**

Monomer Yield: 14 g; 42%; ^{1}H NMR (400 MHz, $CDCl_3$) δ 4.24–4.21 (m, 2H), 3.90–3.86 (m, 4H), 2.63–2.60 (m, 2H), 2.14–2.06 (m, 4H), 1.79–1.66 (m, 2H).^{13}C NMR (100 MHz, $CDCl_3$) δ 175.5, 96.8, 63.3, 59.4, 37.1, 29.3, 27.7, 25.2. ESI-MS: calculated for $C_9H_{15}O_4$ $[M + H]^+$ 187.0965, found 187.0961.

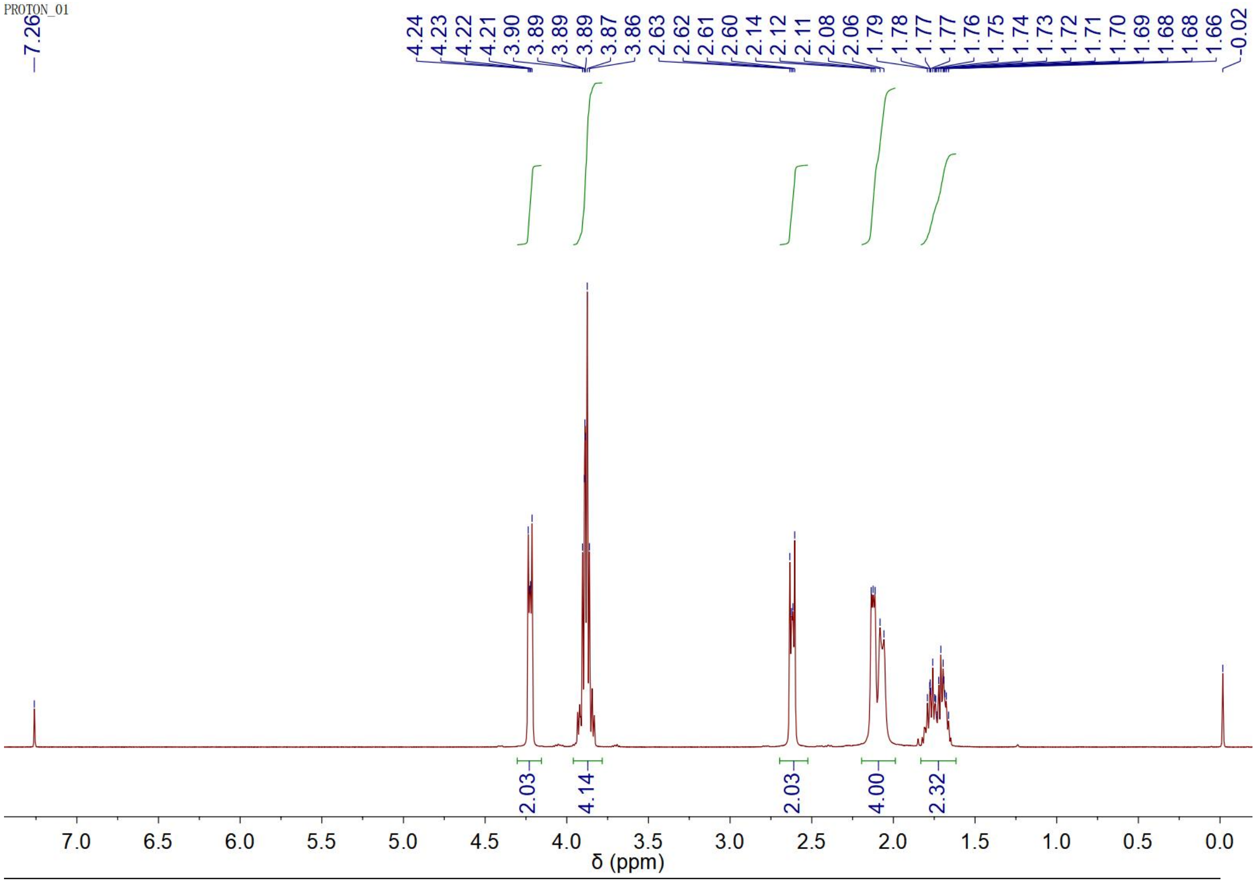

Supplementary Figure 13 ^{1}H NMR ($CDCl_3$, 25 °C) spectrum of **M10**.

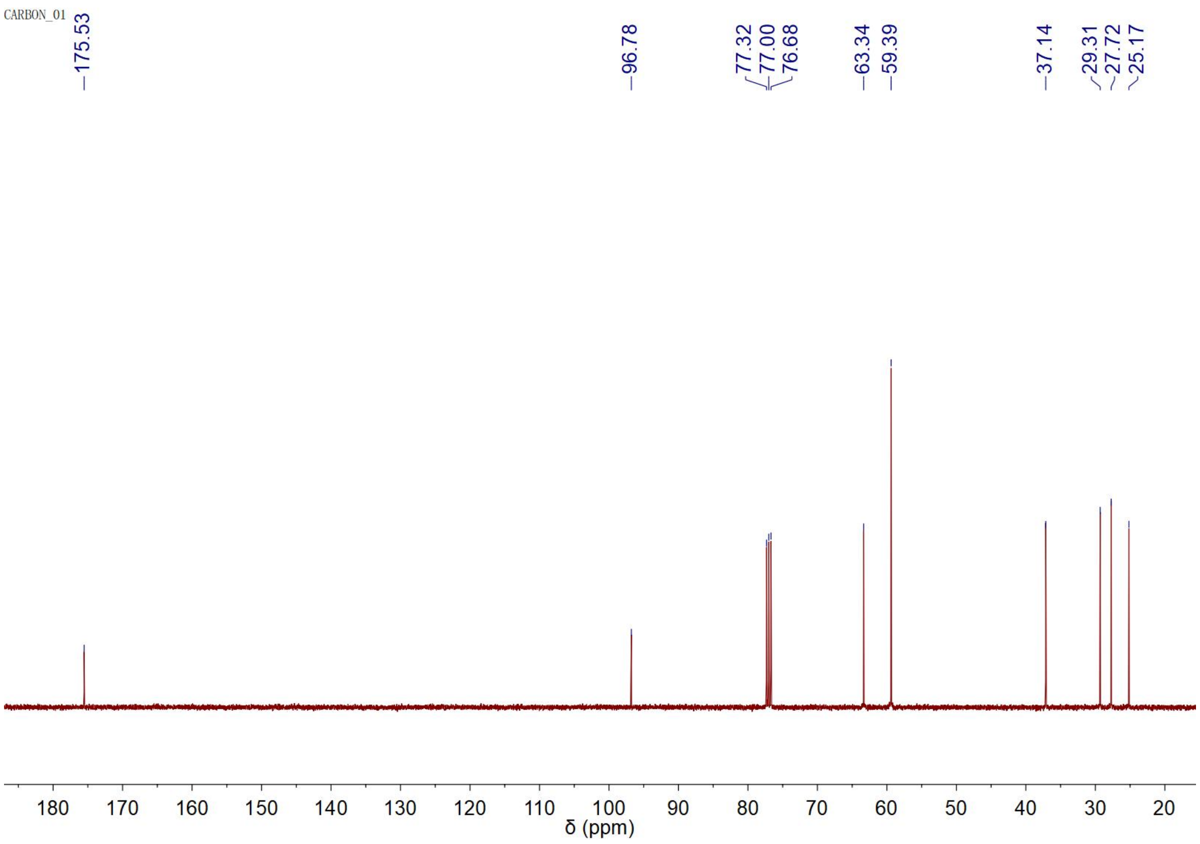

Supplementary Figure 14 ^{13}C NMR ($CDCl_3$, 25 °C) spectrum of **M10**.

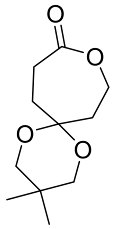

Characterizations of **M11**

Monomer Yield: 15 g; 40%; ^{1}H NMR (400 MHz, $CDCl_3$) δ 4.24–4.21 (m, 2H), 3.51–3.43 (m, 4H), 2.63–2.60 (m, 2H), 2.13–2.03 (m, 4H), 0.98 (s, 3H), 0.93 (s, 3H). ^{13}C NMR (100 MHz, $CDCl_3$) δ 175.6, 96.6, 70.0, 63.5, 36.6, 30.1, 28.9, 27.8, 22.6, 22.5. ESI-MS: calculated for $C_{11}H_{19}O_4$ $[M + H]^+$ 215.1278, found 215.1273.

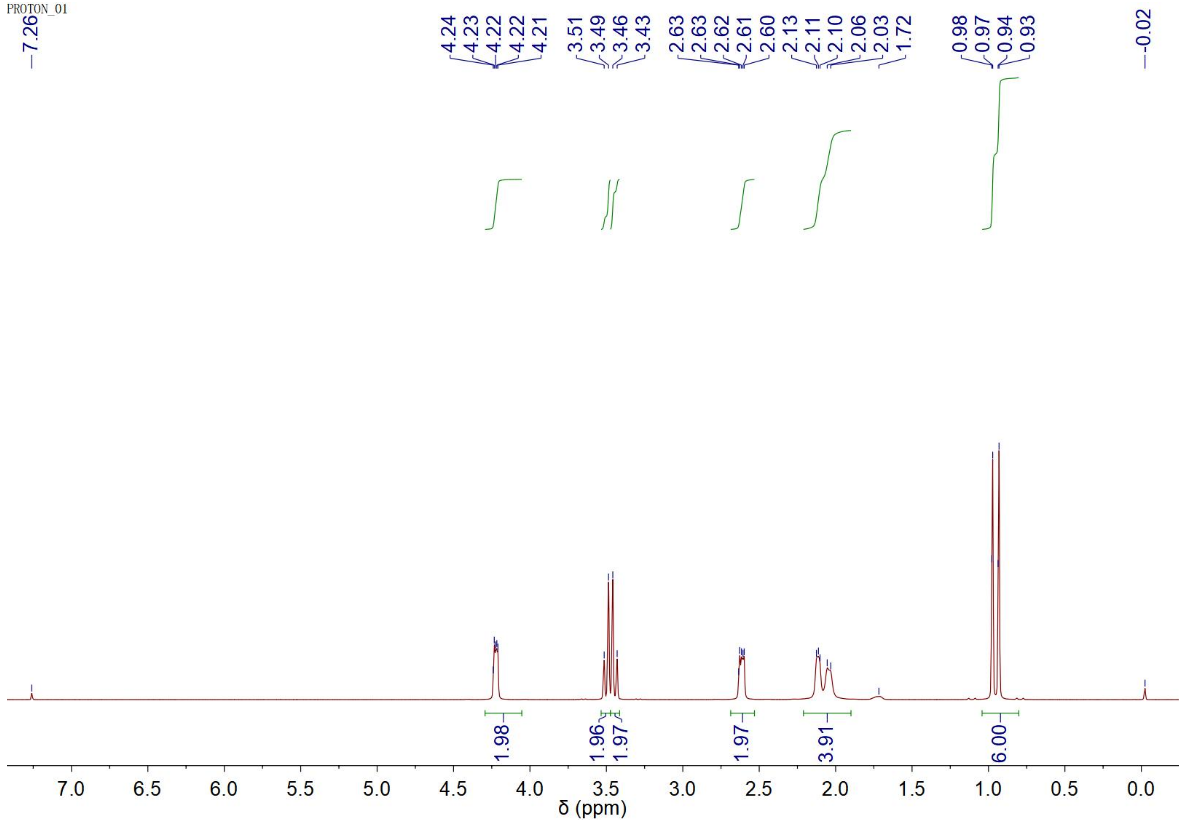

Supplementary Figure 15 ^{1}H NMR ($CDCl_3$, 25 °C) spectrum of **M11**.

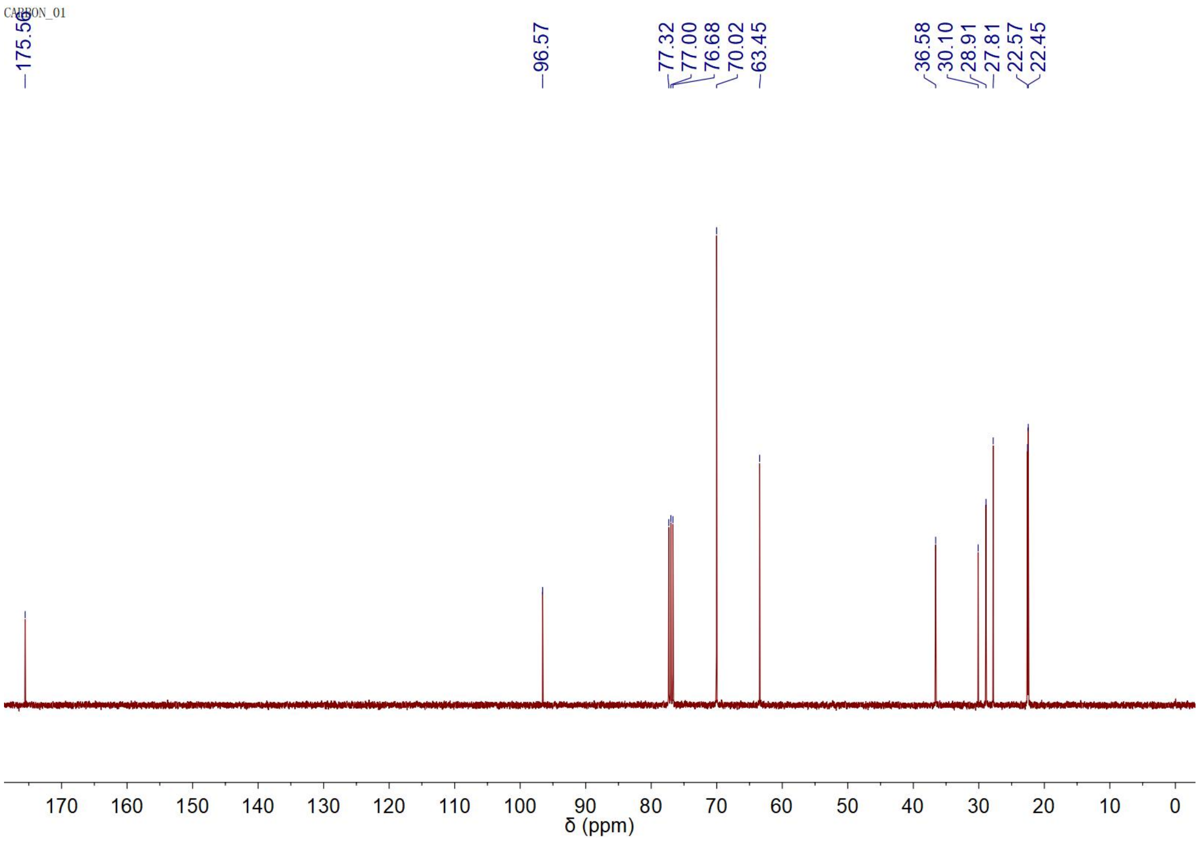

Supplementary Figure 16 ^{13}C NMR ($CDCl_3$, 25 °C) spectrum of **M11**.

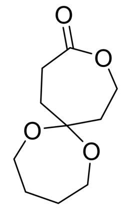

Characterizations of **M12**

Monomer Yield: 14 g; 40%; ^{1}H NMR (400 MHz, $CDCl_3$) δ 4.24–4.22 (m, 2H), 3.69–3.67 (m, 4H), 2.65–2.62 (m, 2H), 2.01–1.98 (m, 2H), 1.92–1.89 (m, 2H), 1.65–1.60 (m, 4H). ^{13}C NMR (100 MHz, $CDCl_3$) δ 175.8, 100.4, 64.2, 62.2, 37.4, 30.9, 29.6, 28.5. ESI-MS: calculated for $C_{10}H_{17}O_4$ $[M + H]^+$ 201.1121, found 201.1118.

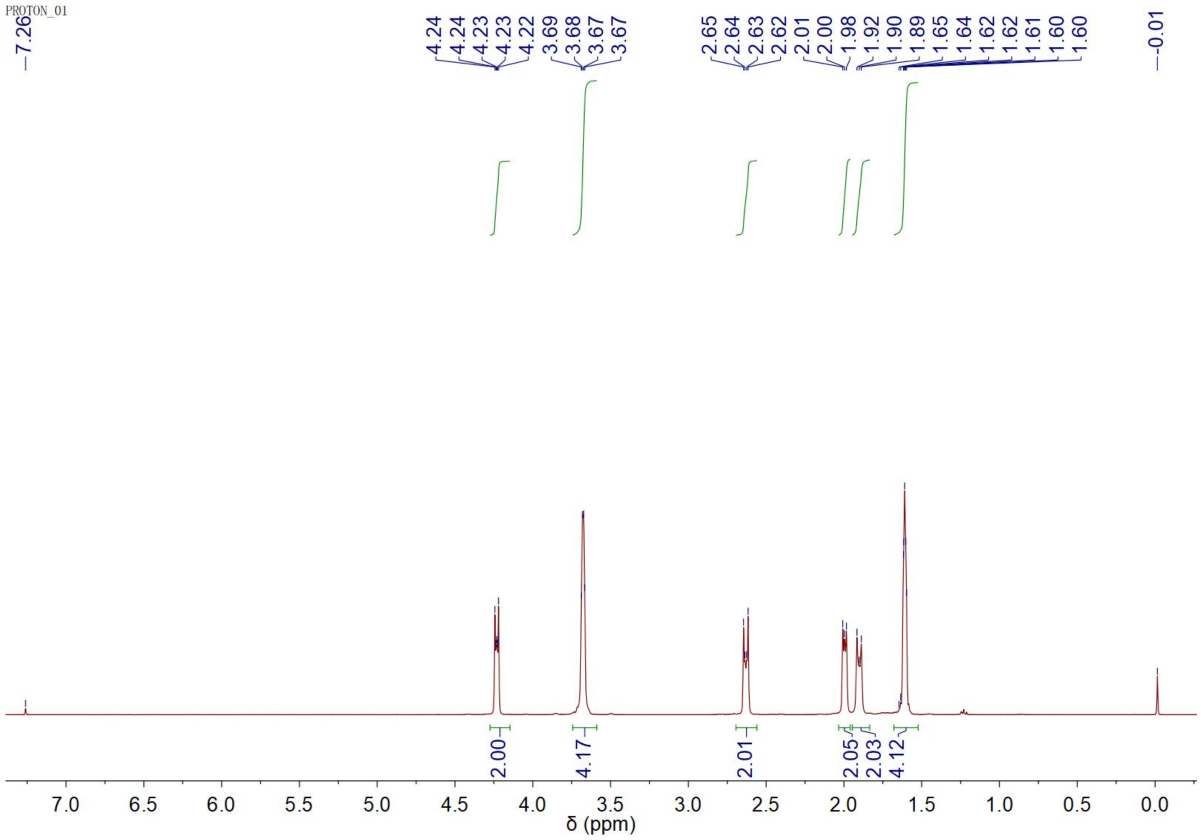

Supplementary Figure 17 ^{1}H NMR ($CDCl_3$, 25 °C) spectrum of **M12**.

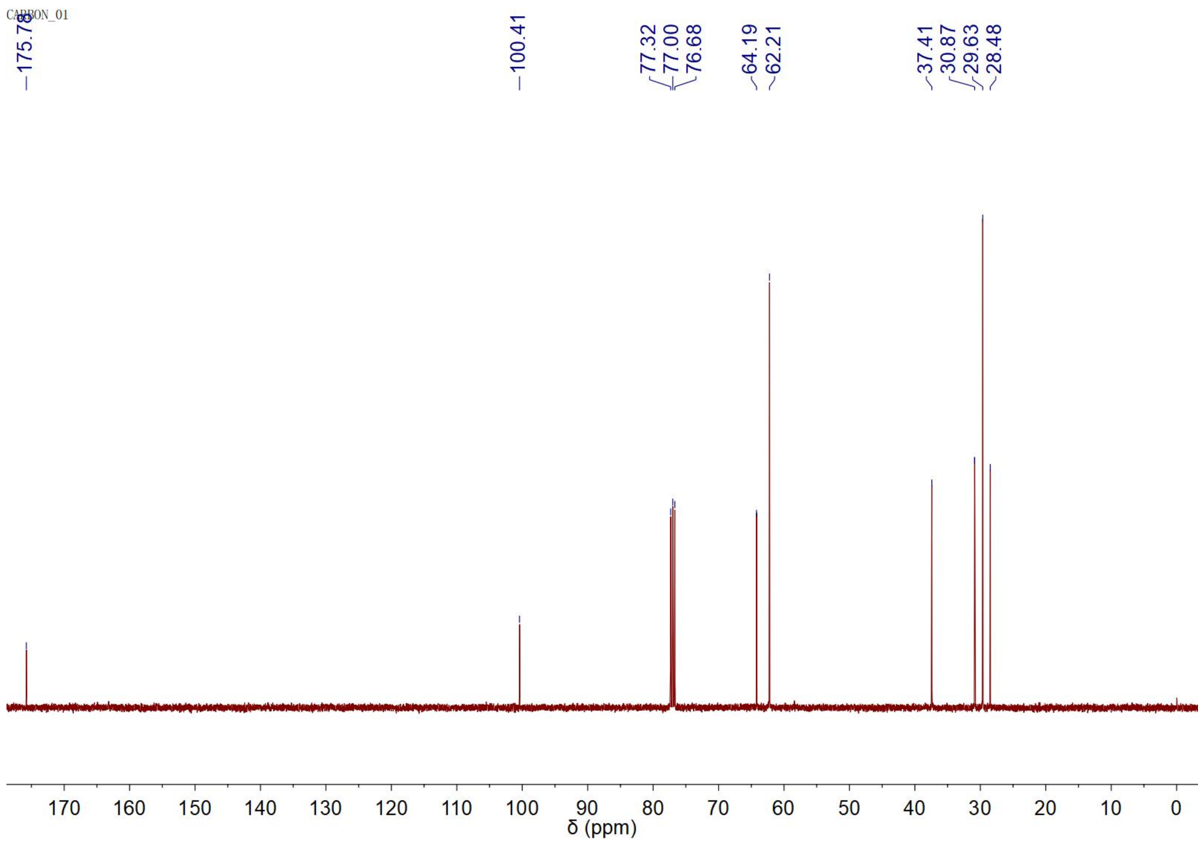

Supplementary Figure 18 ^{13}C NMR ($CDCl_3$, 25 °C) spectrum of **M12**.

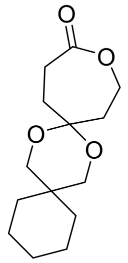

Characterizations of **M13**

Monomer Yield: 30.6 g; 83%; ^{1}H NMR (400 MHz, $CDCl_3$) 4.24–4.22 (m, 2H), 3.58 (s, 4H), 2.64–2.61 (m, 2H), 2.13–2.04 (m, 4H), 1.43–1.38 (m, 10H). ^{13}C NMR (100 MHz, $CDCl_3$) δ 175.6, 97.6, 68.3, 63.5, 36.6, 32.6, 31.6, 31.4, 29.0, 27.9, 26.4, 21.3. ESI-MS: calculated for $C_{14}H_{23}O_4$ $[M + H]^+$ 255.1591, found 255.1588.

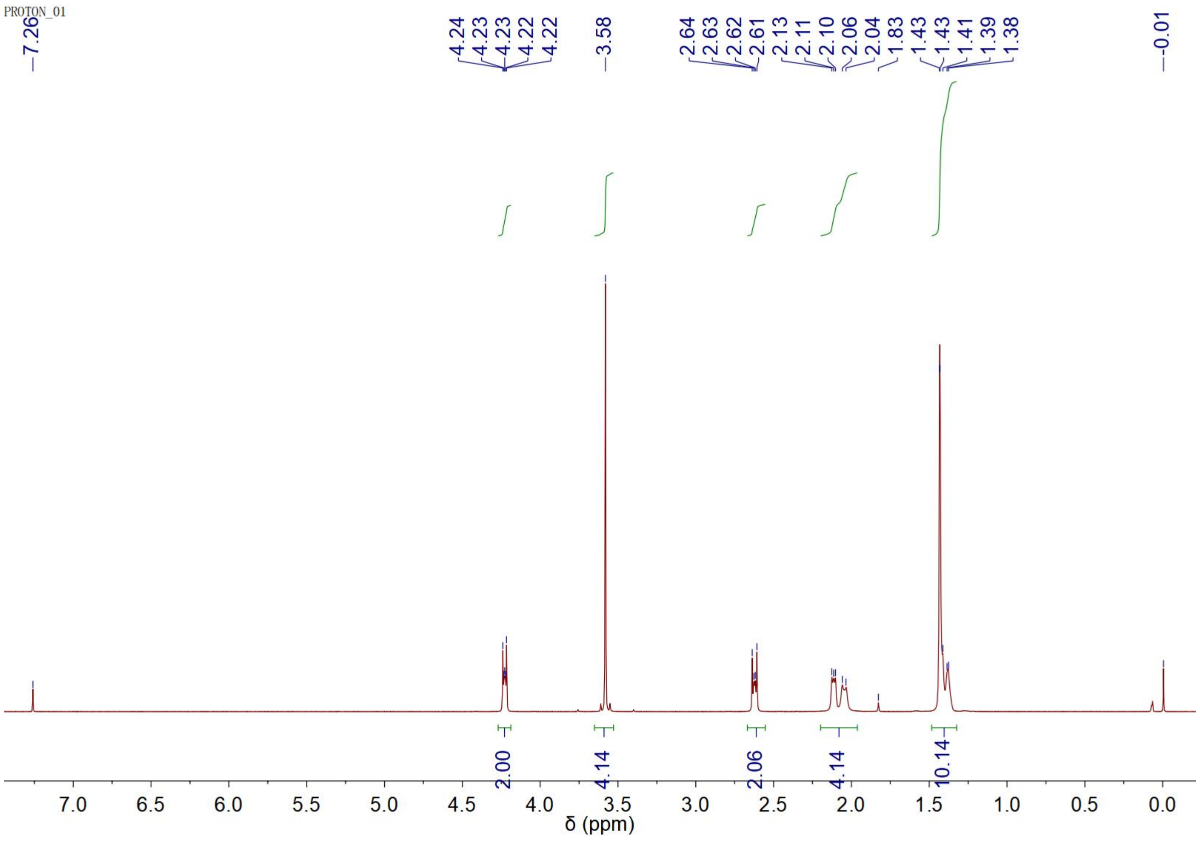

Supplementary Figure 19 ^{1}H NMR ($CDCl_3$, 25 °C) spectrum of **M13**.

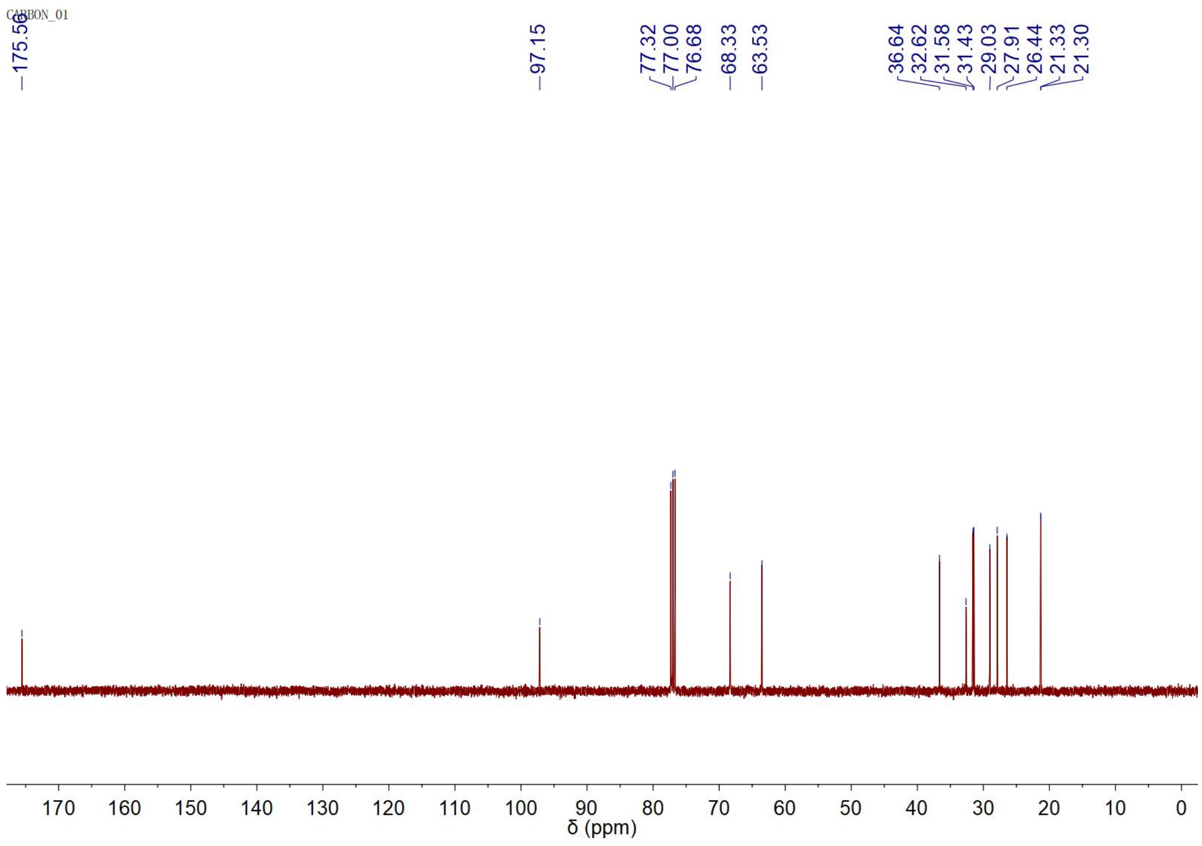

Supplementary Figure 20 ^{13}C NMR ($CDCl_3$, 25 °C) spectrum of **M13**.

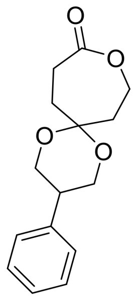

Characterizations of **M14**

Monomer Yield: 10 g; 58% (mixture of diastereomers); ^{1}H NMR (400 MHz, $CDCl_3$) δ 7.34–7.19 (m, 5H), 4.29–4.24 (m, 2H), 4.04–3.92 (m, 4H), 3.17–3.11 (m, 1H), 2.71–2.63 (m, 2H), 2.35–2.26 (m, 2H), 2.11–1.99 (m, 2H). ^{13}C NMR (100 MHz, $CDCl_3$) δ 175.4, 175.3, 138.5, 138.2, 128.8, 128.8, 127.6, 127.5, 127.4, 96.8, 64.5, 64.5, 63.4, 40.8, 40.8, 40.7, 33.6, 32.4, 27.9, 27.8, 25.5. ESI-MS: calculated for $C_{15}H_{19}O_4$ $[M + H]^+$ 263.1278, found 263.1281.

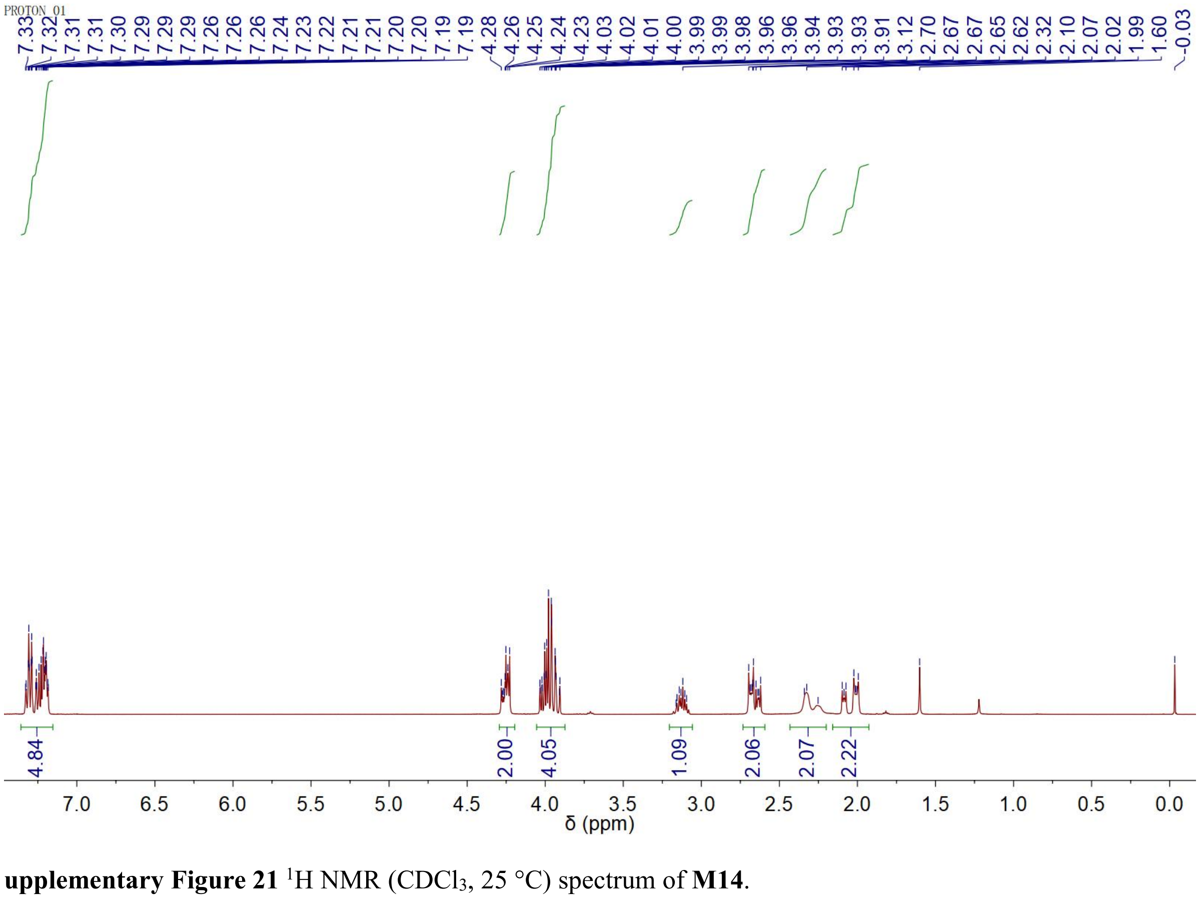

S

upplementary Figure 21 ^{1}H NMR ($CDCl_3$, 25 °C) spectrum of **M14**.

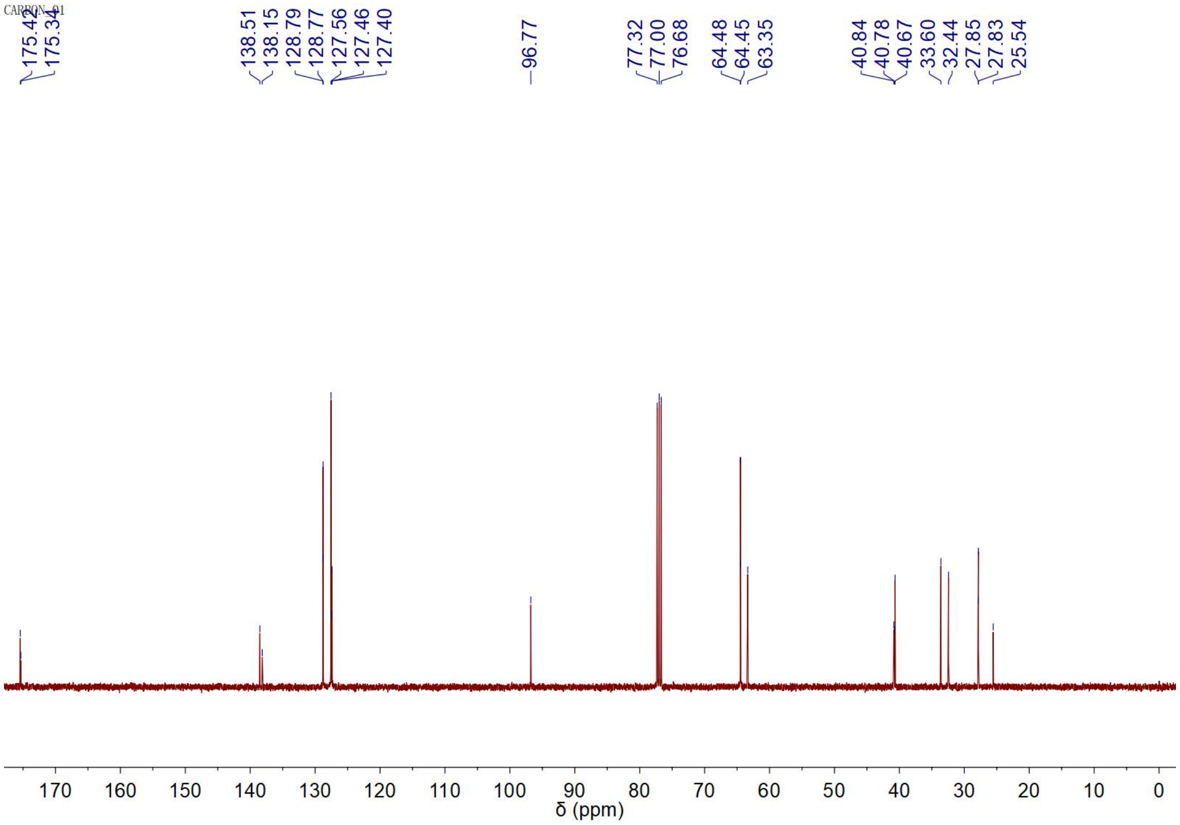

Supplementary Figure 22 ^{13}C NMR ($CDCl_3$, 25 °C) spectrum of **M14**.

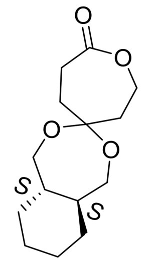

Characterizations of **M15**

Monomer Yield: 10 g; 67% (1*S*,2*S*)-cyclohexane-1,2-diyl-dimethanol was used as starting material; ^{1}H NMR (400 MHz, $CDCl_3$) δ 4.23–4.21 (m, 2H), 3.55–3.39 (m, 4H), 2.67–2.57 (m, 2H), 2.08–1.73 (m, 6H), 1.57–1.53 (m, 2H), 1.30–1.14 (m, 4H), 0.89–0.79 (m, 2H). ^{13}C NMR (100 MHz, $CDCl_3$) δ 175.7, 100.1, 67.3, 67.1, 64.2, 46.1, 46.1, 37.5, 30.9, 28.5, 28.3, 28.3, 26.0. ESI-MS: calculated for $C_{14}H_{23}O_4$ $[M + H]^+$ 255.1591, found 255.1592.

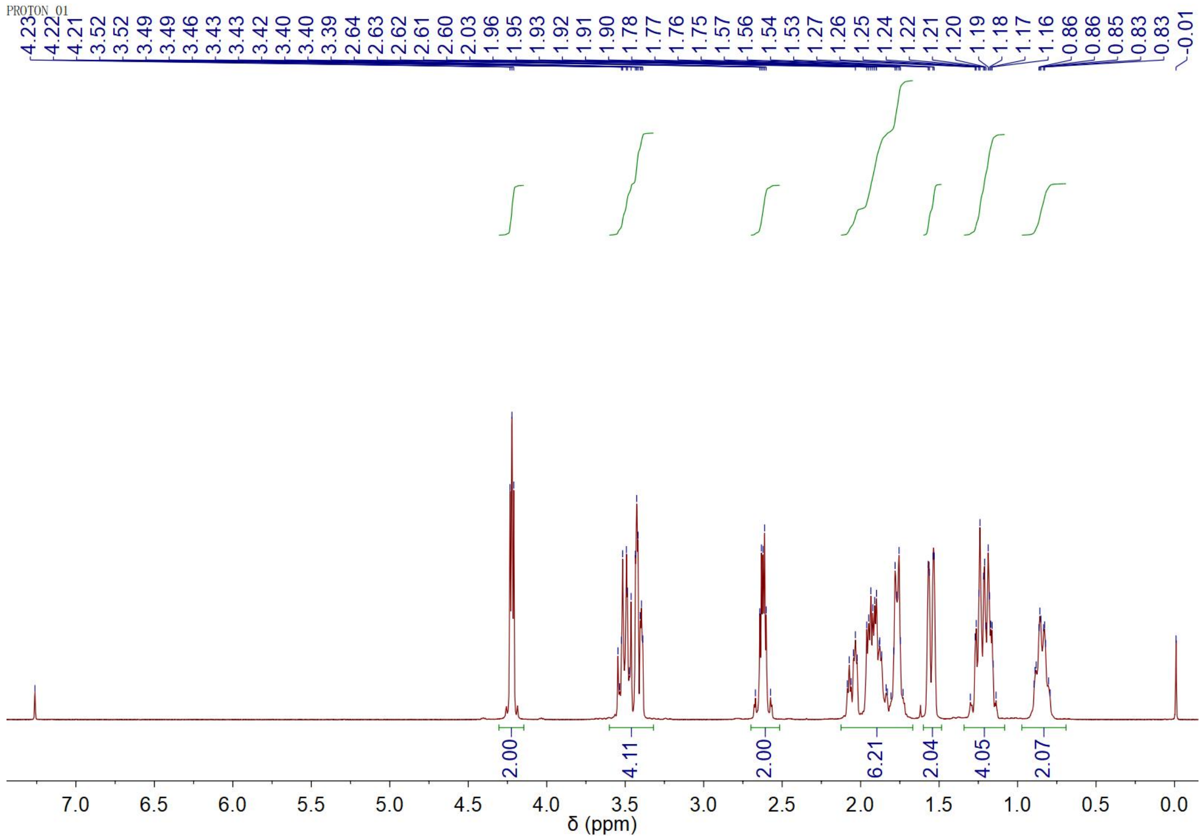

Supplementary Figure 23 ^{1}H NMR ($CDCl_3$, 25 °C) spectrum of **M15**.

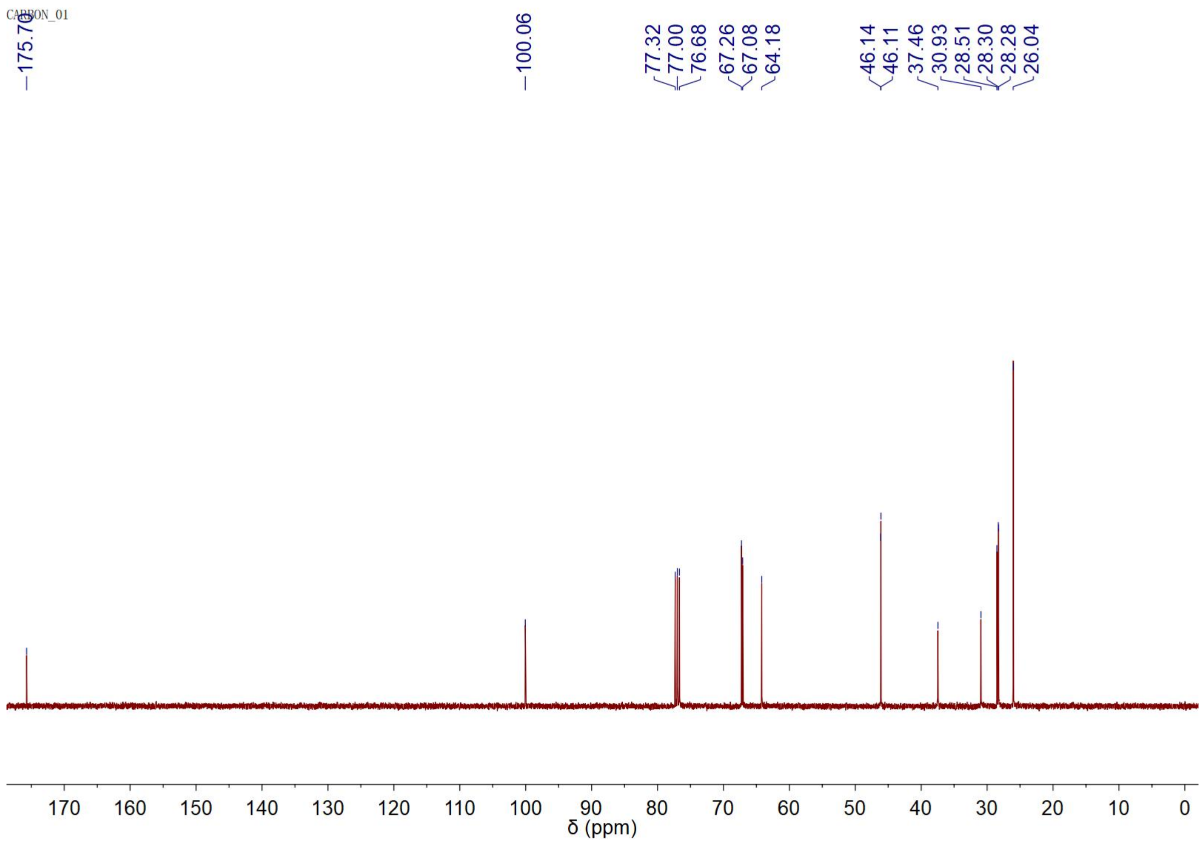

Supplementary Figure 24 ^{13}C NMR ($CDCl_3$, 25 °C) spectrum of **M15**.

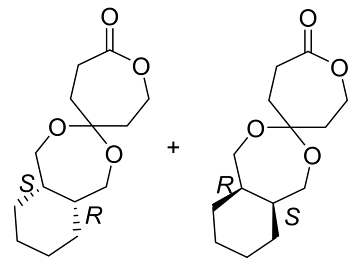

<u>Characterizations of **M16**</u>

Monomer Yield (a mixture of (*R*, *S*)-**M16** and (*S*, *R*)-**M16**): 9.5 g; 73%; ^{1}H NMR (400 MHz, $CDCl_3$) δ 4.24–4.21 (m, 2H), 3.68–3.58 (m, 4H), 2.64–2.61 (m, 2H), 2.01–1.89 (m, 4H), 1.75–1.62 (m, 4H), 1.52–1.36 (m, 6H). ^{13}C NMR (100 MHz, $CDCl_3$) δ 175.8, 100.5, 64.2, 64.2, 37.3, 37.3, 30.8, 30.8, 28.5, 28.5. ESI-MS: calculated for $C_{14}H_{23}O_4$ $[M + H]^+$ 255.1591, found 255.1588.

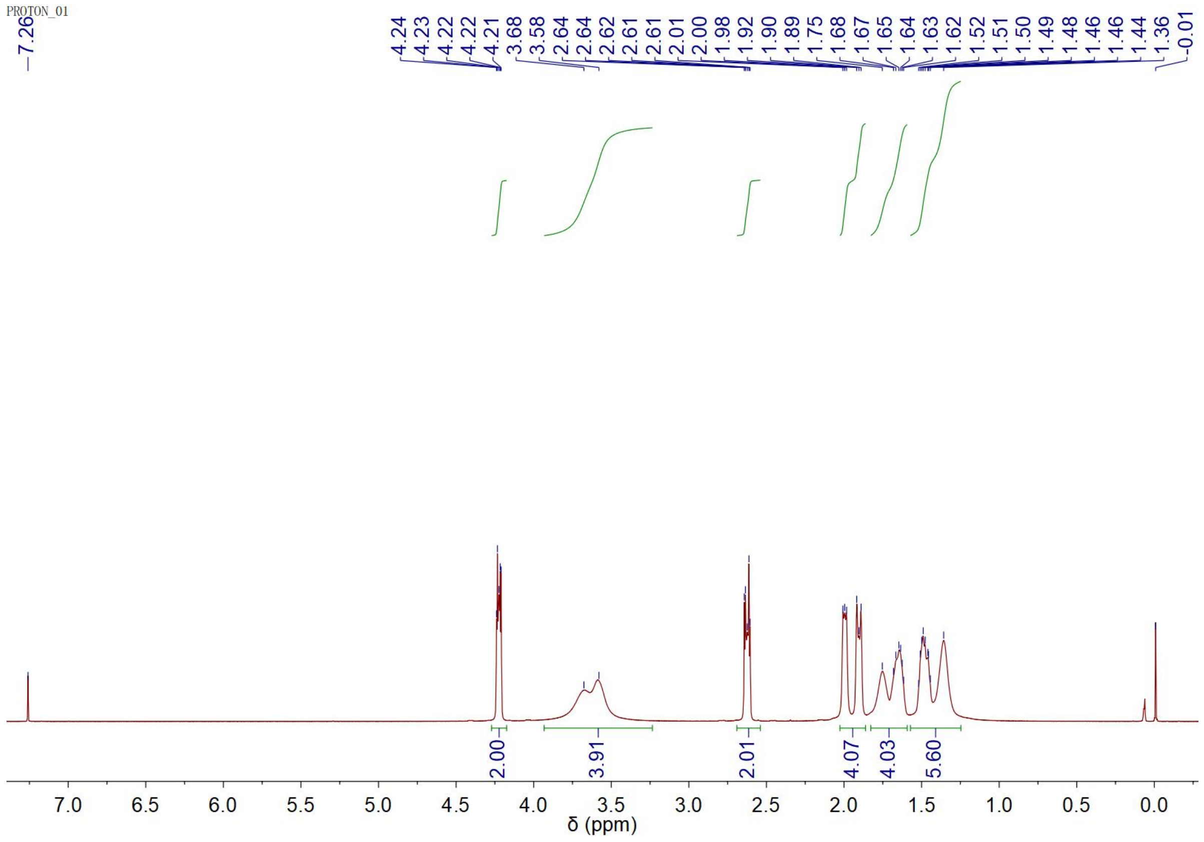

Supplementary Figure 25 ^{1}H NMR ($CDCl_3$, 25 °C) spectrum of **M16**.

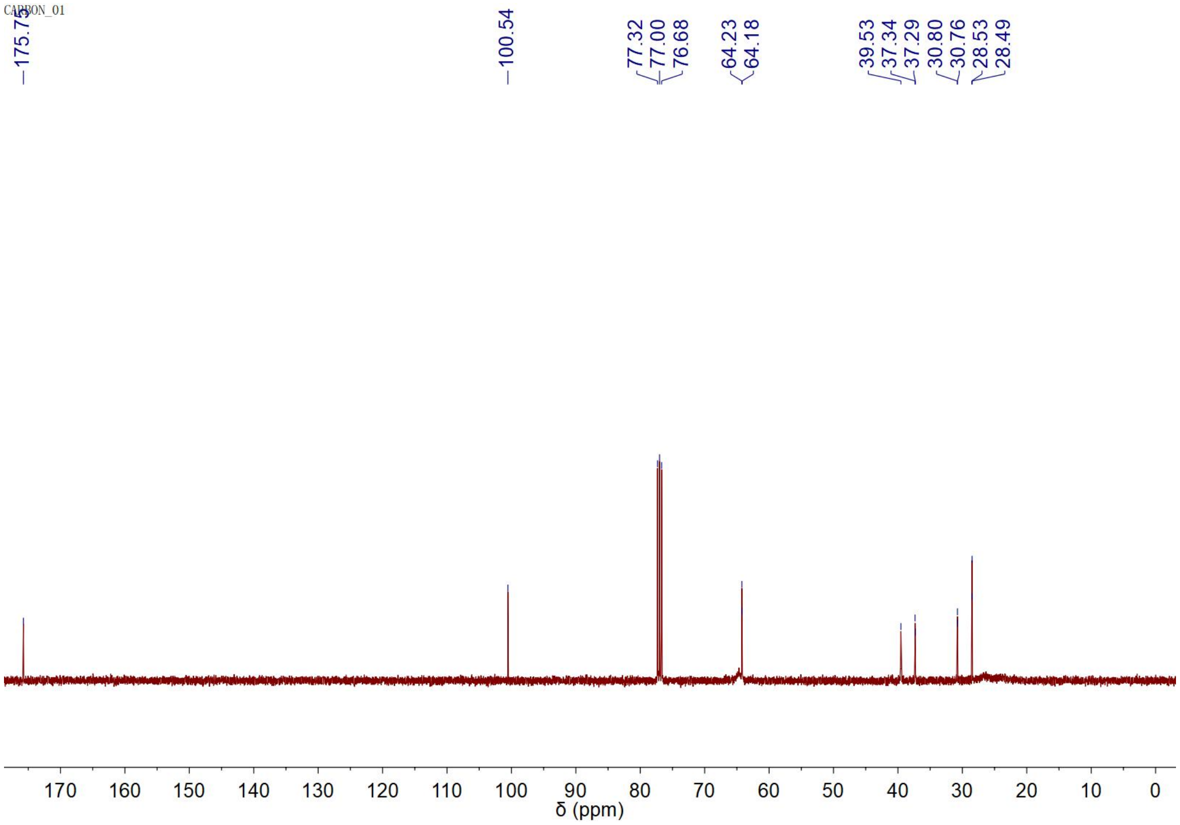

Supplementary Figure 26 ^{13}C NMR ($CDCl_3$, 25 °C) spectrum of **M16**.

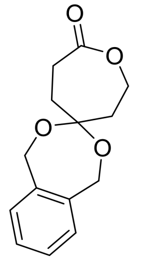

Characterizations of **M17**

Monomer Yield: 12 g; 55%; ^{1}H NMR (400 MHz, $CDCl_3$) δ 7.22–7.19 (m, 2H), 7.09–7.07 (m, 2H), 4.89 (s, 4H), 4.33–4.30 (m, 2H), 2.73–2.70 (m, 2H), 2.22–2.20 (m, 2H), 2.13–2.11 (m, 2H). ^{13}C NMR (100 MHz, $CDCl_3$) δ 175.3, 137.3, 127.0, 126.1, 101.6, 64.6, 64.0, 36.1, 29.4, 28.4. ESI-MS: calculated for $C_{14}H_{17}O_4$ $[M + H]^+$ 249.1121, found 249.1118.

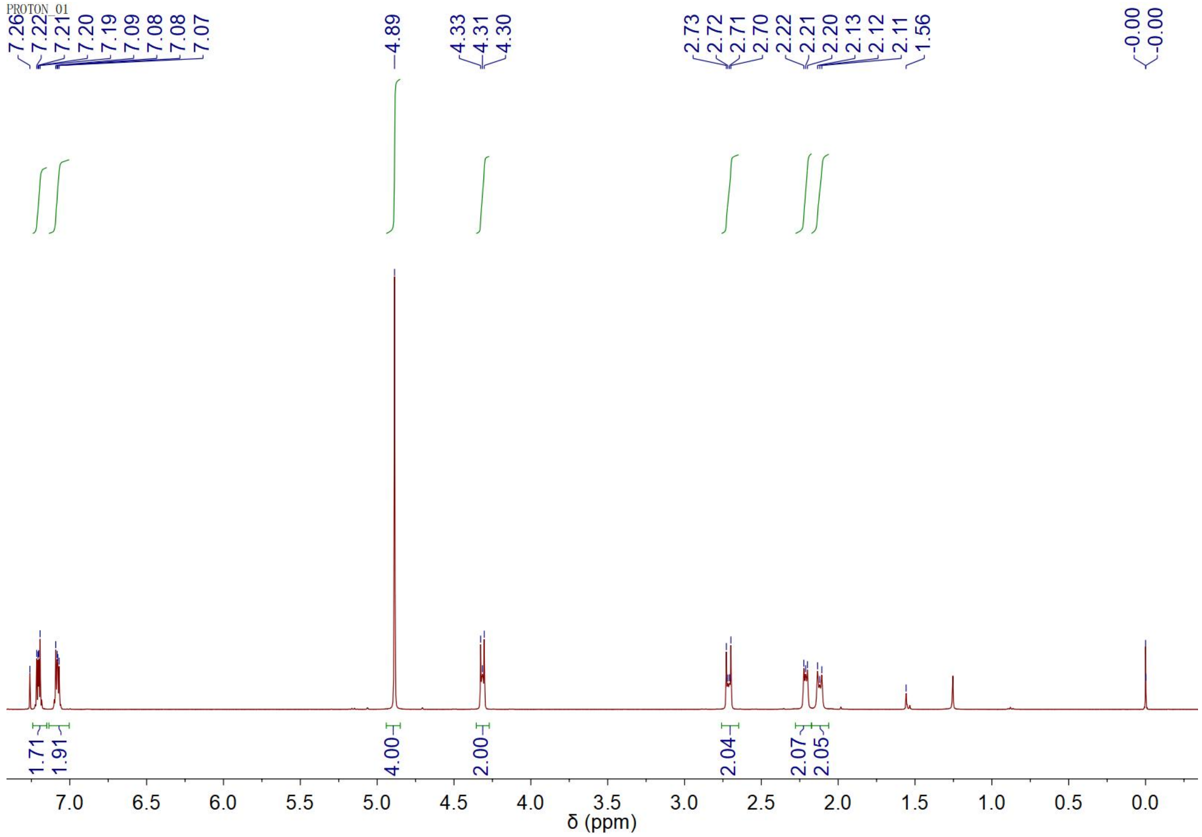

Supplementary Figure 27 ^{1}H NMR ($CDCl_3$, 25 °C) spectrum of **M17**.

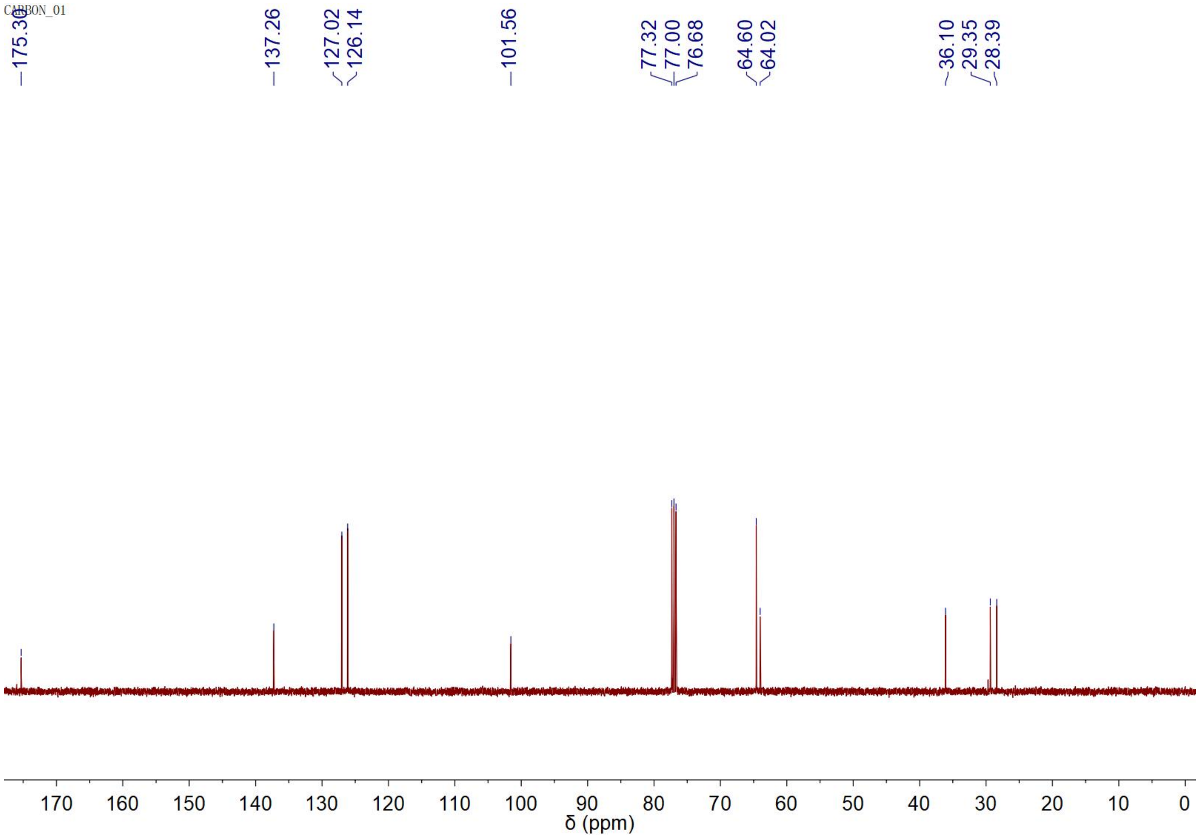

Supplementary Figure 28 ^{13}C NMR ($CDCl_3$, 25 °C) spectrum of **M17**.

General polymerization procedures

Polymerization of monomers

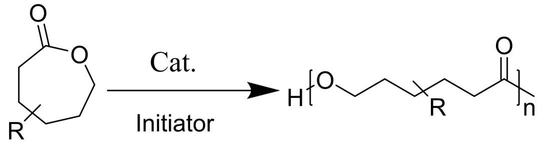

Supplementary Table 1. Results of ROP of monomers [a]

Run	[M]	Cat.	[M]/[Cat.]/[I]	Time (min)	Conv.[b] (%)	M_n[c] (kDa)	$Đ$[c] (M_w/M_n)
1	**M8**	**Y-1**	2000/1/1	3	98	110	1.11
2	**M10**	**Y-1**	2000/1/1	2.5	94	132	1.71
4	**M12**	**Y-1**	2000/1/1	2	94	242	1.95
5[d]	**M13**	**Y-1**	2000/1/1	5	92	149	1.27
6[e]	**M14**	**Y-1**	2000/1/1	5	93	280	1.63
7	**M16**	**Y-1**	2000/1/1	2	94	224	1.68
8	**M5**	**Y-1**	5000/1/1	2.5	87	210	1.16
9	**M9**	**Y-1**	5000/1/1	2.5	96	250	1.45
10[d]	**M10**	**Y-1**	5000/1/1	3	87	279	1.43
11	**M11**	**Y-1**	5000/1/1	2.5	94	257	1.22
12	**M12**	**Y-1**	5000/1/1	2	92	313	1.47
13	**M5**	**Y-1**	10000/1/1	11	85	270	1.31
14	**M11**	**Y-1**	10000/1/1	3	93	392	1.50
15	**M12**	**Y-1**	10000/1/1	10	76	252	1.26
16	**M5**	**Y-1**	20000/1/1	30	75	386	1.31
17[f]	**M9**	**Y-1**	20000/1/1	30	96	407	1.43
18[f]	**M11**	**Y-1**	20000/1/1	30	99	494	1.49
19[g]	**M1**	**$Sn(Oct)_2$**	1000/1/1	16 h	83	94.0	1.64
20[g]	**M2**	**$Sn(Oct)_2$**	1000/1/1	11 h	69	95.2	1.48
21[g]	**M3**	**$Sn(Oct)_2$**	1000/1/1	11 h	75	71.6	1.42
22[g]	**M4**	**$Sn(Oct)_2$**	1000/1/1	11 h	55	63.4	1.23
23[g]	**M5**	**$Sn(Oct)_2$**	1000/1/1	11 h	67	91.5	1.38
24[g]	**M6**	**$Sn(Oct)_2$**	1000/1/1	16 h	71	84.8	1.27
25[g]	**M8**	**$Sn(Oct)_2$**	1000/1/1	11 h	90	144.2	1.61

[a] Condition: Catalyst = **Y-1**, **M** = 200 mg, initiator (I) = *p*-tolylmethanol, solvent = THF, RT, C = 2 M. [b] Monomer conversion measured by ^1H NMR of the quenched solution. [c] Number-average molecular weight (M_n) and dispersity index ($Đ = M_w/M_n$), determined by size exclution chromatography (SEC) at 40 ºC in THF. [d] 60 °C. [e] Solvent = DCM, C = 1 M. [f] Reaction scale: **M** = 400 mg. [g] **M** = 100 mg, 100 °C, solvent = toluene, C = 4 M.

Supplementary Table 2. ROP results of **M7** at low temperature[a].

Run	[**M**]:[**Zn-1**]:[I]	C (mol/L)	Time (h)	Conv.[b] (%)	T (°C)
1	100:1:1	1.0	28	0	0
2	100:1:1	2.0	24	0	0
3	100:1:1	2.0	24	0	−30
4	100:1:1	2.0	26	0	−40

[a] Condition: Catalyst = **Zn-1**, **M7** = 100 mg, initiator (I) = *p*-tolylmethanol, solvent = THF.

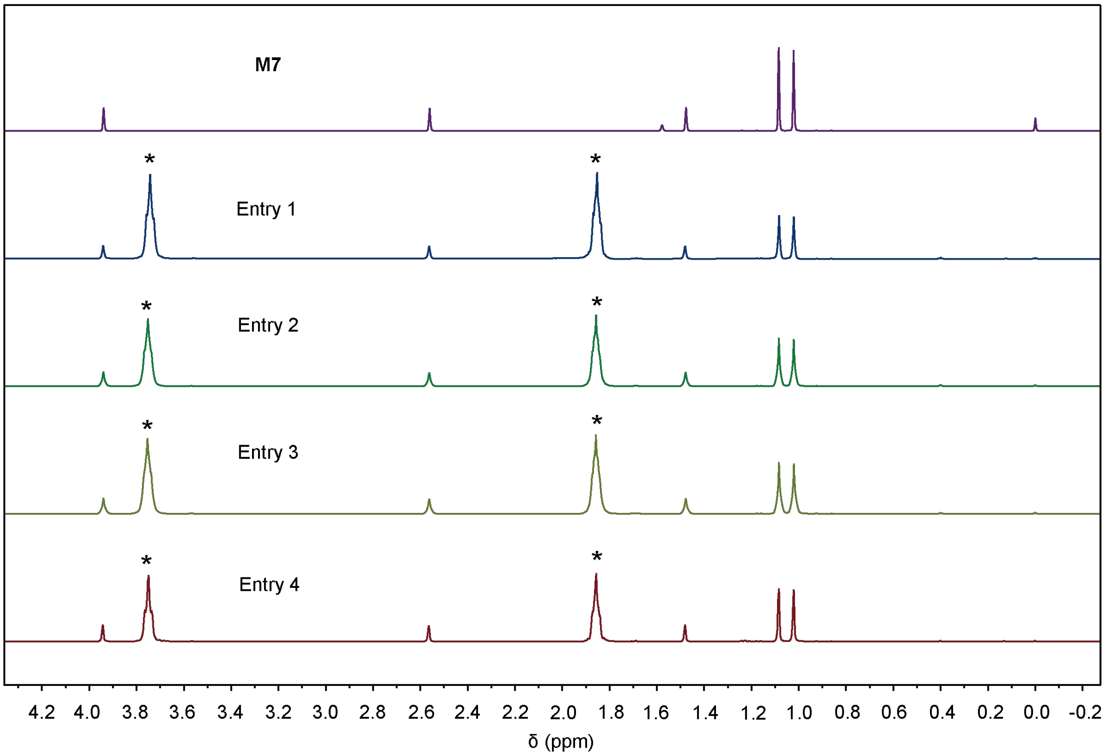

Supplementary Figure 29. ^{1}H NMR spectra of the ROP mixture of **M7** (entries 1−4). *Solvent.

Monomer conversion monitoring in polymerization process

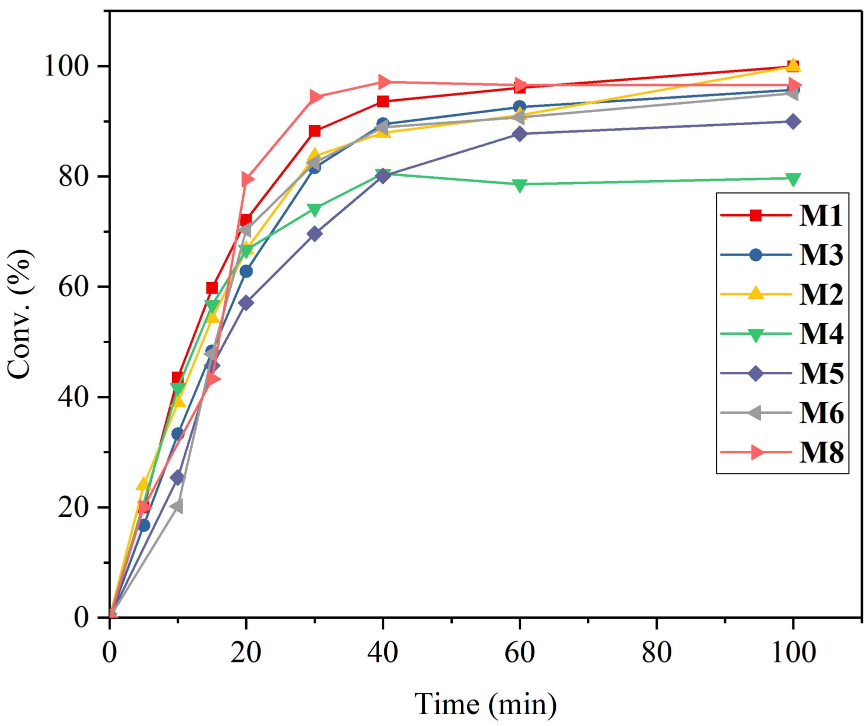

Supplementary Figure 30 The conversion vs. time results of **M1** - **M8** with a [**M**]:[**Zn-1**]:[I] ratio of 1000:1:1, C = 1 M and RT.

NMR Spectra of Produced Polymers

Characterizations of P(**M1**)

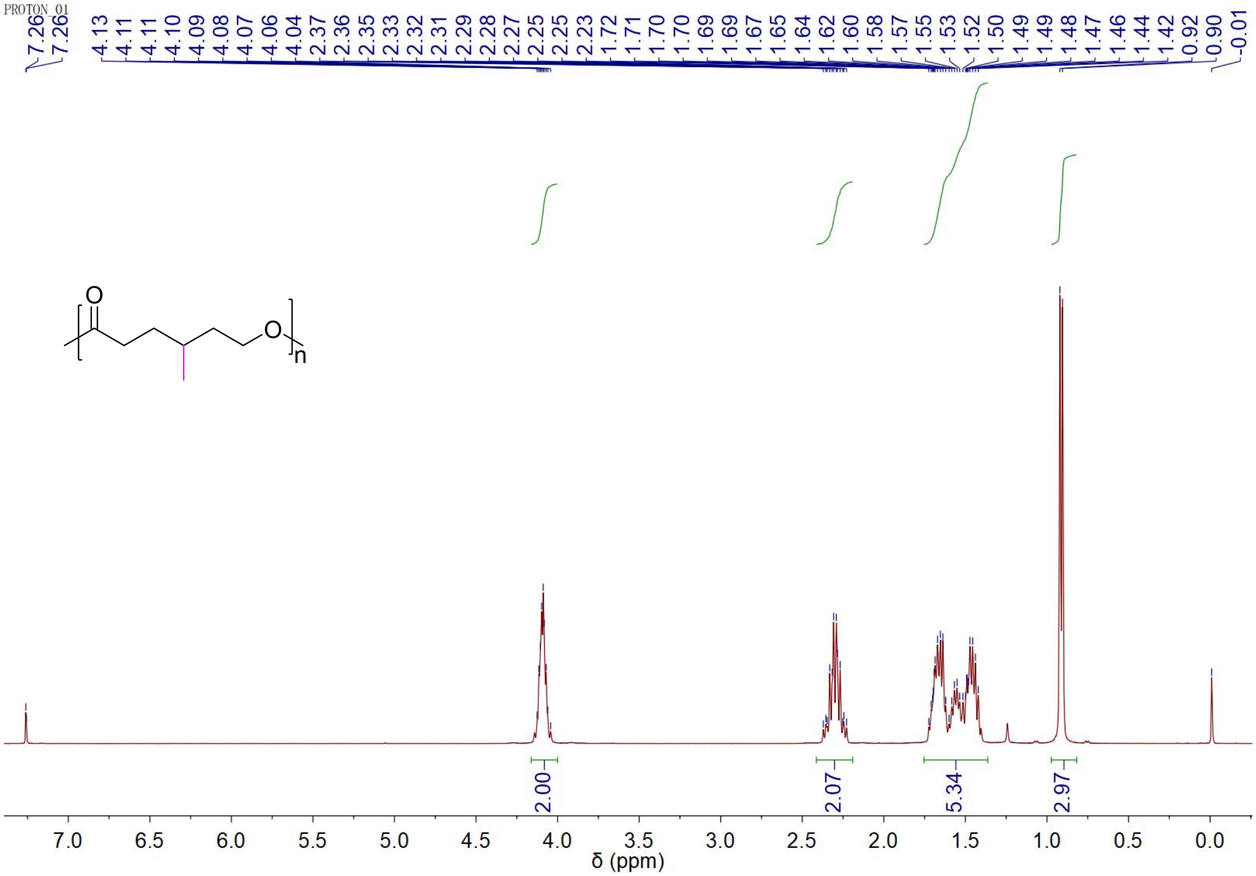

Supplementary Figure 31 ^{1}H NMR ($CDCl_3$, 25 °C) spectrum of P(**M1**) obtained by [**M1**]/[**Zn-1**]/[I] = 1000/1/1.

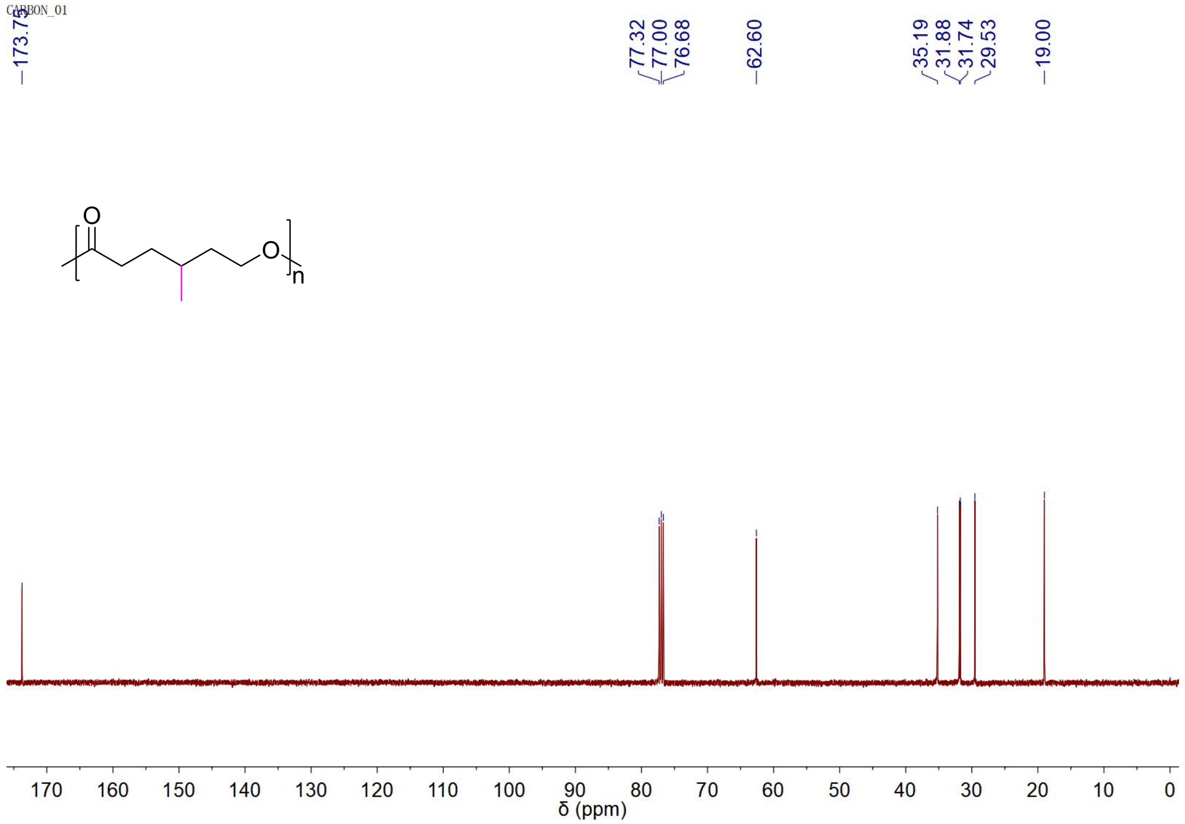

Supplementary Figure 32 ^{13}C NMR ($CDCl_3$, 25 °C) spectrum of P(**M1**) obtained by [**M1**]/[**Zn-1**]/[I] = 1000/1/1.

Characterizations of P(**M2**)

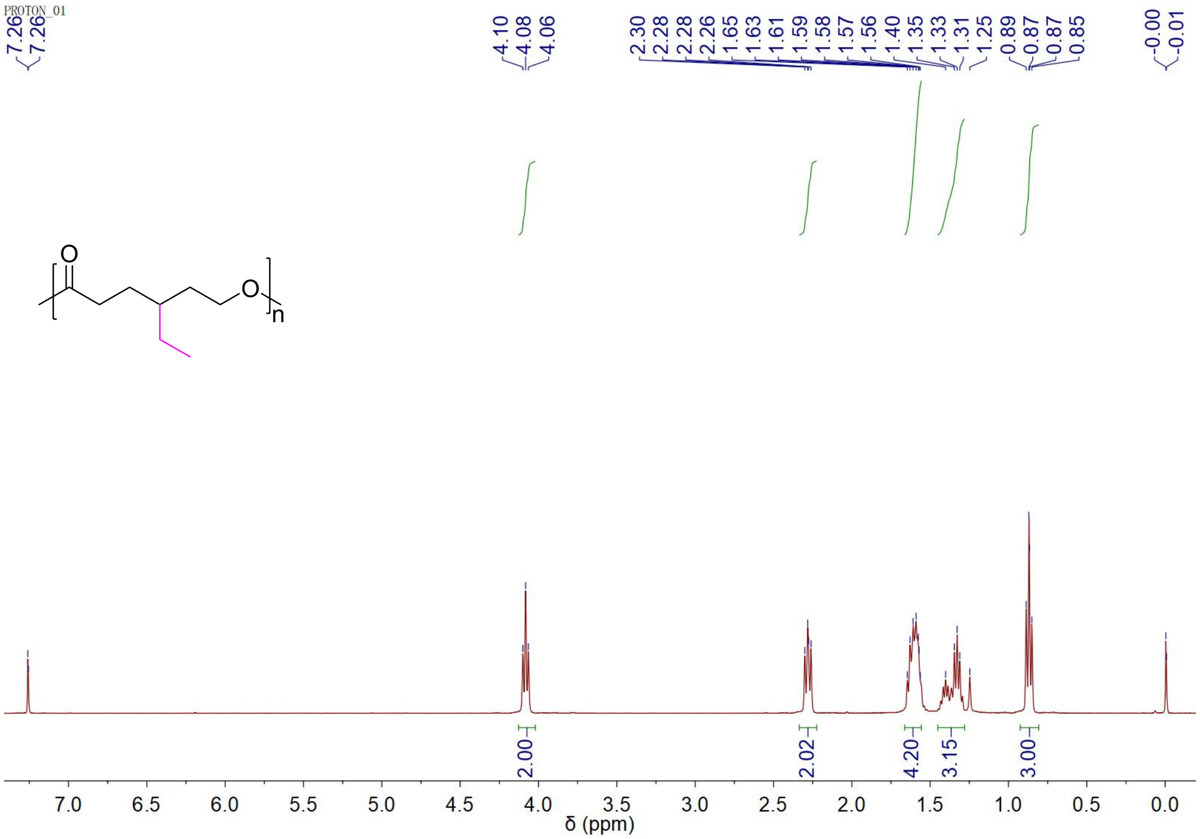

Supplementary Figure 33 ^{1}H NMR ($CDCl_3$, 25 °C) spectrum of P(**M2**) obtained by [**M2**]/[**Zn-1**]/[I] = 1000/1/1.

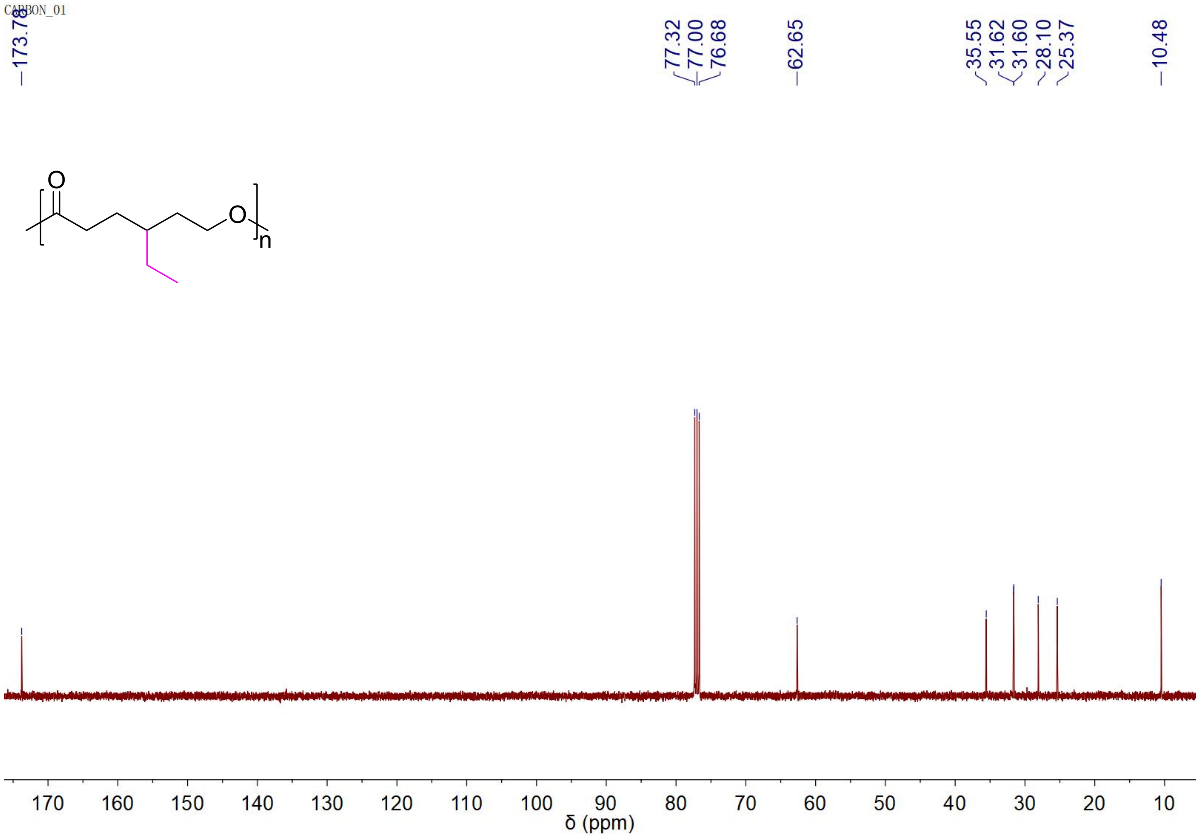

Supplementary Figure 34 ^{13}C NMR ($CDCl_3$, 25 °C) spectrum of P(**M2**) obtained by [**M2**]/[**Zn-1**]/[I] = 1000/1/1.

<u>Characterizations of P(**M3**)</u>

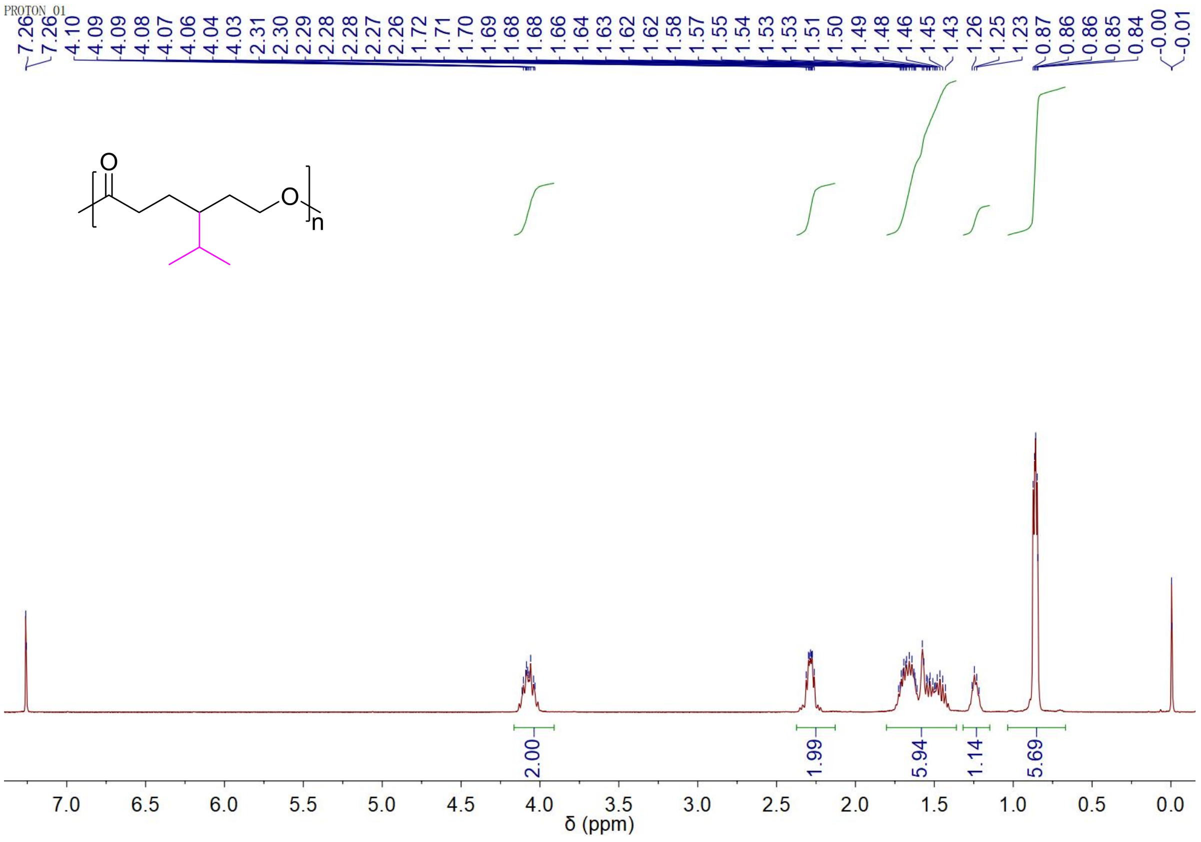

Supplementary Figure 35 ^{1}H NMR ($CDCl_3$, 25 °C) spectrum of P(**M3**) obtained by [**M3**]/[**Zn-1**]/[I] = 1000/1/1.

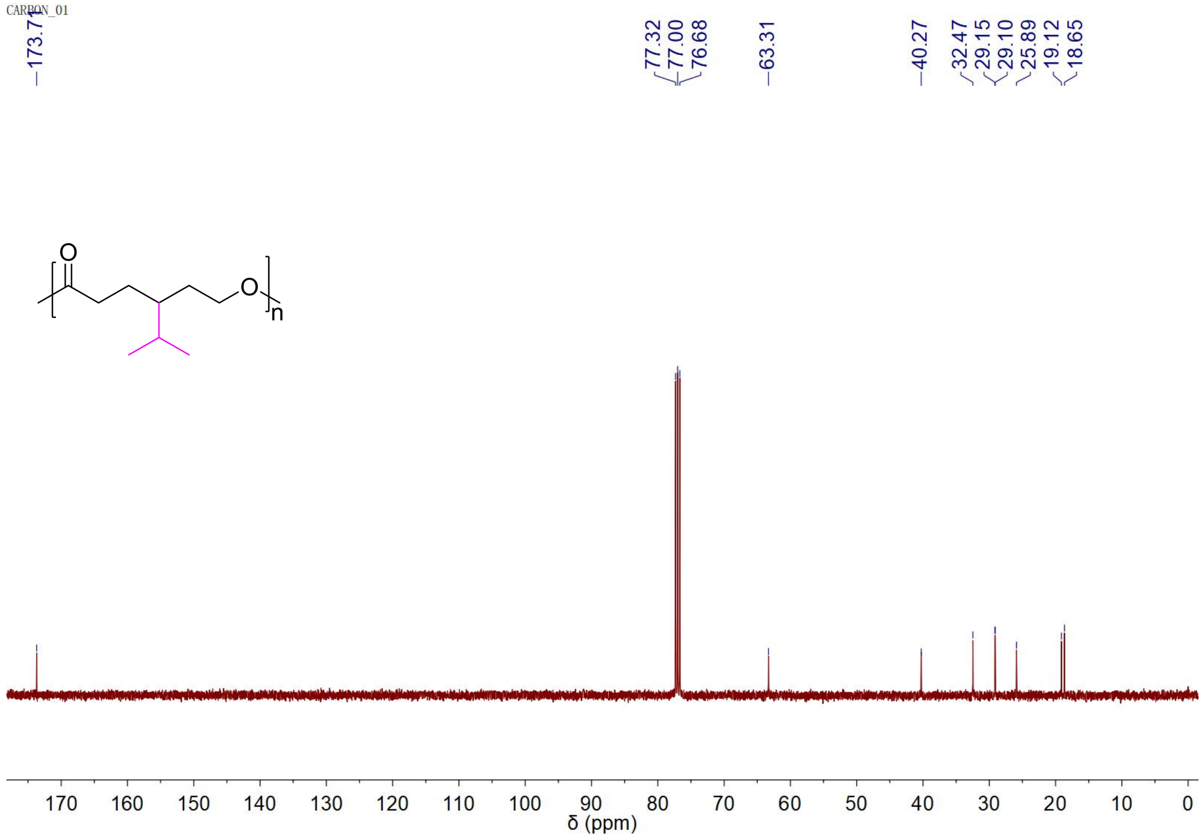

Supplementary Figure 36 ^{13}C NMR ($CDCl_3$, 25 °C) spectrum of P(**M3**) obtained by [**M3**]/[**Zn-1**]/[I] = 1000/1/1.

Characterizations of P(**M4**)

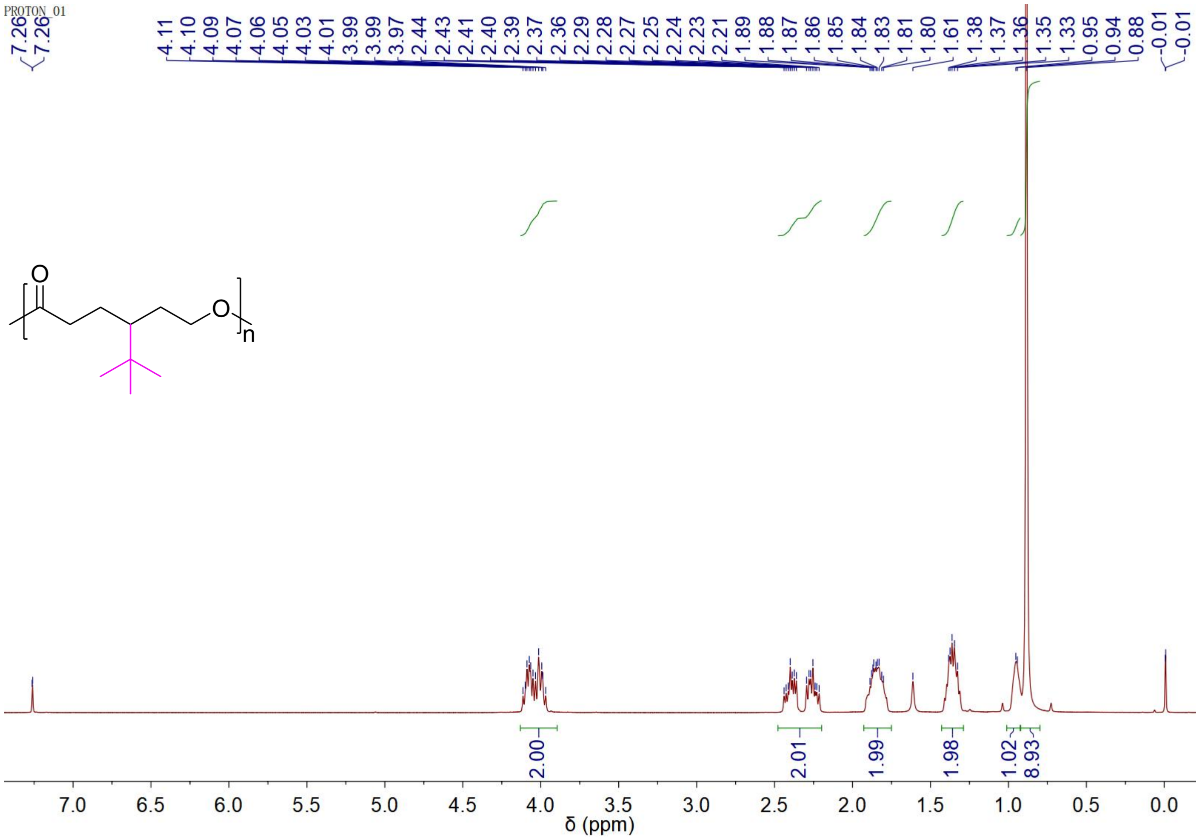

Supplementary Figure 37 ^{1}H NMR ($CDCl_3$, 25 °C) spectrum of P(**M4**) obtained by [**M4**]/[**Zn-1**]/[I] = 1000/1/1.

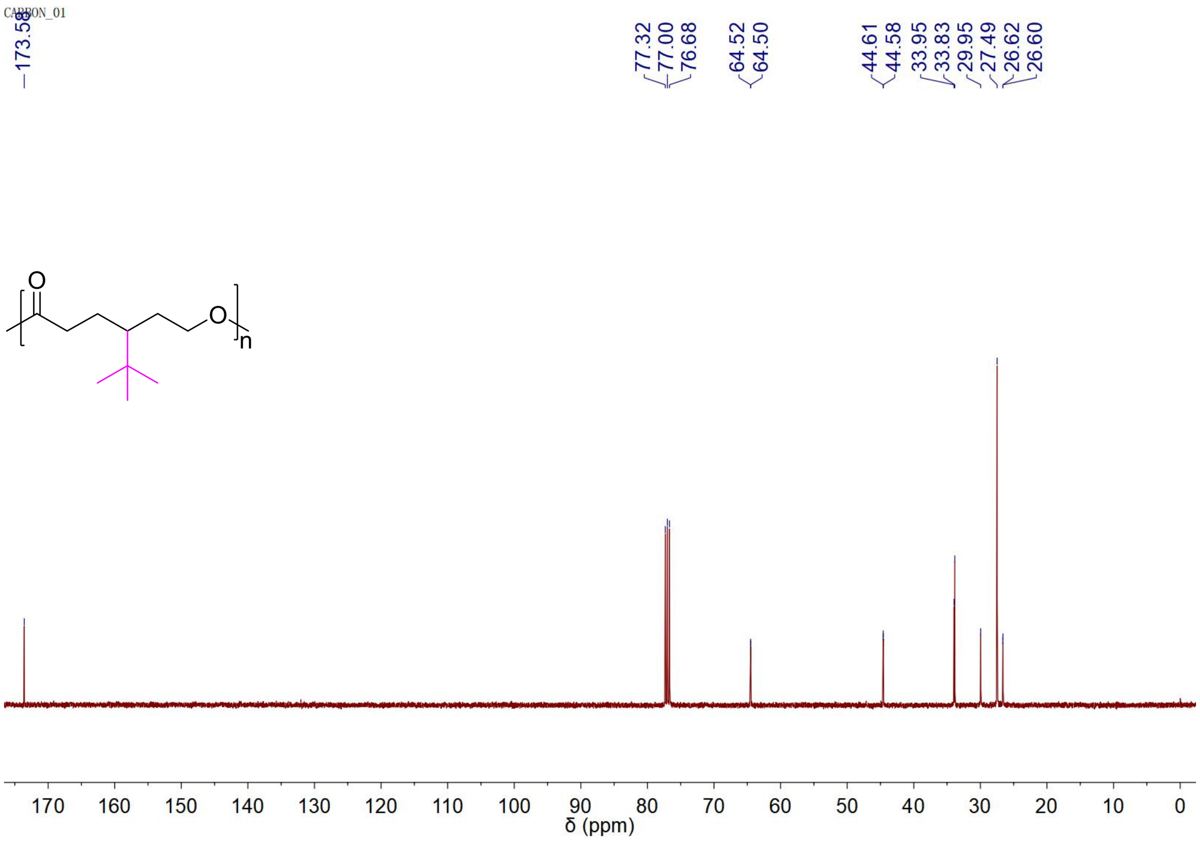

Supplementary Figure 38 ^{13}C NMR ($CDCl_3$, 25 °C) spectrum of P(**M4**) obtained by [**M4**]/[**Zn-1**]/[I] = 1000/1/1.

Characterizations of P(**M5**)

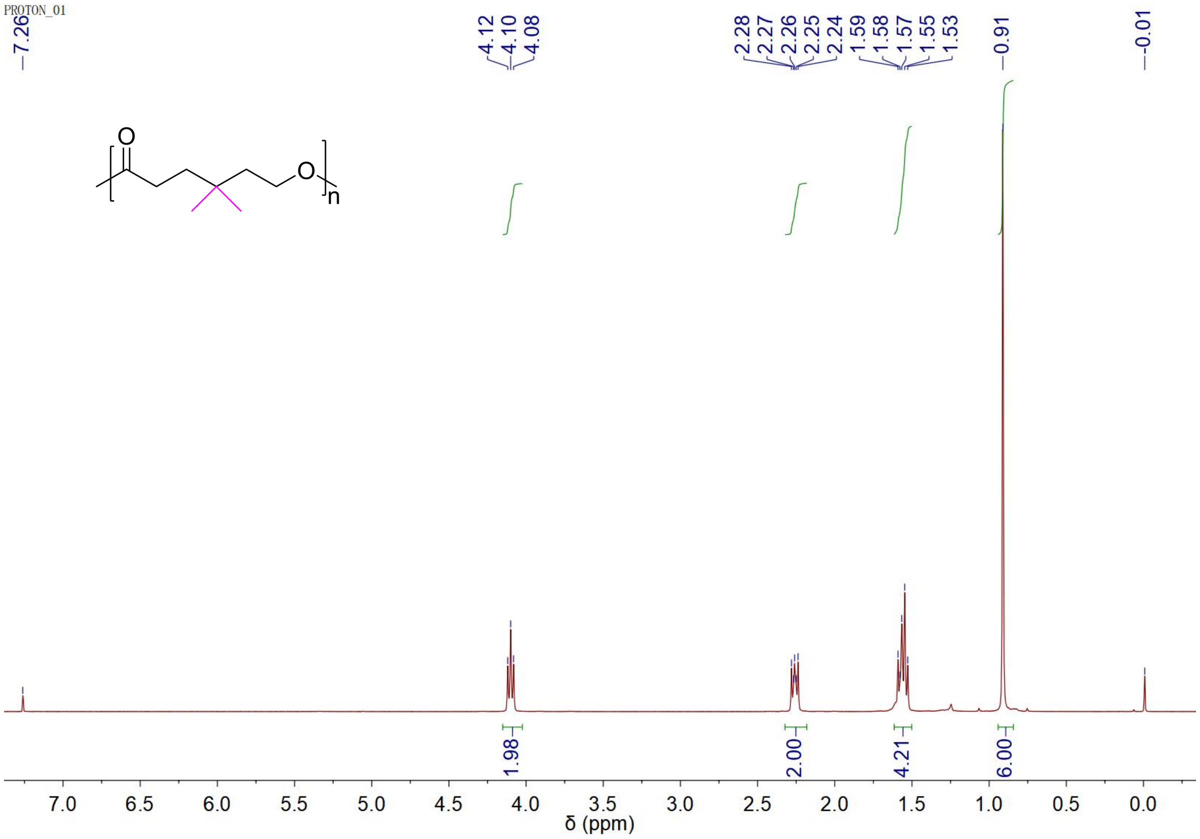

Supplementary Figure 39 ^{1}H NMR ($CDCl_3$, 25 °C) spectrum of P(**M5**) obtained by [**M5**]/[**Zn-1**]/[I] = 1000/1/1.

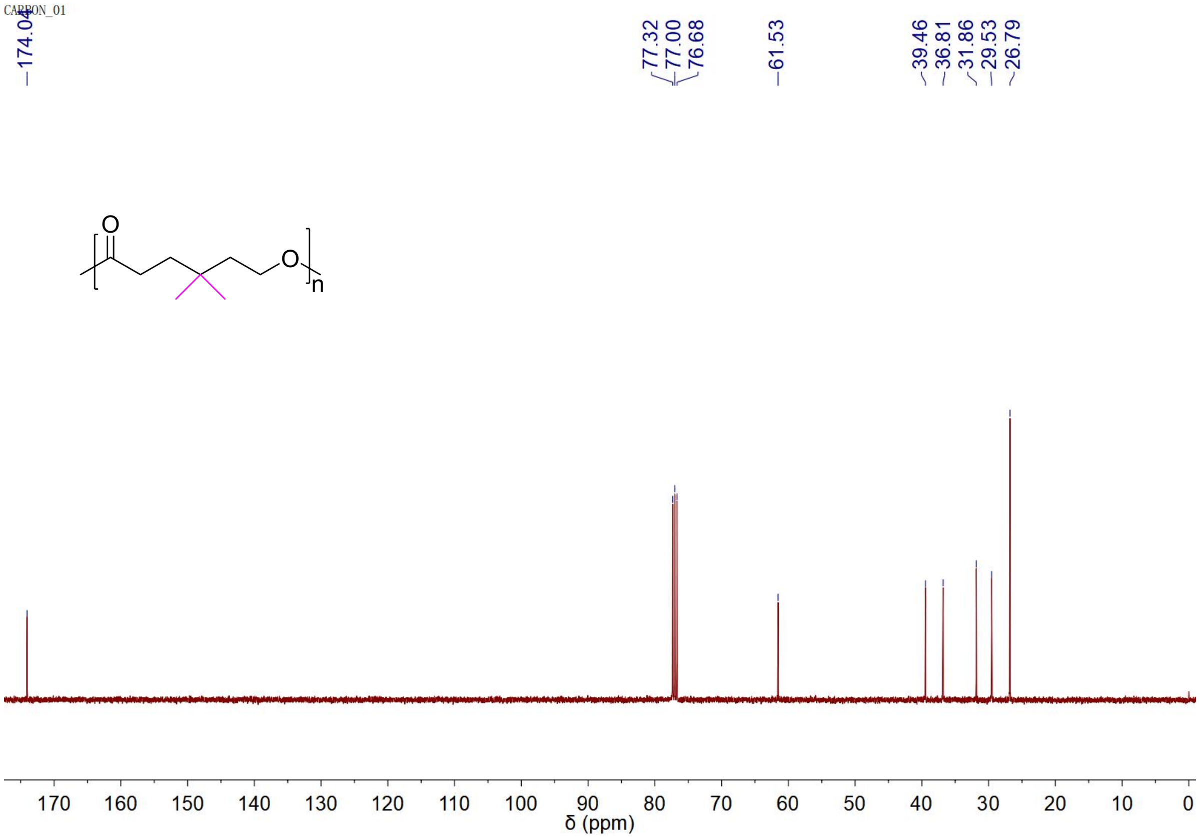

Supplementary Figure 40 ^{13}C NMR ($CDCl_3$, 25 °C) spectrum of P(**M5**) obtained by [**M5**]/[**Zn-1**]/[I] = 1000/1/1.

Characterizations of P(**M6**)

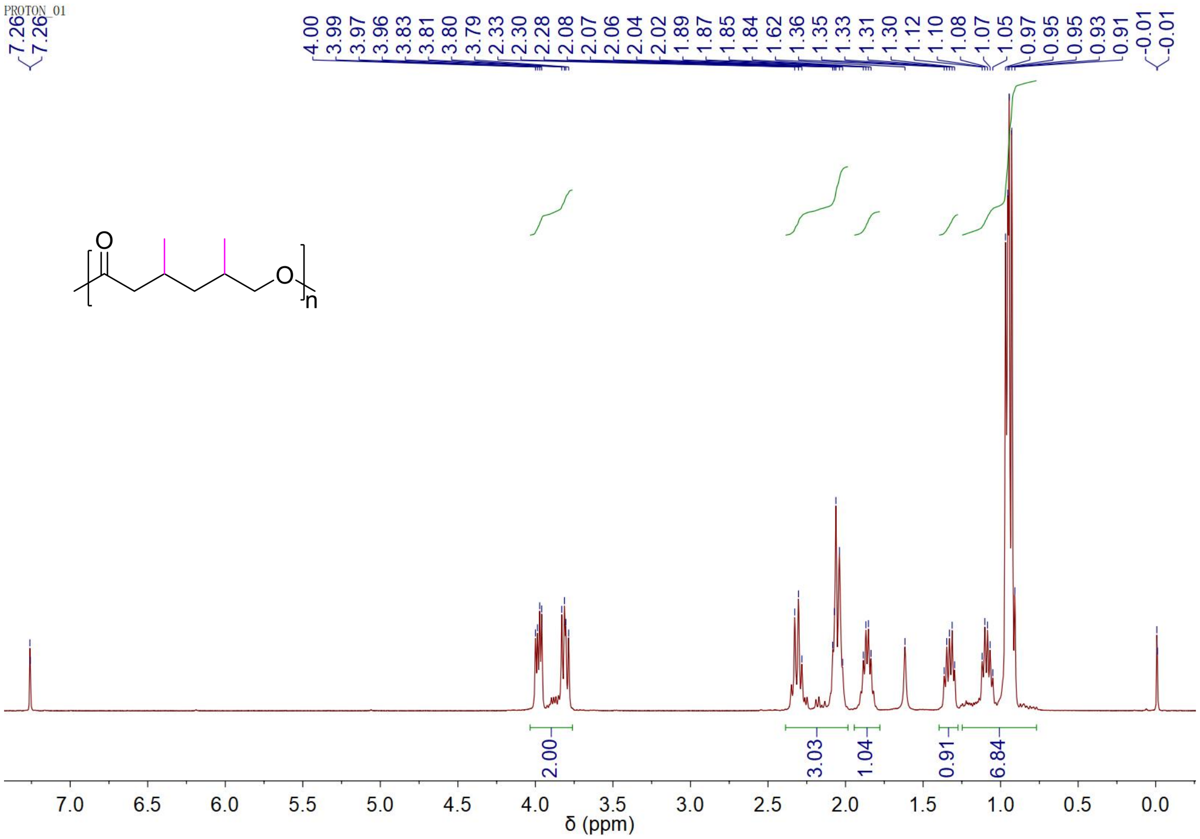

Supplementary Figure 41 ^{1}H NMR ($CDCl_3$, 25 °C) spectrum of P(**M6**) obtained by [**M6**]/[**Zn-1**]/[I] = 1000/1/1.

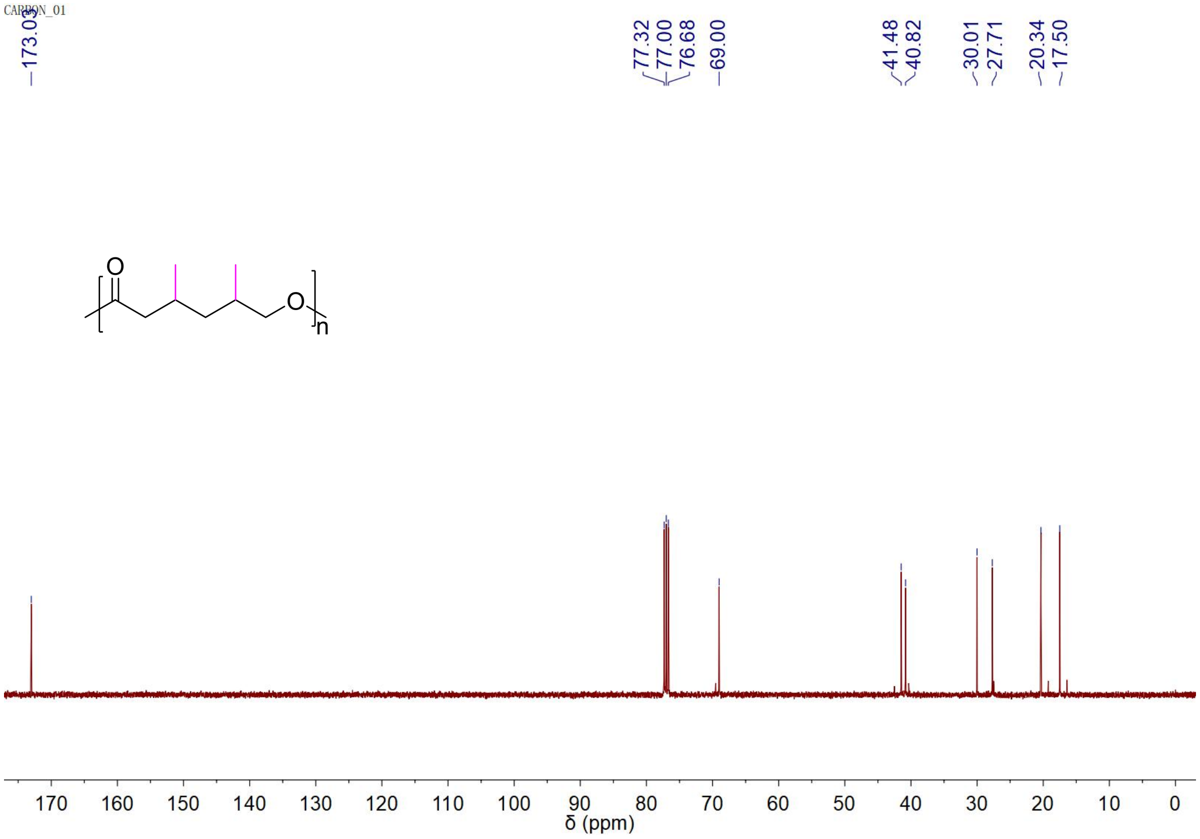

Supplementary Figure 42 ^{13}C NMR ($CDCl_3$, 25 °C) spectrum of P(**M6**) obtained by [**M6**]/[**Zn-1**]/[I] = 1000/1/1.

Characterizations of P(**M8**)

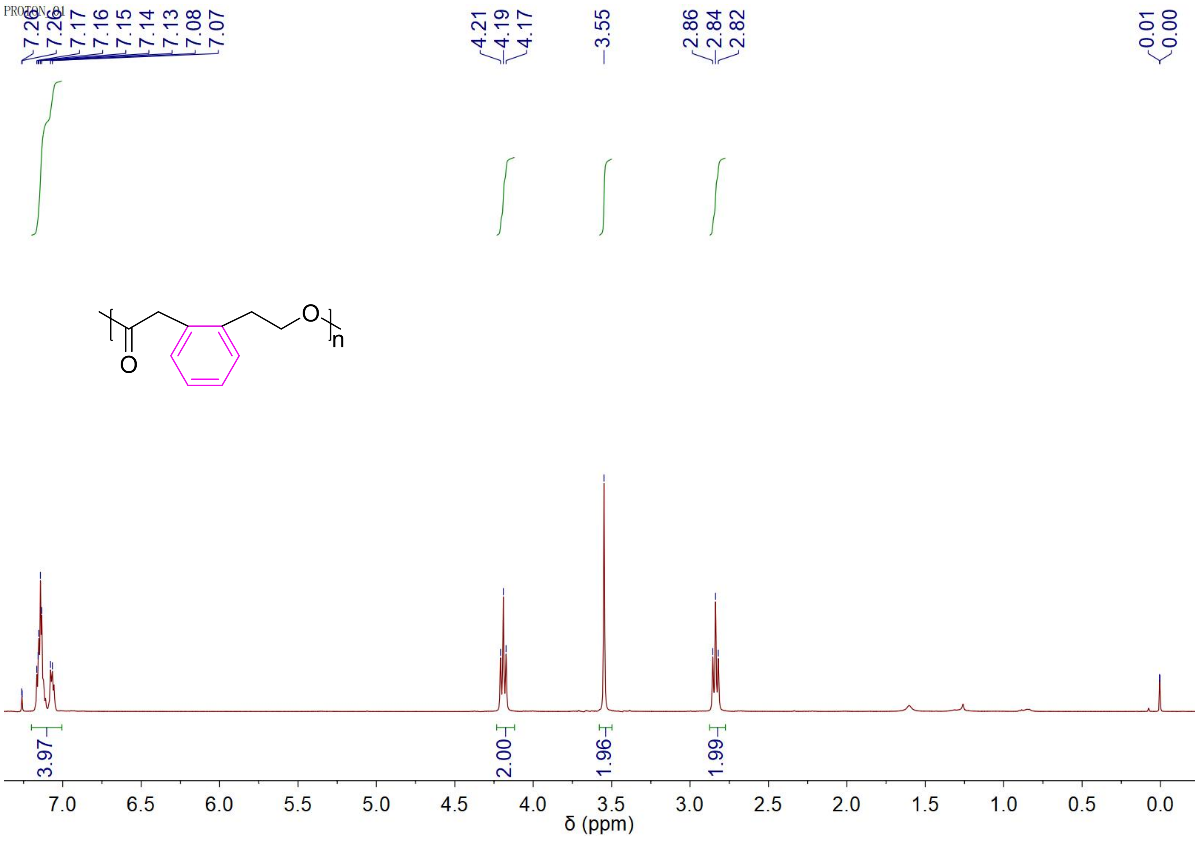

Supplementary Figure 43 ^{1}H NMR ($CDCl_3$, 25 °C) spectrum of P(**M8**) obtained by [**M8**]/[**Zn-1**]/[I] = 1000/1/1.

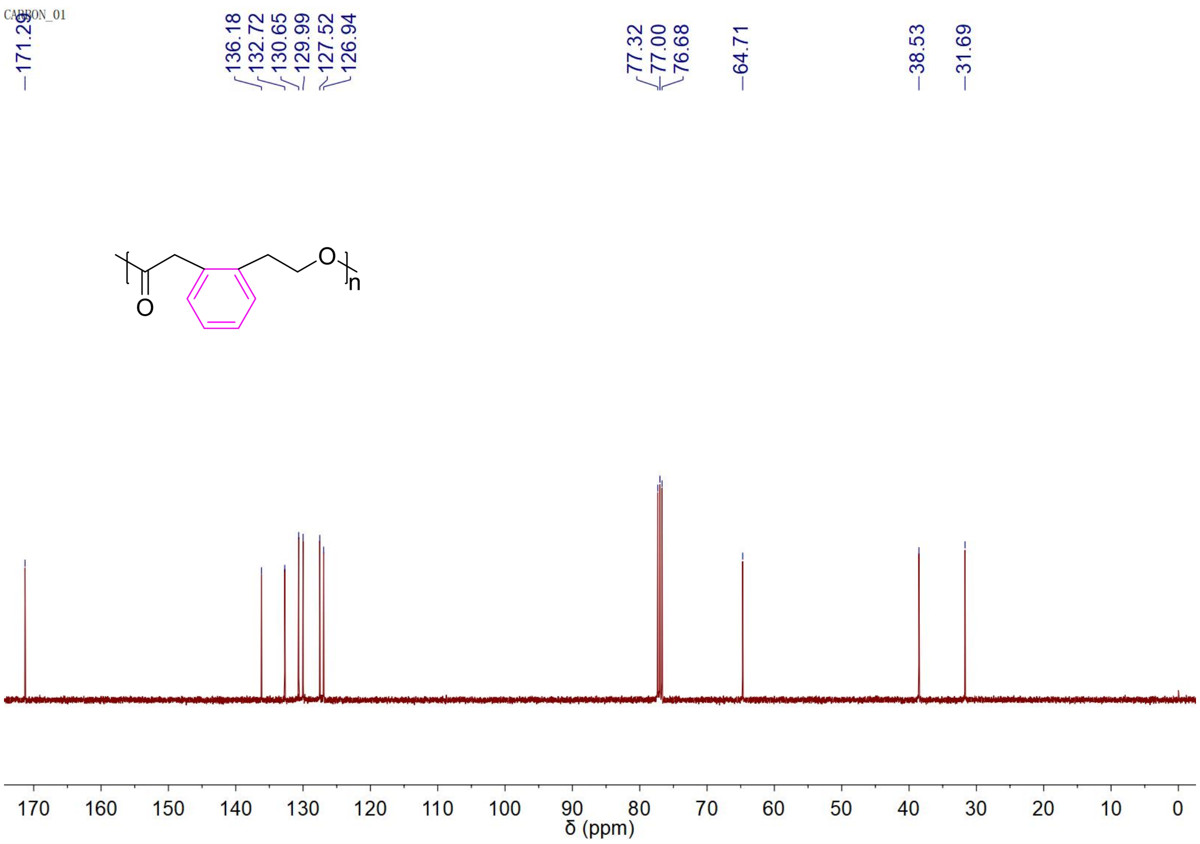

Supplementary Figure 44 ^{13}C NMR ($CDCl_3$, 25 °C) spectrum of P(**M8**) obtained by [**M8**]/[**Zn-1**]/[I] = 1000/1/1.

Characterizations of P(**M9**)

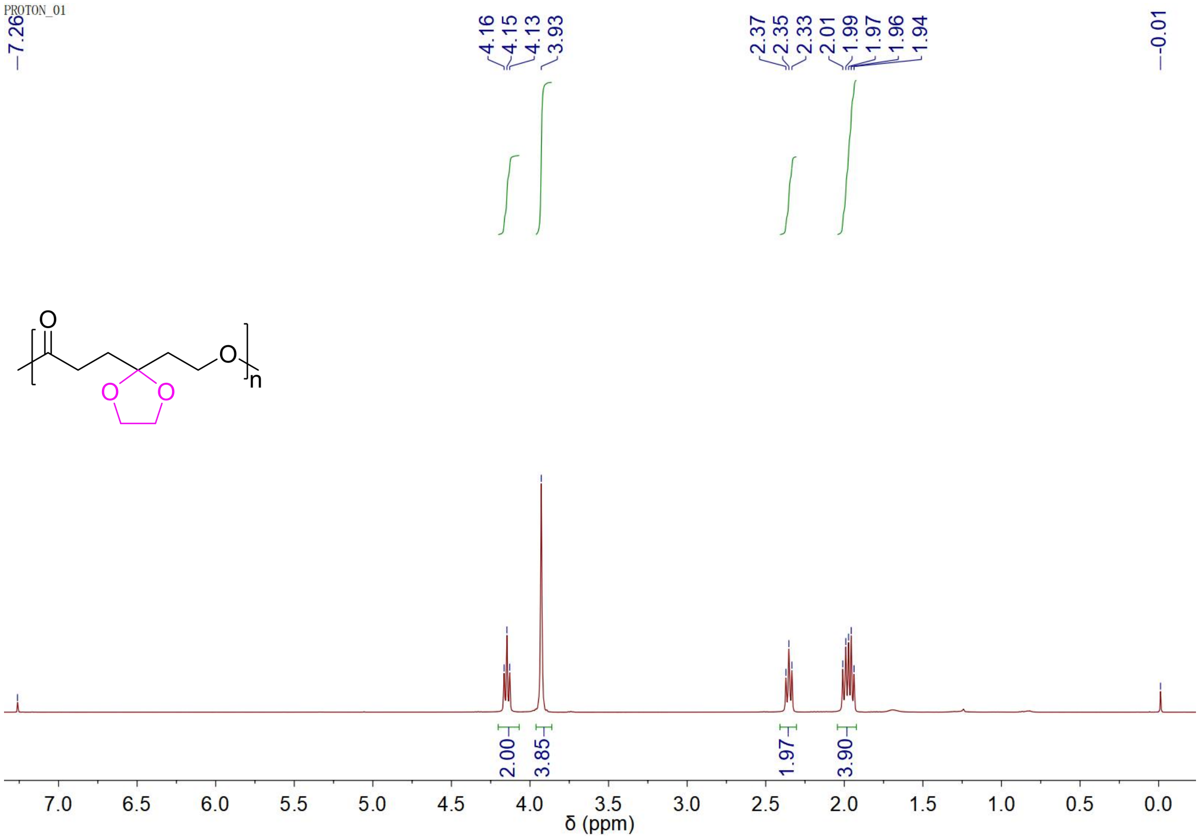

Supplementary Figure 45 ^{1}H NMR ($CDCl_3$, 25 °C) spectrum of P(**M9**) obtained by [**M9**]/[**Zn-1**]/[I] = 1000/1/1.

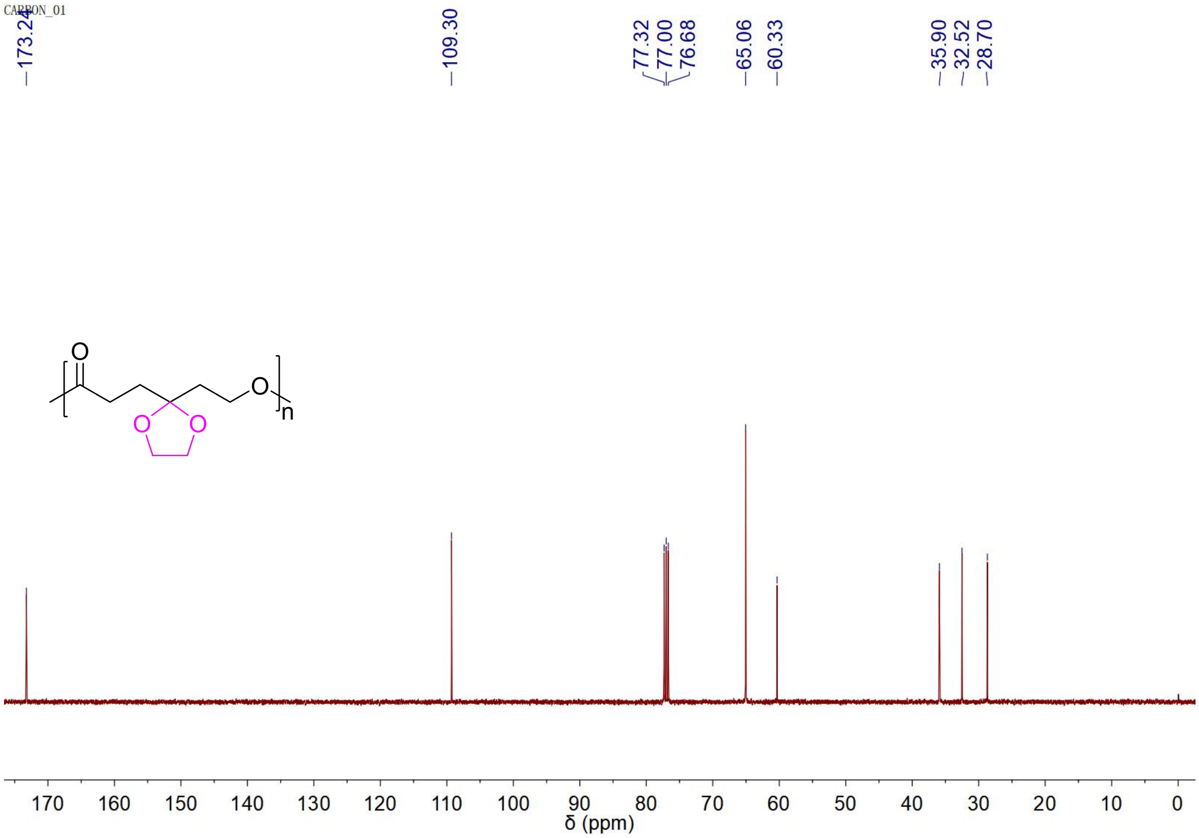

Supplementary Figure 46 ^{13}C NMR ($CDCl_3$, 25 °C) spectrum of P(**M9**) obtained by [**M9**]/[**Zn-1**]/[I] = 1000/1/1.

Characterizations of P(**M10**)

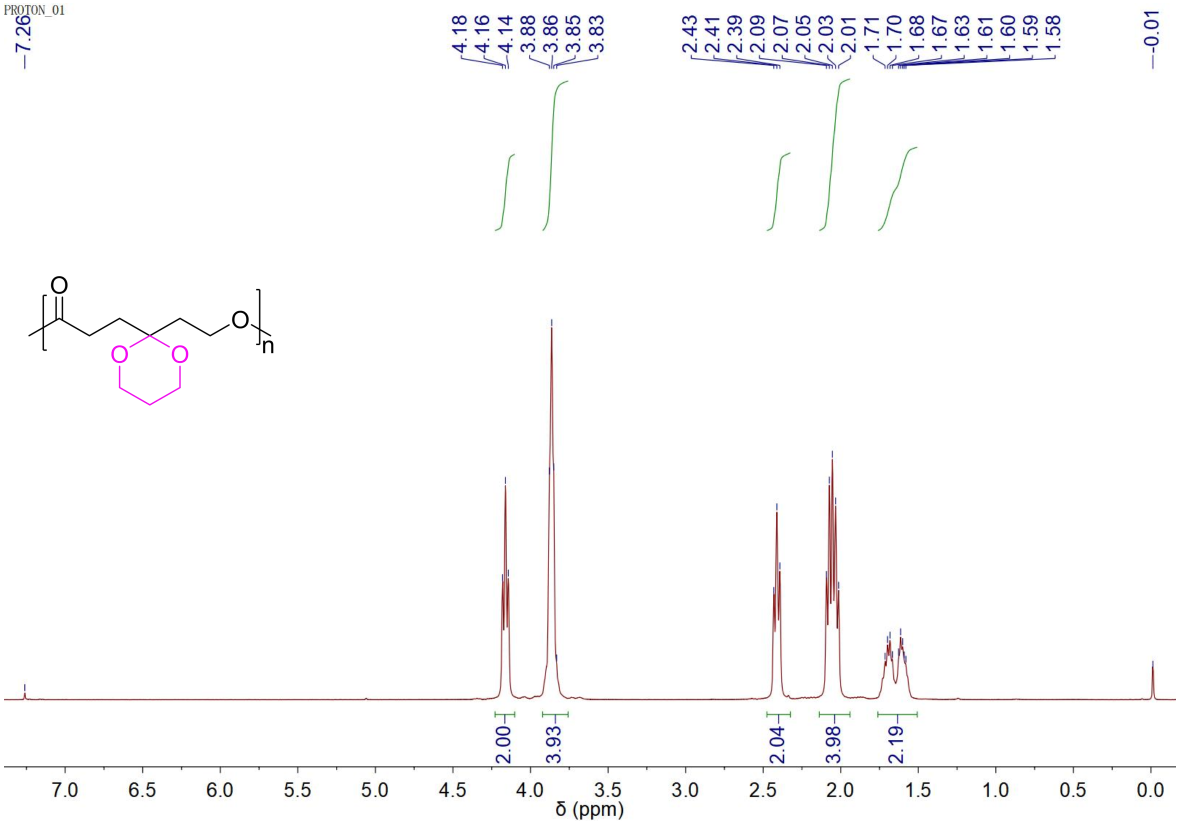

Supplementary Figure 47 ^{1}H NMR ($CDCl_3$, 25 °C) spectrum of P(**M10**) obtained by [**M10**]/[**Zn-1**]/[I] = 1000/1/1.

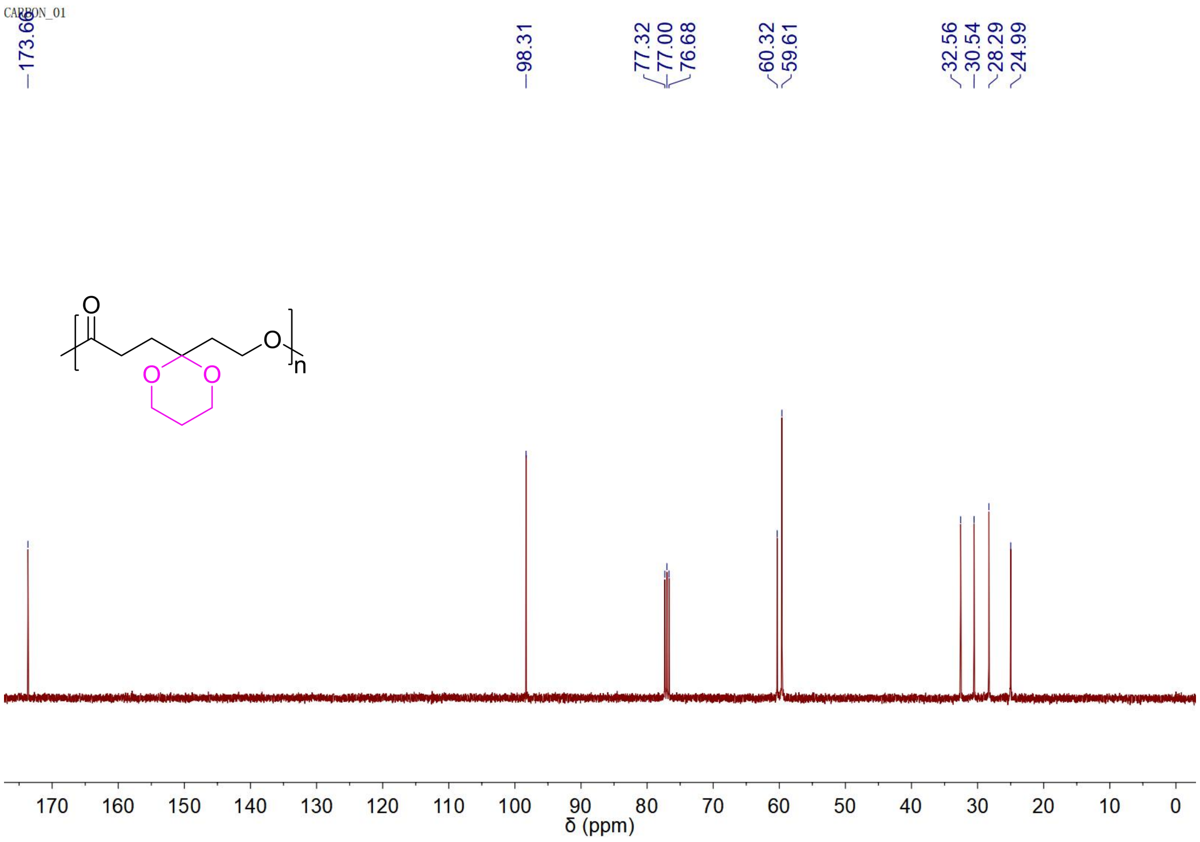

Supplementary Figure 48 ^{13}C NMR ($CDCl_3$, 25 °C) spectrum of P(**M10**) obtained by [**M10**]/[**Zn-1**]/[I] = 1000/1/1.

Characterizations of P(**M11**)

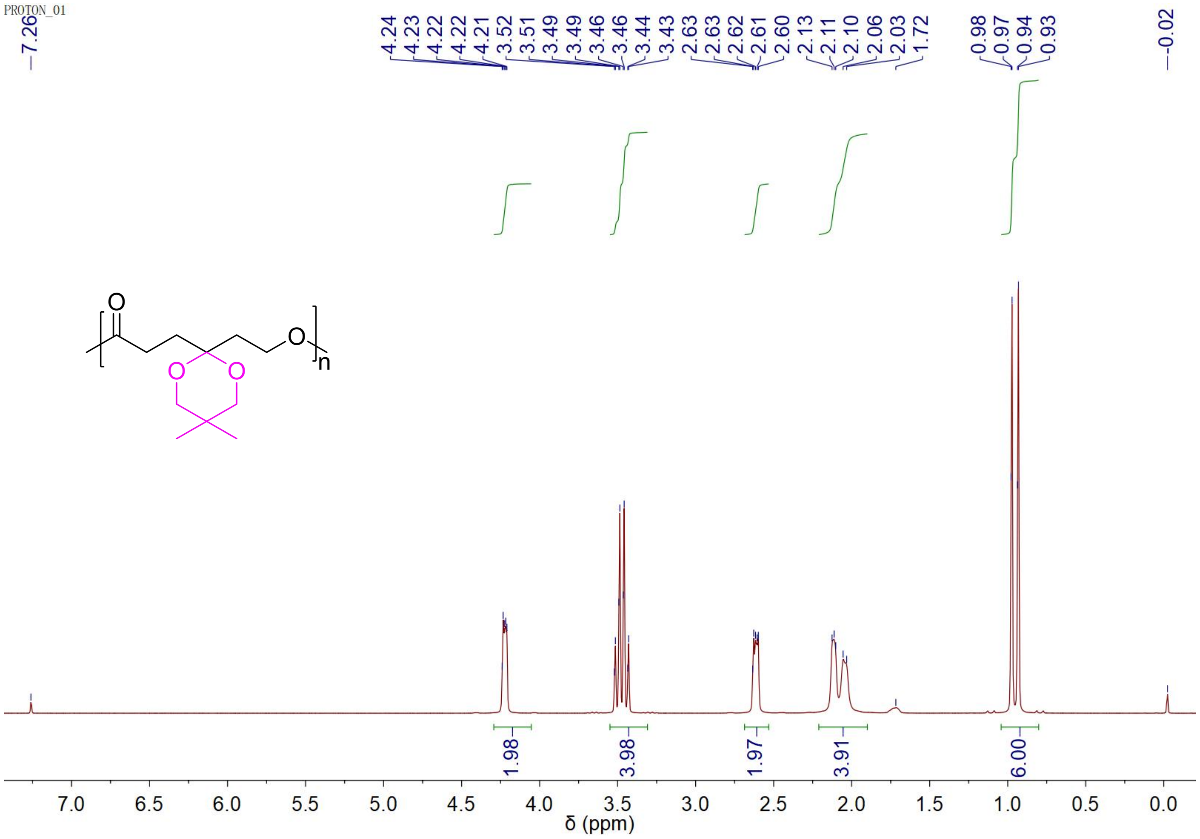

Supplementary Figure 49 ^{1}H NMR ($CDCl_3$, 25 °C) spectrum of P(**M11**) obtained by [**M11**]/[**Zn-1**]/[I] = 1000/1/1.

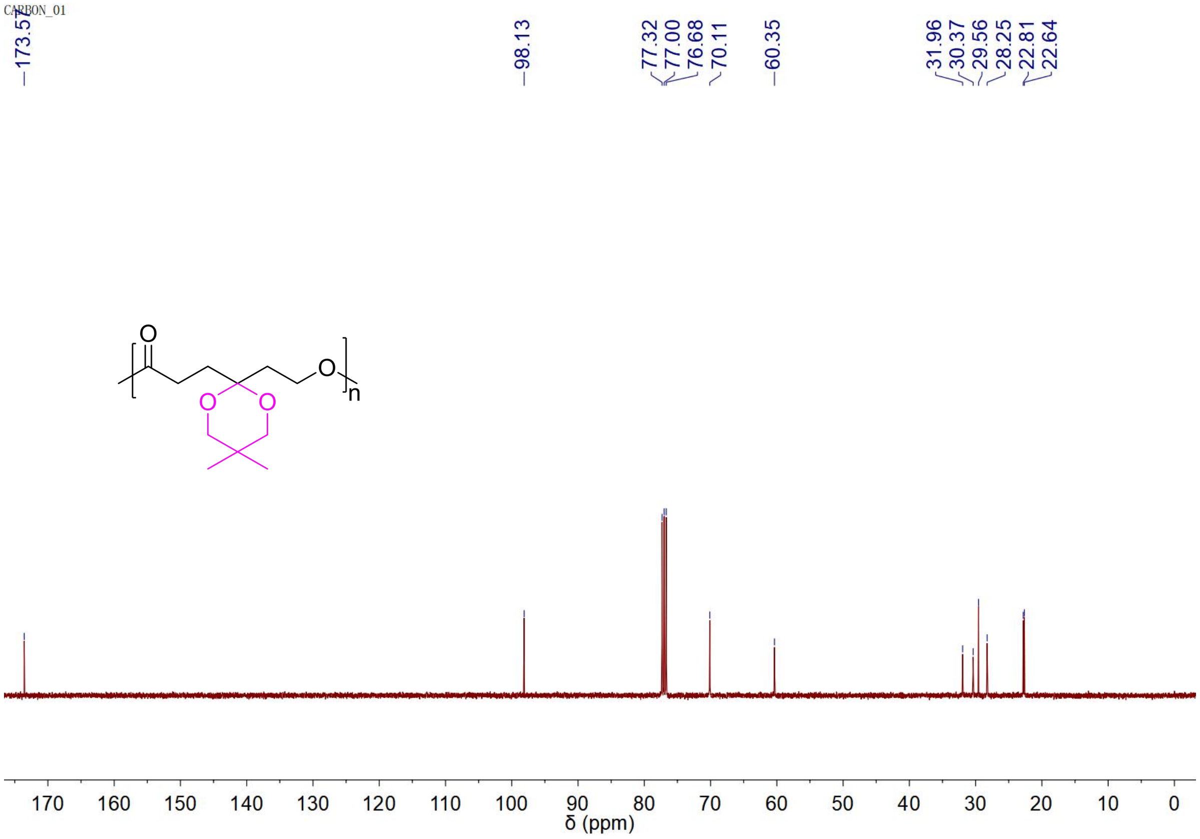

Supplementary Figure 50 ^{13}C NMR ($CDCl_3$, 25 °C) spectrum of P(**M11**) obtained by [**M11**]/[**Zn-1**]/[I] = 1000/1/1.

Characterizations of P(**M12**)

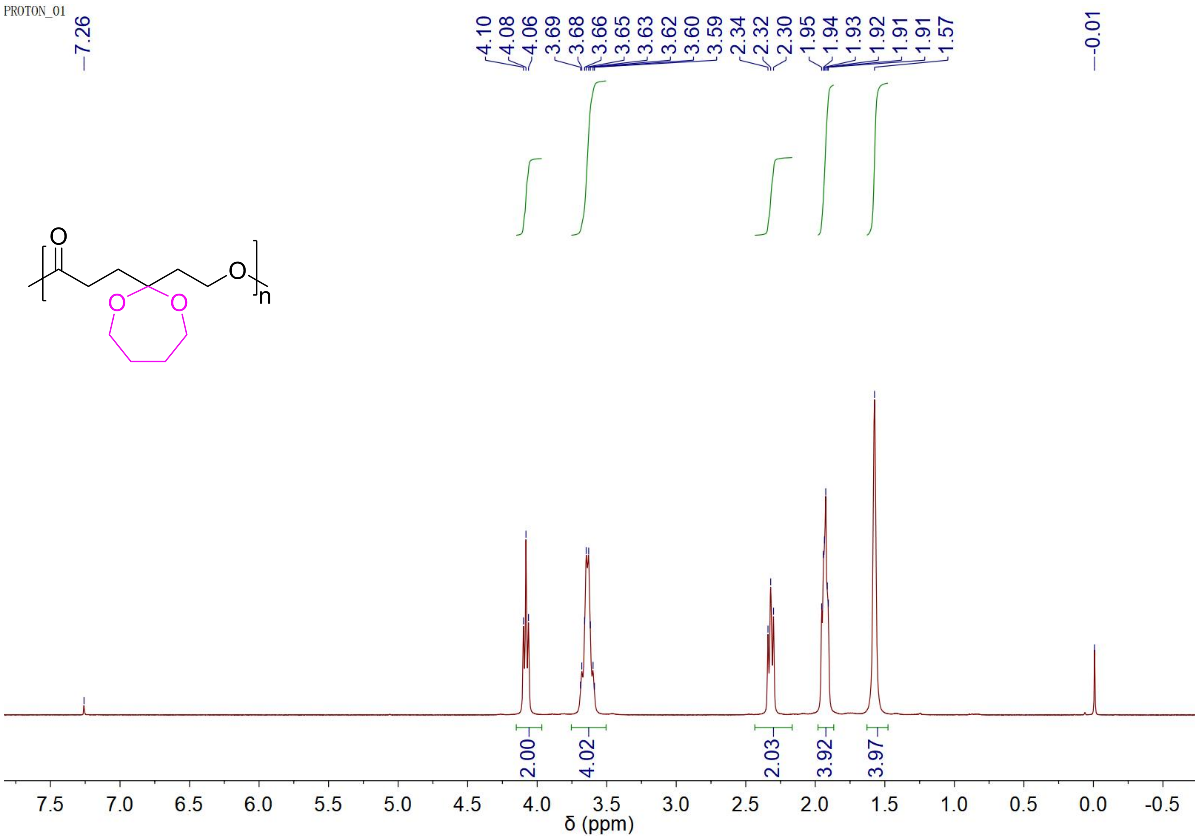

Supplementary Figure 51 ^{1}H NMR ($CDCl_3$, 25 °C) spectrum of P(**M12**) obtained by [**M12**]/[**Zn-1**]/[I] = 1000/1/1.

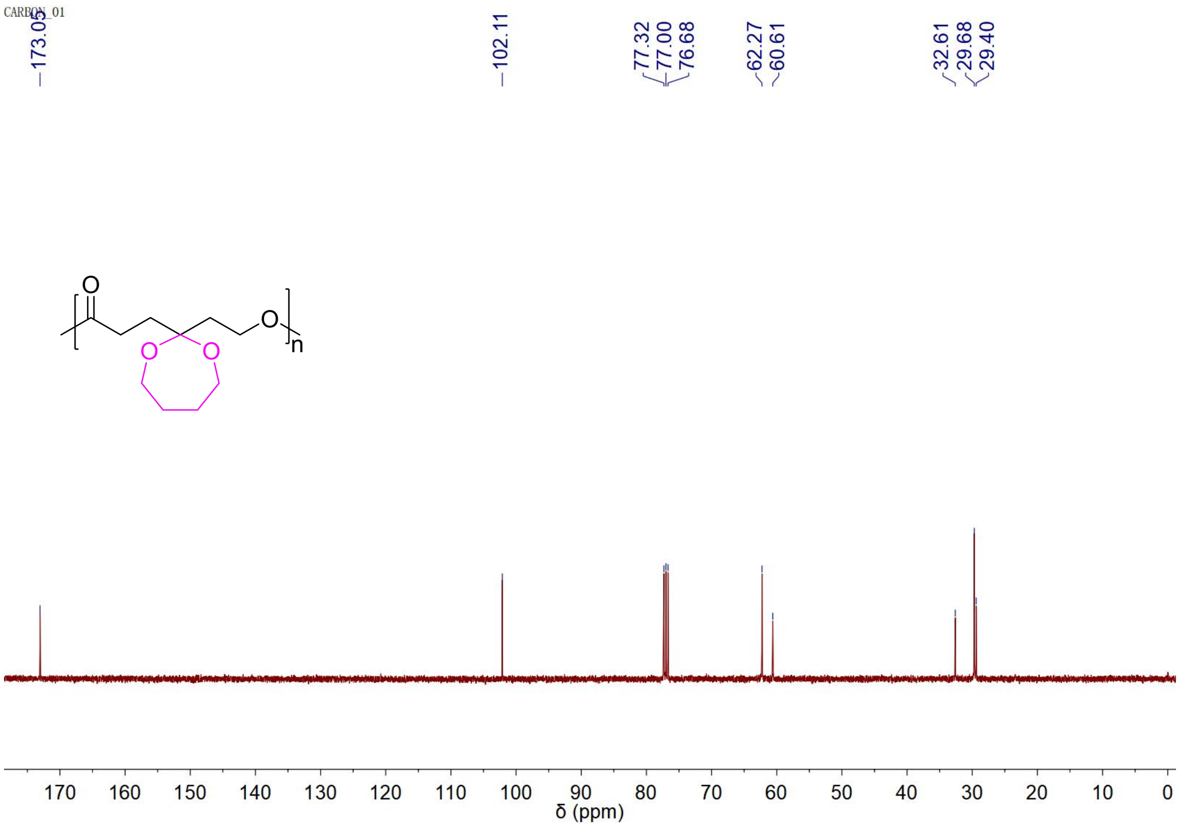

Supplementary Figure 52 ^{13}C NMR ($CDCl_3$, 25 °C) spectrum of P(**M12**) obtained by [**M12**]/[**Zn-1**]/[I] = 1000/1/1.

Characterizations of P(**M13**)

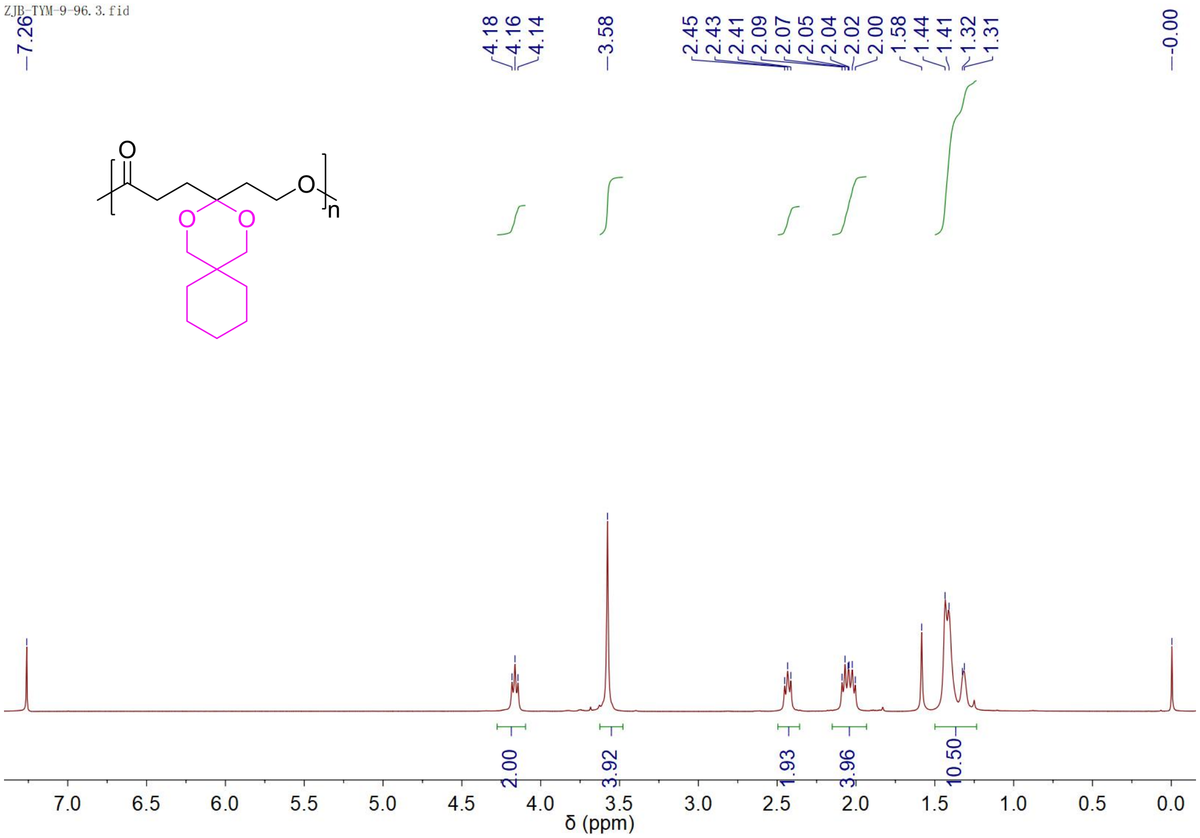

Supplementary Figure 53 ^{1}H NMR ($CDCl_3$, 25 °C) spectrum of P(**M13**) obtained by [**M13**]/[**Zn-1**]/[I] = 1000/1/1.

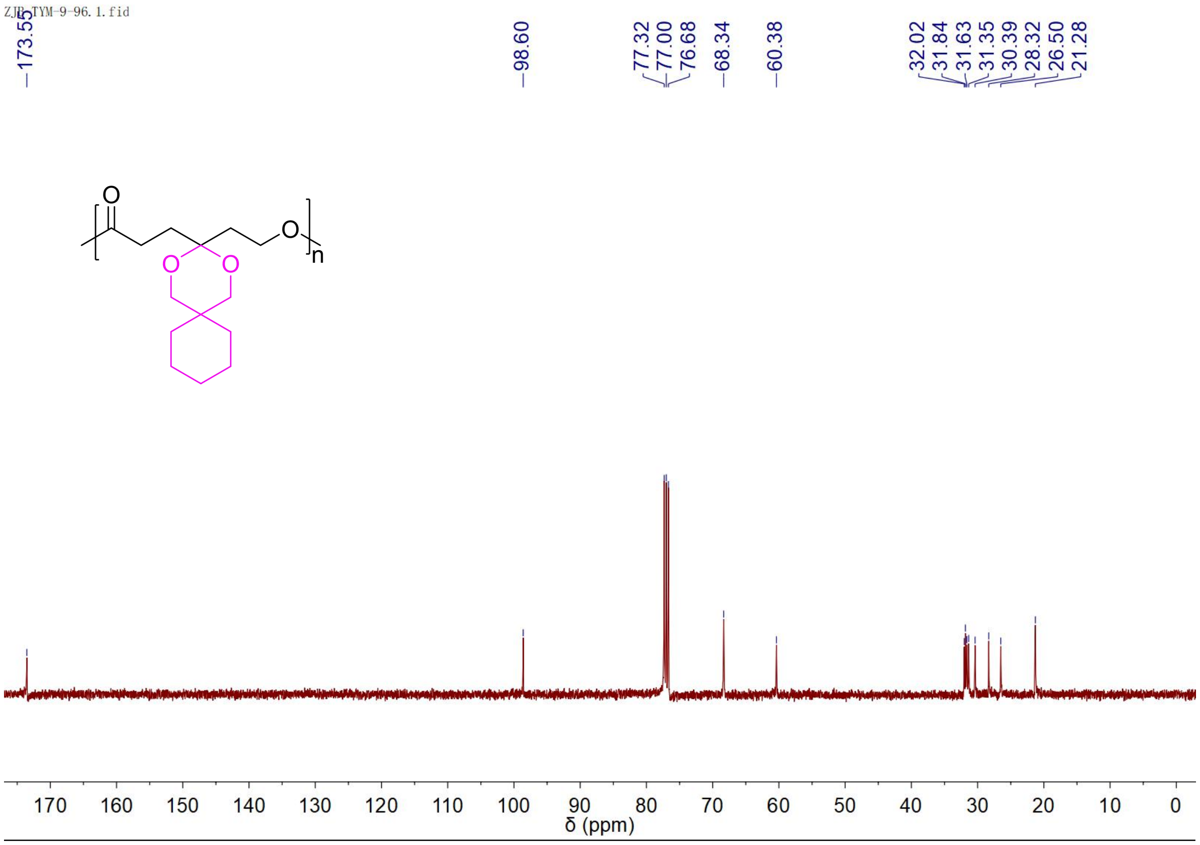

Supplementary Figure 54 ^{13}C NMR ($CDCl_3$, 25 °C) spectrum of P(**M13**) obtained by [**M13**]/[**Zn-1**]/[I] = 1000/1/1.

Characterizations of P(**M14**)

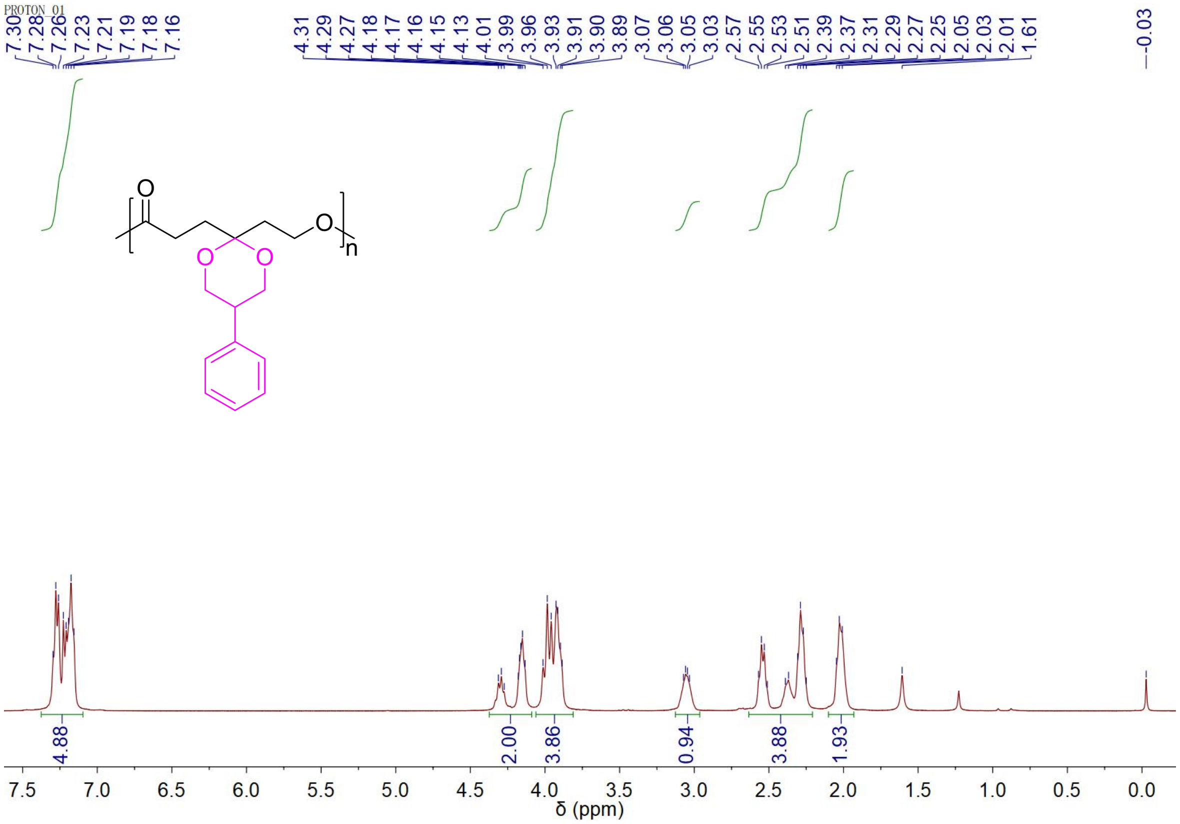

Supplementary Figure 55 ^{1}H NMR ($CDCl_3$, 25 °C) spectrum of P(**M14**) obtained by [**M14**]/[**Zn-1**]/[I] = 1000/1/1.

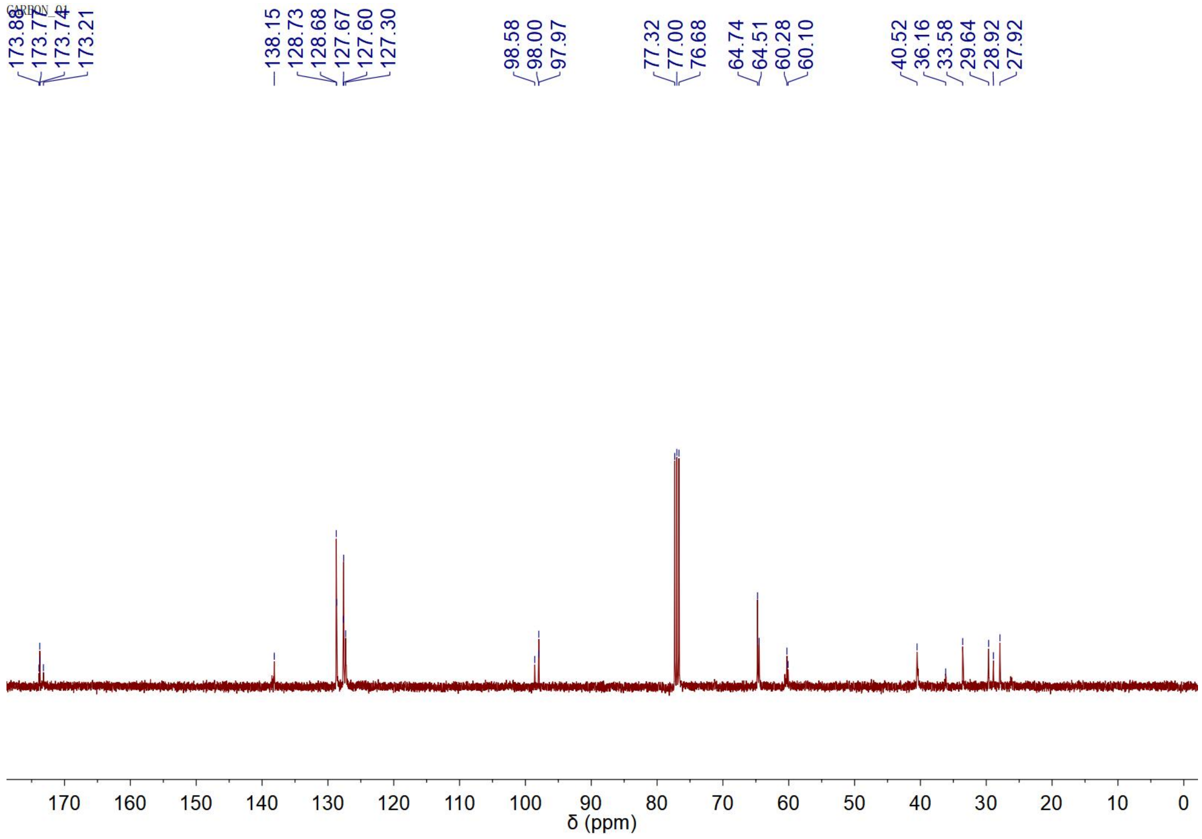

Supplementary Figure 56 ^{13}C NMR ($CDCl_3$, 25 °C) spectrum of P(**M14**) obtained by [**M14**]/[**Zn-1**]/[I] = 1000/1/1.

Characterizations of P(**M15**)

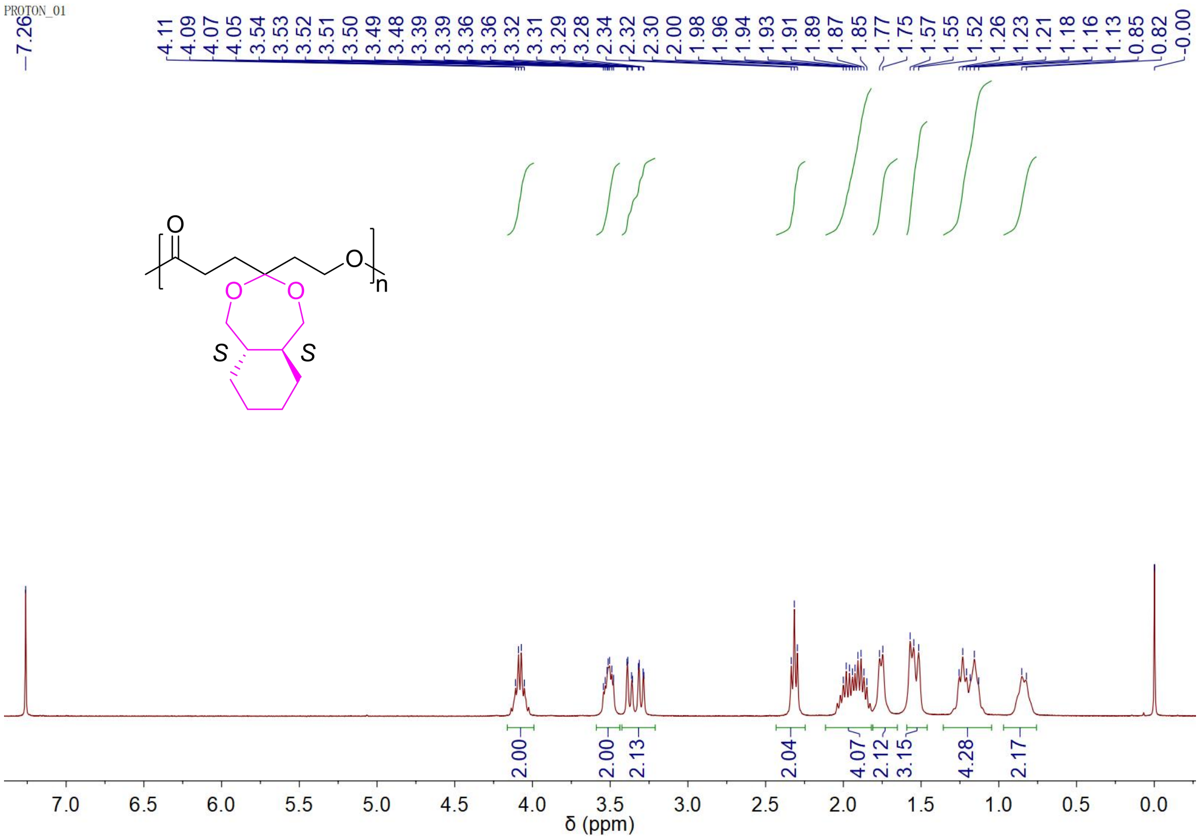

Supplementary Figure 57 ^{1}H NMR ($CDCl_3$, 25 °C) spectrum of P(**M15**) obtained by [**M15**]/[**Zn-1**]/[I] = 1000/1/1.

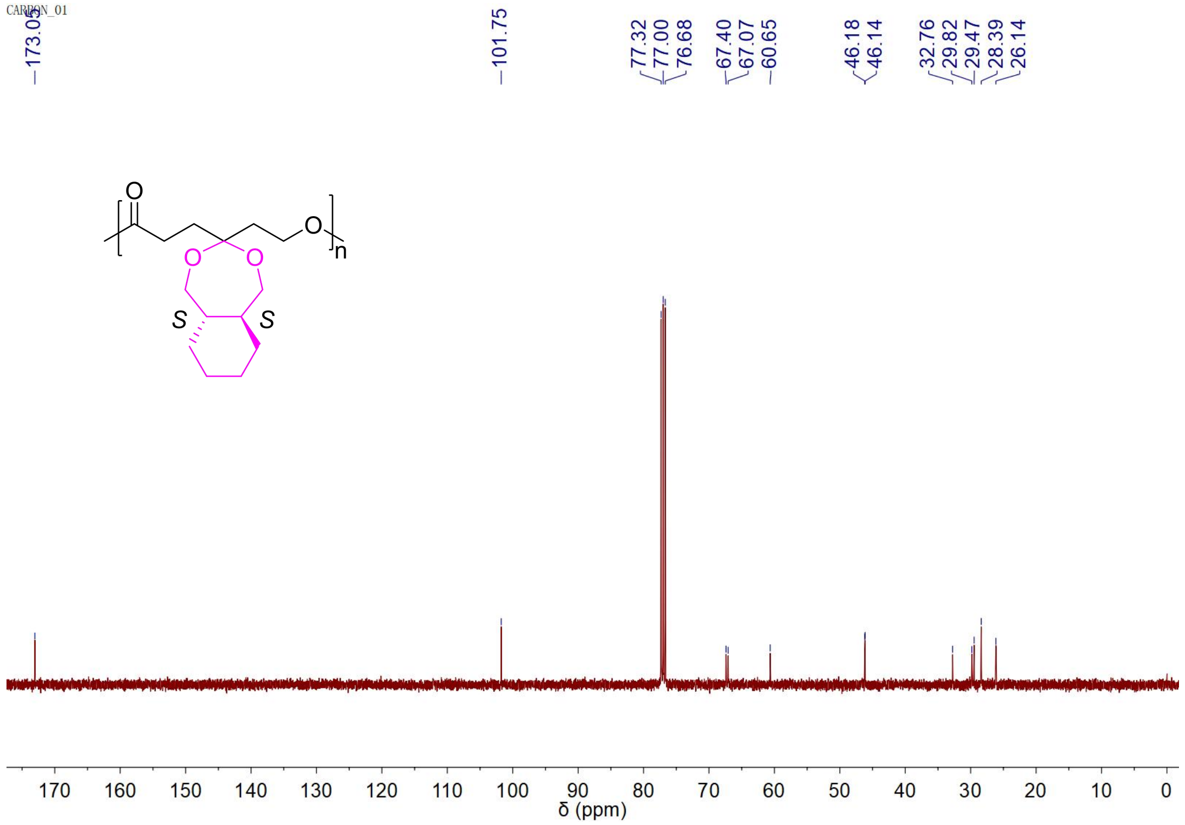

Supplementary Figure 58 ^{13}C NMR ($CDCl_3$, 25 °C) spectrum of P(**M15**) obtained by [**M15**]/[**Zn-1**]/[I] = 1000/1/1.

Characterizations of P(**M16**)

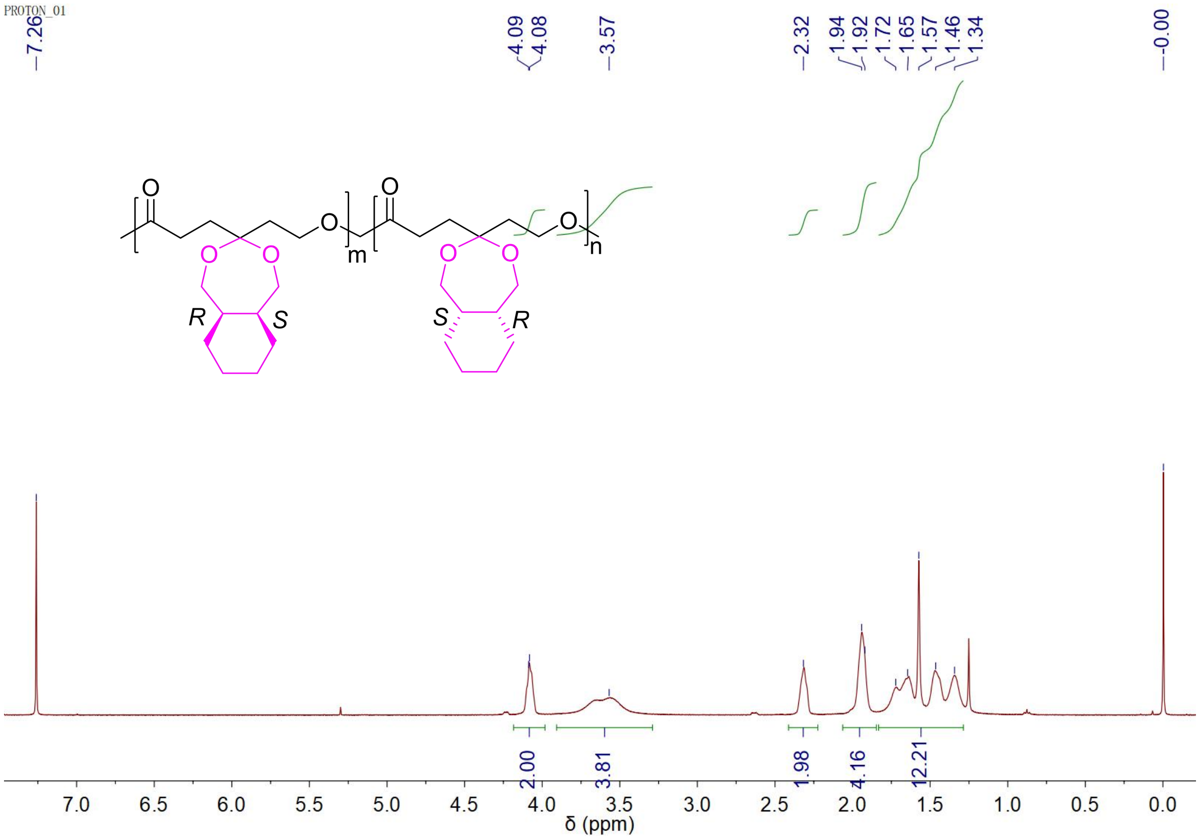

Supplementary Figure 59 ^{1}H NMR ($CDCl_3$, 25 °C) spectrum of P(**M16**) obtained by [**M16**]/[**Zn-1**]/[I] = 1000/1/1.

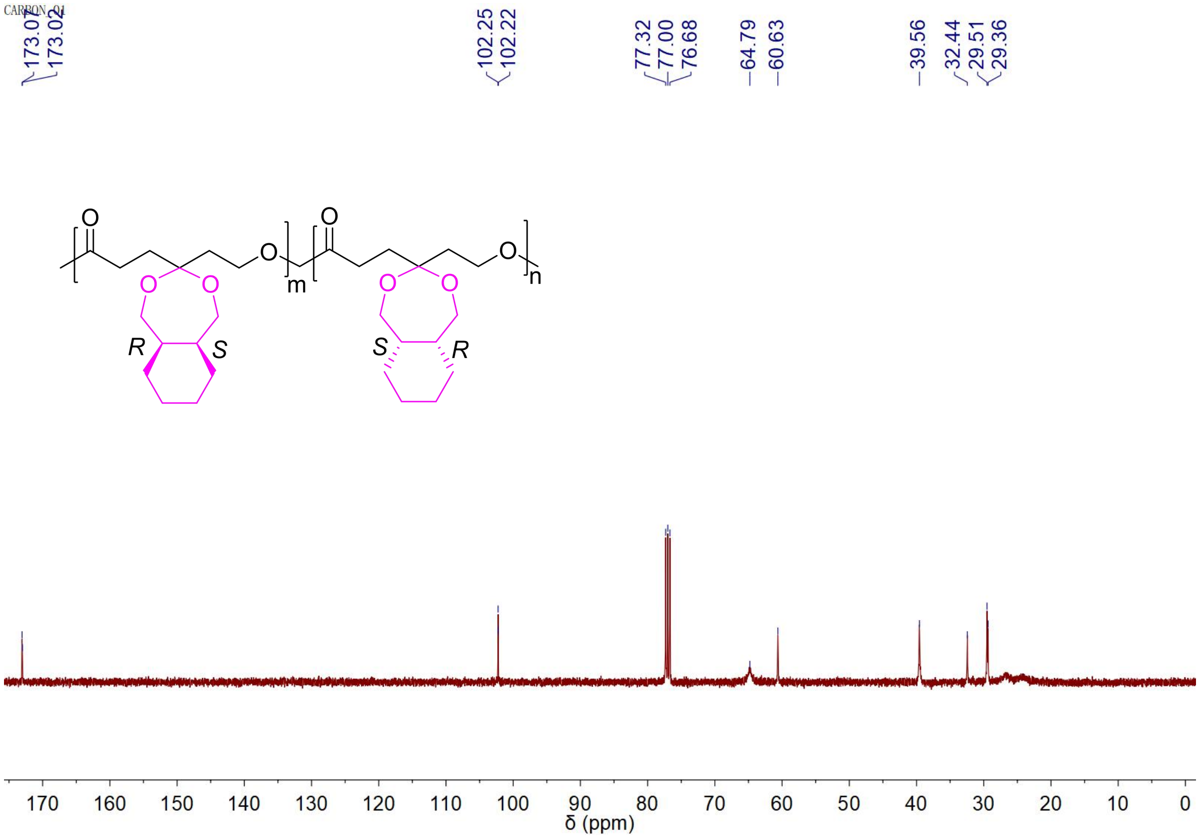

Supplementary Figure 60 ^{13}C NMR ($CDCl_3$, 25 °C) spectrum of P(**M16**) obtained by [**M16**]/[**Zn-1**]/[I] = 1000/1/1.

Characterizations of P(**M17**)

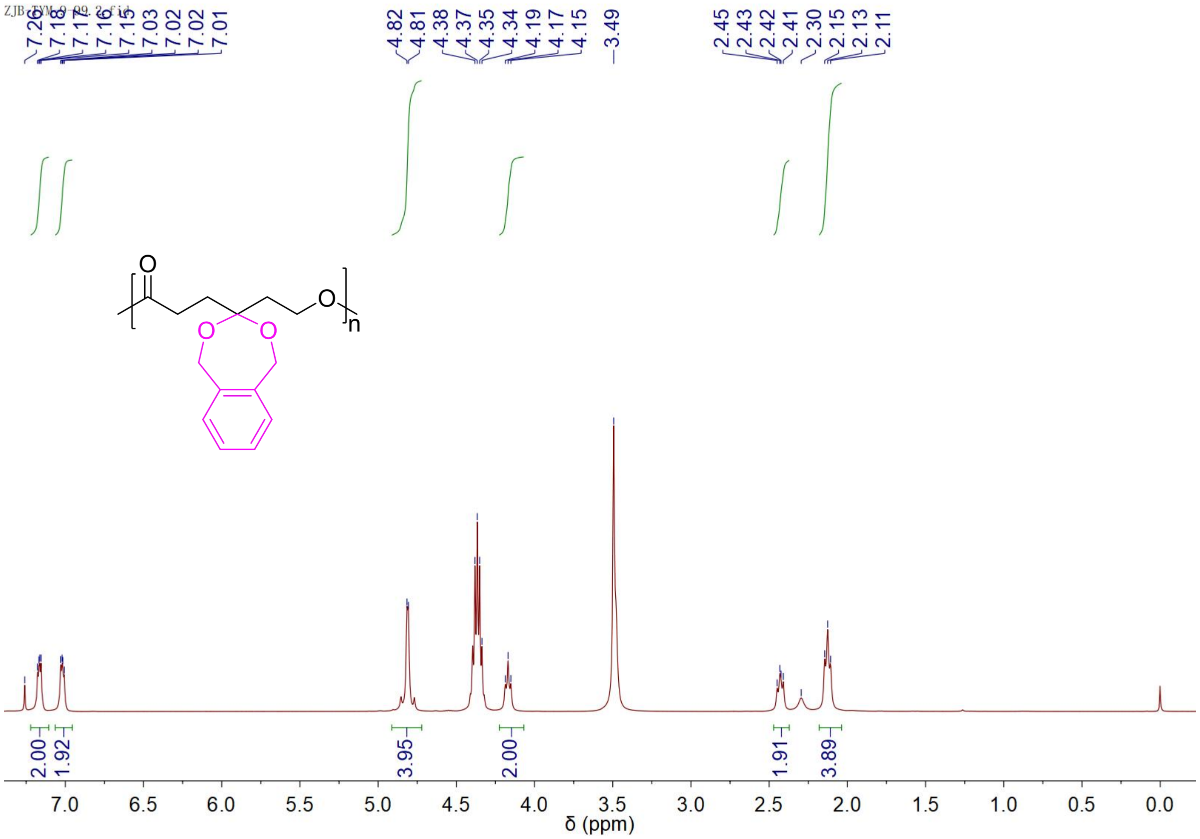

Supplementary Figure 61 ^{1}H NMR ($CDCl_3$:HFIP = 4:1, 25 °C)[12] spectrum of P(**M17**) obtained by [**M17**]/[**Zn-1**]/[I] = 1000/1/1.

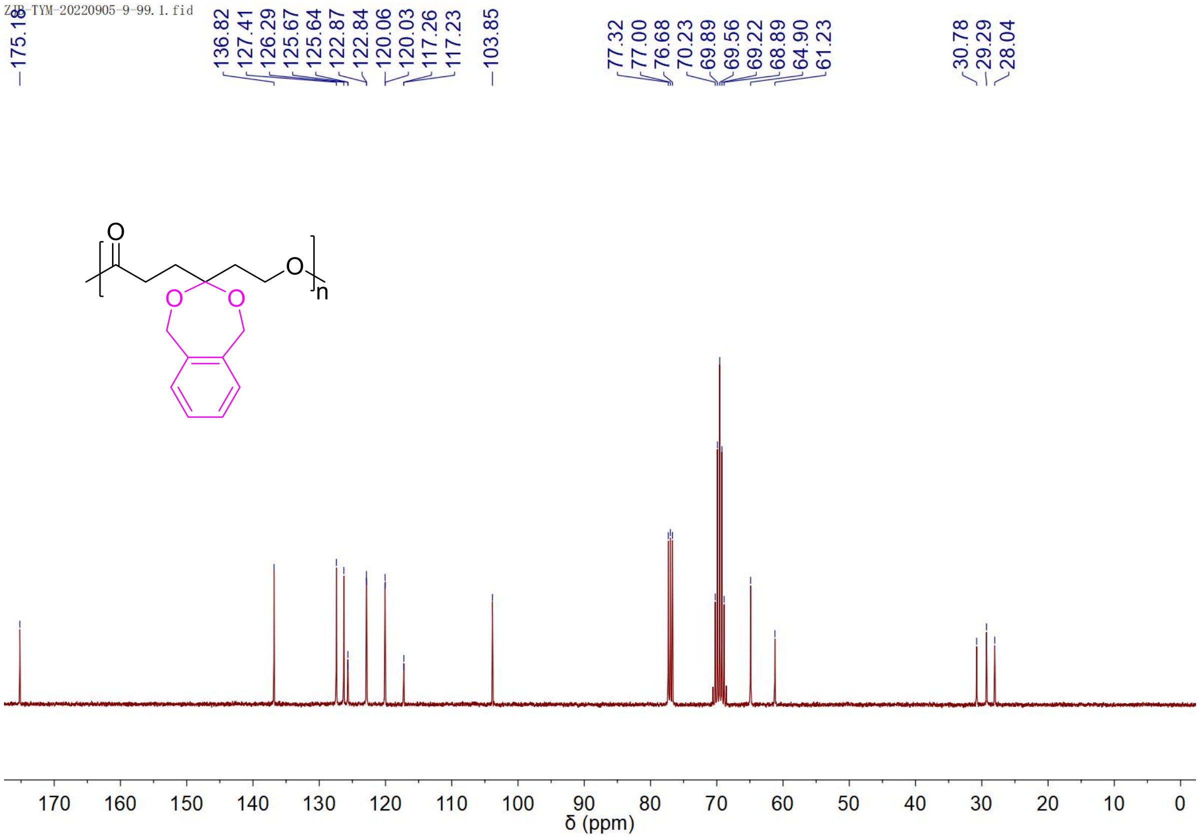

Supplementary Figure 62 ^{13}C NMR ($CDCl_3$: HFIP = 4:1[12], 25 °C) spectrum of P(**M17**) obtained by [**M17**]/[**Zn-1**]/[I] = 1000/1/1.

MALDI-TOF Spectra of Polymer

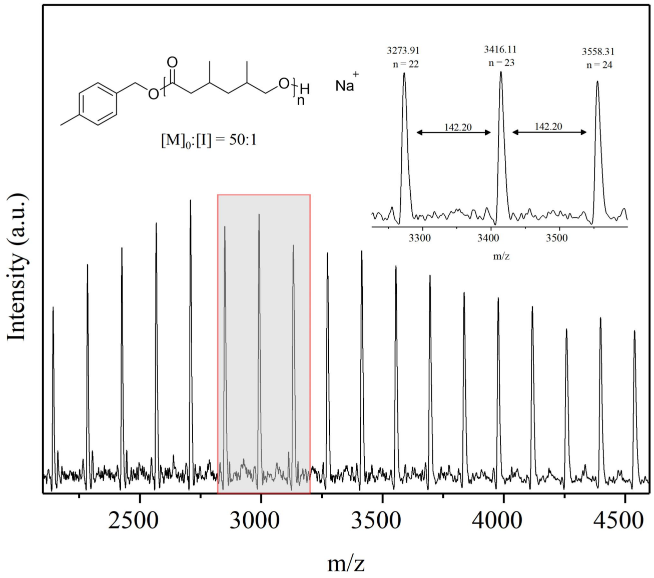

Supplementary Figure 63 MALDI-TOF MS spectrum of P(**M6)** produced by [**M6**]/[**Zn-1**]/[I] = 50/1/1 in 1 M at RT.

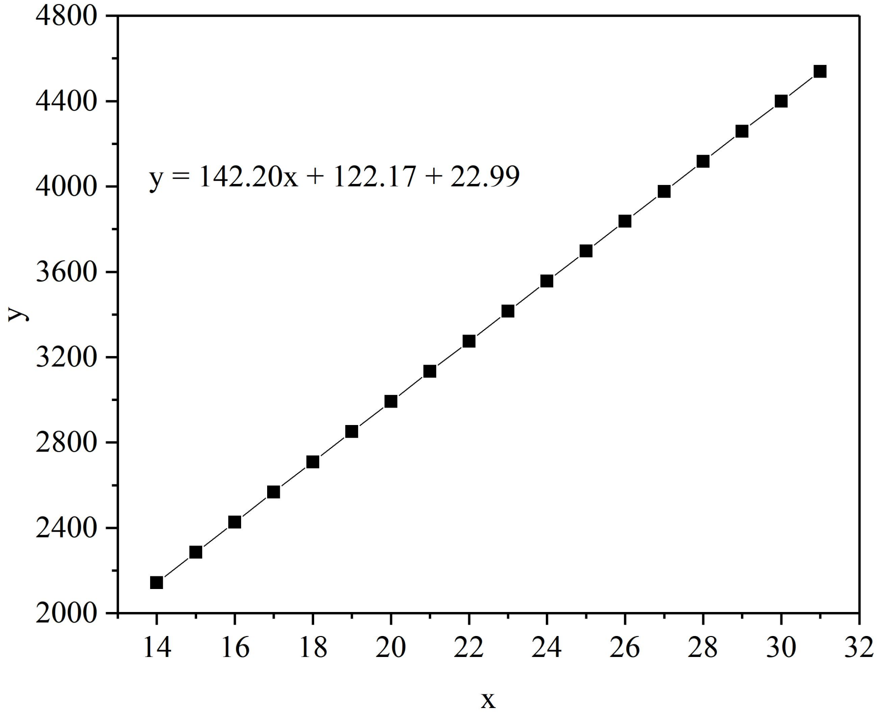

Supplementary Figure 64 Linear plot of m/z values (y) *vs* the number of **M6** repeat units (x).

Thermal Properties of Polymers

TGA and DTG Characterizations of P(**M1**)

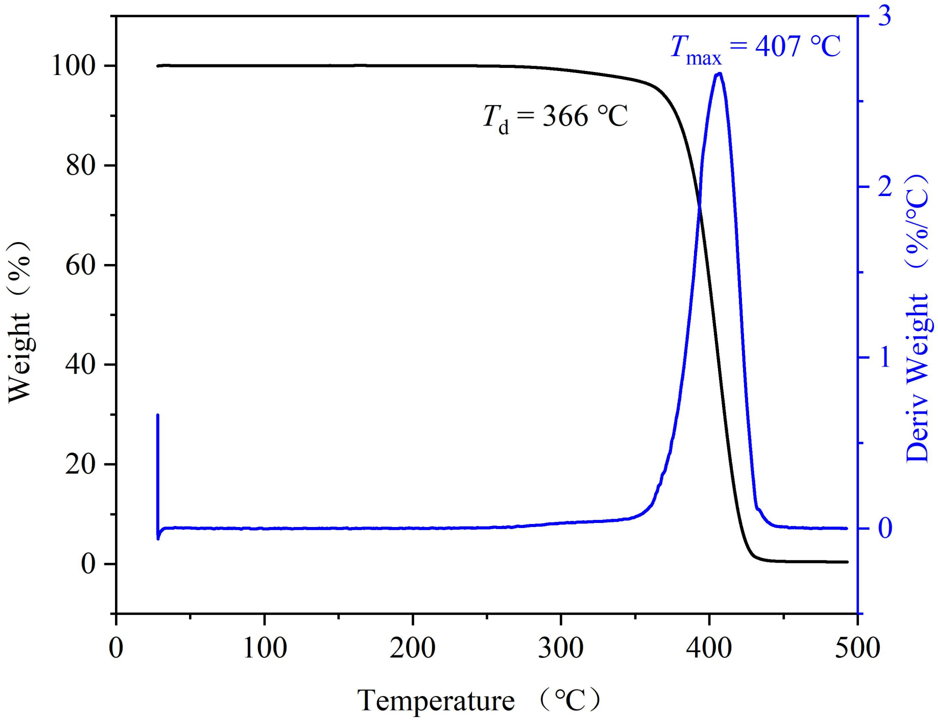

Supplementary Figure 65 TGA and DTG curves for P(**M1**), T_d = 366 °C, T_{max} = 407 °C.

TGA and DTG Characterizations of P(**M2**)

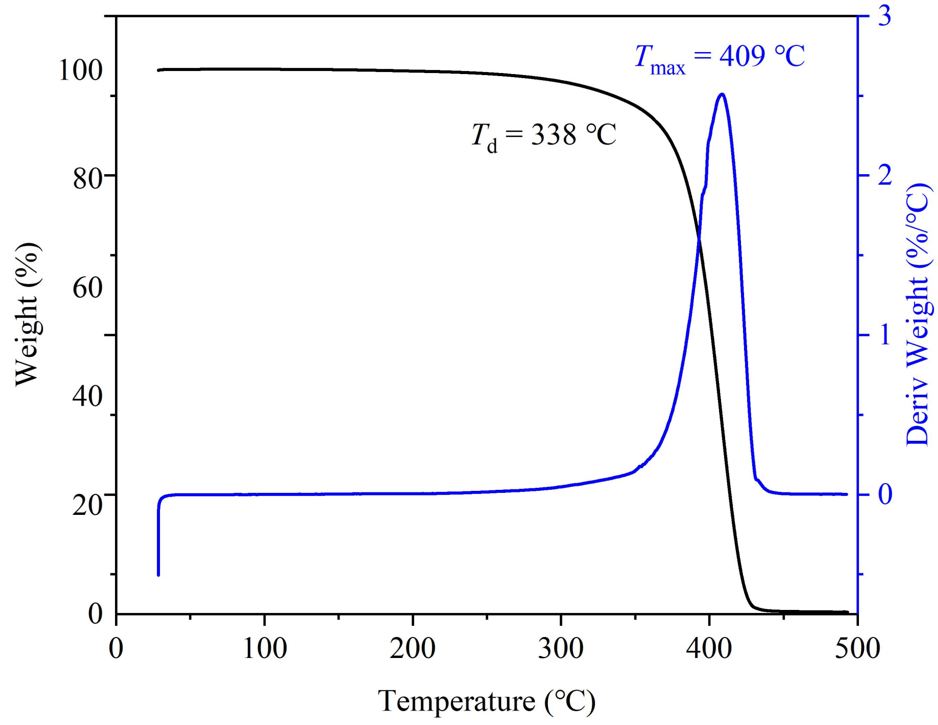

Supplementary Figure 66 TGA and DTG curves for P(**M2**), T_d = 338 °C, T_{max} = 409 °C.

TGA and DTG Characterizations of P(**M3**)

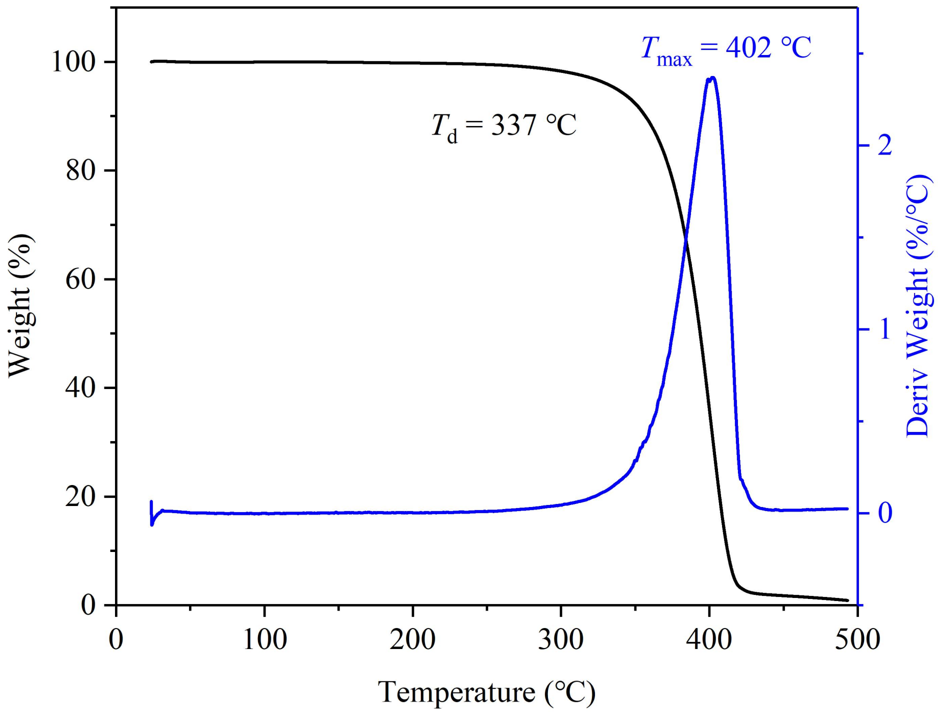

Supplementary Figure 67 TGA and DTG curves for P(**M3**), T_d = 337 °C, T_{max} = 402 °C.

TGA and DTG Characterizations of P(**M4**)

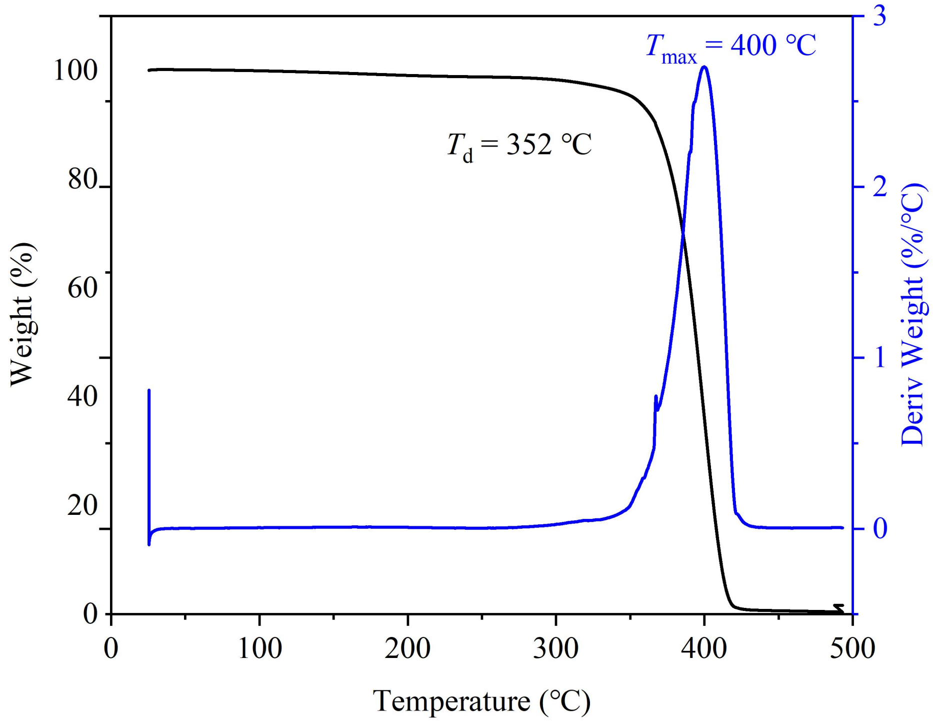

Supplementary Figure 68 TGA and DTG curves for P(**M4**), T_d = 352 °C, T_{max} = 400 °C.

TGA and DTG Characterizations of P(**M5**)

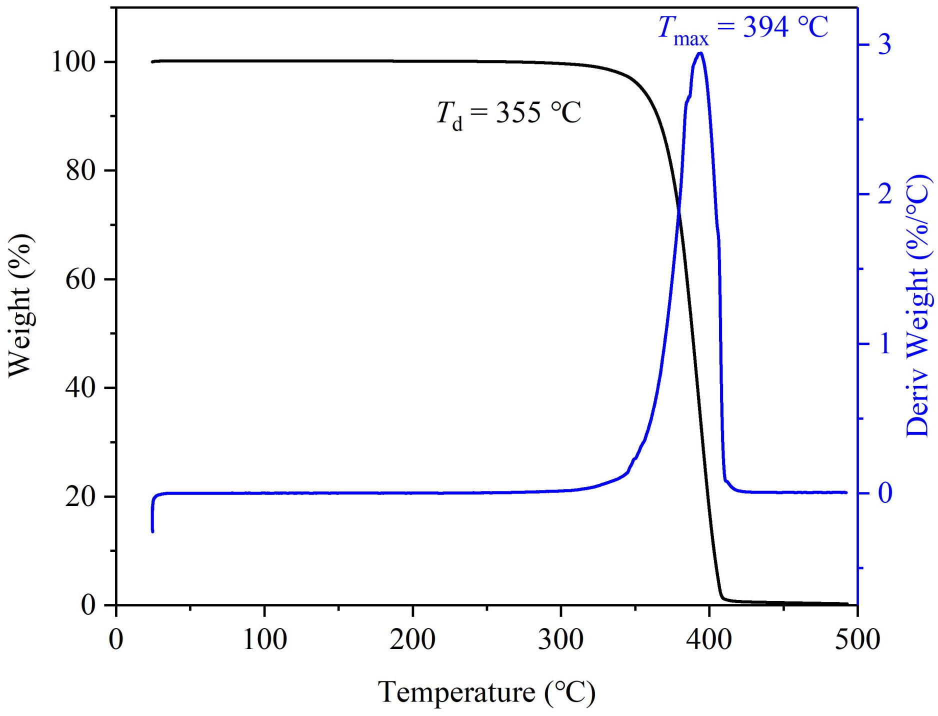

Supplementary Figure 69 TGA and DTG curves for P(**M5**), T_d = 355 °C, T_{max} = 394 °C.

TGA and DTG Characterizations of P(**M6**)

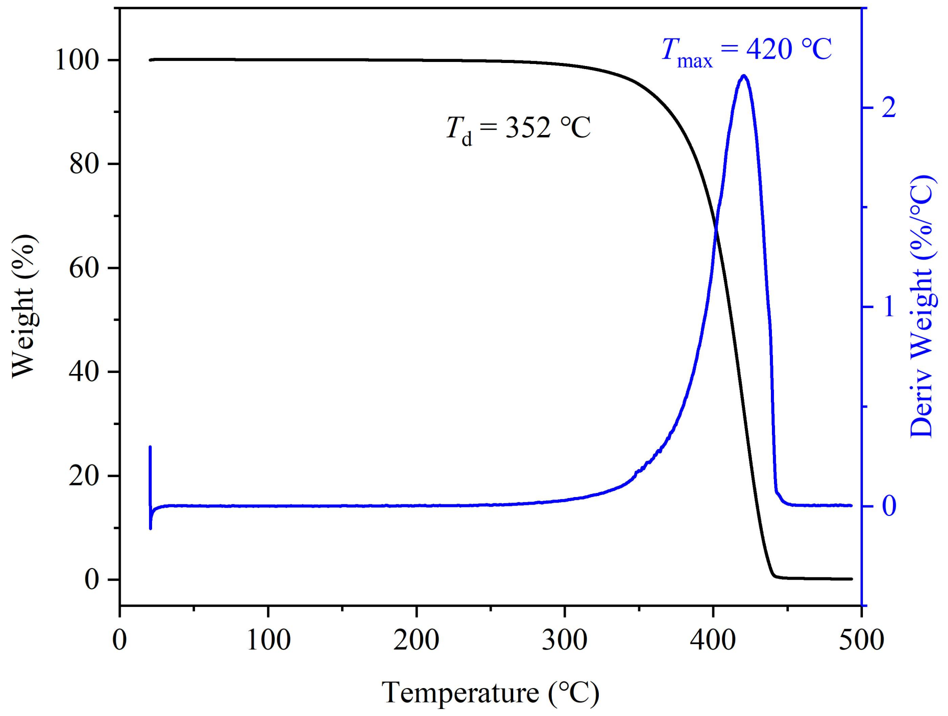

Supplementary Figure 70 TGA and DTG curves for P(**M6**), T_d = 352 °C, T_{max} = 420 °C.

TGA and DTG Characterizations of P(**M8**)

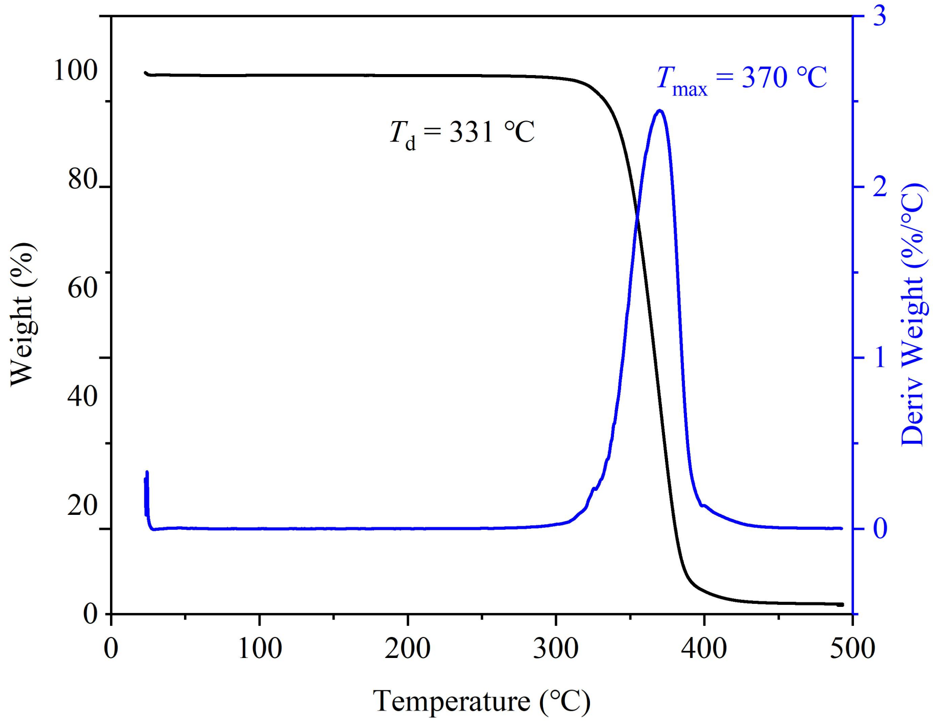

Supplementary Figure 71 TGA and DTG curves for P(**M8**), T_d = 331 °C, T_{max} =370 °C.

TGA and DTG Characterizations of P(**M9**)

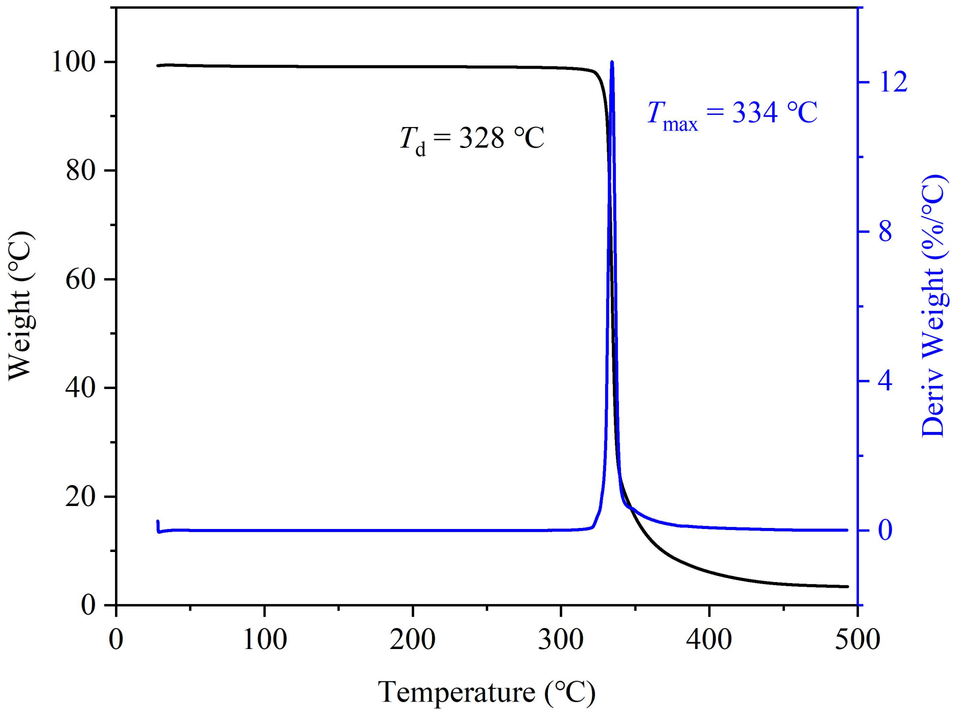

Supplementary Figure 72 TGA and DTG curves for P(**M9**), T_d = 328 °C, T_{max} = 334 °C.

TGA and DTG Characterizations of P(**M10**)

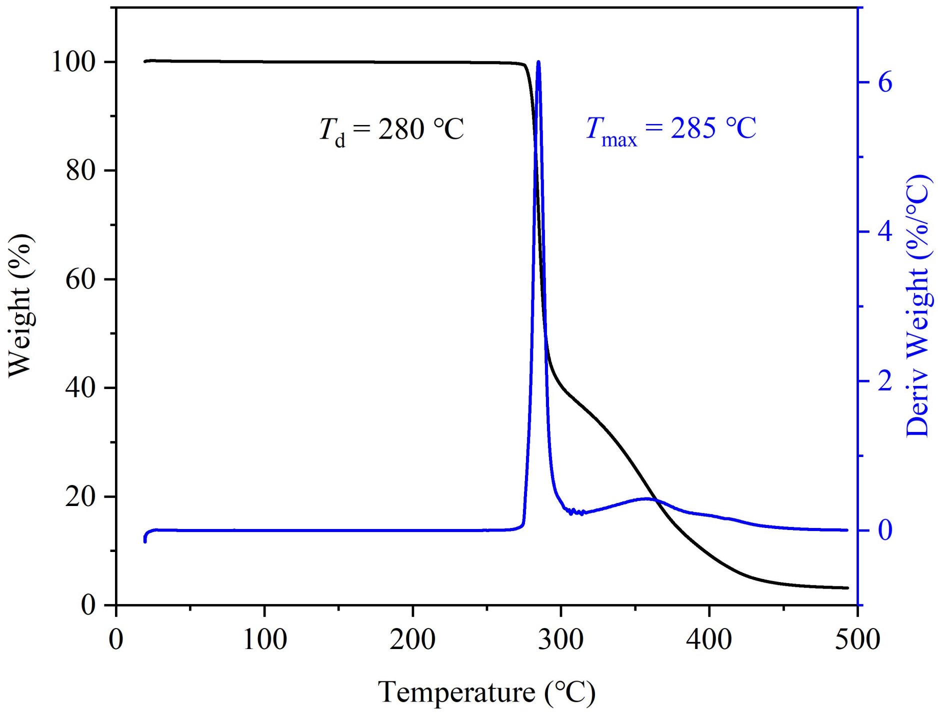

Supplementary Figure 73 TGA and DTG curves for P(**M10**), T_d = 280 °C, T_{max} = 285 °C.

TGA and DTG Characterizations of P(**M11**)

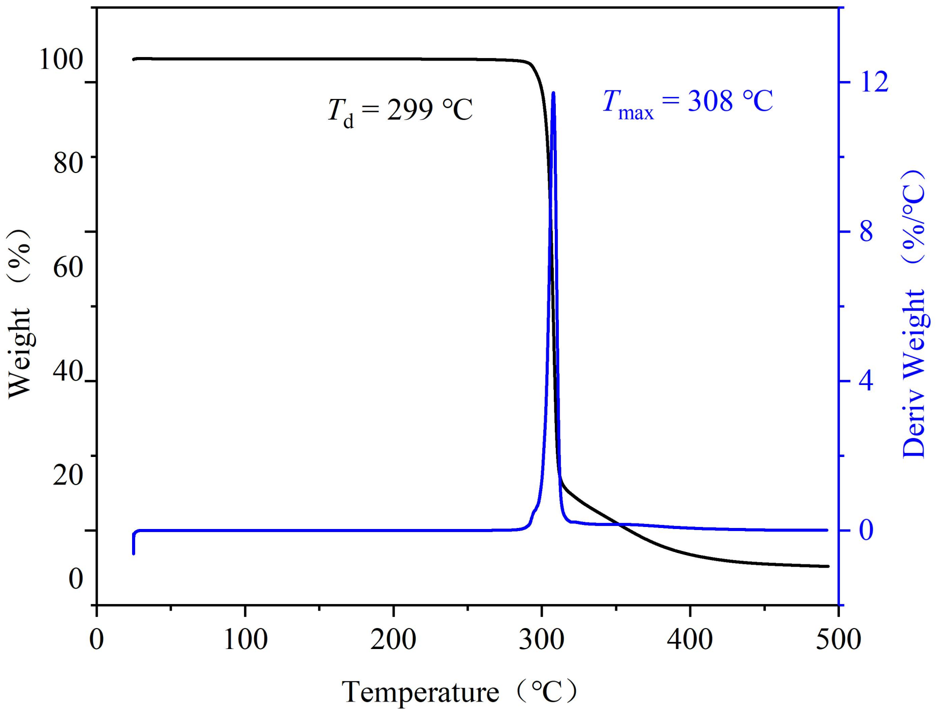

Supplementary Figure 74 TGA and DTG curves for P(**M11**), T_d = 299 °C, T_{max} = 308 °C.

TGA and DTG Characterizations of P(**M12**)

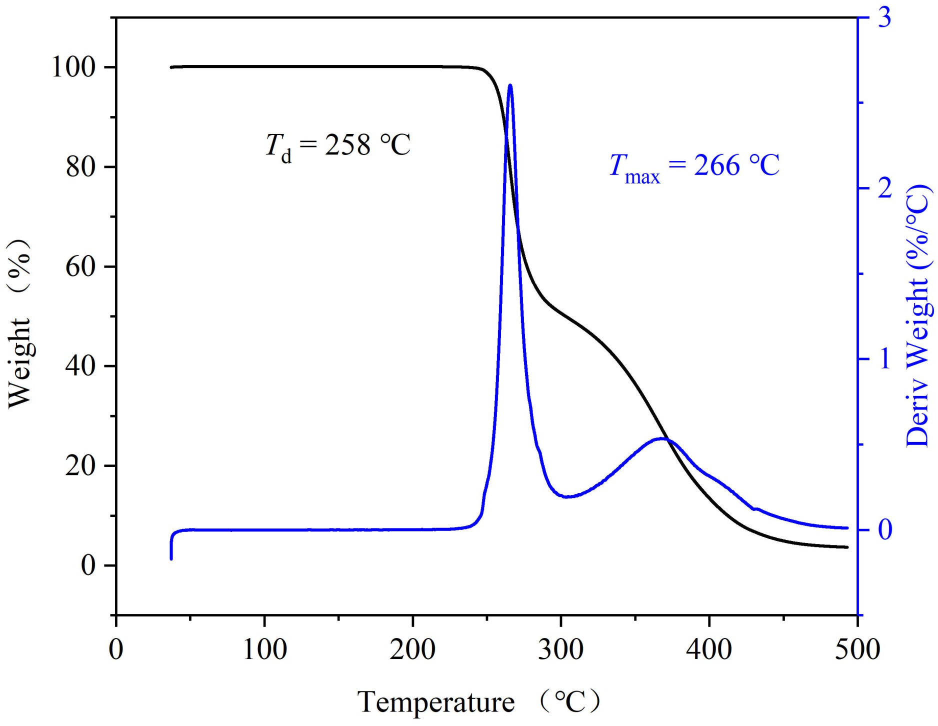

Supplementary Figure 75 TGA and DTG curves for P(**M12**), T_d = 258 °C, T_{max} = 266 °C.

TGA and DTG Characterizations of P(**M13**)

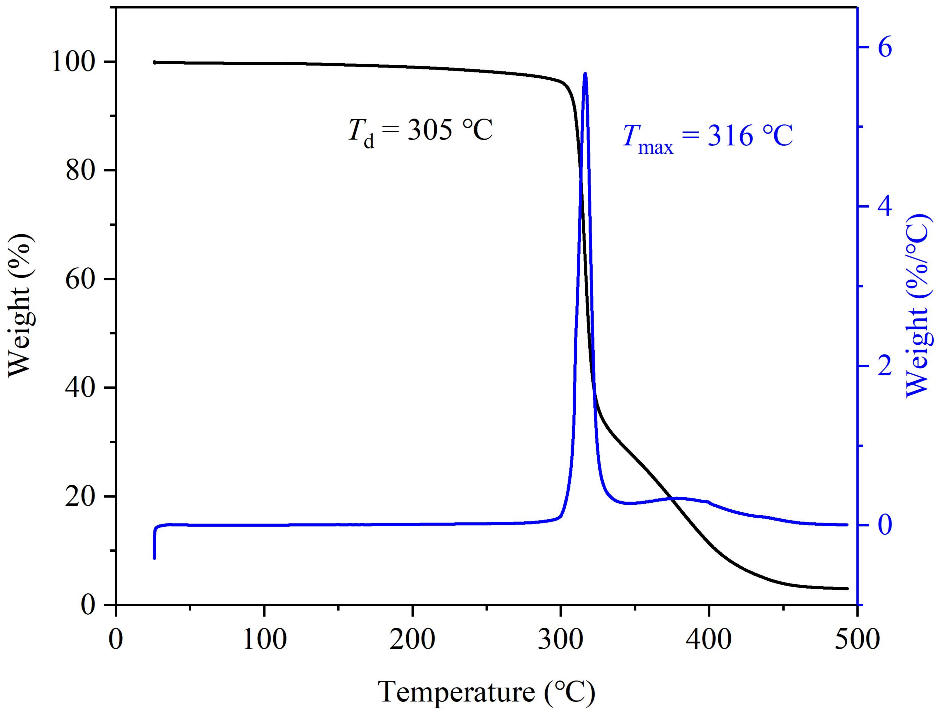

Supplementary Figure 76 TGA and DTG curves for P(**M13**), T_d = 305 °C, T_{max} =316 °C.

TGA and DTG Characterizations of P(**M14**)

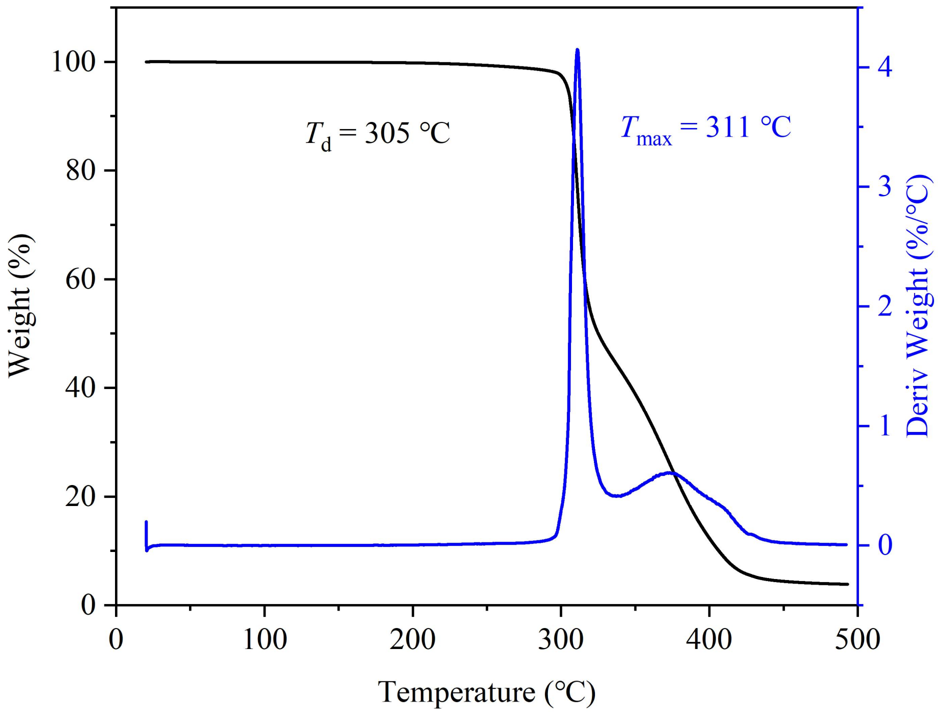

Supplementary Figure 77 TGA and DTG curves for P(**M14**), T_d = 305 °C, T_{max} = 311 °C.

TGA and DTG Characterizations of P(**M15**)

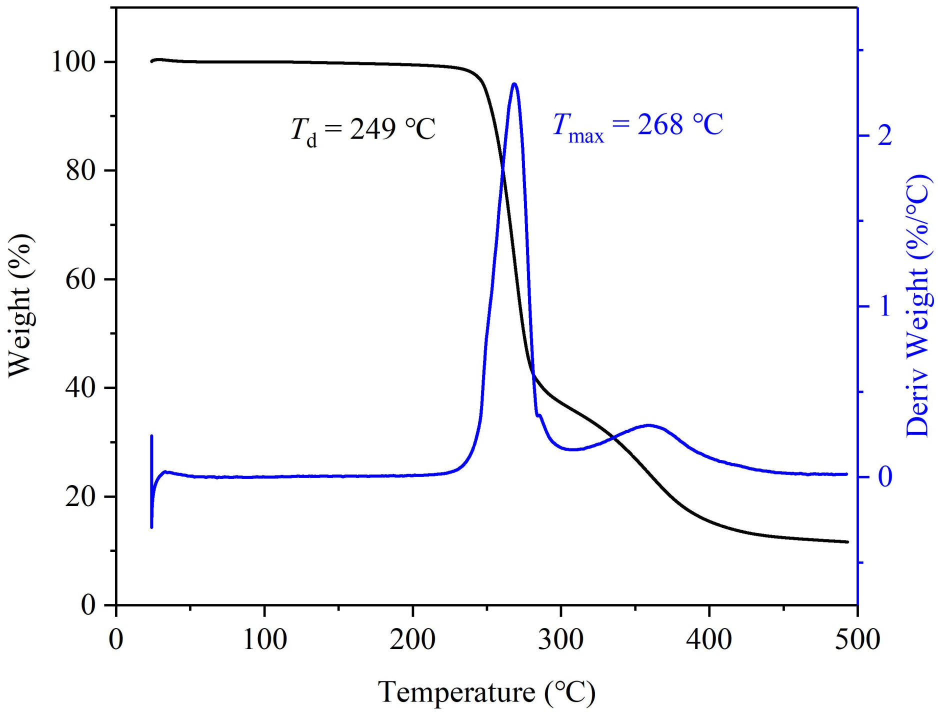

Supplementary Figure 78 TGA and DTG curves for P(**M15**), T_d = 249 °C, T_{max} =268 °C.

TGA and DTG Characterizations of P(**M16**)

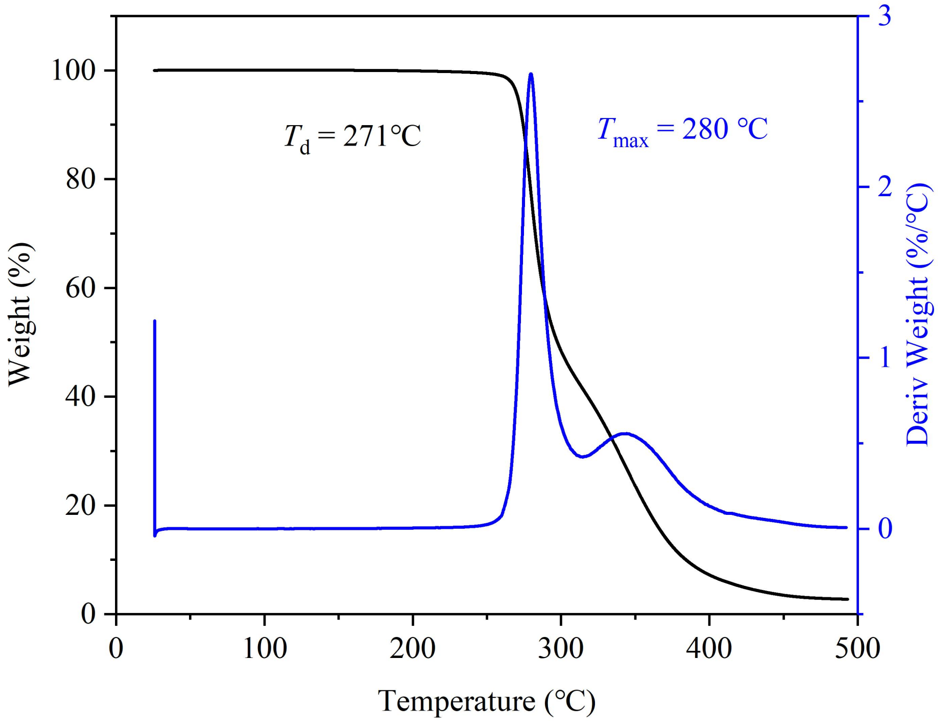

Supplementary Figure 79 TGA and DTG curves for P(**M16**), T_d = 271 °C, T_{max} = 280 °C.

TGA and DTG Characterizations of P(**M17**)

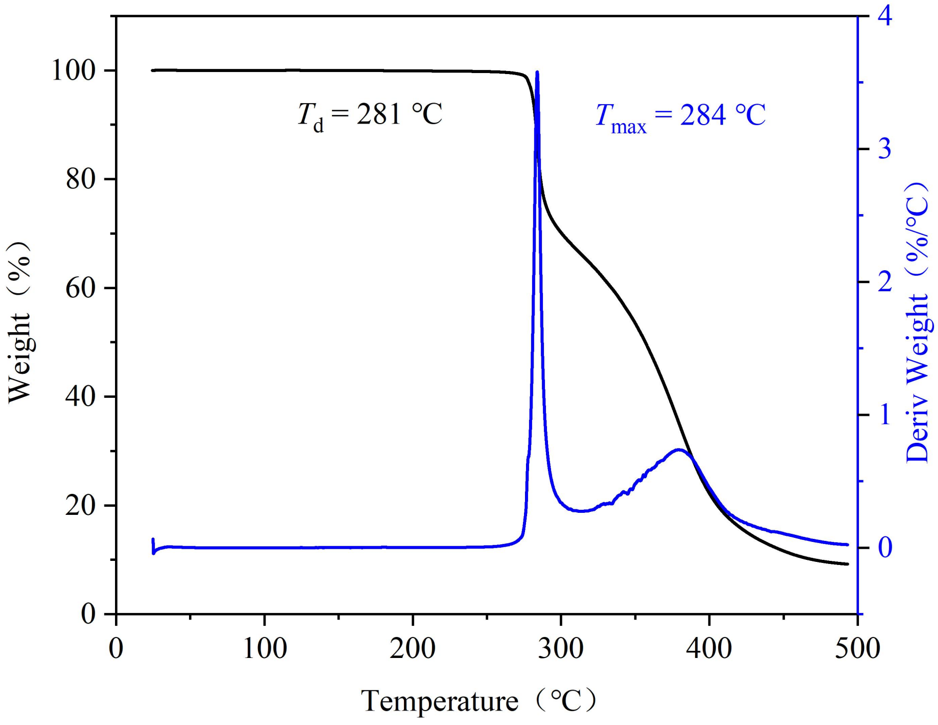

Supplementary Figure 80 TGA and DTG curves for P(**M17**), T_d = 281 °C, T_{max} = 284 °C.

Differential Scanning Calorimetry

DSC Characterizations of P(**M1**)

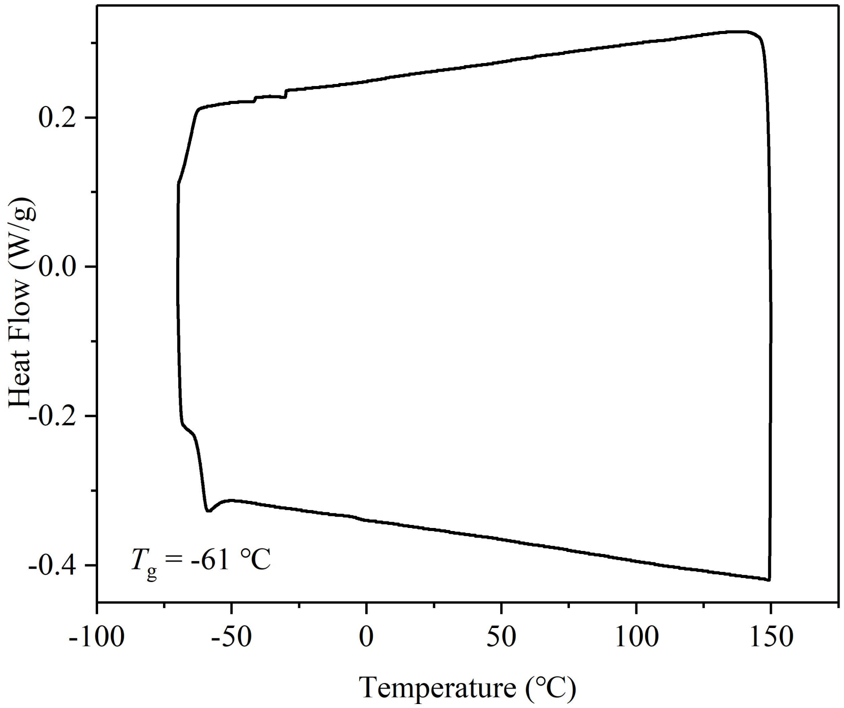

Supplementary Figure 81 DSC curves for P(**M1**), T_g = –61 °C.

DSC Characterizations of P(**M2**)

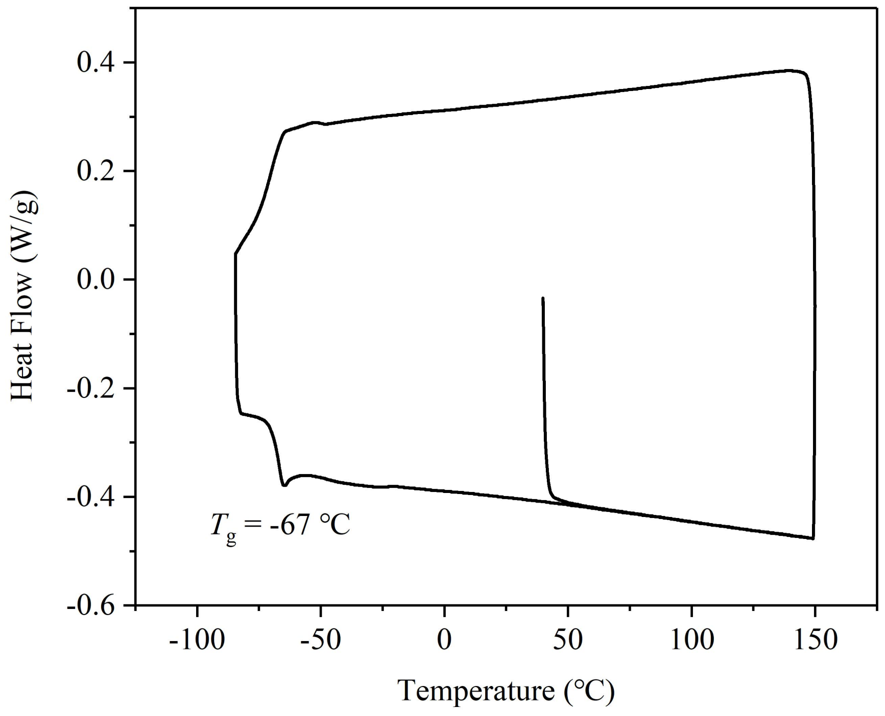

Supplementary Figure 82 DSC curves for P(**M2**), T_g = –67 °C.

DSC Characterizations of P(**M3**)

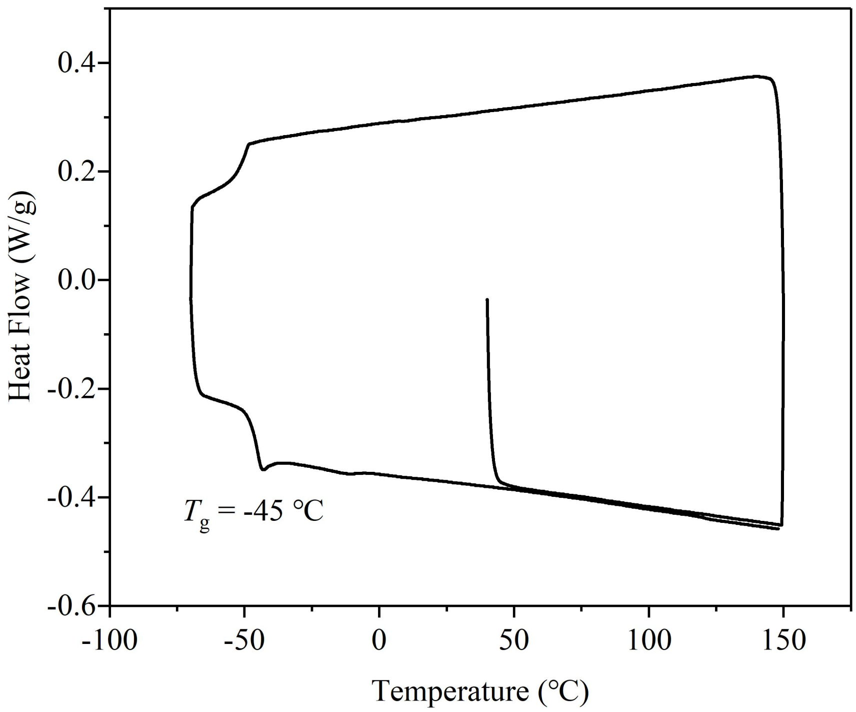

Supplementary Figure 83 DSC curves for P(**M3**), T_g = –45 °C.

DSC Characterizations of P(**M4**)

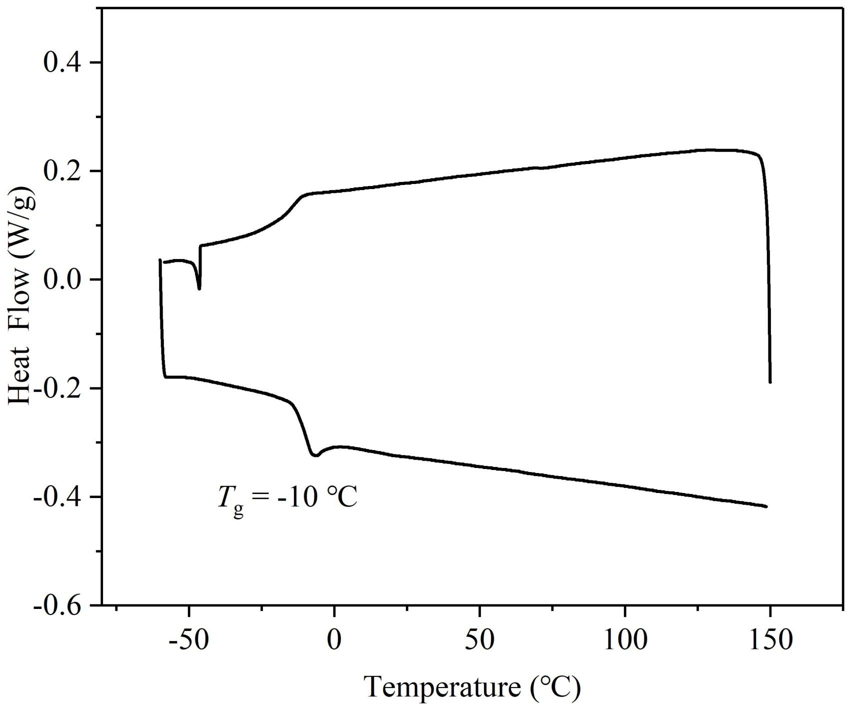

Supplementary Figure 84 DSC curves for P(**M4**), T_g = –10 °C.

DSC Characterizations of P(**M5**)

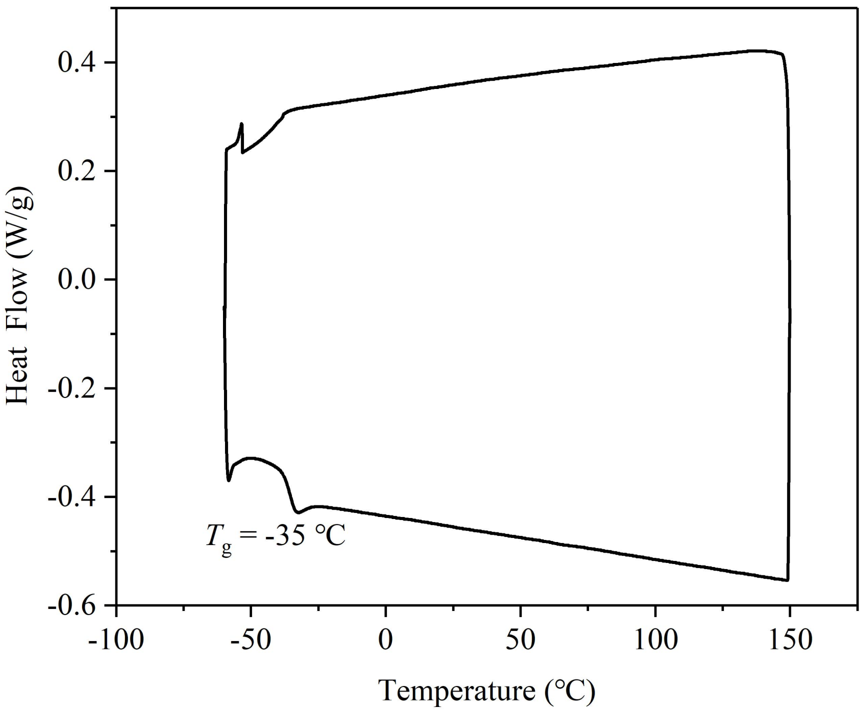

Supplementary Figure 85 DSC curves for P(**M5**), T_g = –35 °C.

DSC Characterizations of P(**M6**)

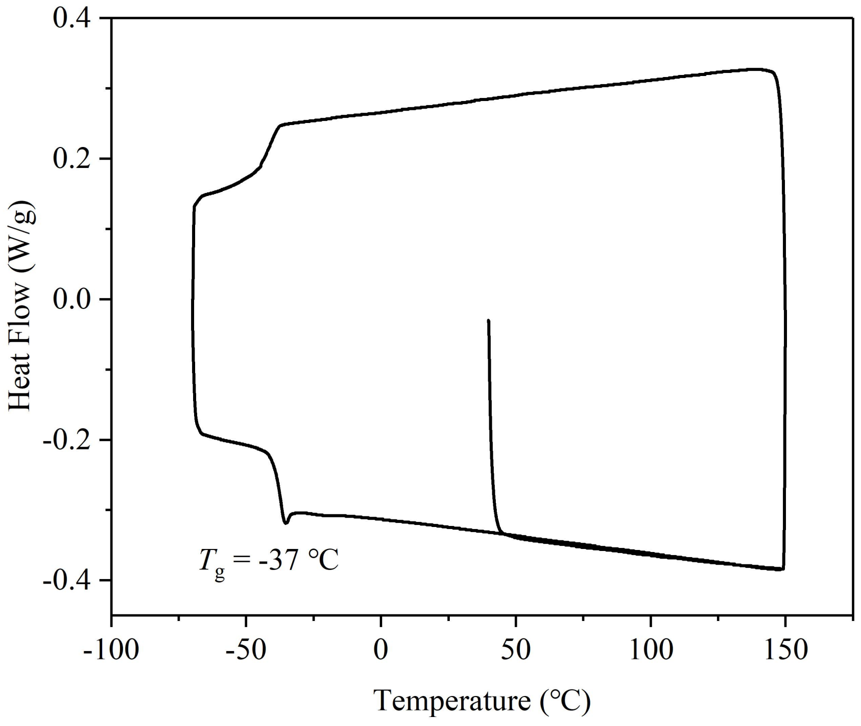

Supplementary Figure 86 DSC curves for P(**M6**), T_g = –37 °C.

DSC Characterizations of P(**M8**)

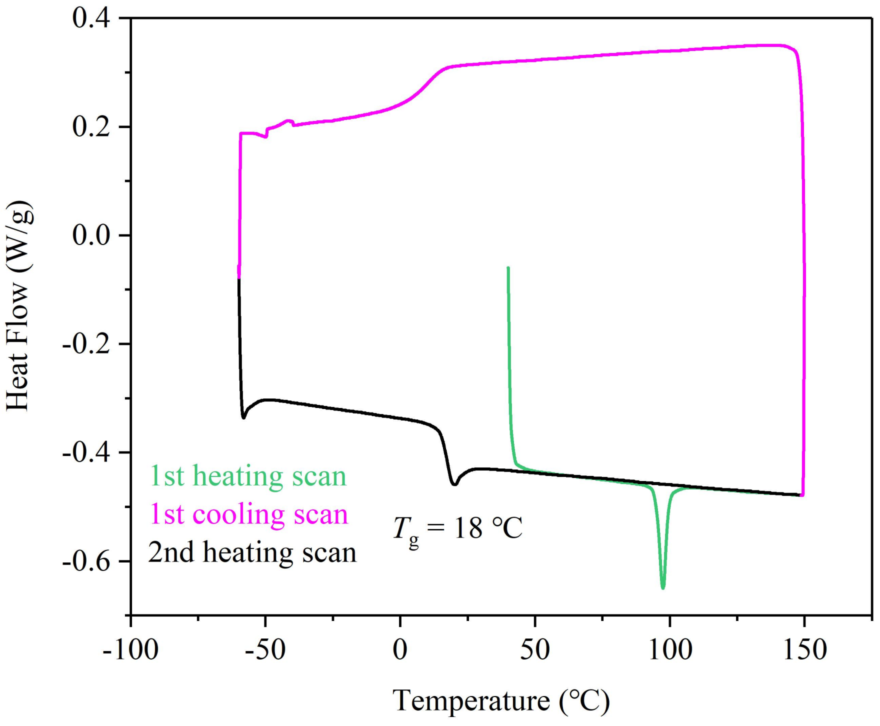

Supplementary Figure 87 DSC curves for P(**M8**), T_g = 18 °C.

DSC Characterizations of P(**M9**)

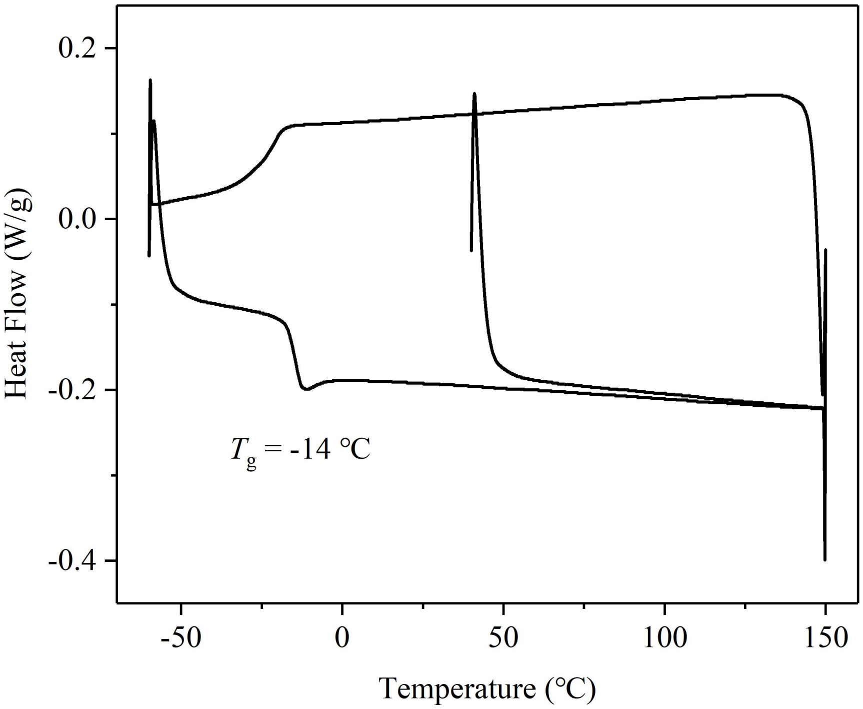

Supplementary Figure 88 DSC curves for P(**M9**), T_g = –14 °C.

DSC Characterizations of P(**M10**)

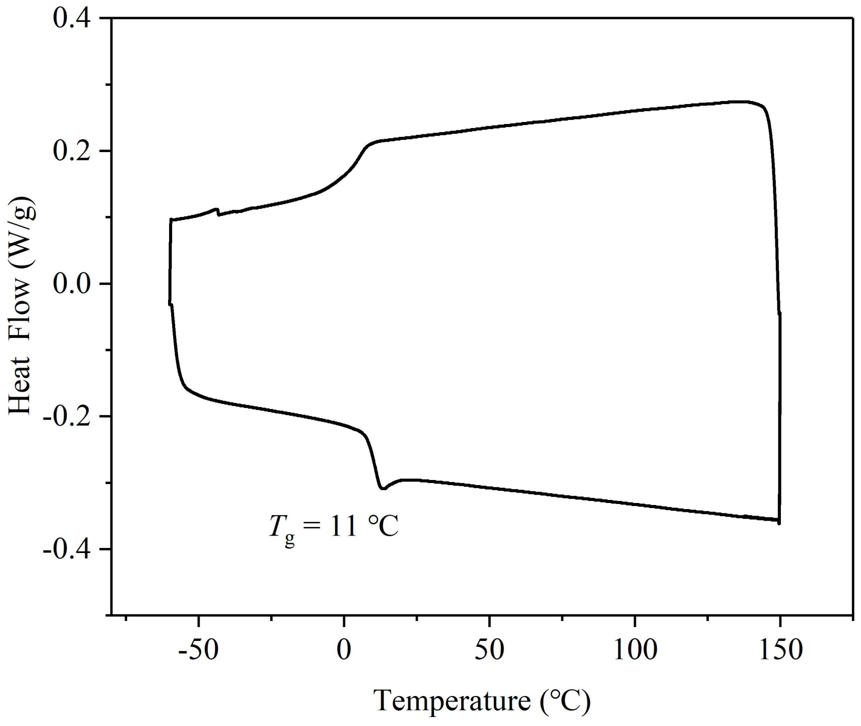

Supplementary Figure 89 DSC curves for P(**M10**), T_g = 11 °C.

DSC Characterizations of P(**M11**)

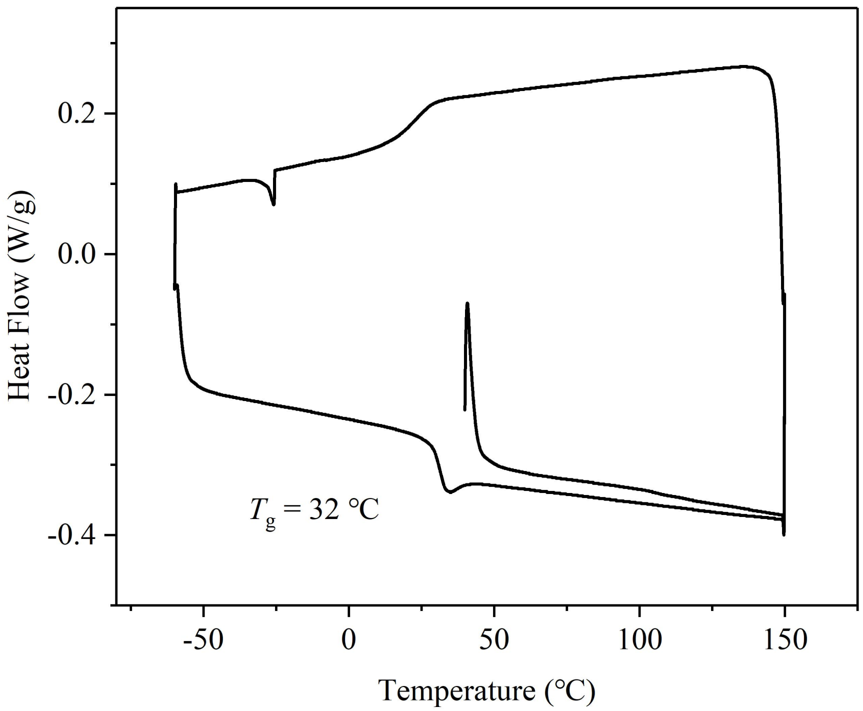

Supplementary Figure 90 DSC curves for P(**M11**), T_g = 32 °C.

DSC Characterizations of P(**M12**)

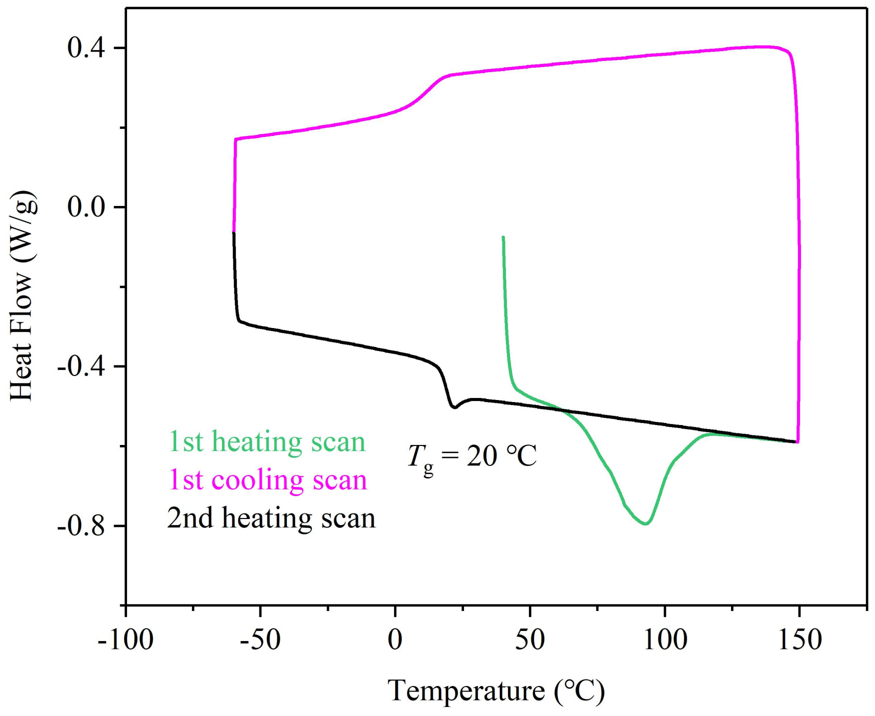

Supplementary Figure 91 DSC curves for P(**M12**), T_g = 20 °C.

DSC Characterizations of P(**M13**)

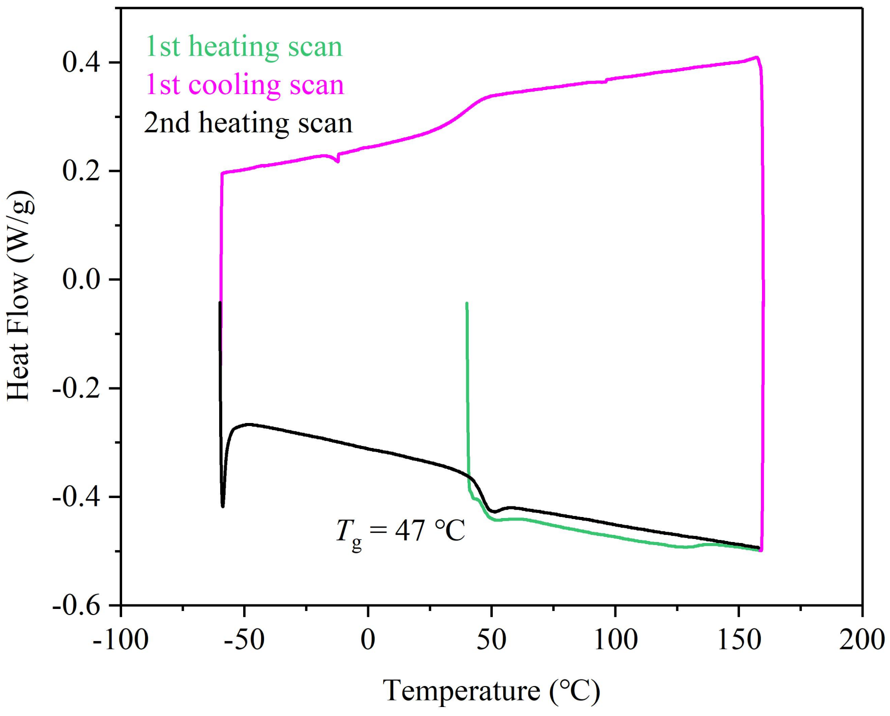

Supplementary Figure 92 DSC curves for P(**M13**), T_g = 47 °C.

DSC Characterizations of P(**M14**)

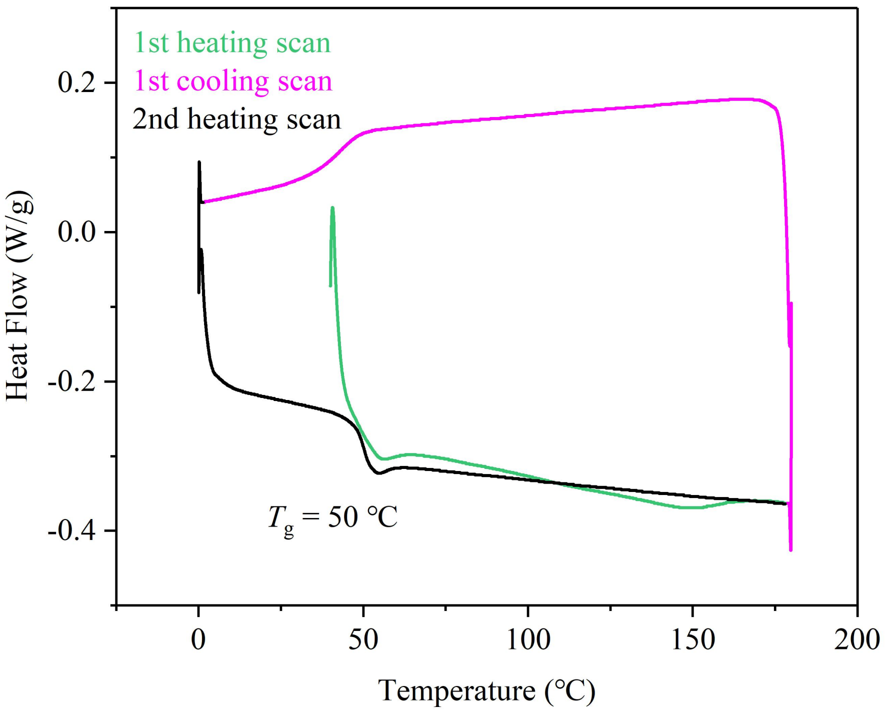

Supplementary Figure 93 DSC curves for P(**M14**), T_g = 50 °C.

DSC Characterizations of P(**M15**)

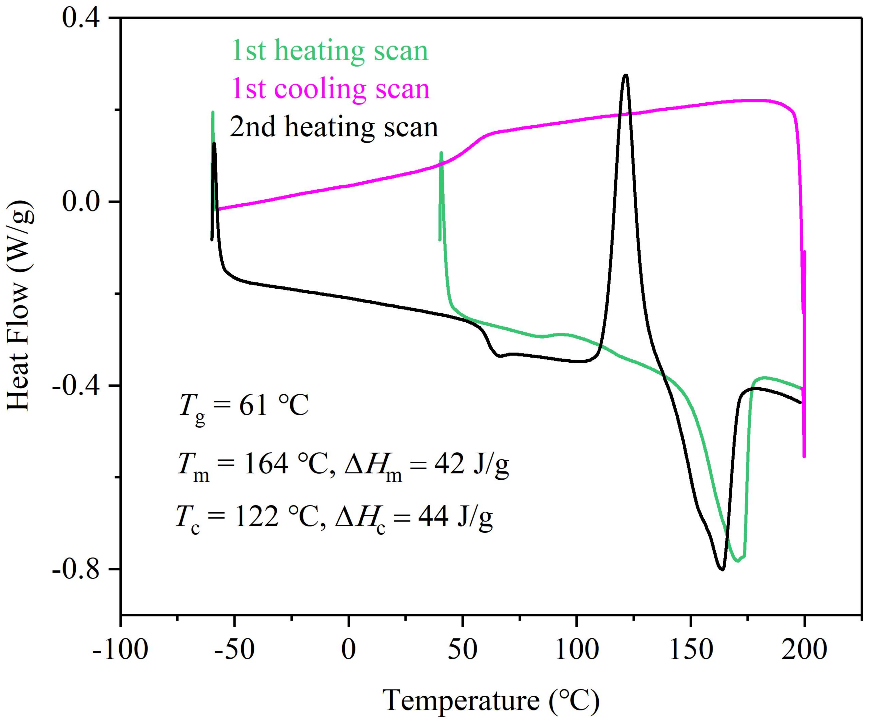

Supplementary Figure 94 DSC curves for P(**M15**), T_g = 61, T_m = 164 °C, T_c = 122 °C.

DSC Characterizations of P(**M16**)

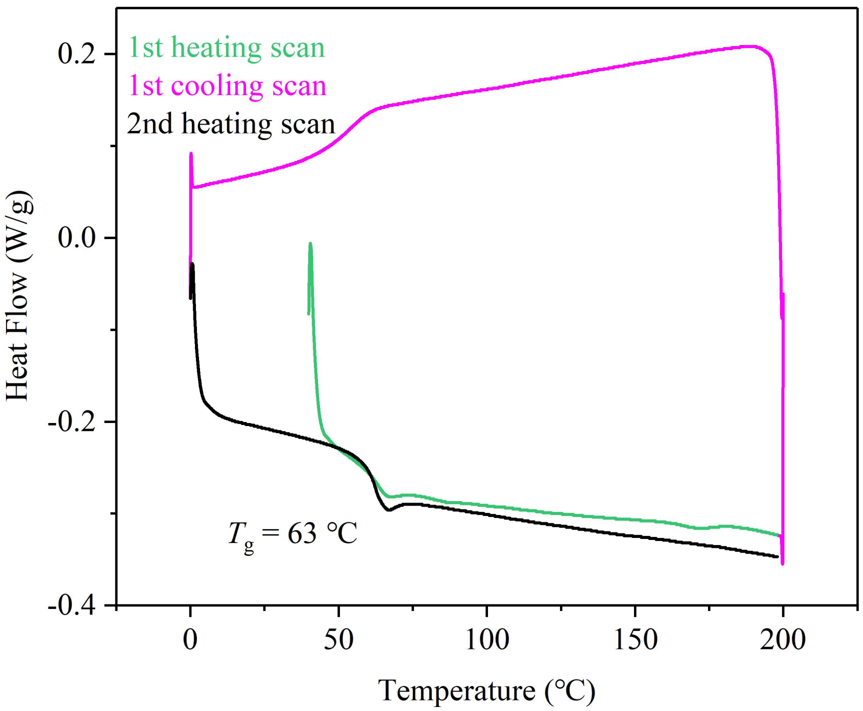

Supplementary Figure 95 DSC curves for P(**M16**), T_g = 63 °C.

DSC Characterizations of P(**M17**)

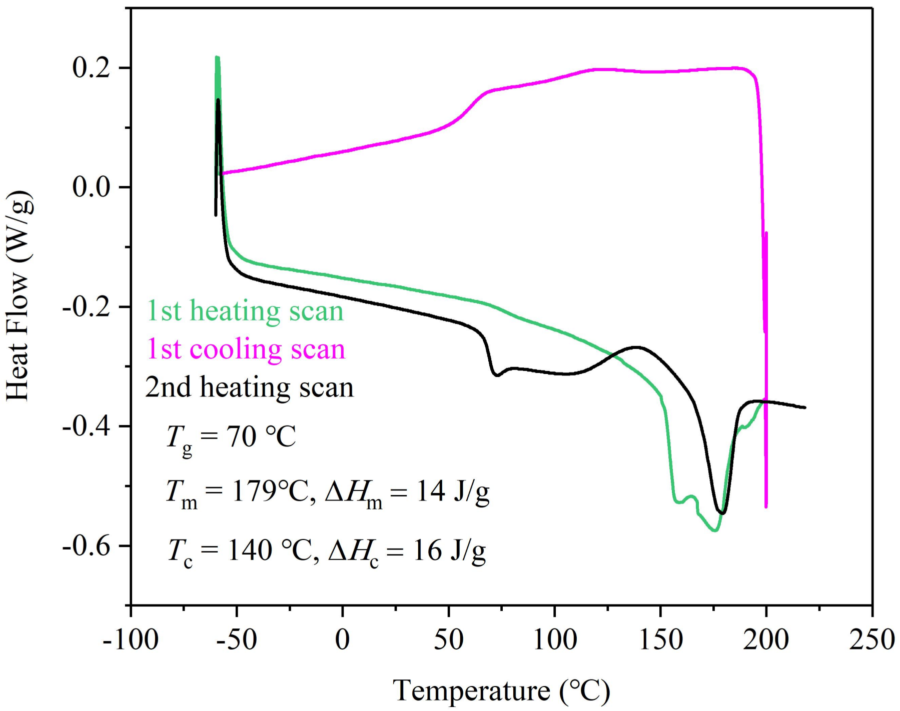

Supplementary Figure 96 DSC curves for P(**M17**), T_g = 70 °C, T_m = 179 °C, T_c = 140 °C.

SEC Traces of Polymers

SEC Characterizations of PCL obtained by [CL]/[**Zn-1**]/[I] = 1000/1/1.

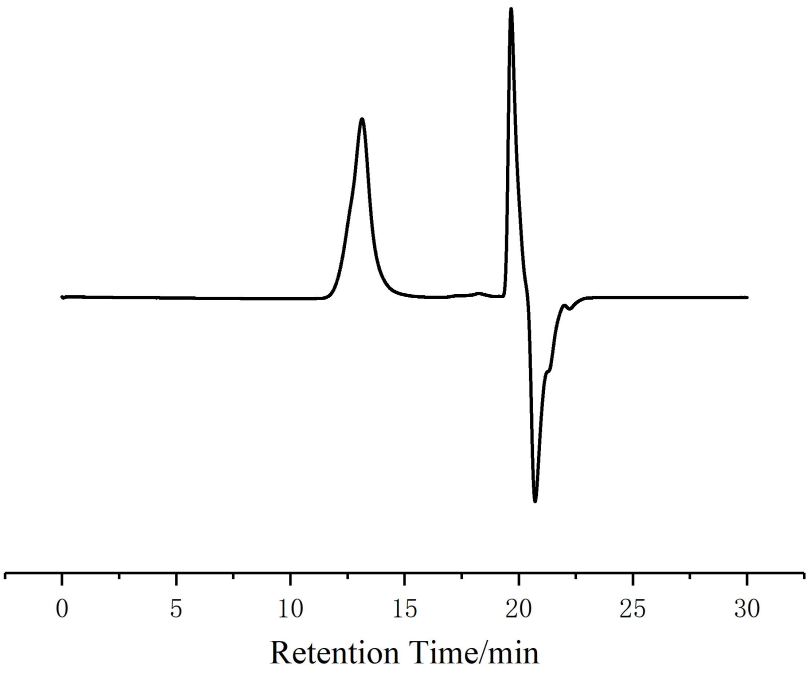

Supplementary Figure 97 SEC trace of PCL, M_n = 121 kg/mol, $Đ$ = 1.35.

SEC Characterizations of P(**M1**) obtained by [**M1**]/[**Zn-1**]/[I] = 1000/1/1.

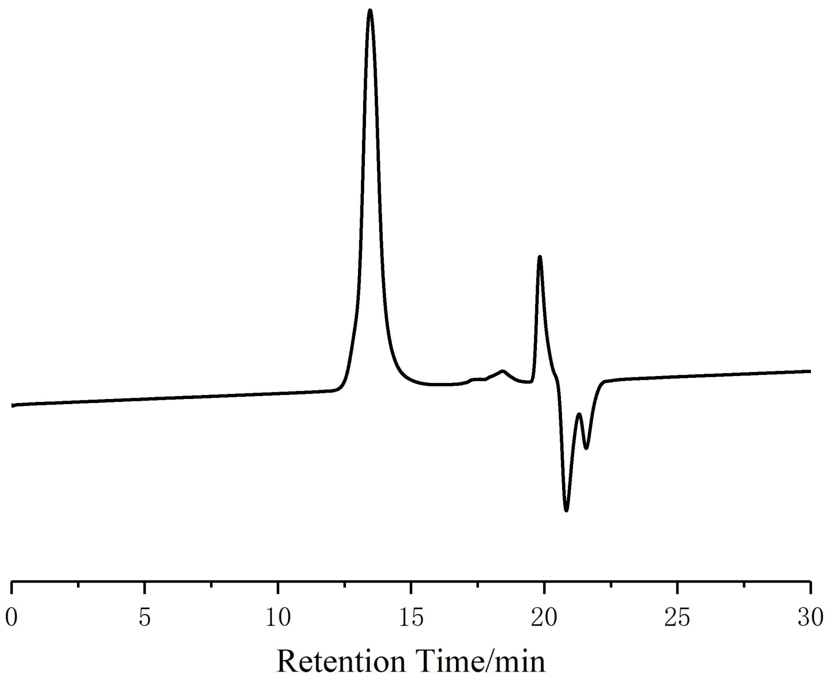

Supplementary Figure 98 SEC trace of P(**M1**), M_n = 93.7 kg/mol, $Đ$ = 1.18.

SEC Characterizations of P(**M2**) obtained by [**M2**]/[**Zn-1**]/[I] = 1000/1/1.

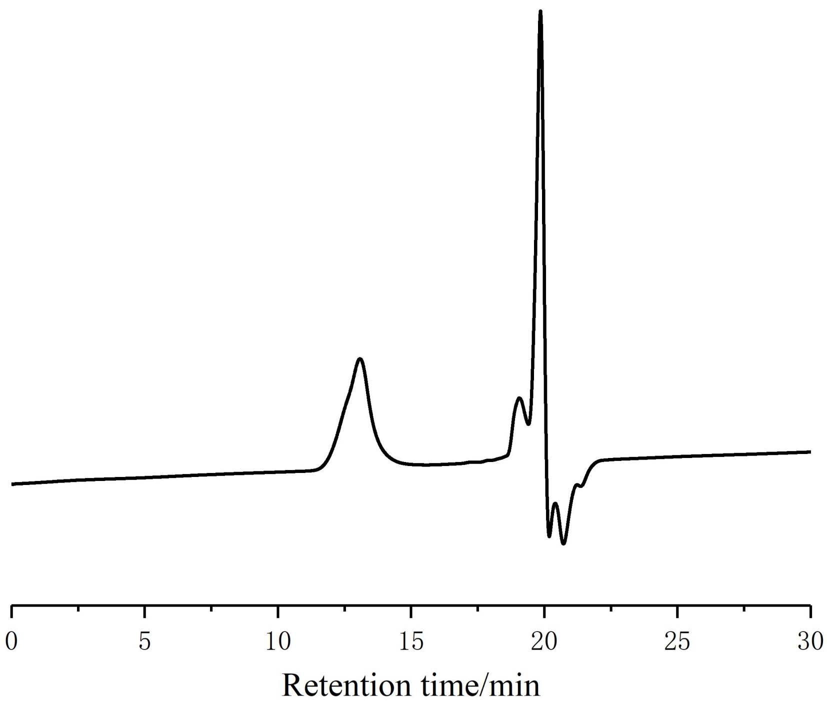

Supplementary Figure 99 SEC trace of P(**M2**), M_n = 137 kg/mol, $Đ$ = 1.43.

SEC Characterizations of P(**M3**) obtained by [**M3**]/[**Zn-1**]/[I] = 1000/1/1.

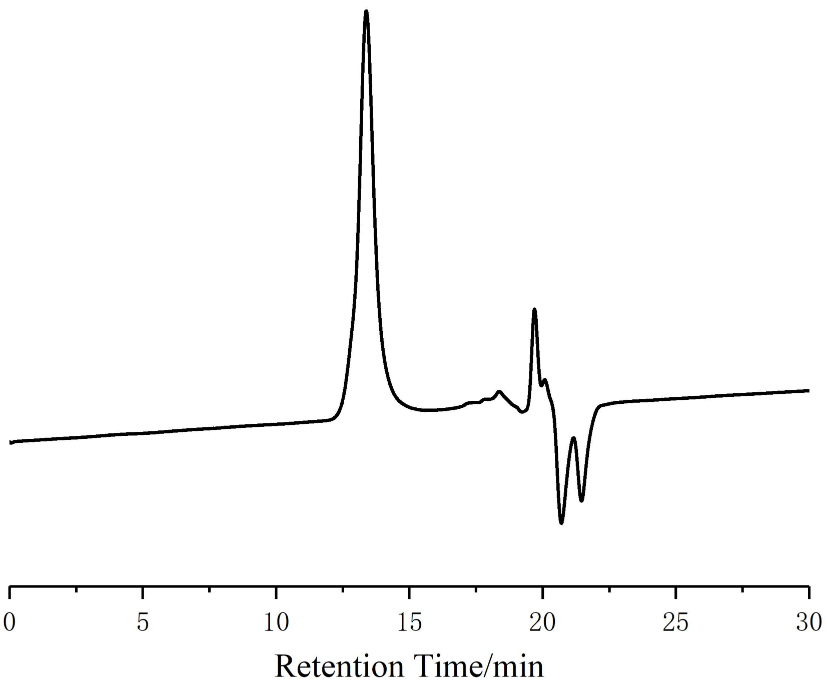

Supplementary Figure 100 SEC trace of P(**M3**), M_n = 103 kg/mol, $Đ$ = 1.19.

SEC Characterizations of P(**M4**) obtained by [**M4**]/[**Zn-1**]/[I] = 1000/1/1.

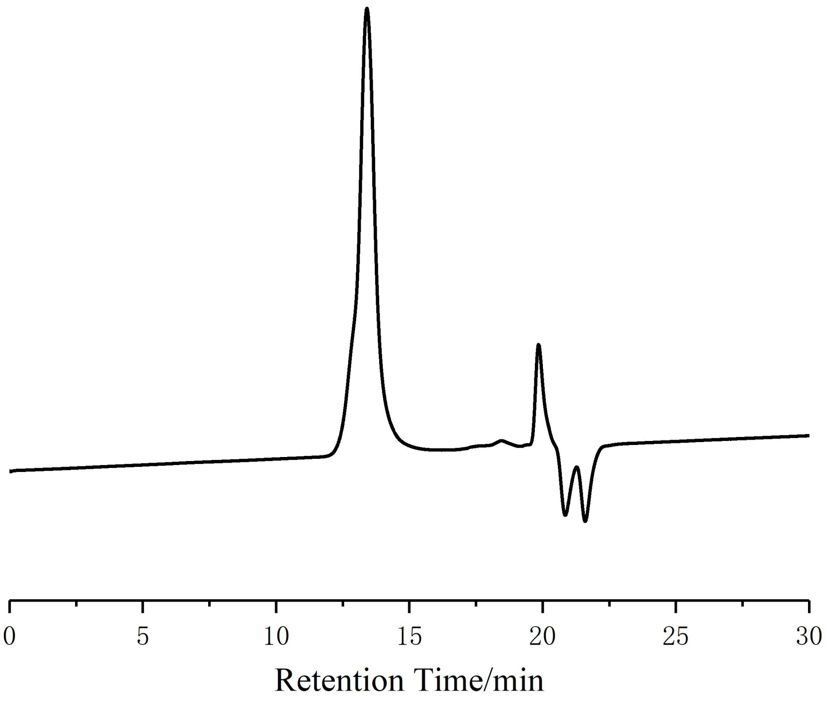

Supplementary Figure 101 SEC trace of P(**M4**), M_n = 107 kg/mol, $Đ$ = 1.18.

SEC Characterizations of P(**M5**) obtained by [**M5**]/[**Zn-1**]/[I] = 1000/1/1.

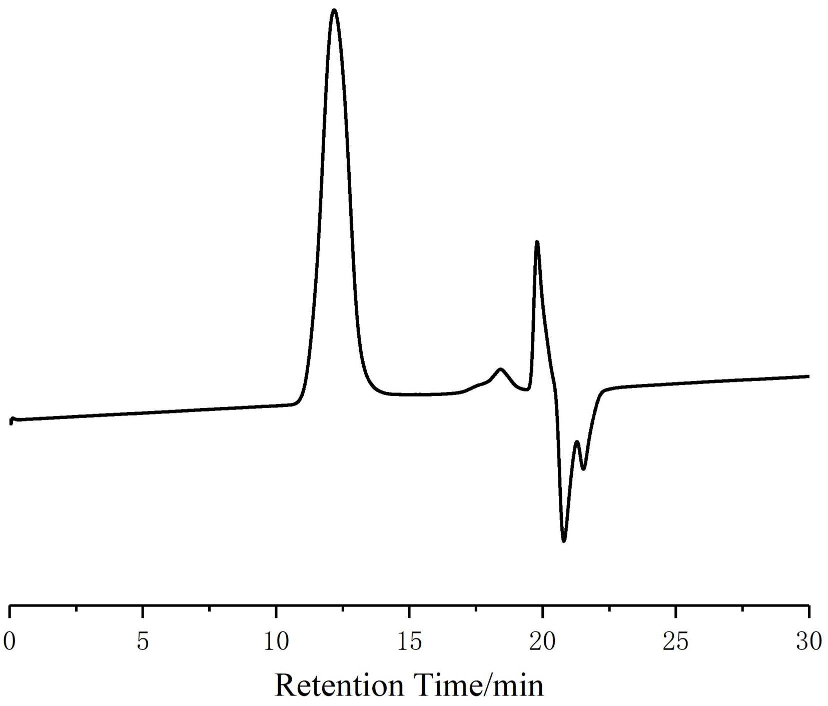

Supplementary Figure 102 SEC trace of P(**M5**), M_n = 110 kg/mol, $Đ$ = 1.11.

SEC Characterizations of P(**M6**) obtained by [**M6**]/[**Zn-1**]/[I] = 1000/1/1.

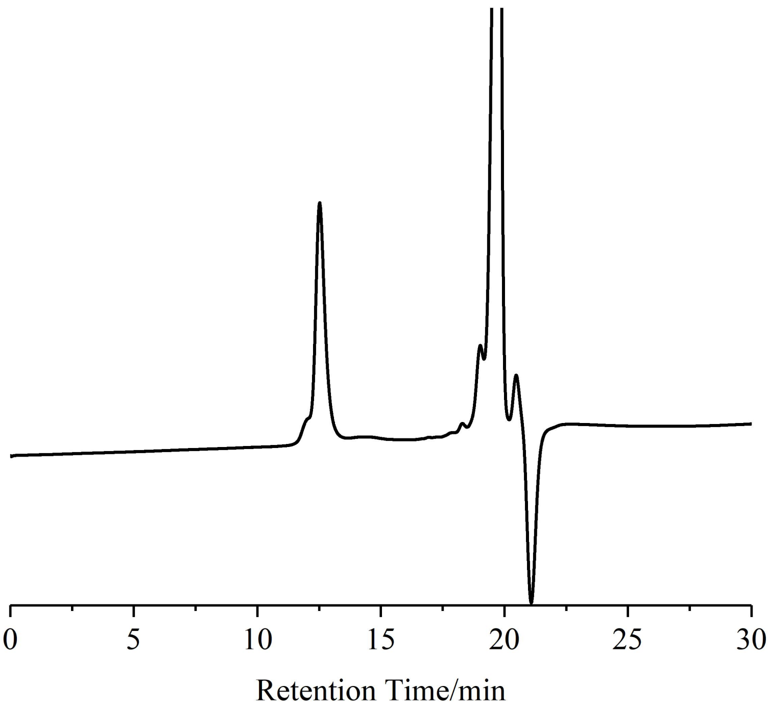

Supplementary Figure 103 SEC trace of P(**M6**), M_n = 209 kg/mol, $Đ$ = 1.05.

SEC Characterizations of P(**M8**) obtained by [**M8**]/[**Zn-1**]/[I] = 1000/1/1.

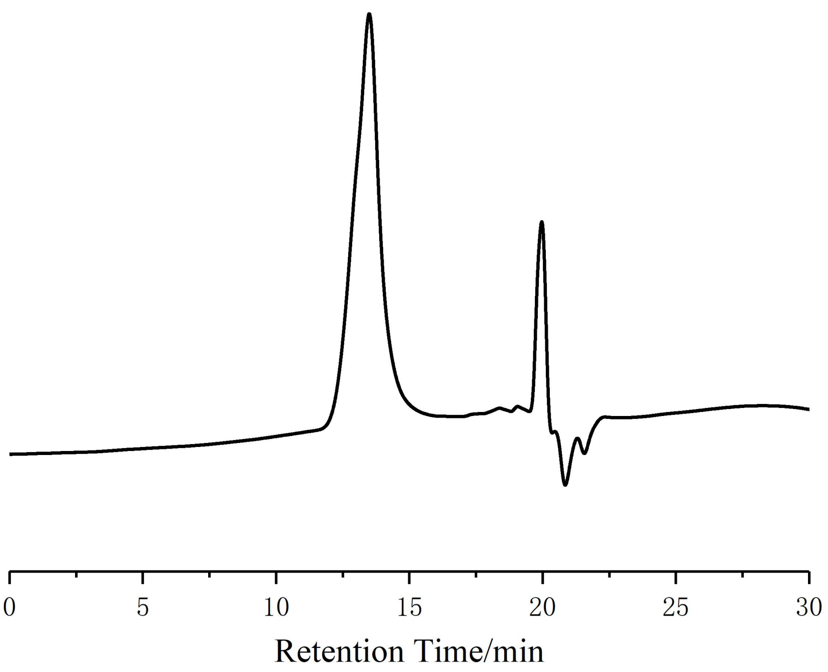

Supplementary Figure 104 SEC trace of P(**M8**), M_n = 103 kg/mol, $Đ$ = 1.34.

SEC Characterizations of P(**M5**) obtained by [**M5**]/[**Y-1**]/[I] = 20000/1/1.

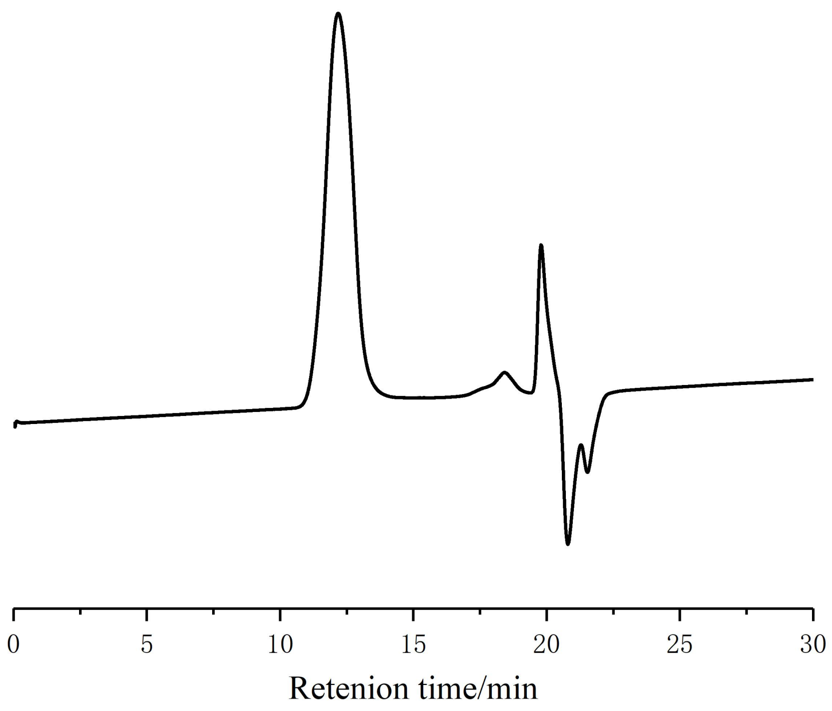

Supplementary Figure 105 SEC trace of P(**M5**), M_n = 386 kg/mol, $Đ$ = 1.31.

SEC Characterizations of P(**M9**) obtained by [**M9**]/[**Y-1**]/[I] = 20000/1/1.

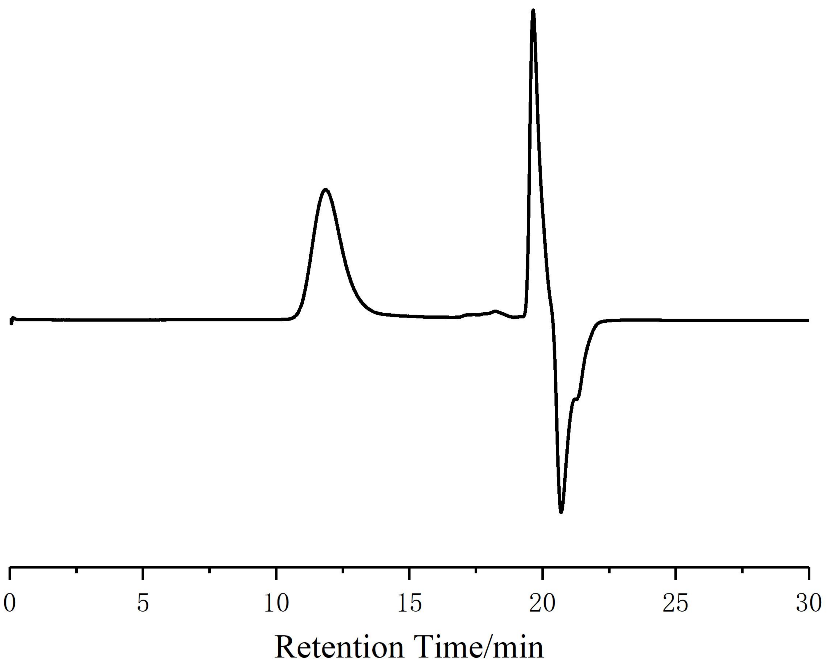

Supplementary Figure 106 SEC trace of P(**M9**), M_n = 407 kg/mol, $Đ$ = 1.43.

SEC Characterizations of P(**M11**) obtained by [**M11**]/[**Y-1**]/[I] = 20000/1/1.

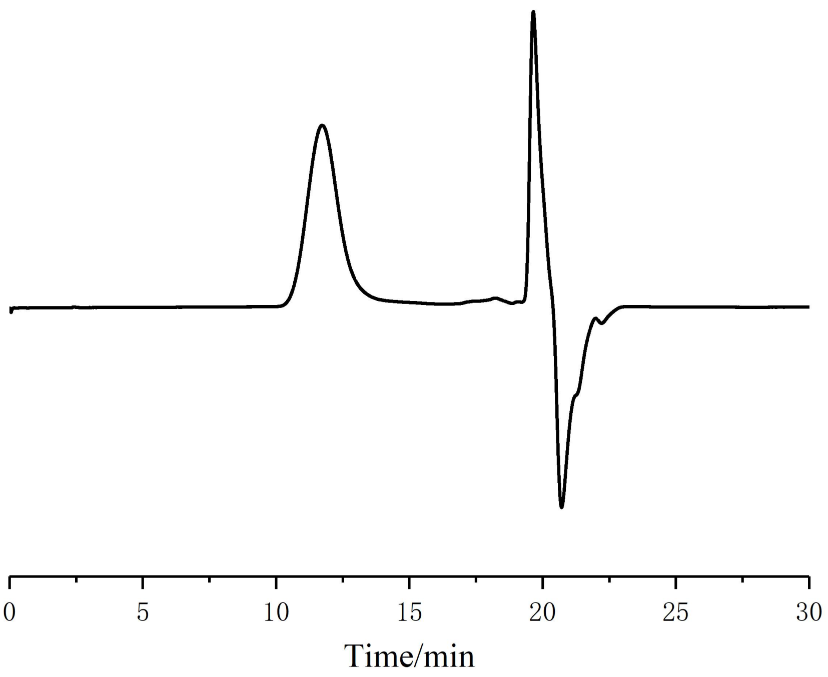

Supplementary Figure 107 SEC trace of P(**M11**), M_n = 497 kg/mol, $Đ$ = 1.49.

Thermodynamic Study

For the testing thermodynamic study of **M1−M8**, we used the following method.
A solution of **M** in d^8-Tol was prepared using a 2 mL volumetric flask. To 4 mL glass vials was added **Zn-1** (0.002−0.01 mmol, [**M**]/[**Zn-1**]/[I] = 50/1/1 or 100/1/1) and **M** solution (0.01−0.50 mmol). The mixture was stirred for 5 min, and the resulting solution was divided into 4 NMR tubes, with each tube containing 0.5 mL reaction solution. These four tubes were placed in four pre-heated oil baths at different temperature (40, 50, 60, and 70 °C). The above procedure was repeated twice so that three parallel samples were studied at each temperature. According to our kinetic studies, all polymerizations at room temperature could reach their equilibrium within ~1.5 h (Supplementary Figure 112). After the reaction reached the equilibrium, each polymerization for the thermodynamic studies was quenched with 0.1 mL $CHCl_3$ acidified with benzoic acid (2%). The monomer concentration at the equilibrium was determined by ^{1}H NMR spectroscopy.

For the thermodynamic study of **M9−M12**, we used the variable temperature NMR spectroscopy to monitor the polymerization since these monomers were prone to acetal hydrolysis under the acidic conditions.
In an argon-filled glovebox, a toluene-d_8 stock solution of **M** (0.1–0.5 mmol) and *p*-tolylmethanol (0.002–0.01 mmol) was prepared in a 1 mL volumetric flask was added to 4 mL glass reactors which was charged with **Zn-1** (0.002–0.01 mmol, [**M**]/[**Zn-1**]/[I] = 50/1/1). 0.6 mL stock solution was added to an NMR tube. Then the NMR sample was sealed and brought outside of glovebox. Variable-temperature ^{1}H NMR (400 MHz) study of the reaction mixture was conducted. The polymerization reaction reached equilibrium in ~30 mins at 25–65 °C (Supplementary Figures 119–123) and the conversion of **M** was monitored by ^{1}H NMR spectroscopy.

The Van't Hoff plot of ln[**M**]$_{eq}$ versus 1/T gave a linear fitting with a slope and an intercept, from which the thermodynamic parameters were calculated to be ΔH_p^o and ΔS_p^o, based on the equation ln[**M**]$_{eq}$ = ΔH_p^o/RT − ΔS_p^o/R, where R is the molar gas constant. T_c was calculated to be $\Delta H_p^o/\Delta S_p^o$ at [**M**]$_0$ = 1 M, based on the equation $T_c = \Delta H_p^o/(\Delta S_p^o + R\ln [\mathbf{M}]_0)$.

Summary of thermodynamic results

Supplementary Table 3. Polymerization Thermodynamic Data for Monomers.

Entry	**M**	ΔH_p^o (kJ/mol)	ΔS_p^o (J/mol K^{-1})	T_c at 1 M (°C)
1	**M9**	−17.0	−21.9	503
2	**M10**	−17.8	−27.9	365
3	**M11**	−18.5	−30.2	340
4	**M12**	−19.7	−41.9	197

Thermodynamic Calculation of **M1**

Supplementary Table 4. Raw data over equilibrium conversion at various temperatures for **M1**.

Run	T (°C)	T (K)	Conversion (%)	$[M]_0$ (mol/L)	$T^{-1}\times10^3$ (K^{-1})	$[M]_{eq}$ (mol/L)	$\ln[M]_{eq}$ (mol/L)
			88.22			0.01178	-4.4414
1	40	313	87.21	0.1	3.19	0.01279	-4.3591
			87.69			0.01231	-4.3973
			86.17			0.01383	-4.2809
2	50	323	86.17	0.1	3.10	0.01383	-4.2809
			84.84			0.01516	-4.1891
			82.17			0.01604	-4.1327
3	60	333	82.98	0.1	3	0.01702	-4.0734
			83.96			0.01783	-4.0269
			79.42			0.02058	-3.8834
4	70	343	78.93	0.1	2.91	0.02107	-3.8599
			77.85			0.02215	-3.8099

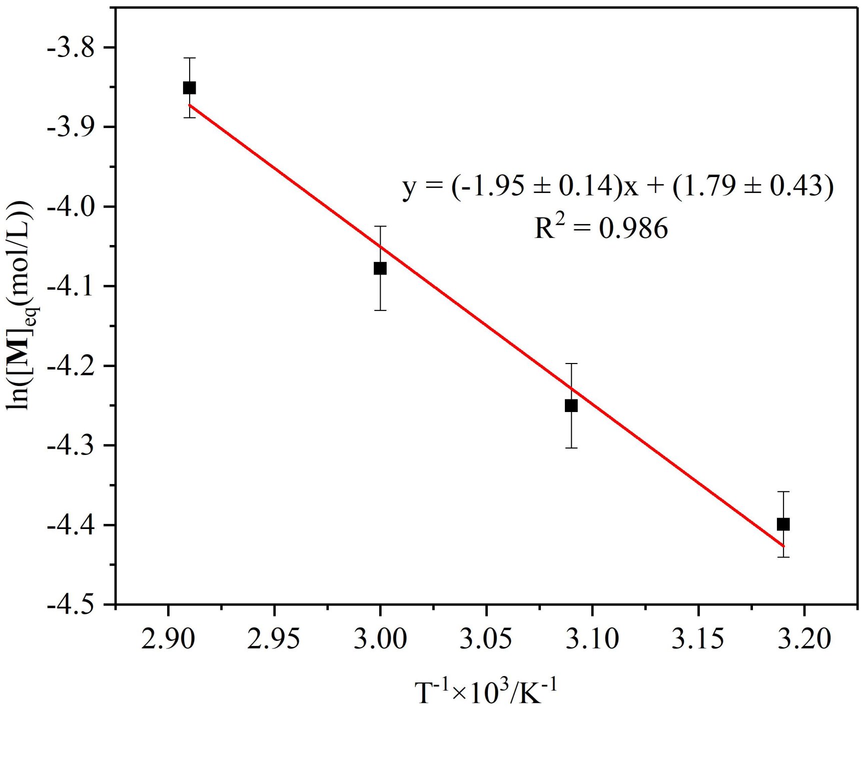

Supplementary Figure 108 Van't Hoff plot of $\ln[\mathbf{M1}]_{eq}$ *vs.* reciprocal of the absolute temperature (T^{-1}).

Thermodynamic Calculation of **M2**

Supplementary Table 5. Raw data over equilibrium conversion at various temperatures for **M2**.

Run	T (°C)	T (K)	Conversion (%)	$[M]_0$ (mol/L)	$T^{-1}\times10^3$ (K^{-1})	$[M]_{eq}$ (mol/L)	$\ln[M]_{eq}$ (mol/L)
			74.26			0.02574	-3.6597
1	40	313	74.68	0.1	3.19	0.02564	-3.6762
			73.79			0.02621	-3.6416
			68.70			0.03130	-3.4641
2	50	323	70.97	0.1	3.10	0.02903	-3.5394
			70.54			0.02946	-3.5247
			65.34			0.03466	-3.3622
3	60	333	65.34	0.1	3	0.03466	-3.3622
			63.44			0.03656	-3.3088
			58.93			0.04107	-3.1925
4	70	343	58.93	0.1	2.91	0.04107	-3.1925
			59.35			0.04065	-3.2028

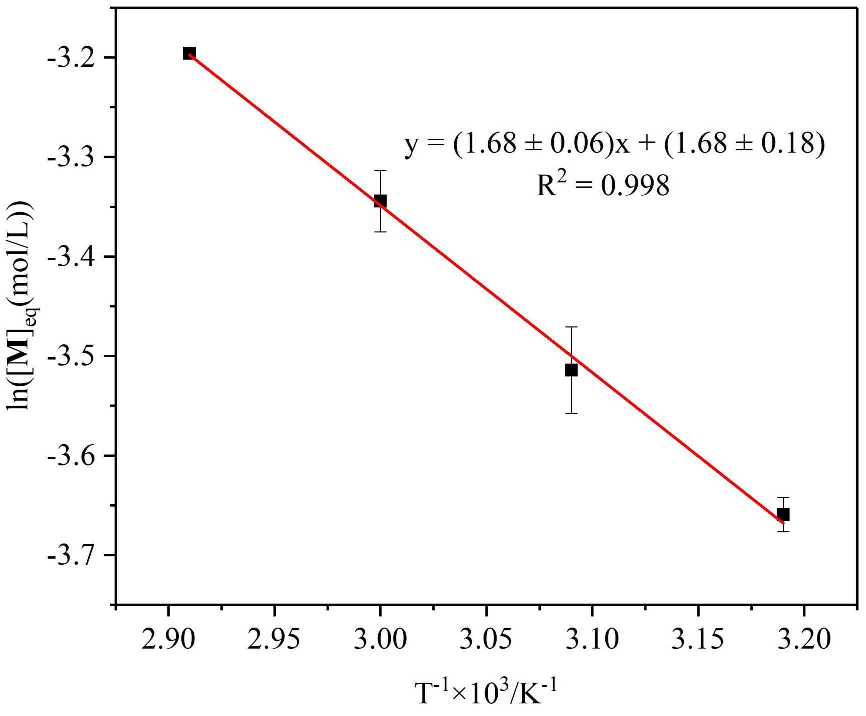

Supplementary Figure 109 Van't Hoff plot of $\ln[\mathbf{M2}]_{eq}$ *vs.* reciprocal of the absolute temperature (T^{-1}).

Thermodynamic Calculation of **M3**

Supplementary Table 6. Raw data over equilibrium conversion at various temperatures for **M3**.

Run	T (°C)	T (K)	Conversion (%)	$[M]_0$ (mol/L)	$T^{-1}\times10^3$ (K^{-1})	$[M]_{eq}$ (mol/L)	$\ln[M]_{eq}$ (mol/L)
			77.25			0.04551	-3.0898
1	40	313	77.90	0.2	3.19	0.04420	-3.1190
			77.80			0.04440	-3.1145
			73.44			0.05312	-2.9352
2	50	323	73.54	0.2	3.10	0.05292	-2.9390
			72.64			0.05472	-2.9055
			68.25			0.06349	-2.7569
3	60	333	67.69	0.2	3	0.06462	-2.7392
			67.85			0.06431	-2.7440
			63.17			0.07366	-2.6083
4	70	343	63.47	0.2	2.91	0.07313	-2.6155
			63.90			0.07220	-2.6283

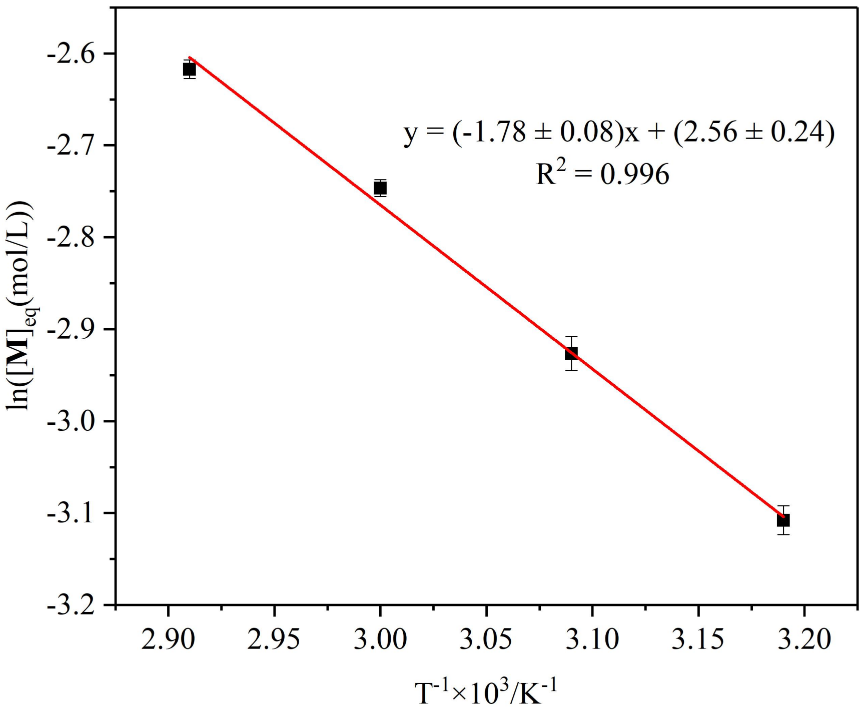

Supplementary Figure 110 Van't Hoff plot of $\ln[\mathbf{M3}]_{eq}$ *vs.* reciprocal of the absolute temperature (T^{-1}).

Thermodynamic Calculation of **M4**

Supplementary Table 7. Raw data over equilibrium conversion at various temperatures for **M4**.

Run	T (°C)	T (K)	Conversion (%)	$[M]_0$ (mol/L)	$T^{-1}\times10^3$ (K^{-1})	$[M]_{eq}$ (mol/L)	$\ln[M]_{eq}$ (mol/L)
			70.10			0.1495	-1.9372
1	40	313	69.33	0.5	3.19	0.1534	-1.8750
			69.60			0.1520	-1.8859
			67.48			0.1626	-1.8165
2	50	323	65.99	0.5	3.10	0.1701	-1.7717
			66.10			0.1695	-1.7749
			60.08			0.1996	-1.6114
3	60	333	61.46	0.5	3	0.1927	-1.6466
			61.54			0.1923	-1.6487
			54.85			0.2257	-1.4885
4	70	343	55.46	0.5	2.91	0.2227	-1.5019
			53.38			0.2331	-1.4563

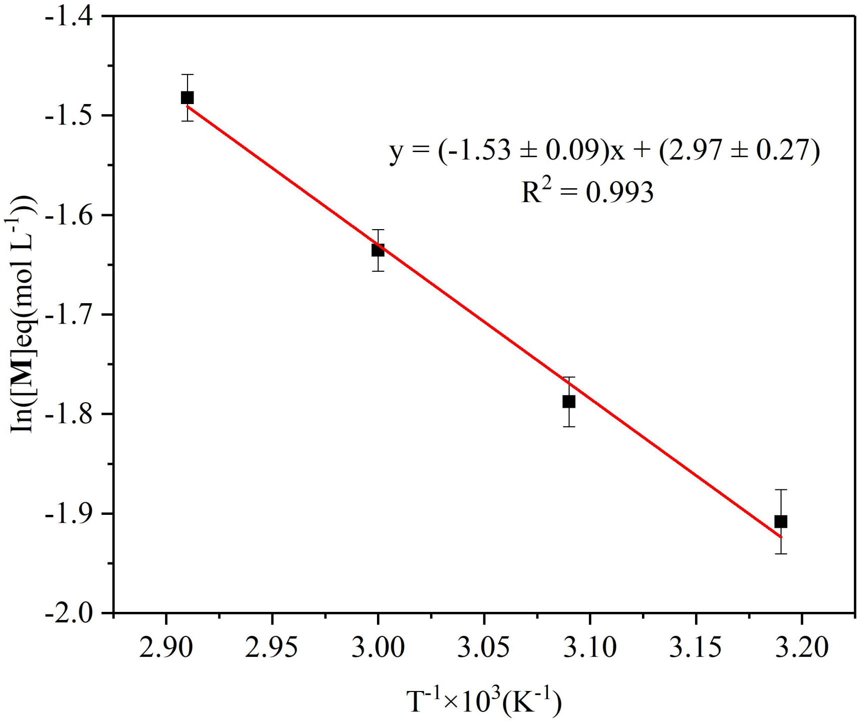

Supplementary Figure 111 Van't Hoff plot of $\ln[\mathbf{M4}]_{eq}$ *vs.* reciprocal of the absolute temperature (T^{-1}).

Thermodynamic Calculation of **M5**

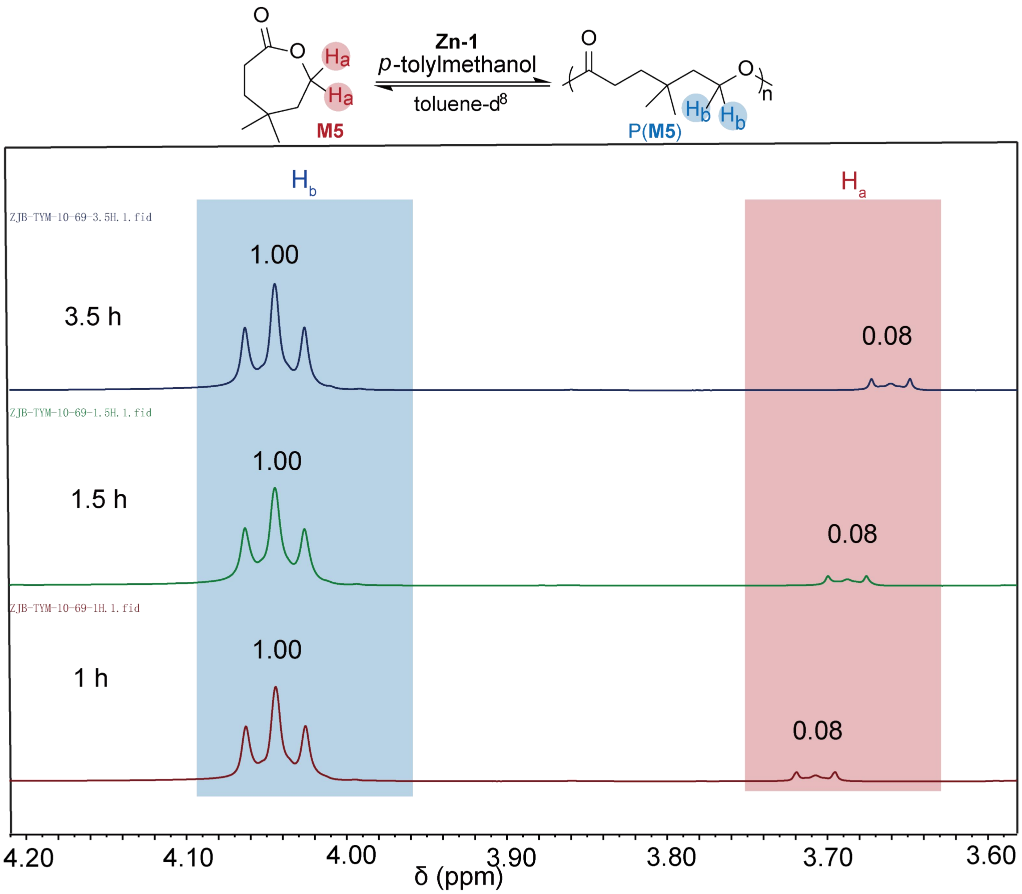

Supplementary Figure 112 ^{1}H NMR spectra (400 MHz, d^{8}-Tol) of the reaction mixture from ROP of **M5** at 40 °C monitored over 4 h. The reaction could reach equilibrium within 1 h. The integration ratio of the resonance around 3.98–4.11 ppm and the resonance around 3.60–3.74 ppm was used to calculate the concentration ratio of P(**M5**) and **M5** in the reaction mixture.

Supplementary Table 8. Raw data over equilibrium conversion at various temperatures for **M5**.

Run	T (°C)	T (K)	Conversion (%)	$[M]_0$ (mol/L)	T^{-1}×10^3 (K^{-1})	$[M]_{eq}$ (mol/L)	ln$[M]_{eq}$ (mol/L)
			92.21			0.03895	-3.2455
1	40	313	92.50	0.5	3.19	0.03751	-3.2831
			92.37			0.03817	-3.2657
			91.80			0.04100	-3.1190
2	50	323	91.09	0.5	3.10	0.04456	3.1109
			90.70			0.04642	-3.0700
			88.95			0.05525	-2.8959
3	60	333	89.18	0.5	3	0.05411	-2.9167
			89.17			0.05417	-2.9156
			87.58			0.06210	-2.7790
4	70	343	87.39	0.5	2.91	0.06305	-2.7638
			87.08			0.06460	-2.7395

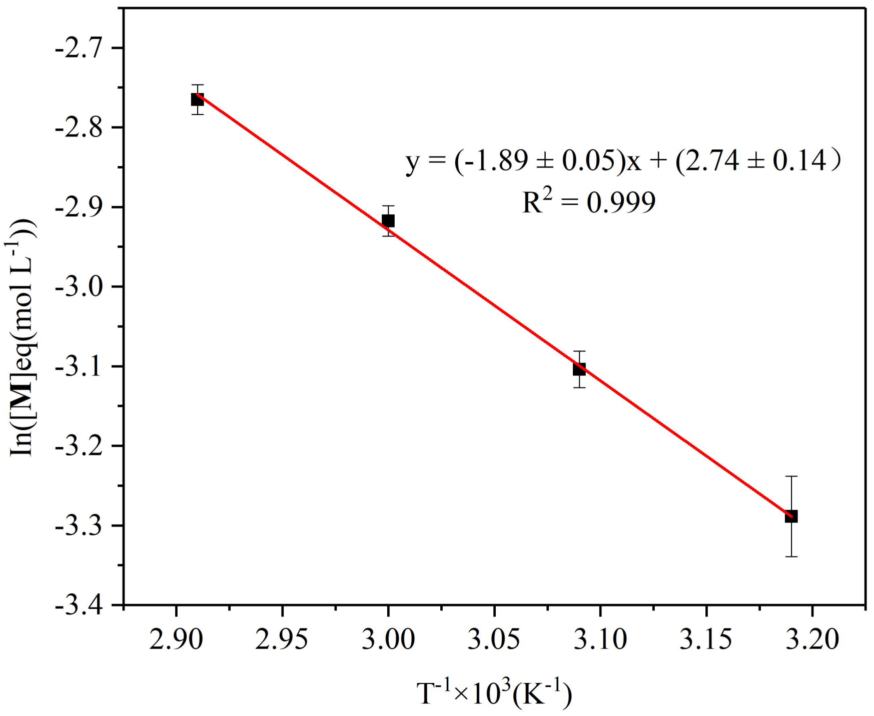

Supplementary Figure 113 Van't Hoff plot of ln[**M5**]$_{eq}$ *vs.* reciprocal of the absolute temperature (T^{-1}).

Thermodynamic Calculation of **M6**

Supplementary Table 9. Raw data over equilibrium conversion at various temperatures for **M6**.

Run	T (°C)	T (K)	Conversion (%)	$[M]_0$ (mol/L)	$T^{-1}\times10^3$ (K^{-1})	$[M]_{eq}$ (mol/L)	$\ln[M]_{eq}$ (mol/L)
			94.29			0.02855	-3.5560
1	40	313	93.89	0.5	3.19	0.03053	-3.4920
			94.33			0.02836	-3.5628
			92.84			0.03579	-3.3301
2	50	323	92.92	0.5	3.10	0.03541	-3.3408
			93.39			0.03303	-3.4103
			90.71			0.04645	-3.0694
3	60	333	90.49	0.5	3	0.04755	-3.0460
			90.74			0.04630	-3.0726
			88.46			0.05770	-2.8525
4	70	343	89.58	0.5	2.91	0.05211	-2.9544
			88.24			0.05880	-2.8336

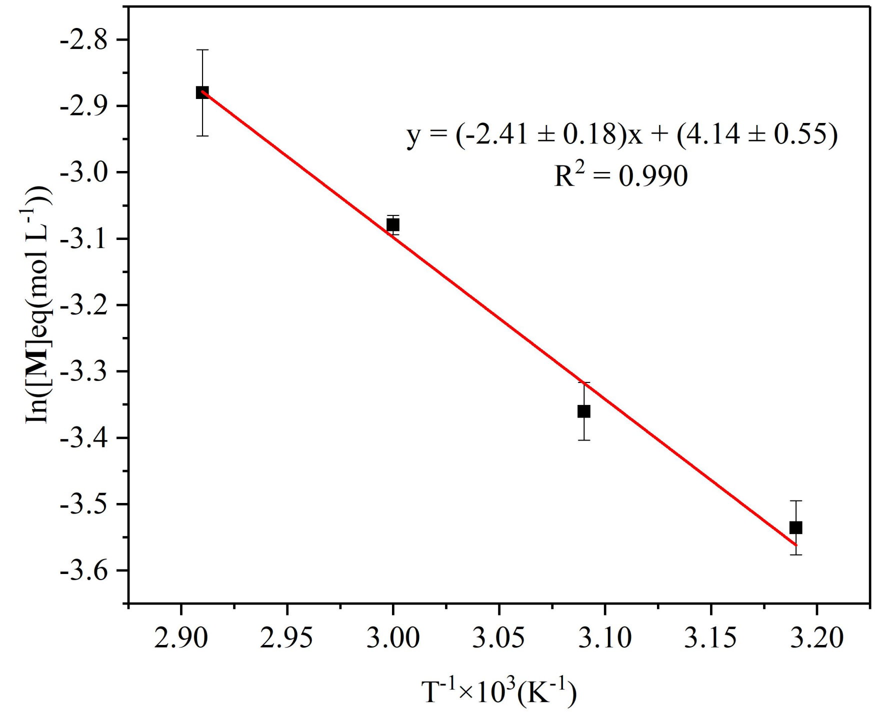

Supplementary Figure 114 Van't Hoff plot of $\ln[\mathbf{M6}]_{eq}$ *vs.* reciprocal of the absolute temperature (T^{-1}).

Thermodynamic Calculation of **M8**

Supplementary Table 10. Raw data over equilibrium conversion at various temperatures for **M8**.

Run	T (°C)	T (K)	Conversion (%)	$[M]_0$ (mol/L)	$T^{-1}\times10^3$ (K^{-1})	$[M]_{eq}$ (mol/L)	$\ln[M]_{eq}$ (mol/L)
			89.70			0.02060	-3.8825
1	40	313	89.95	0.2	3.19	0.02010	-3.9070
			89.80			0.02040	-3.8922
			87.21			0.02571	-3.6609
2	50	323	87.42	0.2	3.10	0.02516	-3.6825
			87.47			0.02506	-3.6865
			84.80			0.03040	-3.4933
3	60	333	85.65	0.2	3	0.02870	-3.5509
			84.59			0.03082	-3.4796
			81.75			0.03650	-3.3063
4	70	343	81.58	0.2	2.91	0.03683	-3.3014
			81.45			0.03710	-3.2941

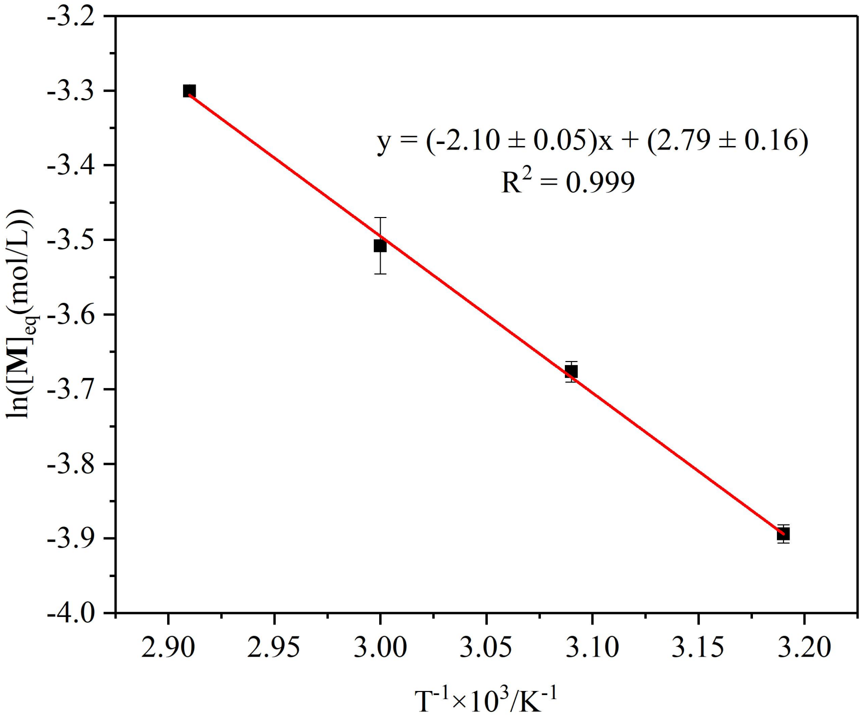

Supplementary Figure 115 Van't Hoff plot of $\ln[\mathbf{M8}]_{eq}$ *vs.* reciprocal of the absolute temperature (T^{-1}).

Thermodynamic Calculation of **M9**

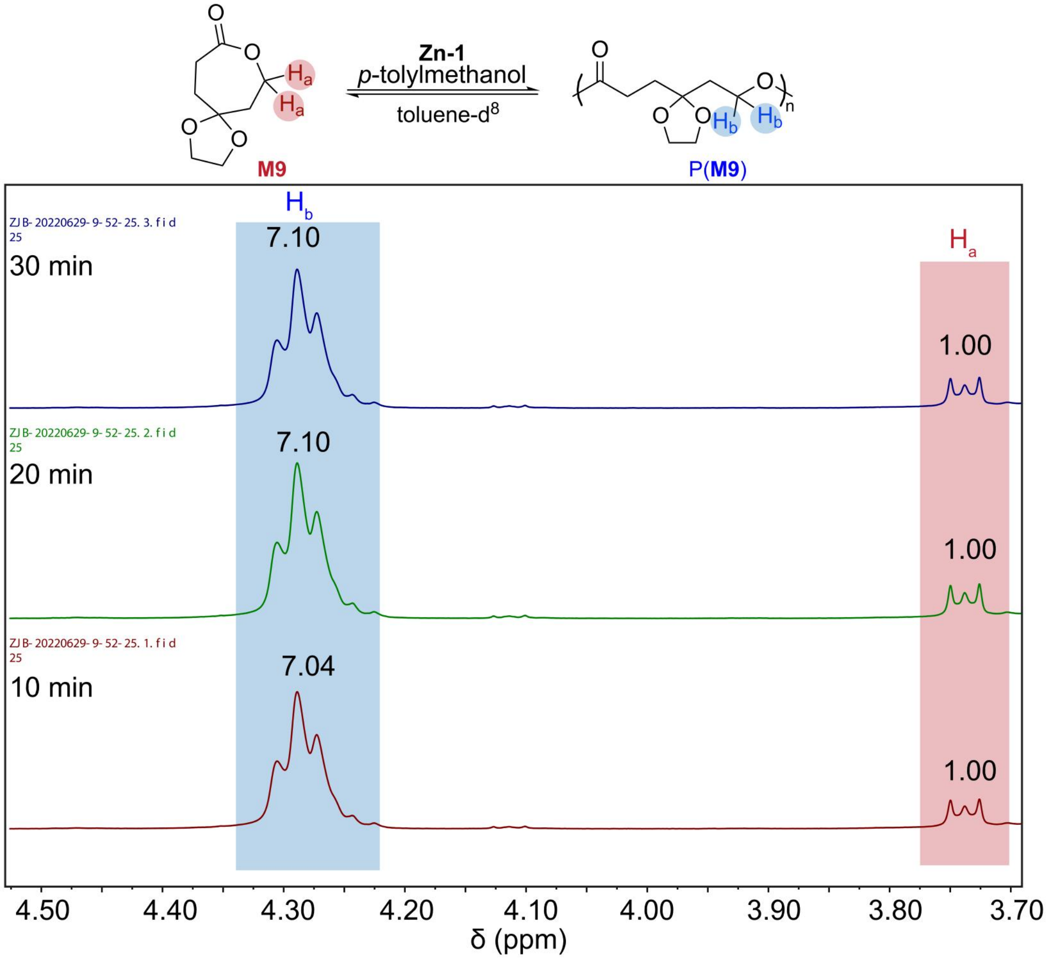

Supplementary Figure 116 ^{1}H NMR spectra (400 MHz, d^8-Tol) of the reaction mixture from ROP of **M9** at 25 °C monitored over 30 mins. The integration ratio of the resonance centered at ~4.28 ppm and the resonance centered at ~3.73 ppm was used to calculate the concentration ratio of P(**M9**) and **M9** in the reaction mixture.

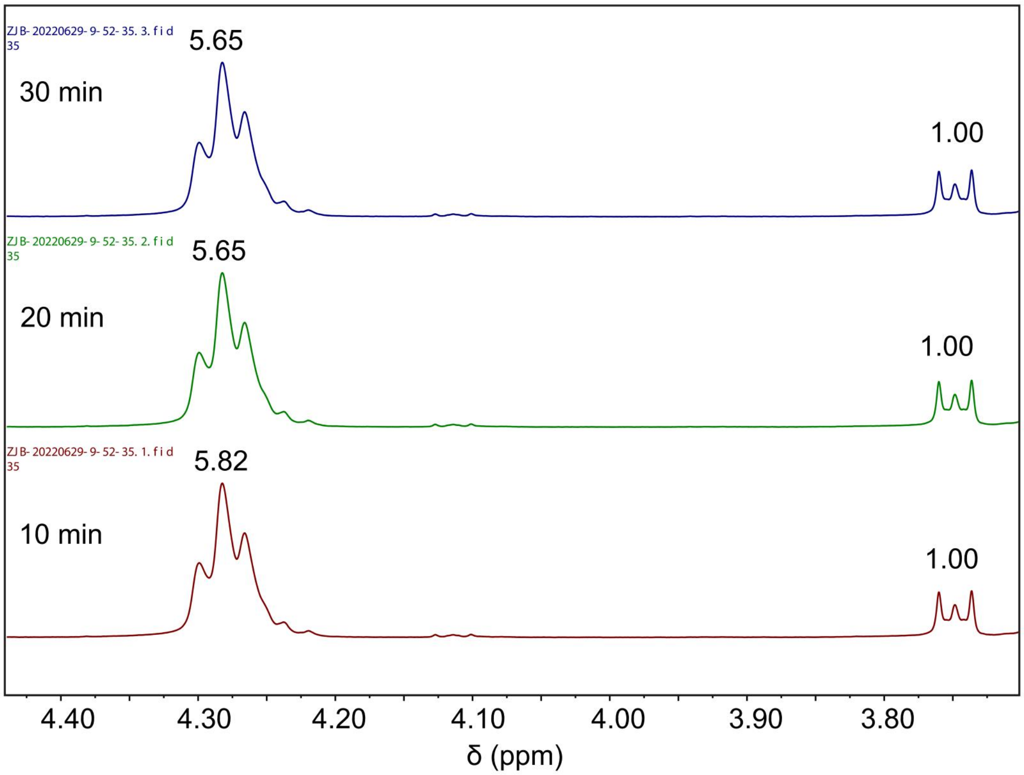

Supplementary Figure 117 ^1H NMR spectra (400 MHz, d^8-Tol) of the reaction mixture from ROP of **M9** at 35 °C monitored over 30 mins.

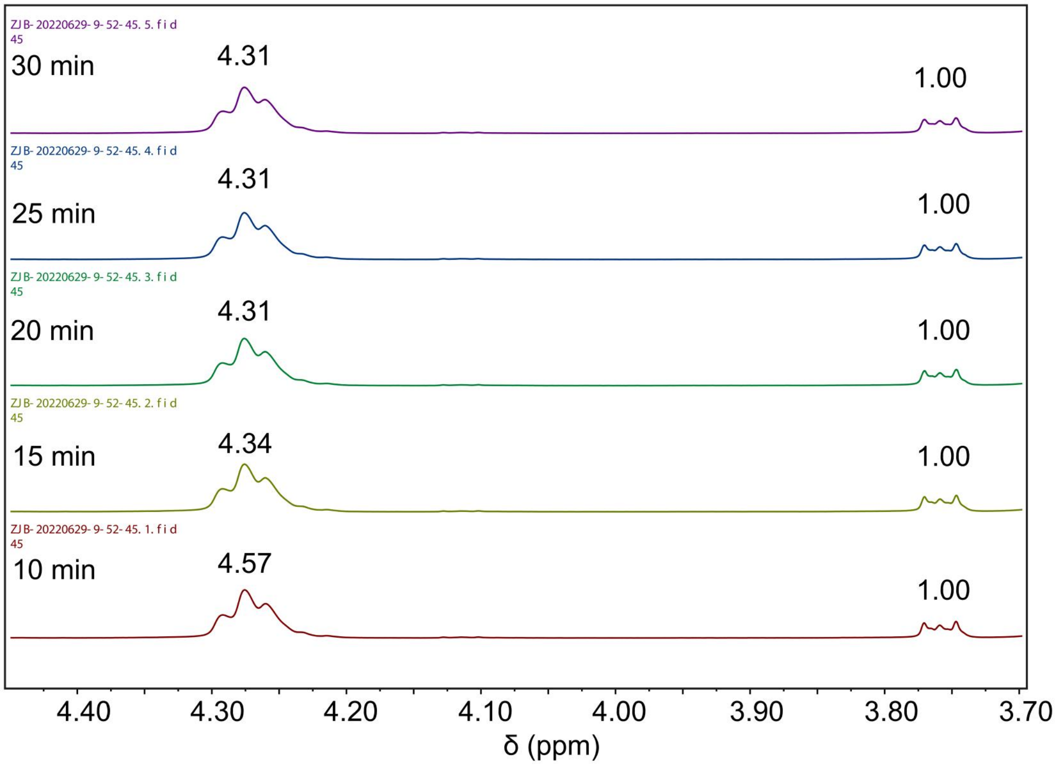

Supplementary Figure 118 ^{1}H NMR spectra (400 MHz, d^8-Tol) of the reaction mixture from ROP of **M9** at 45 °C monitored over 30 mins.

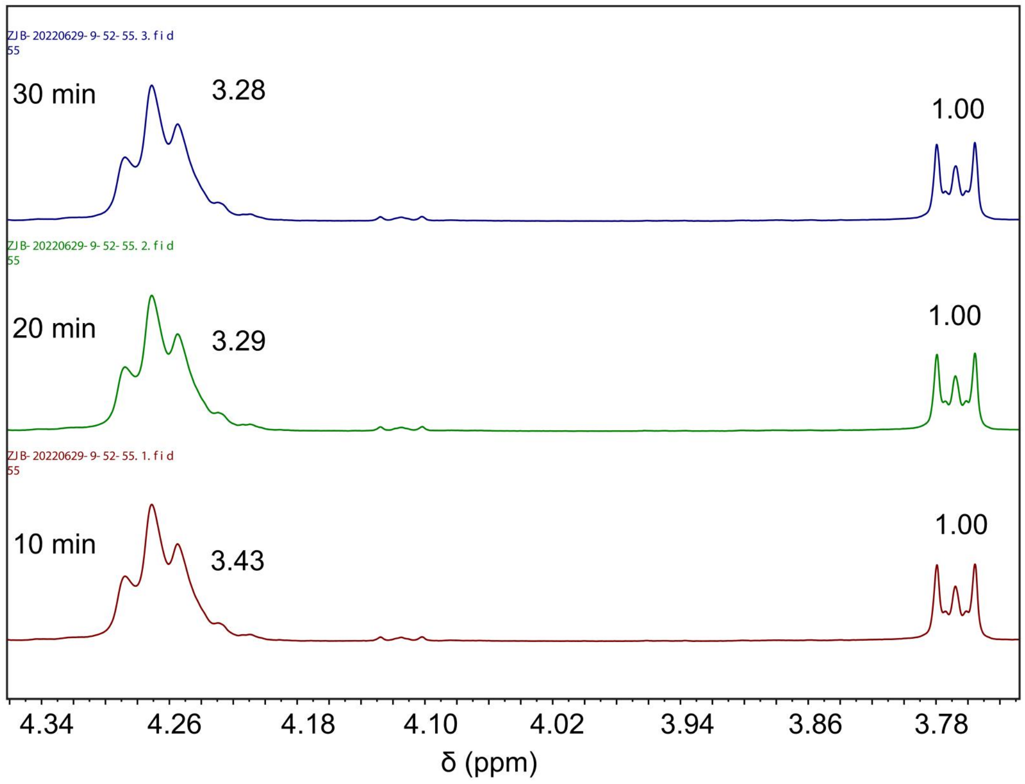

Supplementary Figure 119 ^{1}H NMR spectra (400 MHz, d^8-Tol) of the reaction mixture from ROP of **M9** at 55 °C monitored over 30 mins.

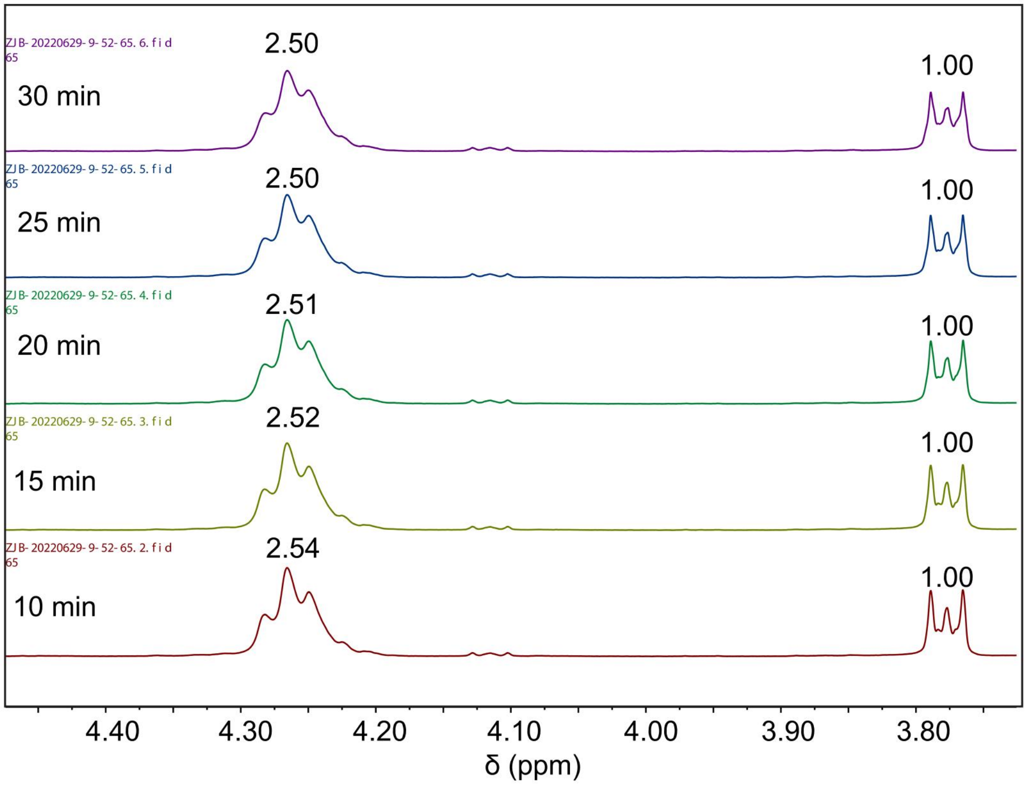

Supplementary Figure 120 ^{1}H NMR spectra (400 MHz, d^8-Tol) of the reaction mixture from ROP of **M9** at 65 °C monitored over 30 mins.

Supplementary Table 11. Raw data over equilibrium conversion at various temperatures for **M9**.

Run	T (°C)	T (K)	Conversion (%)	$[M]_0$ (mol/L)	$T^{-1}\times10^3$ (K^{-1})	$[M]_{eq}$ (mol/L)	$\ln[M]_{eq}$ (mol/L)
1	25	298	87.67	0.1	3.36	0.01233	-4.3957
2	35	308	84.96	0.1	3.25	0.01504	-4.1970
3	45	318	81.13	0.1	3.14	0.01897	-3.9649
4	55	328	76.64	0.1	3.05	0.02336	-3.7567
5	65	338	71.39	0.1	2.96	0.02865	-3.5526

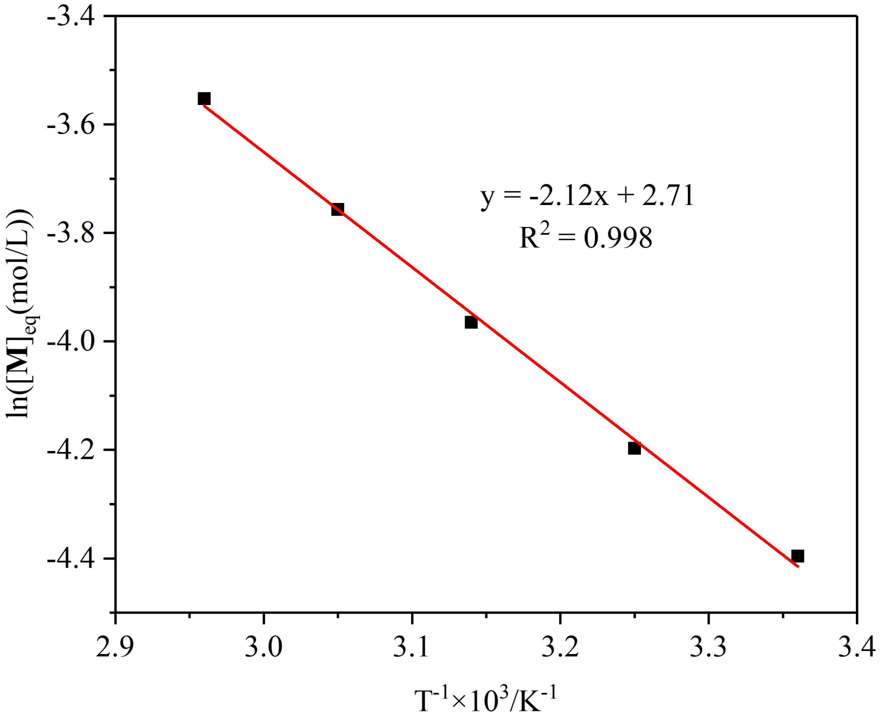

Supplementary Figure 121 Van't Hoff plot of ln[**M9**]$_{eq}$ *vs.* reciprocal of the absolute temperature (T^{-1}).

Thermodynamic Calculation of **M10**

Supplementary Table 12 . Raw data over equilibrium conversion at various temperatures for **M10**.

Run	T (°C)	T (K)	Conversion (%)	$[M]_0$ (mol/L)	$T^{-1}\times10^3$ (K^{-1})	$[M]_{eq}$ (mol/L)	$\ln[M]_{eq}$ (mol/L)
1	25	298	89.28	0.2	3.36	0.02144	-3.8425
2	35	308	86.34	0.2	3.25	0.02732	-3.6001
3	45	318	83.05	0.2	3.14	0.03390	-3.3843
4	55	328	79.72	0.2	3.05	0.04156	-3.1806
5	65	338	74.62	0.2	2.96	0.05076	-2.9806

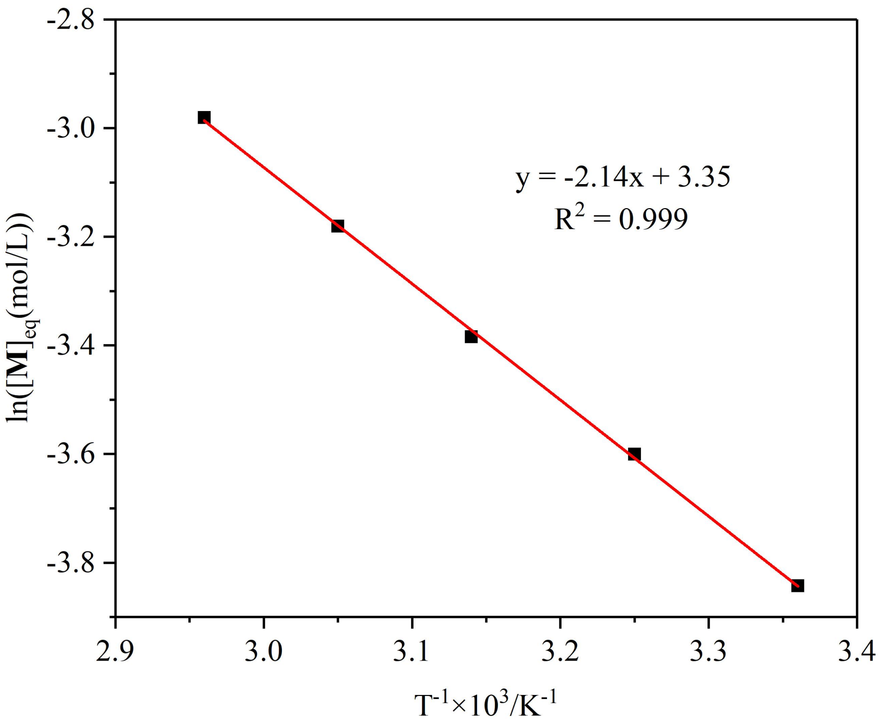

Supplementary Figure 122 Van't Hoff plot of ln[**M10**]$_{eq}$ *vs.* reciprocal of the absolute temperature (T^{-1}).

Thermodynamic Calculation of **M11**

Supplementary Table 13. Raw data over equilibrium conversion at various temperatures for **M11**.

Run	T (°C)	T (K)	Conversion (%)	$[M]_0$ (mol/L)	$T^{-1}\times10^3$ (K^{-1})	$[M]_{eq}$ (mol/L)	$\ln[M]_{eq}$ (mol/L)
1	25	298	88.84	0.2	3.36	0.02232	-3.8023
2	35	308	85.92	0.2	3.25	0.02816	-3.5699
3	45	318	82.18	0.2	3.14	0.03565	-3.3340
4	55	328	77.94	0.2	3.05	0.04412	-3.1208
5	65	338	72.98	0.2	2.96	0.05405	-2.9178

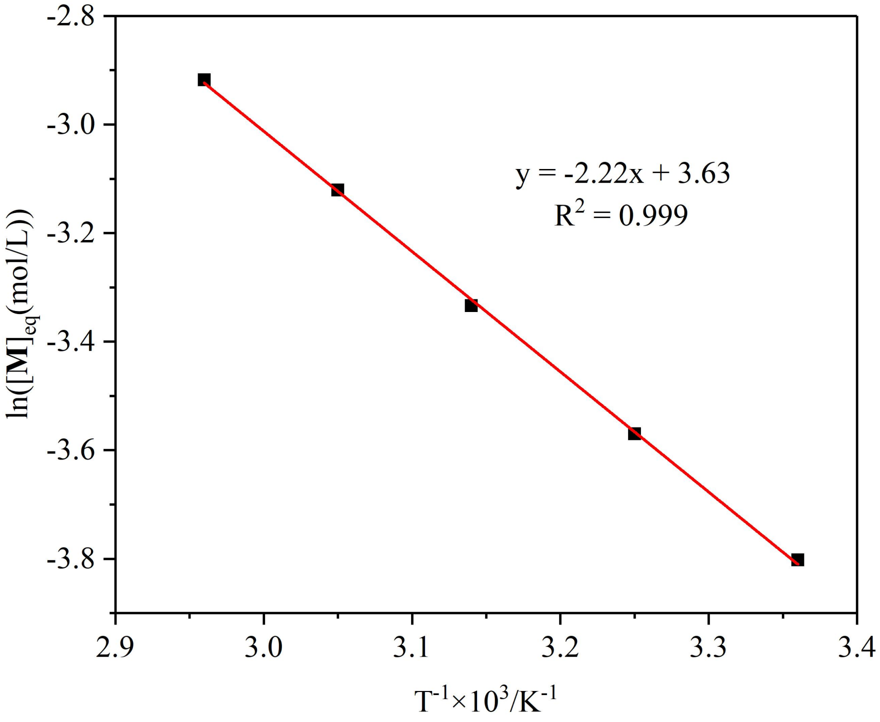

Supplementary Figure 123 Van't Hoff plot of $\ln[\mathbf{M11}]_{eq}$ *vs.* reciprocal of the absolute temperature (T^{-1}).

Thermodynamic Calculation of **M12**

Supplementary Table 14 Raw data over equilibrium conversion at various temperatures for **M12**.

Run	T (°C)	T (K)	Conversion (%)	$[M]_0$ (mol/L)	$T^{-1}\times10^3$ (K^{-1})	$[M]_{eq}$ (mol/L)	$\ln[M]_{eq}$ (mol/L)
1	25	298	89.01	0.5	3.36	0.05495	-2.9013
2	35	308	85.73	0.5	3.25	0.07133	-2.6404
3	45	318	82.14	0.5	3.14	0.08930	-2.4158
4	55	328	77.40	0.5	3.05	0.1130	-2.1804
5	65	338	71.72	0.5	2.96	0.1414	-1.9562

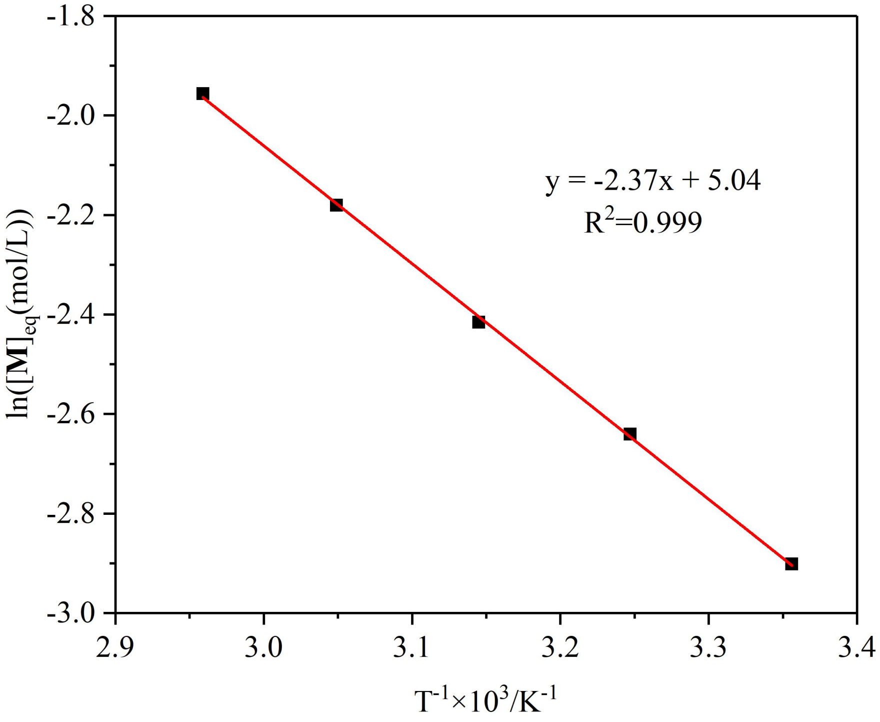

Supplementary Figure 124 Van't Hoff plot of $\ln[\mathbf{M12}]_{eq}$ *vs.* reciprocal of the absolute temperature (T^{-1}).

Chemical Recycling to Monomer (CRM)

General procedure for the CRM of polymers in dilute solutions

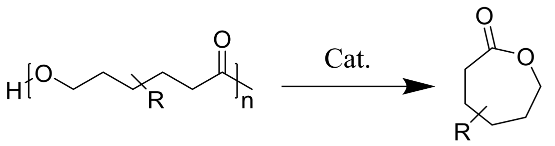

Supplementary Table 15. Results of chemical recycling of P(**M**)s in dilute solution [a]

Run	Polymer	M_n[b] (kg/mol)	$Đ$[b] (M_w/M_n)	Monomer Conversion[b] (%)
1	P(**M1**)	81.9	1.68	69
2	P(**M2**)	158	1.48	88
3	P(**M3**)	92.3	1.75	93
4	P(**M4**)	42.9	1.36	>99
5	P(**M5**)	60.4	1.66	94
6	P(**M6**)	83.0	1.14	96
7	P(**M8**)	48.0	1.32	91
8	P(**M9**)	61.7	1.52	88
9	P(**M10**)	87.3	1.66	92
10	P(**M11**)	69.3	1.62	93
11	P(**M12**)	60.6	1.40	>99
12	P(**M13**)	116	1.15	96
13	P(**M14**)	153	1.85	96
14	P(**M16**)	68.3	1.22	94

[a]Condition: All reactions were in toluene (0.02 M) and glovebox, catalyst: **Zn-1** 2 mol%, time: 1 h, T: 140 °C. [b]Number-average molecular weight (M_n) and dispersity index ($Đ = M_w/M_n$), determined by gel permeation chromatography (SEC) at 40 °C in THF. [c]The monomer conversion determined by ^1H NMR analysis.

Supplementary Table 16. Results of chemical recycling of P(**M1**) in dilute solution [a]

Run	T (°C)	C (mol/L)	Monomer Conversion[b] (%)
1	70	0.02	44
2	140	0.01	86
3	140	0.04	41

[a]Condition: All reactions were in glovebox, catalyst: **Zn-1** 2 mol%, P(**M1**) (M_n = 115 kg/mol, $Đ$ = 1.65), time: overnight. [b]The monomer conversion determined by ^1H NMR analysis.

Solution Depolymerization of P(**M1**)

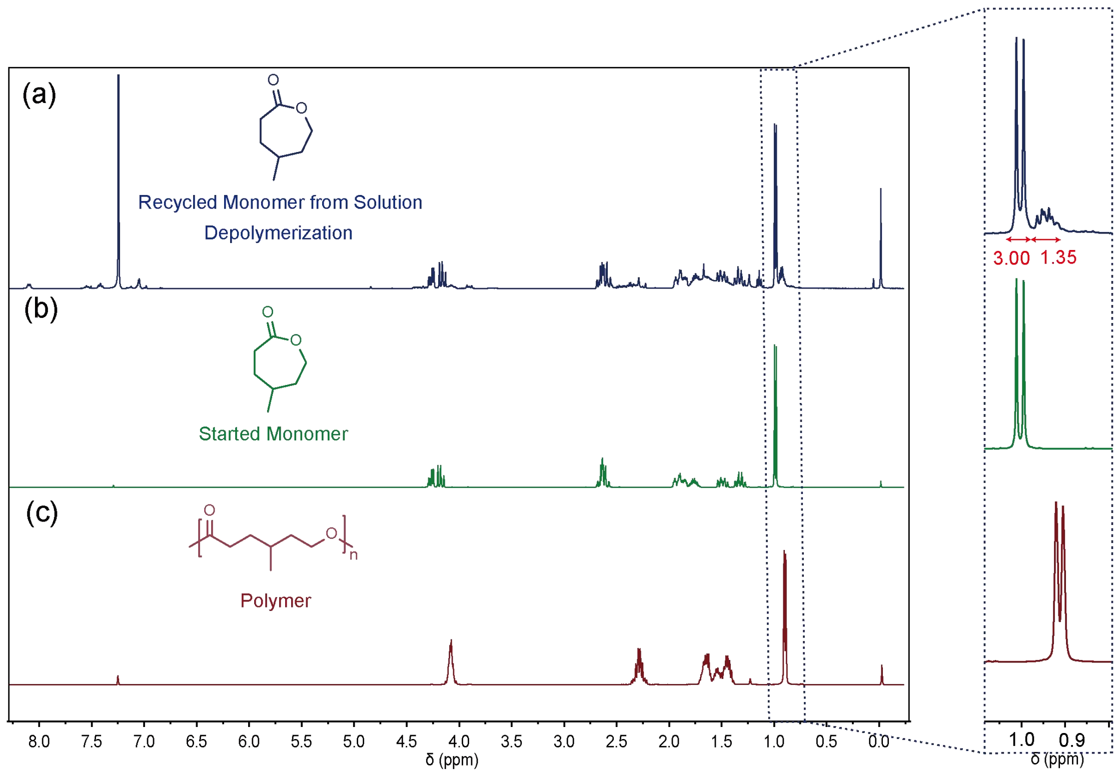

Supplementary Figure 125 ^{1}H NMR spectra of a) recycled **M1** by the solution depolymerization (Monomer conversion = 3.00/(3.00 + 1.35) = 0.69), top; b) starting **M1** for comparison, middle; c) P(**M1**) (M_n = 81.9 kg/mol, $Đ$ = 1.68), bottom.

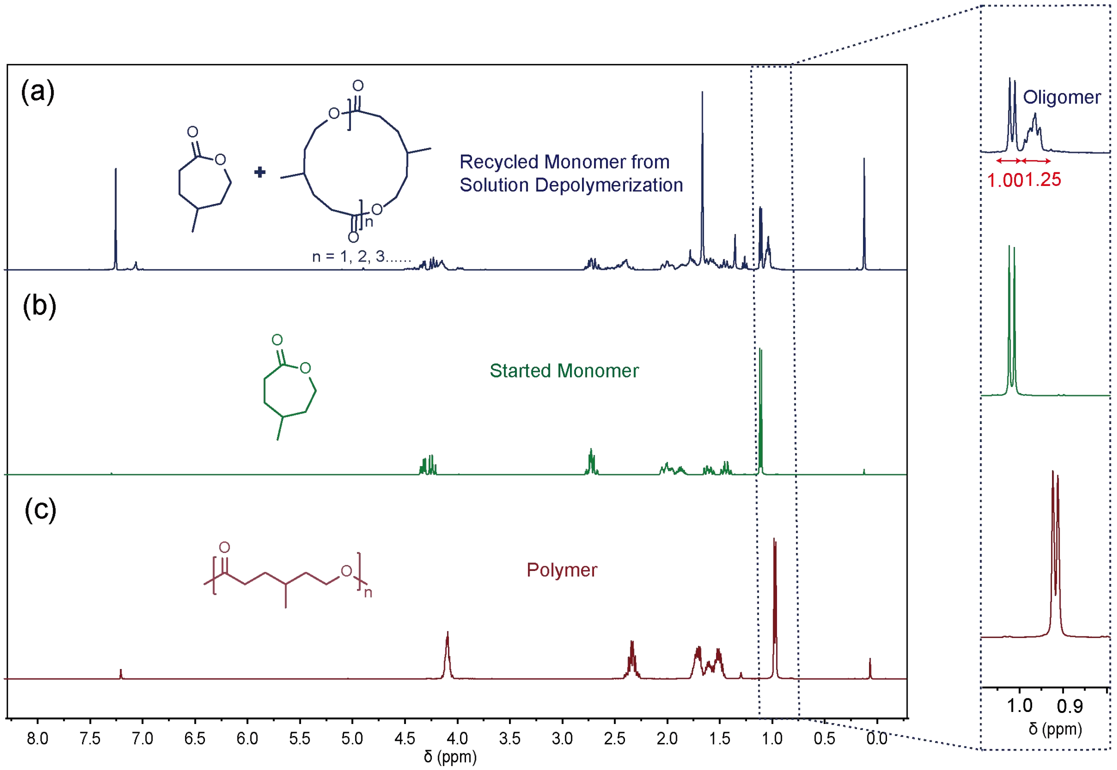

Supplementary Figure 126 Depolymerization study of P(**M1**) at 70 °C. ^{1}H NMR spectra of a) recycled **M1** by the solution depolymerization (**Supplementary Table 16** entry 1, monomer conversion = 1.00/(1.00 + 1.25) = 0.44), top; b) starting **M1** for comparison, middle; c) P(**M1**) (M_n = 115 kg/mol, $Đ$ = 1.65), bottom.

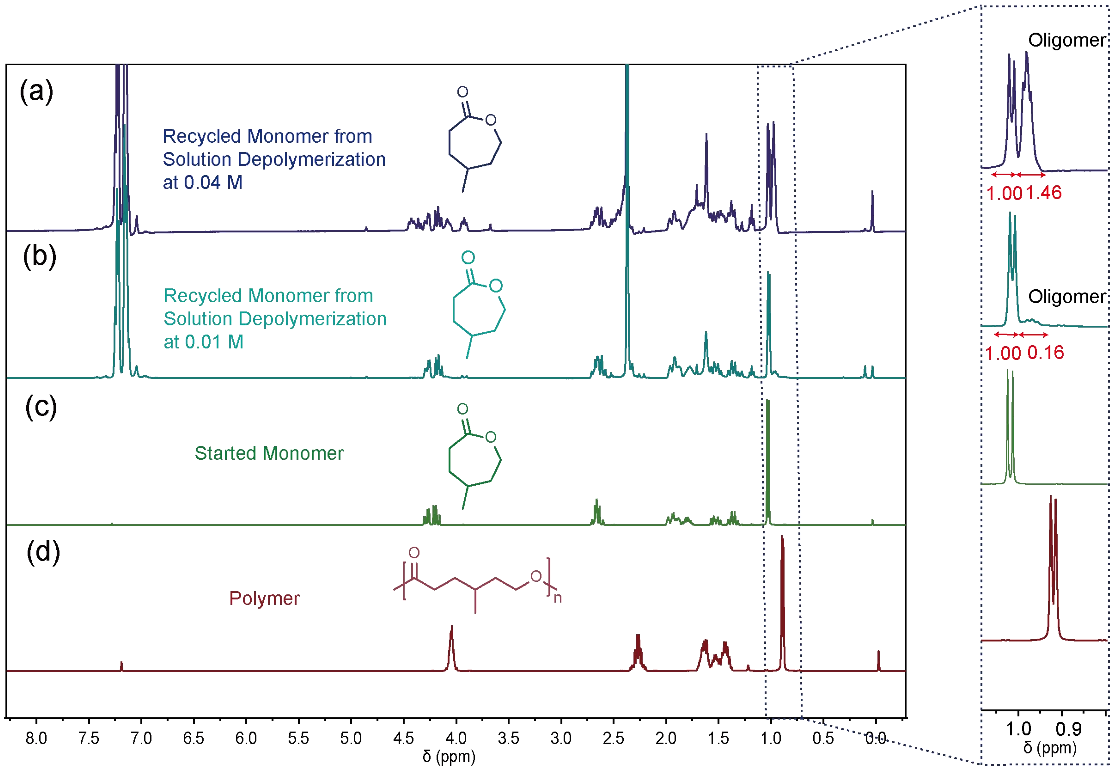

Supplementary Figure 127 Depolymerization study of P(**M1**) at different concentrations. ^{1}H NMR spectra of a) recycled **M1** by the solution depolymerization（**Supplementary Table 16** entry 3, monomer conversion = 1.00/(1.00 + 1.46) = 0.41), top; b) recycled **M1** by the solution depolymerization（**Supplementary Table 16** entry 2, monomer conversion = 1.00/ (1.00 + 0.16) = 0.86); c) starting **M1** for comparison, middle; d) P(**M1**) (M_n = 81.9 kg/mol, $Đ$ = 1.68), bottom.

Solution Depolymerization of P(**M2**)

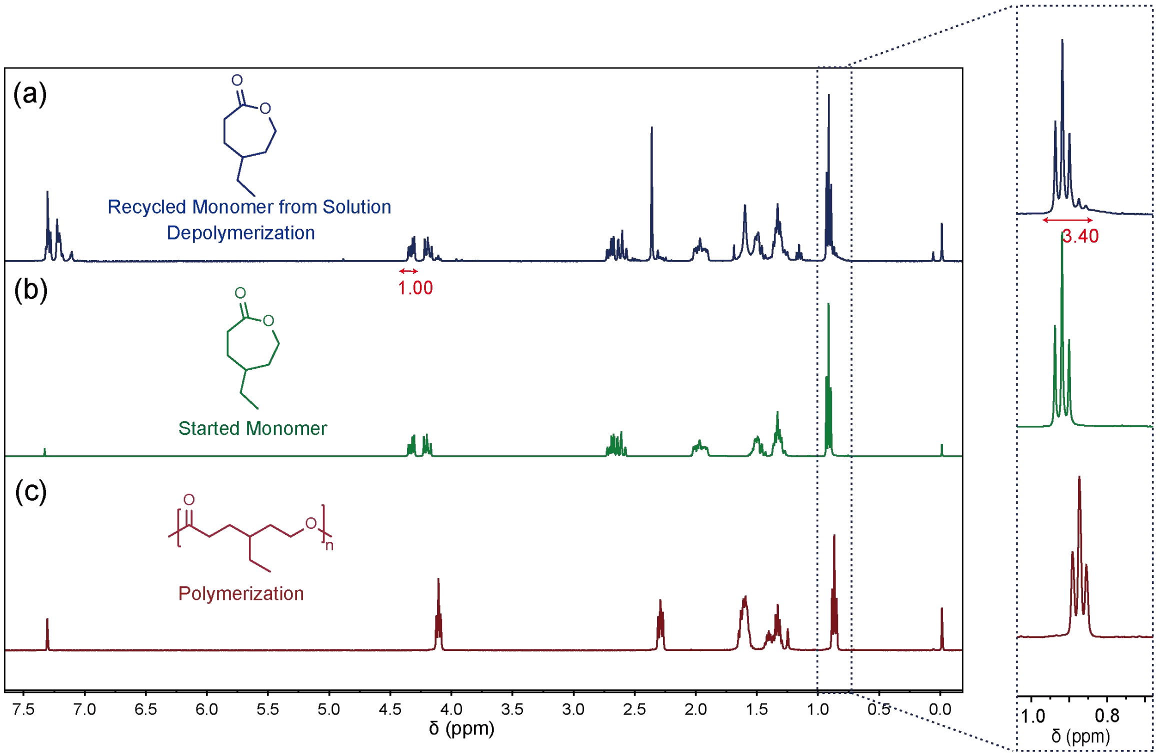

Supplementary Figure 128 ^{1}H NMR spectra of a) recycled **M2** by the solution depolymerization (Monomer conversion = 3 × 1.00/3.40 = 0.88), top; b) starting **M2** for comparison, middle; c) P(**M2**) (M_n = 158 kg/mol, $Đ$ = 1.48), bottom.

Solution Depolymerization of P(**M3**)

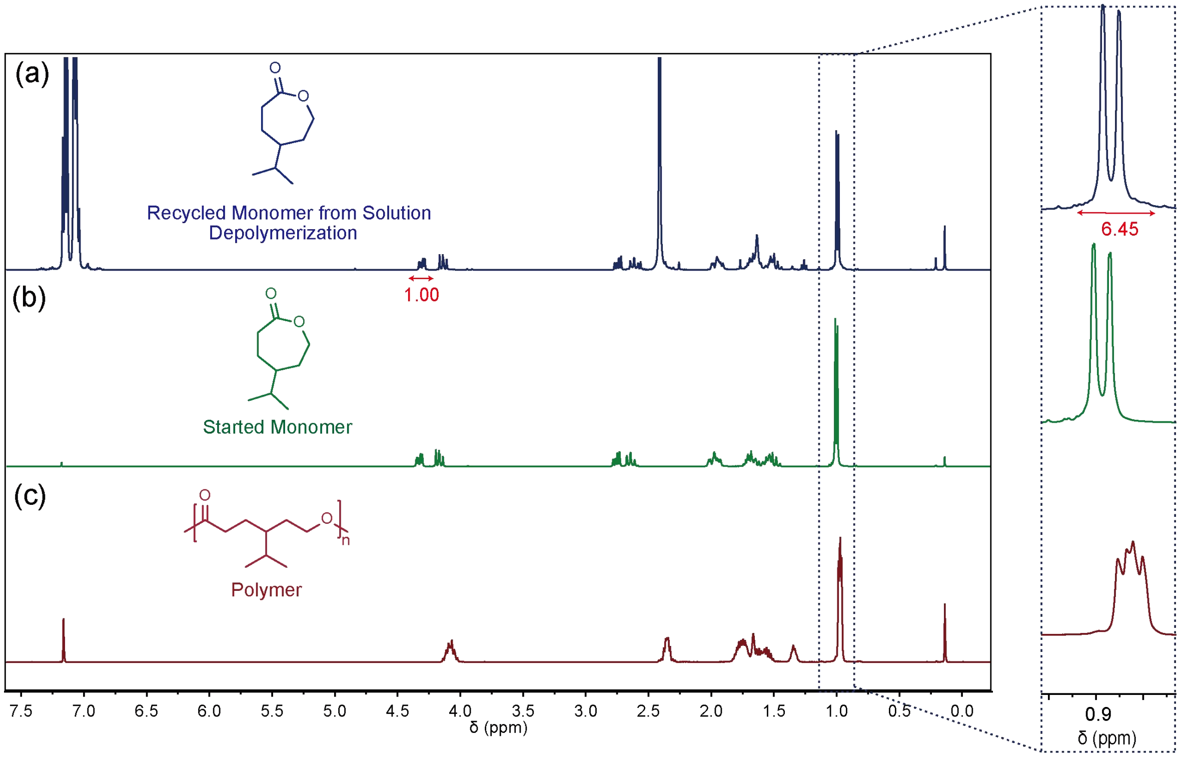

Supplementary Figure 129 ^{1}H NMR spectra of a) recycled **M3** by the solution depolymerization (Monomer conversion = 6 × 1.00/6.45 = 0.93), top; b) starting **M3** for comparison, middle; c) P(**M3**) (M_n = 92.3 kg/mol, $Đ$ = 1.75), bottom.

Solution Depolymerization of P(**M4**)

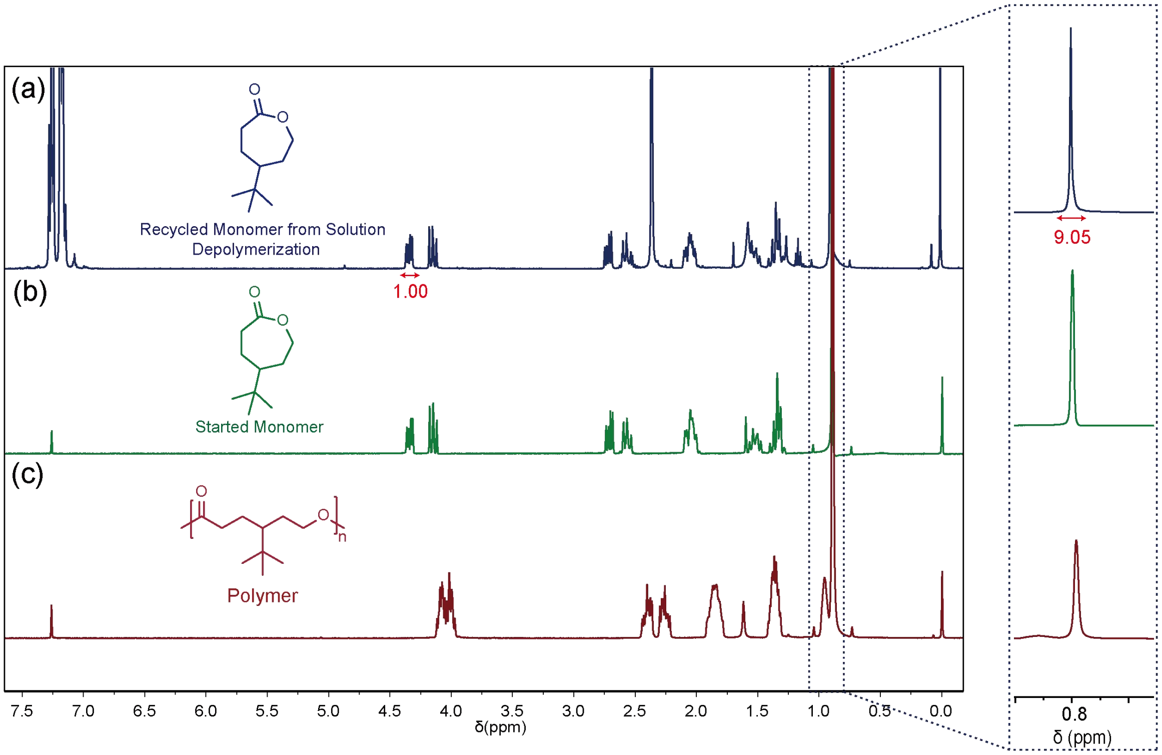

Supplementary Figure 130 ^{1}H NMR spectra of a) recycled **M4** by the solution depolymerization (Monomer conversion = 9 × 1.00/9.05 = 0.99), top; b) starting **M4** for comparison, middle; c) P(**M4**) (M_n = 42.9 kg/mol, $Đ$ = 1.36), bottom.

Solution Depolymerization of P(**M5**)

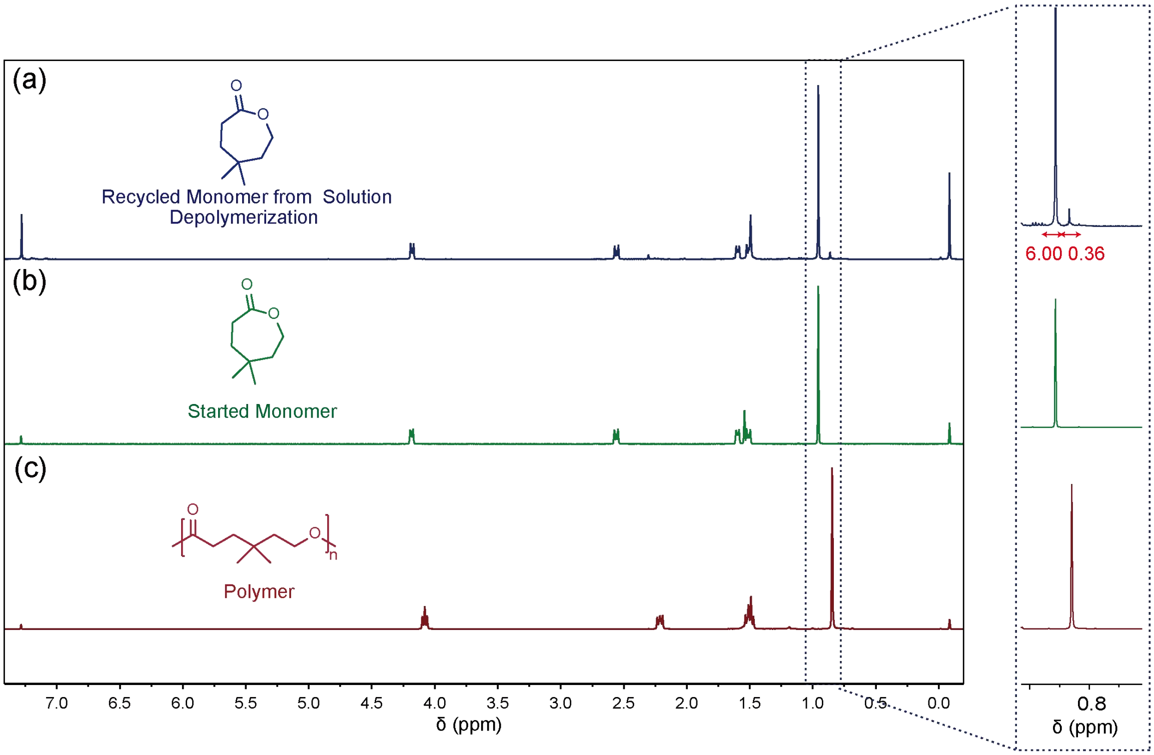

Supplementary Figure 131 ^{1}H NMR spectra of a) recycled **M5** by the solution depolymerization (Monomer conversion = 6.00/(6.00 + 0.36) = 0.94), top; b) starting **M5** for comparison, middle; c) P(**M5**) (M_n = 60.4 kg/mol, $Đ$ = 1.66), bottom.

Solution Depolymerization of P(**M6**)

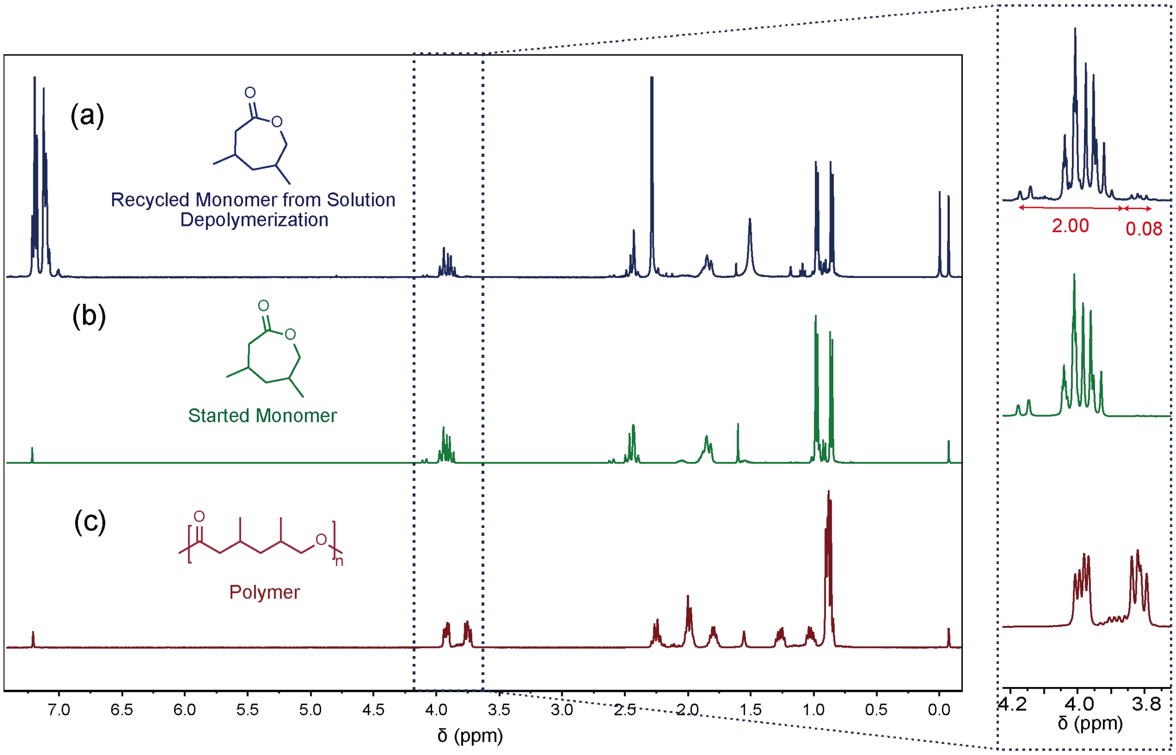

Supplementary Figure 132 ^{1}H NMR spectra of a) recycled **M6** by the solution depolymerization (Monomer conversion = 2.00/(2.00 + 0.08) = 0.96), top; b) starting **M6** for comparison, middle; c) P(**M6**) (M_n = 83.0 kg/mol, $Đ$ = 1.14), bottom.

Solution Depolymerization of P(**M8**)

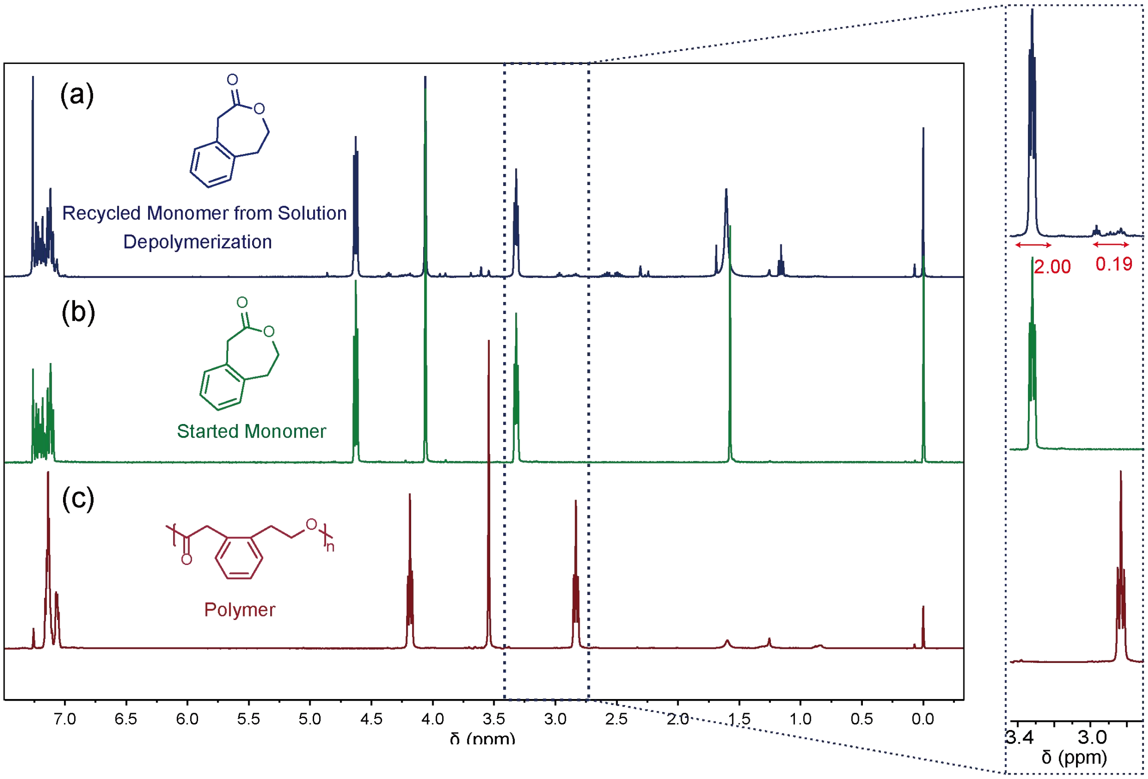

Supplementary Figure 133 ^{1}H NMR spectra of a) recycled **M8** by the solution depolymerization (Monomer conversion = 2.00/(2.00 + 0.19) = 0.91), top; b) starting **M8** for comparison, middle; c) P(**M8**) (M_n = 48.0 kg/mol, $Đ$ = 1.32), bottom.

Solution Depolymerization of P(**M9**)

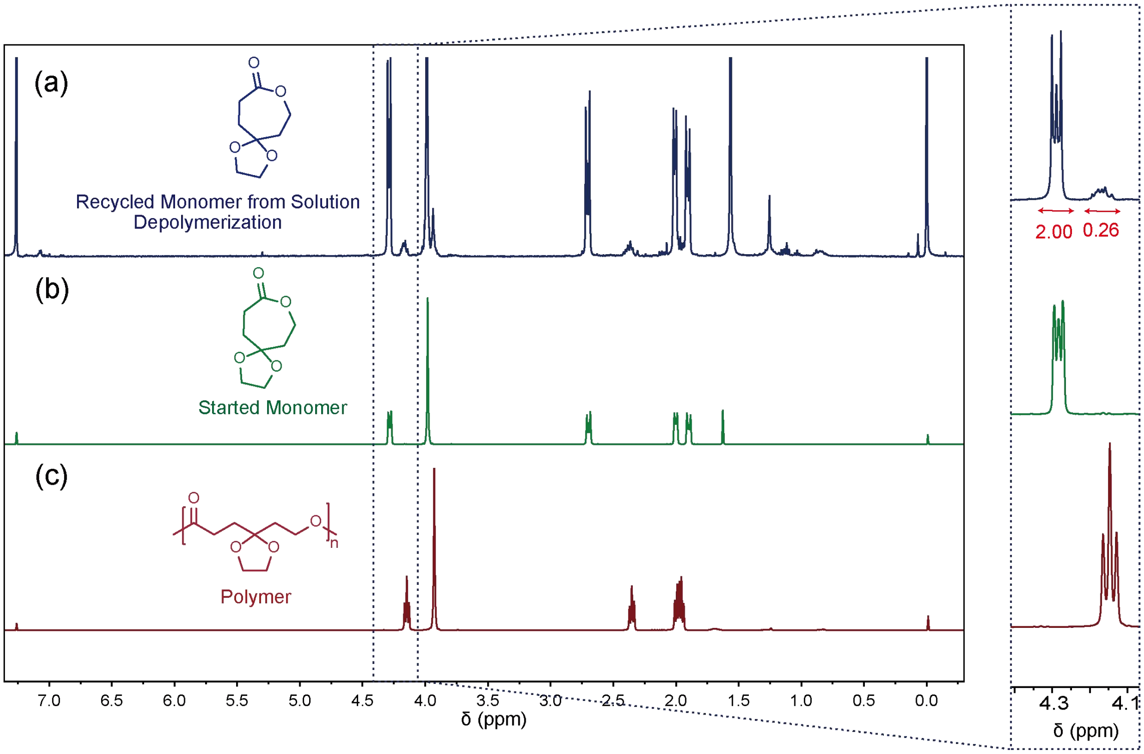

Supplementary Figure 134 ^{1}H NMR spectra of a) recycled **M9** by the solution depolymerization (Monomer conversion = 2.00/(2.00 + 0.26) = 0.88), top; b) starting **M9** for comparison, middle; c) P(**M9**) (M_n = 61.7 kg/mol, $Đ$ = 1.52), bottom.

Solution Depolymerization of P(**M10**)

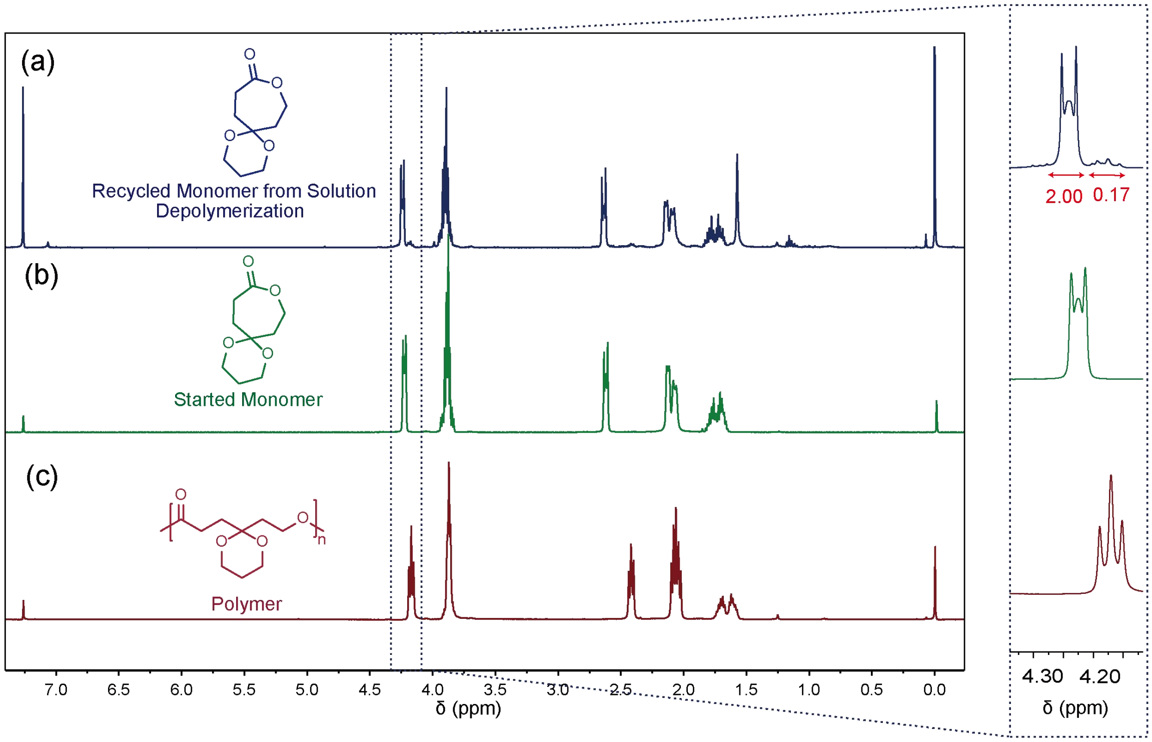

Supplementary Figure 135 ^{1}H NMR spectra of a) recycled **M10** by the solution depolymerization (Monomer conversion = 2.00/(2.00 + 0.17) = 0.92), top; b) starting **M10** for comparison, middle; c) P(**M10**) (M_n = 87.3 kg/mol, $Đ$ = 1.66), bottom.

Solution Depolymerization of P(**M11**)

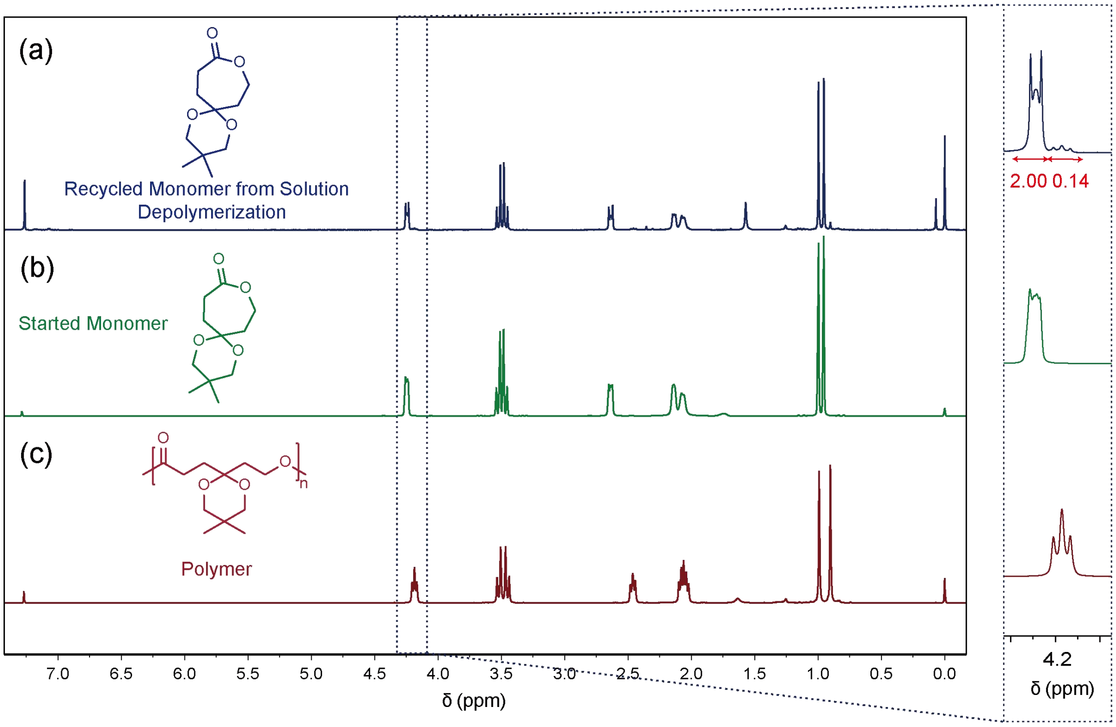

Supplementary Figure 136 ^{1}H NMR spectra of a) recycled **M11** by the solution depolymerization (Monomer conversion = 2.00/(2.00 + 0.14) = 0.93), top; b) starting **M11** for comparison, middle; c) P(**M11**) (M_n = 69.3 kg/mol, $Đ$ = 1.62), bottom.

Solution Depolymerization of P(**M12**)

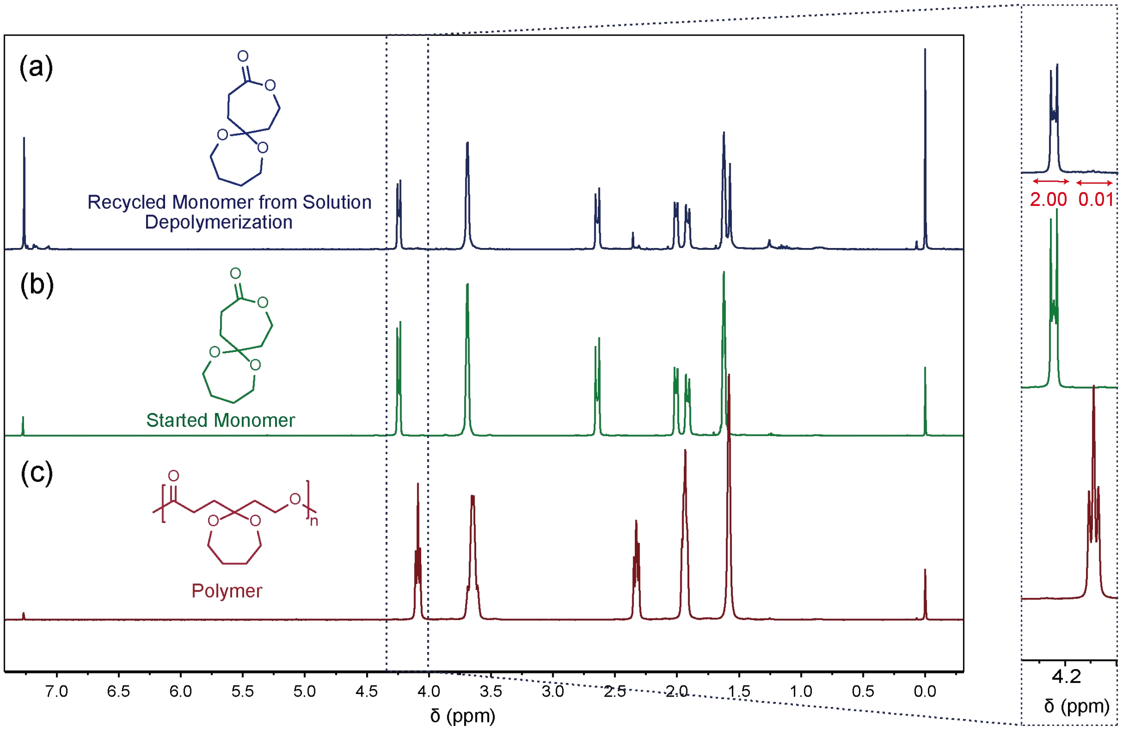

Supplementary Figure 137 ^{1}H NMR spectra of a) recycled **M12** by the solution depolymerization (Monomer conversion = 2.00/(2.00 + 0.01) = 0.99), top; b) starting **M12** for comparison, middle; c) P(**M12**) (M_n = 60.6 kg/mol, $Đ$ = 1.40), bottom.

Solution Depolymerization of P(**M13**)

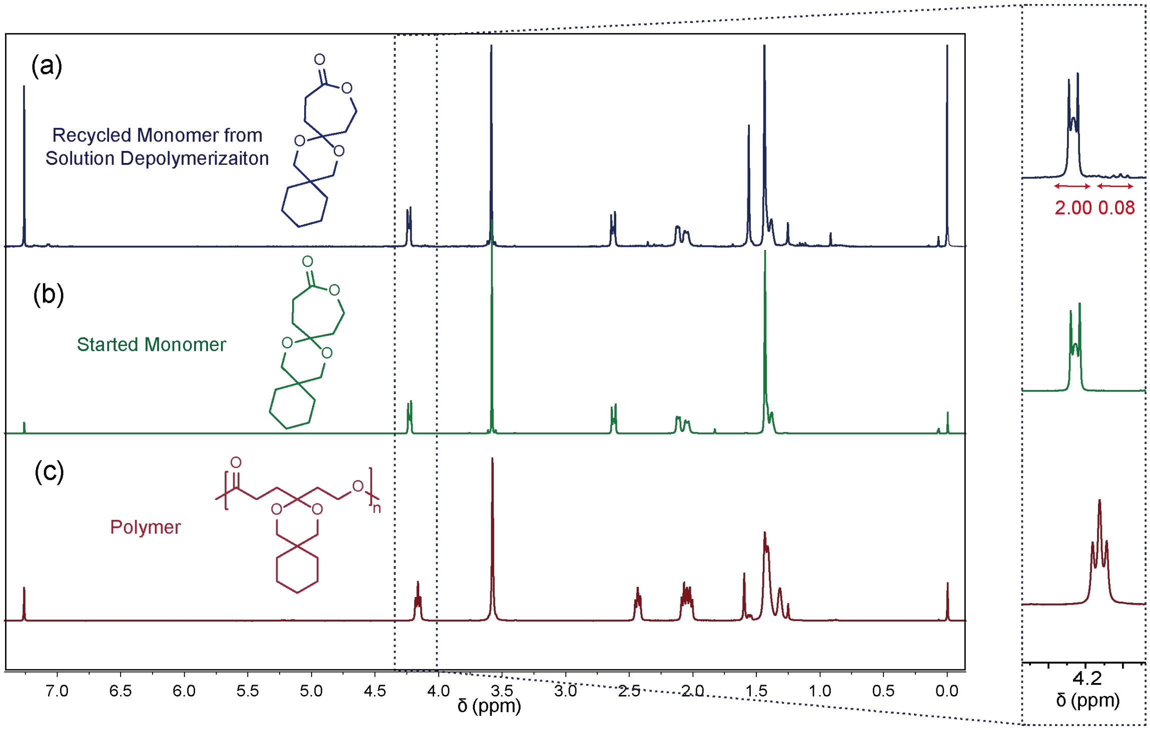

Supplementary Figure 138 ^{1}H NMR spectra of a) recycled **M13** by the solution depolymerization (Monomer conversion = 2.00/(2.00 + 0.08) = 0.96), top; b) starting **M13** for comparison, middle; c) P(**M13**) (M_n = 116 kg/mol, $Đ$ = 1.15), bottom.

Solution Depolymerization of P(**M14**)

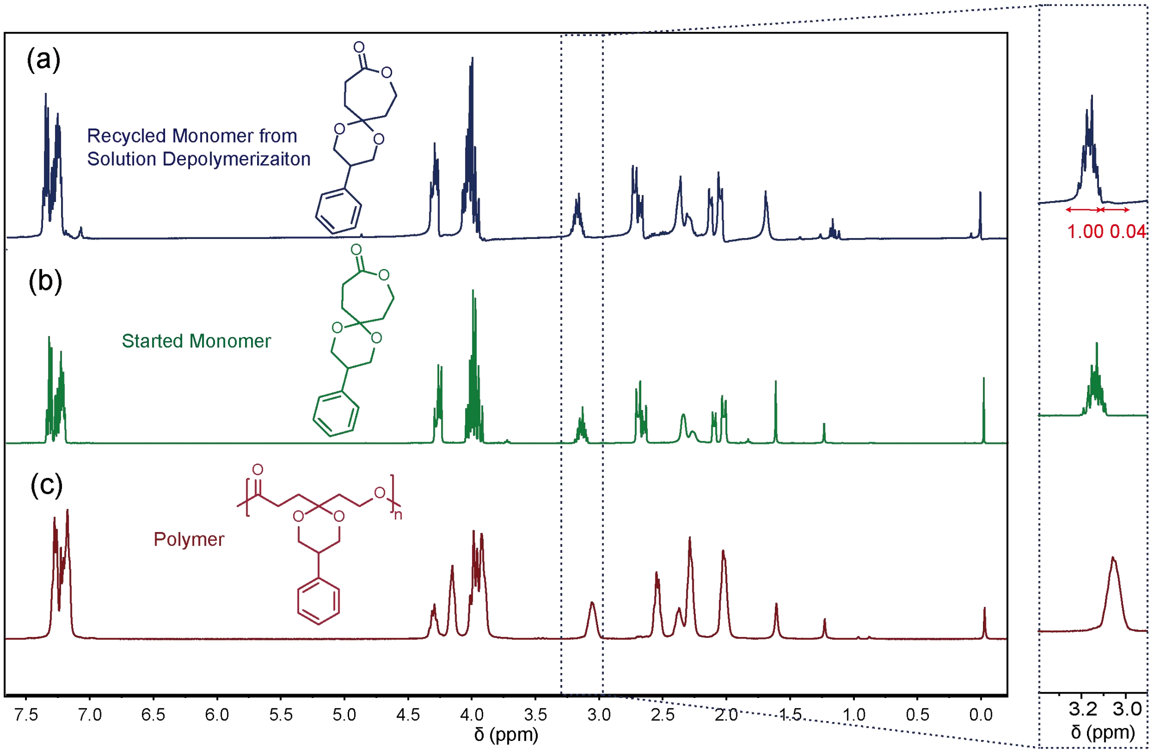

Supplementary Figure 139 ^{1}H NMR spectra of a) recycled **M14** by the solution depolymerization (Monomer conversion = 1.00/(1.00 + 0.04) = 0.96), top; b) starting **M14** for comparison, middle; c) P(**M14**) (M_n = 153 kg/mol, $Đ$ = 1.85), bottom.

Solution Depolymerization of P(**M16**)

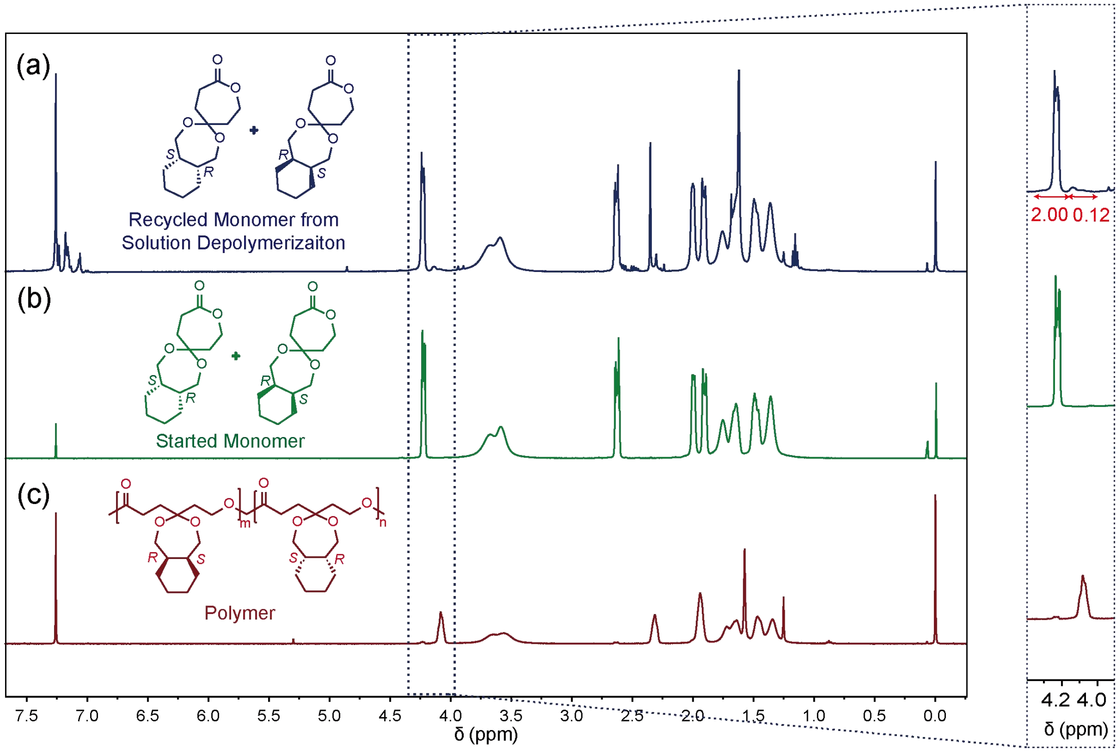

Supplementary Figure 140 ^{1}H NMR spectra of a) recycled **M16** by the solution depolymerization (Monomer conversion = 2/(2.00 + 0.12) = 0.94), top; b) starting **M16** for comparison, middle; c) P(**M16**) (M_n = 68.3 kg/mol, $Đ$ = 1.22), bottom.

General procedure for the CRM of polymers under bulk thermal.

PEO-10000 and polymer were mixed with the weight ratio of 4:1, and a catalytic amount of La[(N(SiMe$_3$)$_2$)$_3$] was added, and the mixture was kept at 160–180 °C with stirring in vacuo for a certain time. After the reaction was stopped, the sublimate was collected, weighted, and characterized by ^{1}H NMR spectroscopy.

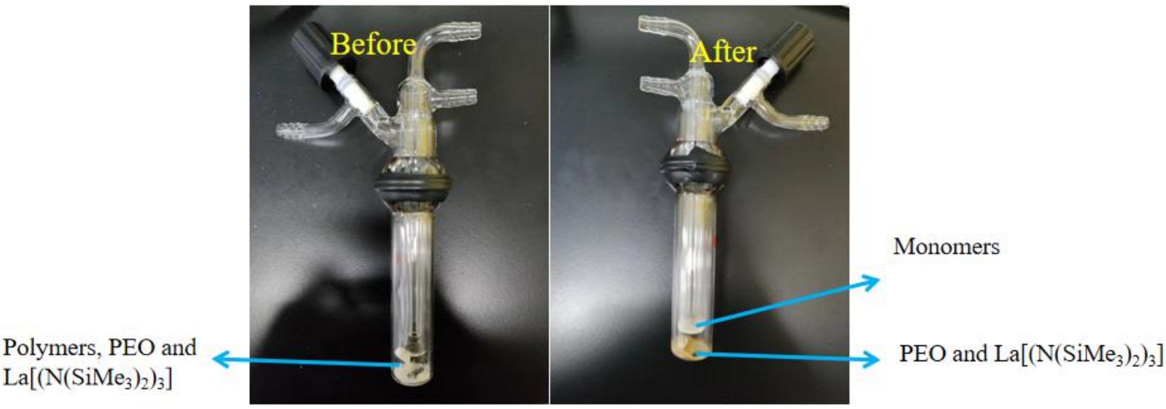

Supplementary Figure 141 Thermal depolymerization of polymers to monomers via sublimation.

Supplementary Table 17. Results of bulk thermal chemical recycling of P(**M**).[a]

Run	Polymer	Cat. (mol %)	Time (h)	T/°C	Monomer Yield[b] (%)
1	P(**M11**)	2	11	160	98
2	P(**M15**)	2	6	170	95
3	P(**M17**)	2	6.5	180	92

[a]Condition: all polymers were purified twice, catalyst: La[(N(SiMe$_3$)$_2$)$_3$]. [b]The monomer yield determined by the amount of the sublimate and the purity of recycled monomer determined by ^{1}H NMR spectroscopy. The cooling temperature was 0 °C. m_{PEO}:$m_{polymer}$ = 4:1.

Supplementary Table 18. Results of re-polymerization of recycled **M11** produced by bulk thermal chemical recycling.[a]

Run	M	[**M**]/[**Zn-1**]/[I]	Time (min)	Conv.[b] (%)	M_n[c] (kg/mol)	$Đ$[c] (M_w/M_n)
1	**M11**	500/1/1	15	94	86.3	1.28
2	Recycled **M11**[d]	500/1	15	86	136	1.44

[a]Condition: Catalyst = **Zn-1**, solvent = THF, RT. [b]Monomer conversion measured by ^{1}H NMR of the quenched solution. [c]Number-average molecular weight (M_n) and dispersity index ($Đ$ = M_w/M_n), determined by size exclusion chromatography (SEC) at 40 °C in THF. [d]Monomer was used from the depolymerized P[**M**] (500/1/1) under 160 °C.

Bulk Thermal Depolymerization of P(**M15**)

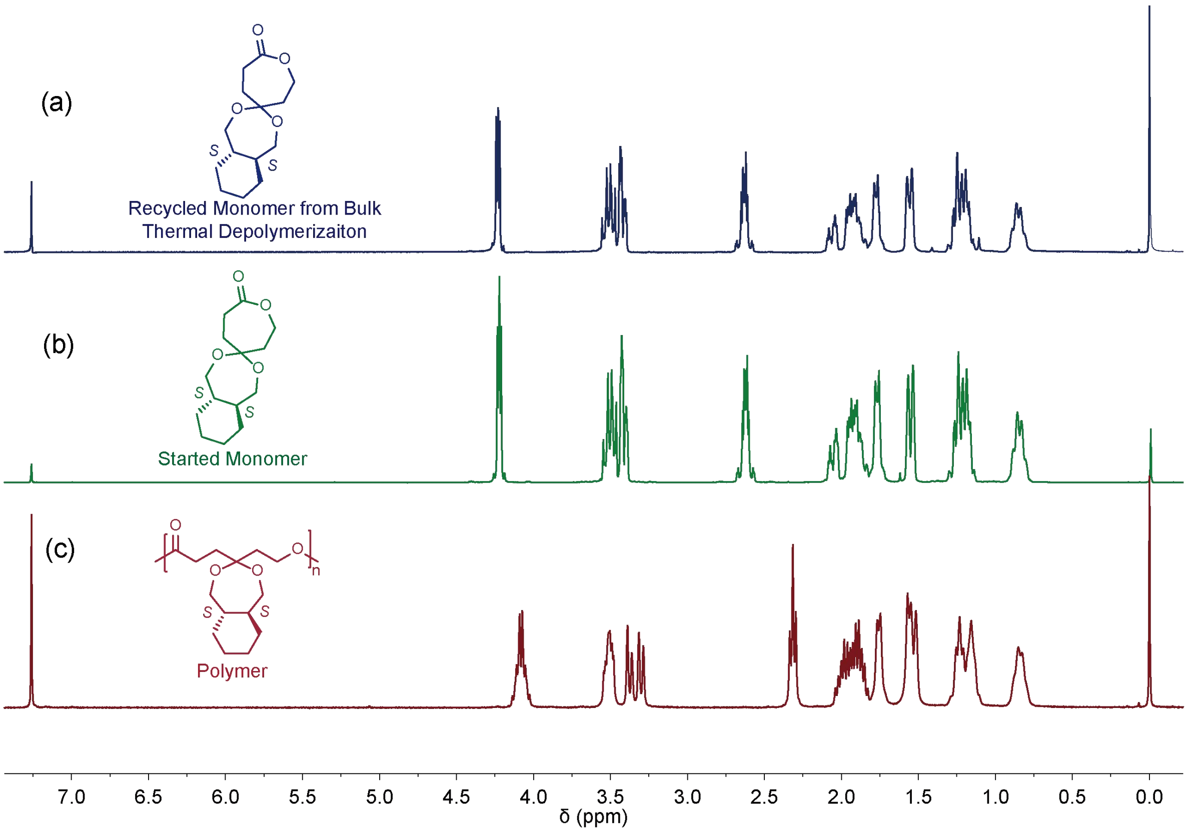

Supplementary Figure 142 ^{1}H NMR spectra of a) recycled **M15** by the bulk thermal depolymerization, top; b) starting **M15** for comparison, middle; c) P(**M15**) obtained by [**M15**]/[**Zn-1**]/[I] = 1000/1/1, bottom.

Bulk Thermal Depolymerization of P(**M17**)

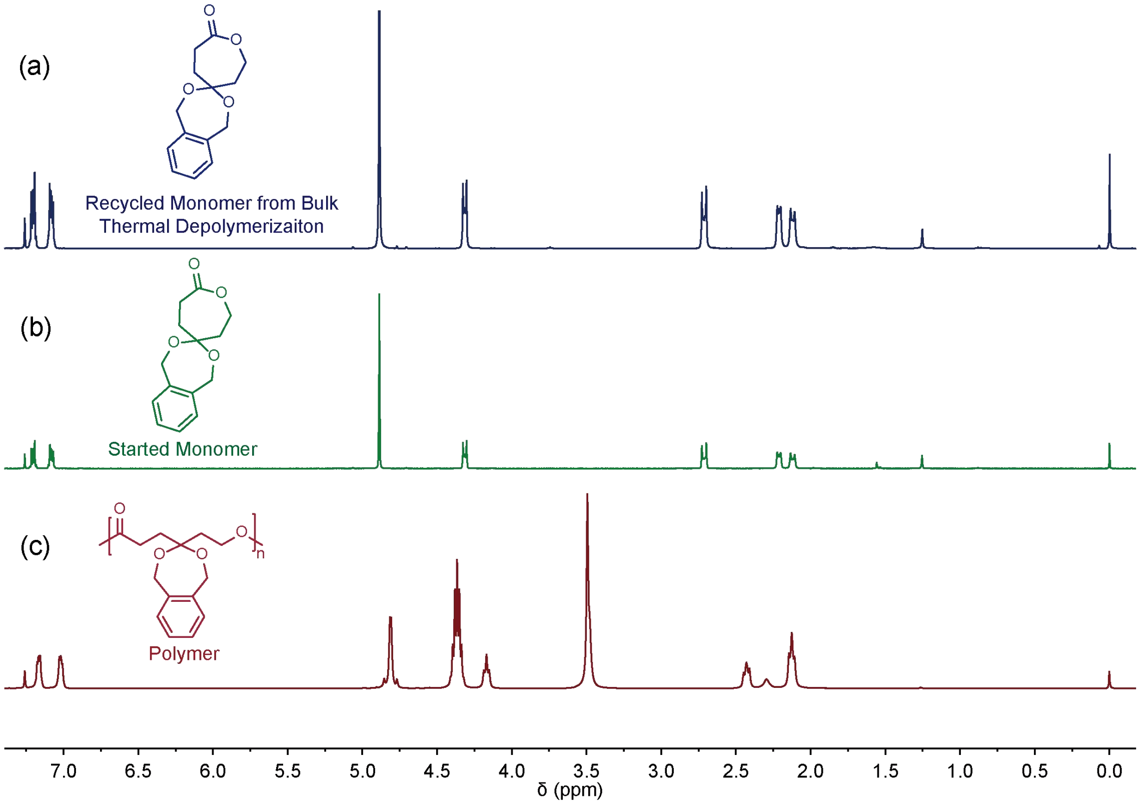

Supplementary Figure 143 ^{1}H NMR spectra of a) recycled **M17** by the bulk thermal depolymerization, top; b) starting **M17** for comparison, middle; c) P(**M17**) obtained by [**M17**]/[**Zn-1**]/[I] = 1000/1/1, bottom.

Mechanical Property

Summary of Mechanical Properties

Supplementary Table 19. Summary of mechanical properties of P(**M**)s.[a]

Entry	Polymers	$M_{n\text{-before}}$ (kg/mol)/ $Đ$	$M_{n\text{-after}}$(kg/mol)/ $Đ$	E/MPa	σ_Y /MPa	σ_B/MPa	Elongation/%
1	P(**M8**)	149/1.60	129/1.72	3.2±2.6	-[b]	4.5±1.6	1250±138
2	P(**M10**)	192/1.52	189/1.61	-[b]	-[b]	2.84±0.23	585±35
3	P(**M11)**	297/1.52	246/1.59	1010±100	15.7±0.8	14.2±2.2	257±19
4	P(**M12**)	197/1.62	178/1.71	2.2±1.1	-[b]	4.4±1.2	960±73
5	P(**M13**)	326/1.56	281/1.67	1155±105	34.7±3.6	18.5±1.2	141±23
6	P(**M14**)	278/1.25	241/1.32	1892±203	-[b]	49.8±5.0	4.1±0.3
7	P(**M16**)	249/1.57	224/1.68	1798±123	-[b]	35.2±0.7	2.4±0.2

[a]Condition: All polymers were obtained by [**M**]/[**Y-1**]/[I] = 2000/1/1 with a scale of 1.5–2 g monomer. And tested by uniaxial tensile tests. Strain rate of 20–100 mm/min, $M_{n\text{-before}}$: Before hot pressure, $M_{n\text{-after}}$: After hot pressure, E: Tensile modulus, σ_Y: yield strength, σ_B: break strength. [b]Not detected.

Extension test for P(**M8**)

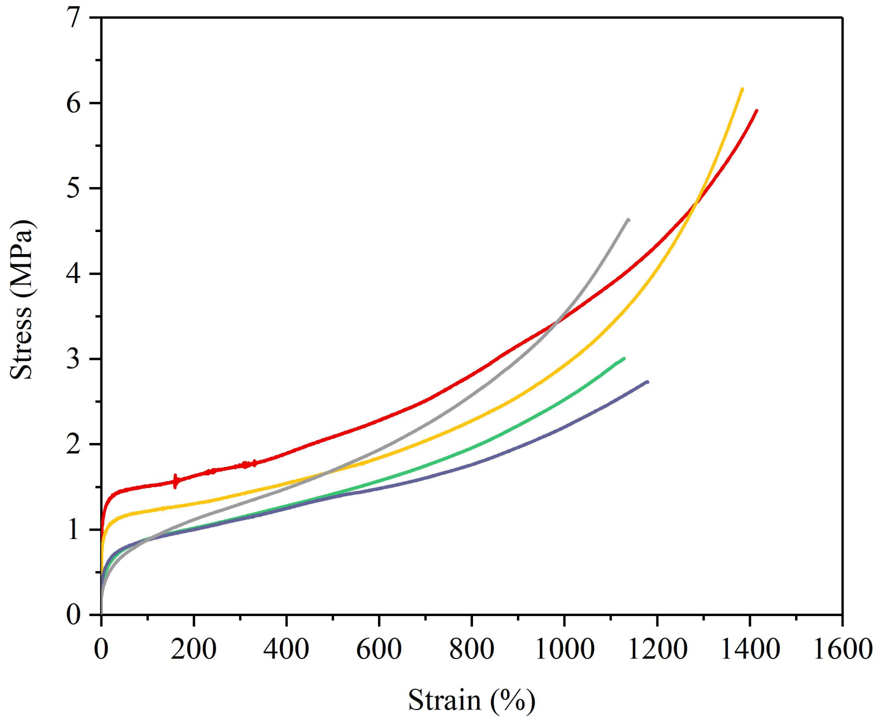

Supplementary Figure 144 Stress-strain curve of P(**M8**).

Supplementary Table 20. Summary of mechanical properties of P(**M8**).[a]

Entry	E/MPa	σ_Y /MPa	σ_B /MPa	Elongation/%
1	1.4	-[b]	6.2	1384
2	6.4	-[b]	5.9	1415
3	5.7	-[b]	2.7	1180
4	1.8	-[b]	2.9	1132
5	0.9	-[b]	4.6	1139
Average	3.2	-[b]	4.5	1250
S_{dev}	2.6	-[b]	1.6	138

[a]Condition: Tested by uniaxial tensile tests. Strain rate of 100 mm/min, E: Tensile modulus, σ_Y: yield strength. σ_B: break strength. [b]not detected.

Extension Test for P(**M10**)

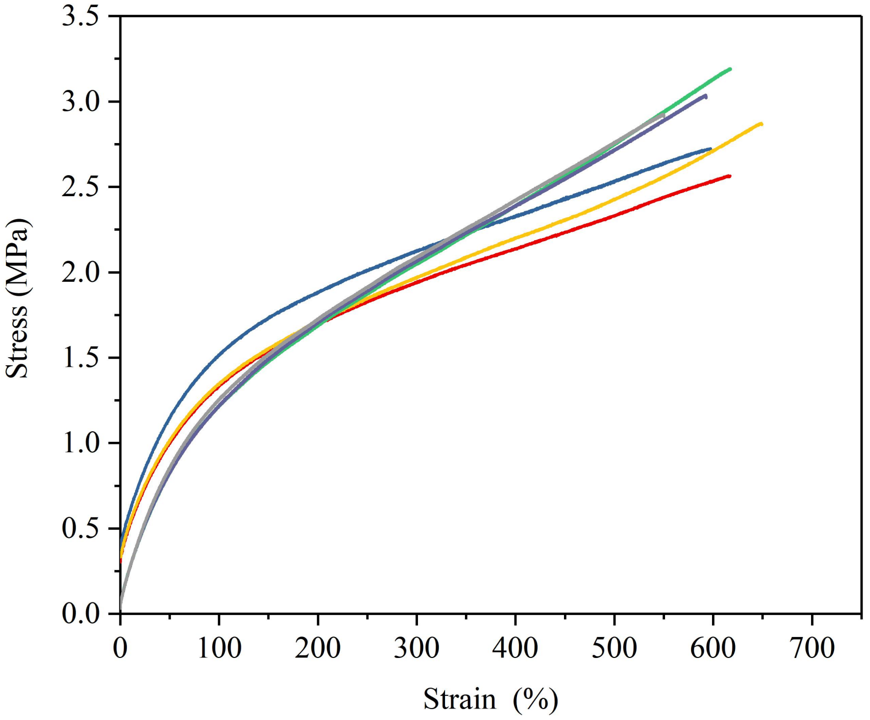

Supplementary Figure 145 Stress-strain curve of P(**M10**).

Supplementary Table 21. Summary of mechanical properties of P(**M10**).[a]

Entry	E/GPa	σ_Y /MPa	σ_B/MPa	Elongation/%
1	-[b]	-[b]	2.75	535
2	-[b]	-[b]	3.03	593
3	-[b]	-[b]	3.19	618
4	-[b]	-[b]	2.80	550
5	-[b]	-[b]	2.56	617
6	-[b]	-[b]	2.72	598
Average	-[b]	-[b]	2.84	585
S_{dev}	-[b]	-[b]	0.23	35

[a]Condition: Tested by uniaxial tensile tests. Strain rate of 100 mm/min, E: Tensile modulus, σ_Y: yield strength. σ_B: break strength. [b]not detected.

Extension test for P(**M11**)

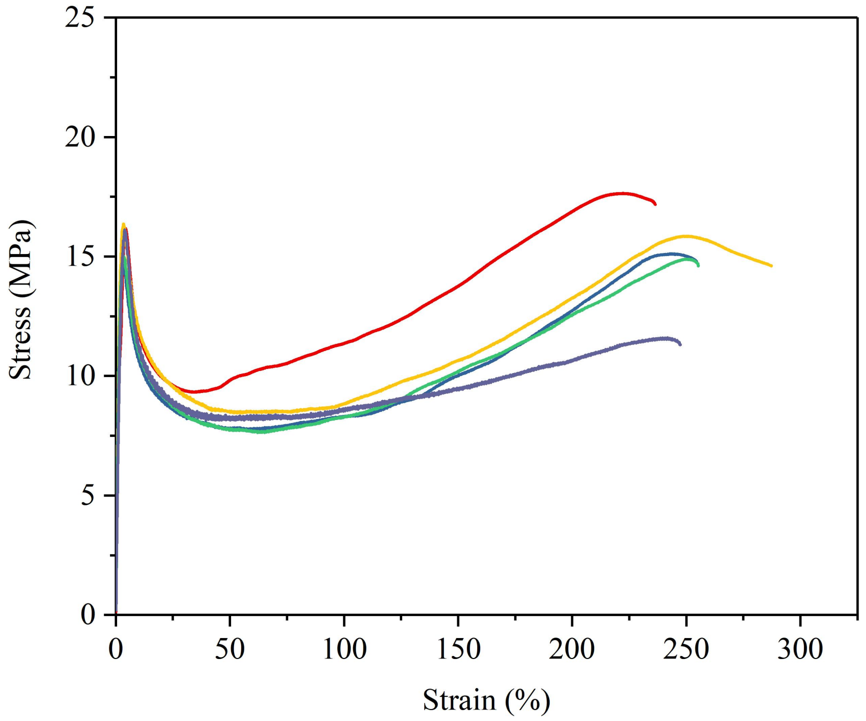

Supplementary Figure 146 Stress-strain curve of P(**M11**).

Supplementary Table 22. Summary of mechanical properties of P(**M11**).[a]

Entry	E/Ma	σ_Y /MPa	σ_B/MPa	Elongation/%
1	860	16.1	10.9	248
2	1060	16.4	14.6	288
3	970	14.6	14.7	255
4	1050	15.0	13.7	256
5	1120	16.2	17.1	236
Average	1010	15.7	14.2	257
S_{dev}	100	0.8	2.2	19

[a]Condition: Tested by uniaxial tensile tests. Strain rate of 20 mm/min, E: Tensile modulus, σ_Y: yield strength. σ_B: break strength.

Extension test for P(**M12**)

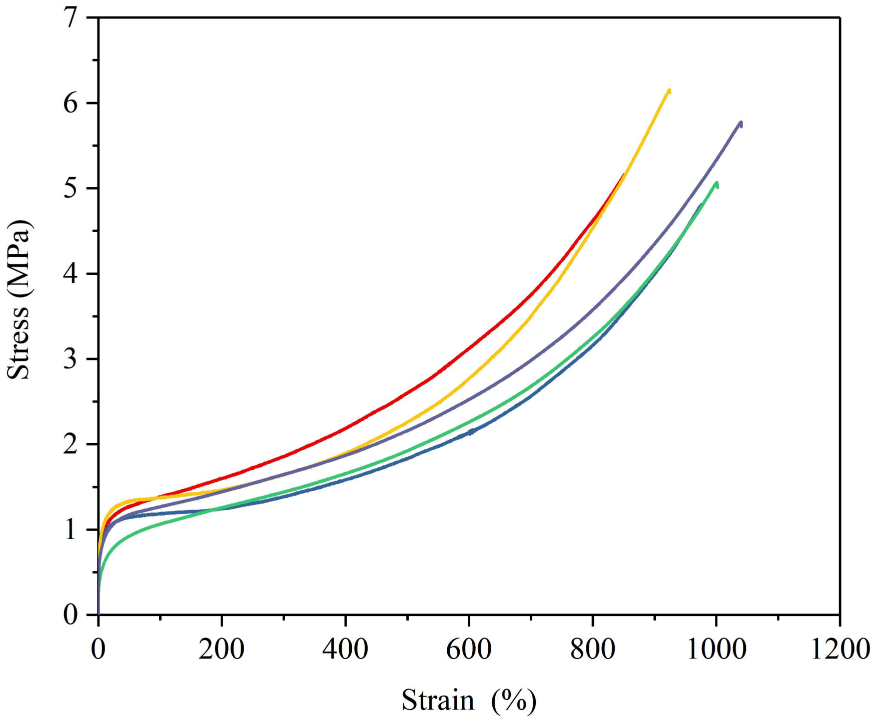

Supplementary Figure 147 Stress-strain curve of P(**M12**).

Supplementary Table 23. Summary of mechanical properties of P(**M12**).[a]

Entry	E/MPa	σ_Y /MPa	σ_B/MPa	Elongation/%
1	3.7	-[b]	5.2	852
2	1.4	-[b]	6.1	925
3	2.8	-[b]	4.8	980
4	1.1	-[b]	4.9	1003
5	1.8	-[b]	5.8	1040
Average	2.2	-[b]	4.4	960
S_{dev}	1.1	-[b]	1.2	73

[a]Condition: Tested by uniaxial tensile tests. Strain rate of 100 mm/min, E: Tensile modulus, σ_Y: yield strength. σ_B: break strength. [b]not detected.

Extension test for P(**M13**)

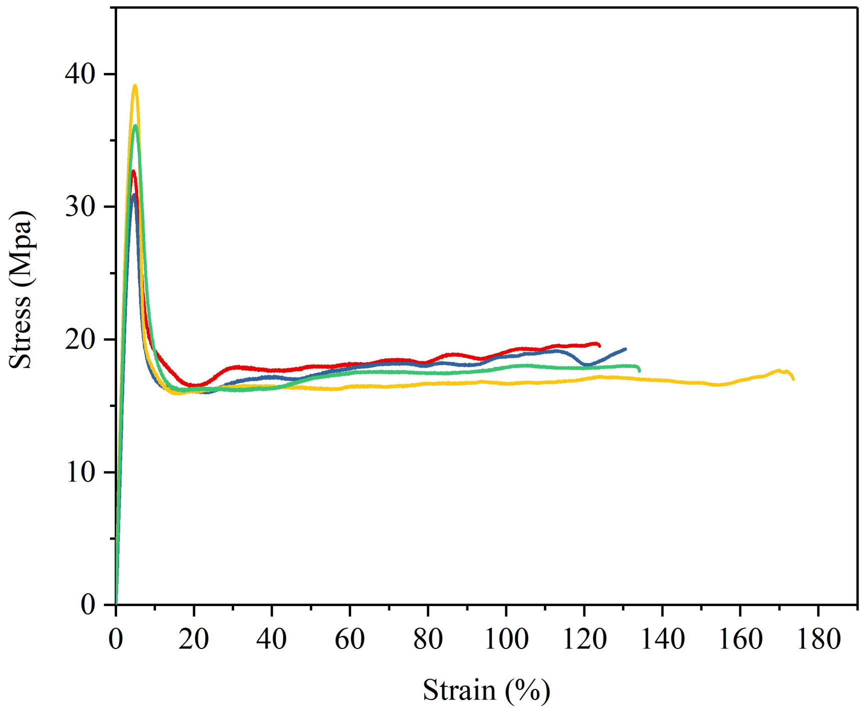

Supplementary Figure 148 Stress-strain curve of P(**M13**).

Supplementary Table 24. Summary of mechanical properties of P(**M13**).[a]

Entry	E/MPa	σ_Y /MPa	σ_B/MPa	Elongation/%
1	1292	39.1	17.1	175
2	1140	36.1	17.9	134
3	1152	32.6	19.6	124
4	1036	31.0	19.3	131
Average	1155	34.7	18.5	141
S_{dev}	105	3.6	1.2	23

[a]Condition: Tested by uniaxial tensile tests. Strain rate of 20 mm/min, E: Tensile modulus, σ_Y: yield strength. σ_B: break strength.

Extension test for P(**M14**)

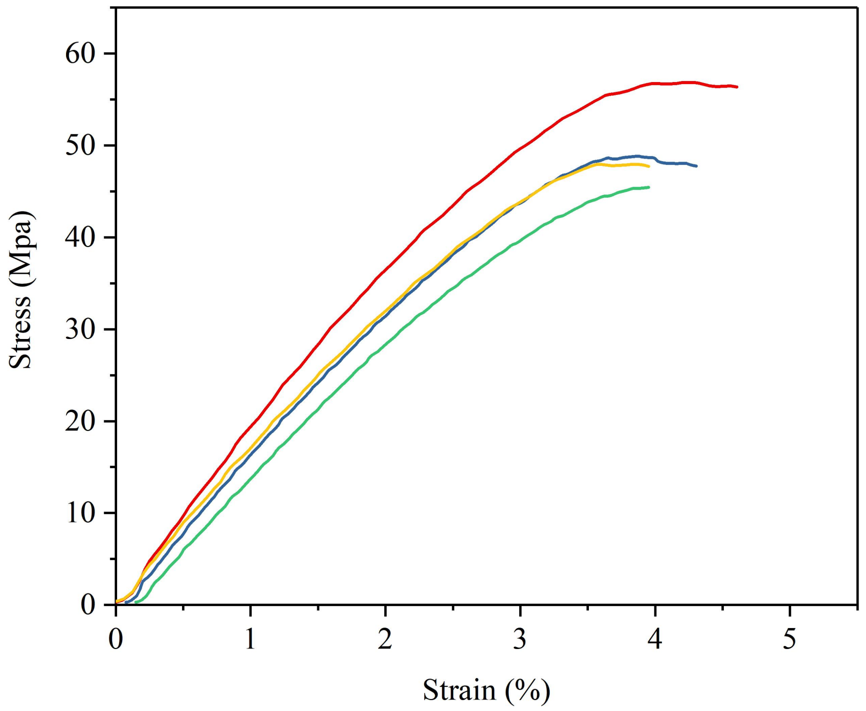

Supplementary Figure 149 Stress-strain curve of P(**M14**).

Supplementary Table 25. Summary of mechanical properties of P(**M14**).[a]

Entry	E/MPa	σ_Y/MPa	σ_B/MPa	Elongation/%
1	2153	-	56.9	4.5
2	1820	-	48.8	4.2
3	1671	-	45.4	4.0
4	1923	-	48.0	3.8
Average	1892	-[b]	49.8	4.1
S_{dev}	203	-[b]	5.0	0.3

[a]Condition: Tested by uniaxial tensile tests. Strain rate of 20 mm/min, E: Tensile modulus, σ_Y: yield strength. σ_B: break strength. [b]not detected.

Extension test for P(**M16**)

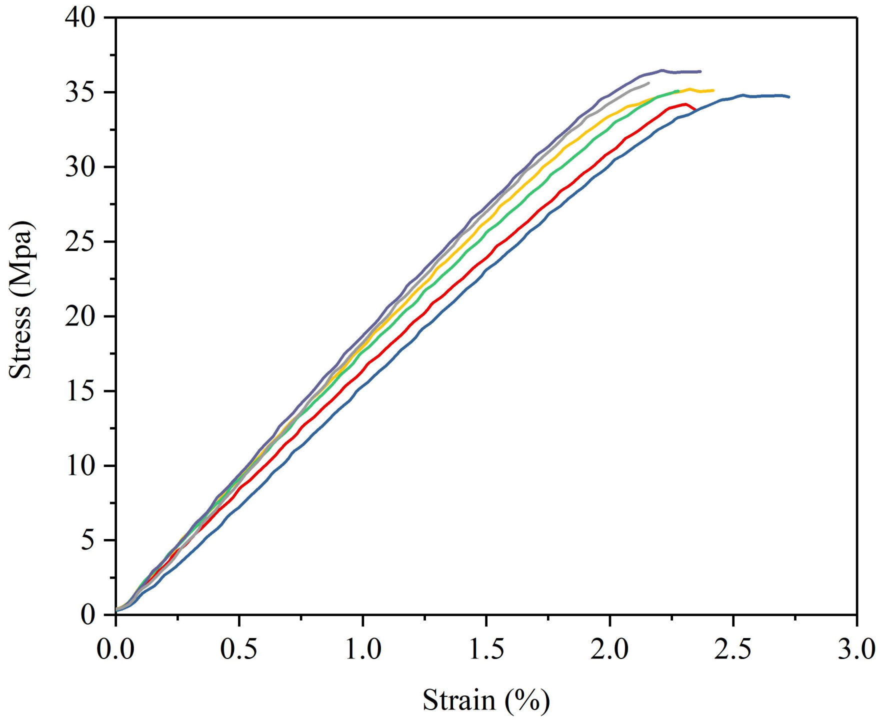

Supplementary Figure 150 Stress-strain curve of P(**M16**).

Supplementary Table 26. Summary of mechanical properties of P(**M16**).[a]

Entry	E/MPa	σ_Y /MPa	σ_B/MPa	Elongation/%
1	1677	-[b]	34.2	2.3
2	1615	-[b]	34.8	2.7
3	1861	-[b]	35.1	2.4
4	1821	-[b]	35.1	2.3
5	1911	-[b]	35.6	2.2
6	1902	-[b]	36.4	2.4
Average	1798	-[b]	35.2	2.4
S_{dev}	123	-[b]	0.7	0.2

[a]Condition: Tested by uniaxial tensile tests. Strain rate of 20 mm/min, E: Tensile modulus, σ_Y: yield strength. σ_B: break strength. [b]not detected.

Tensile testing of elastomers

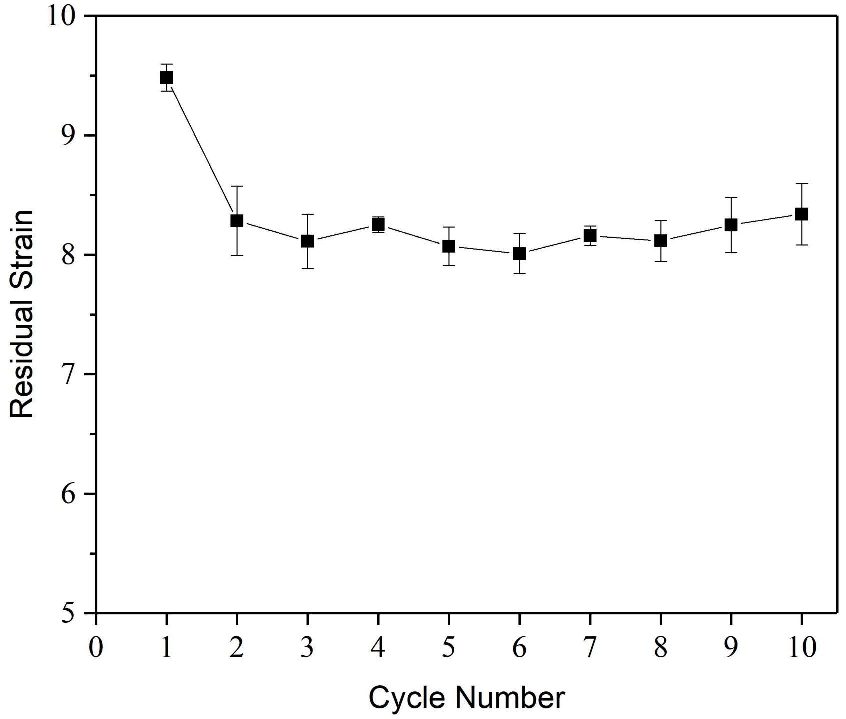

Supplementary Figure 151 Residual strain as a function of cycle number. Samples of P(**M8**) were extended to a maximum of 100% strain at a rate of 100 mm min^{-1}. Error bars represent a standard deviation of 3 specimens.

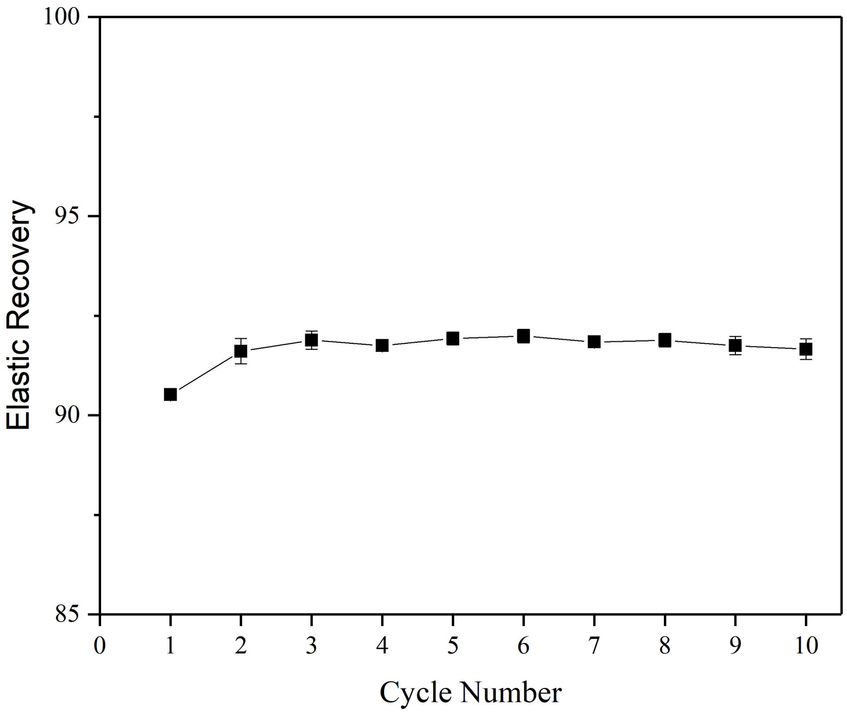

Supplementary Figure 152 Elastic recovery as a function of cycle number. Samples of P(**M8**) were extended to a maximum of 100% strain at a rate of 100 mm min^{-1}. Error bars represent a standard deviation of 3 specimens.

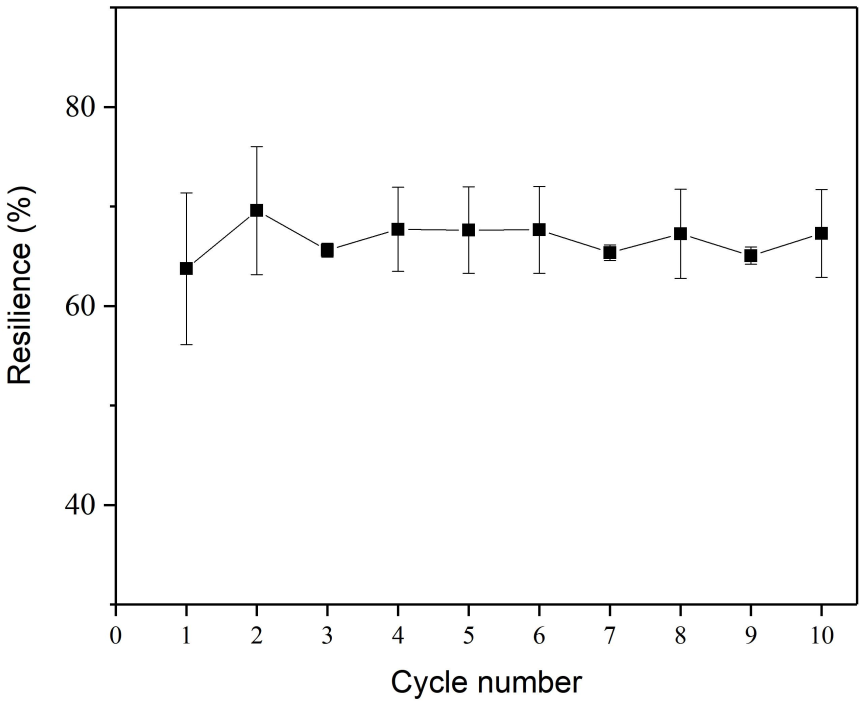

Supplementary Figure 153 Resilience as a function of cycle number. Samples of P(**M8**) were extended to a maximum of 100% strain at a rate of 100 mm min^{-1}. Error bars represent a standard deviation of 3 specimens.

Supplementary References

1 Shakaroun, R. M. *et al.* Stereoselective ring-opening polymerization of functional β-lactones: influence of the exocyclic side-group. *Polym. Chem.* **12**, 4022-4034, (2021).

2 Cheng, M. *et al.* Single-site beta-diiminate zinc catalysts for the alternating copolymerization of CO2 and epoxides: catalyst synthesis and unprecedented polymerization activity. *J. Am. Chem. Soc.* **123**, 8738-8749, (2001).

3 Kyte, B. G., Rouviere, P., Cheng, Q. & Stewart, J. D. Assessing the substrate selectivities and enantioselectivities of eight novel Baeyer-Villiger monooxygenases toward alkyl-substituted cyclohexanones. *J. Org. Chem.* **69**, 12-17, (2004).

4 Murphy, J. A. *et al.* Direct conversion of N-methoxy-N-methylamides (Weinreb amides) to ketones via a nonclassical Wittig reaction. *Org. Lett.* **7**, 1427-1429, (2005).

5 Chow, S. *et al.* Novel cuticular hydrocarbons from the cane beetle Antitrogus parvulus--4,6,8,10,16-penta- and 4,6,8,10,16,18-hexamethyldocosanes-unprecedented anti-anti-anti-stereochemistry in the 4,6,8,10-methyltetrad. *J. Org. Chem.* **70**, 1808-1827, (2005).

6 Ikeuchi, T. Curable composition with good adhesion. JP2015000909A (2015).

7 Zhang, X., Huang, K., Hou, G., Cao, B. & Zhang, X. Electron-donating and rigid P-stereogenic bisphospholane ligands for highly enantioselective rhodium-catalyzed asymmetric hydrogenations. *Angew. Chem. Int. Ed.* **49**, 6421-6424, (2010).

8 Lee, S. *et al.* Discovery of Octahydroindenes as PAR1 Antagonists. *ACS Med. Chem. Lett.* **4**, 1054-1058, (2013).

9 Harvey, R. G., Pataki, J., Cortez, C., Di Raddo, P. & Yang, C. X. A new general synthesis of polycyclic aromatic compounds based on enamine chemistry. *J. Org. Chem.* **56**, 1210-1217, (2002).

10 Kageyama, T., Kawahara, S., Kitamura, K., Ueno, Y. & Okawara, M. A Facile Oxidative Lactonization of 1,ω-Diols with Sodium Bromite. *Chem. Lett.* **12**, 1097-1100, (1983).

11 Zou, J., Yang, Y., Liu, Y., Chen, F. & Li, X. Release kinetics and cellular profiles for bFGF-loaded electrospun fibers: Effect of the conjugation density and molecular weight of heparin. *Polymer* **52**, 3357-3367, (2011).

12 Tu, Y. M. *et al.* Biobased High-Performance Aromatic-Aliphatic Polyesters with Complete Recyclability. *J. Am. Chem. Soc.* **143**, 20591-20597, (2021).
